# Assessing habitat connectivity of rare species to inform urban conservation planning

**DOI:** 10.1002/ece3.11105

**Published:** 2024-03-04

**Authors:** Eric M. McCluskey, Faith C. Kuzma, Helen D. Enander, Ashley Cole‐Wick, Michela Coury, David L. Cuthrell, Caley Johnson, Marianne Kelso, Yu Man Lee, Diana Methner, Logan Rowe, Alyssa Swinehart, Jennifer A. Moore

**Affiliations:** ^1^ Biology Department Grand Valley State University Allendale Michigan USA; ^2^ Michigan Natural Features Inventory Michigan State University Extension Lansing Michigan USA

**Keywords:** bees, Circuitscape, conservation, landscape ecology, reptiles, urban ecology

## Abstract

Urbanization is commonly associated with biodiversity loss and habitat fragmentation. However, urban environments often have greenspaces that can support wildlife populations, including rare species. The challenge for conservation planners working in these systems is identifying priority habitats and corridors for protection before they are lost. In a rapidly changing urban environment, this requires prompt decisions informed by accurate spatial information. Here, we combine several approaches to map habitat and assess connectivity for a diverse set of rare species in seven urban study areas across southern Michigan, USA. We incorporated multiple connectivity tools for a comprehensive appraisal of species‐habitat patterns across these urban landscapes. We observed distinct differences in connectivity by taxonomic group and site. The three turtle species (Blanding's, Eastern Box, and Spotted) consistently had more habitat predicted to be suitable per site than other evaluated species. This is promising for this at‐risk taxonomic group and allows conservation efforts to focus on mitigating threats such as road mortality. Grassland and prairie‐associated species (American Bumble Bee, Black and Gold Bumble Bee, and Henslow's Sparrow) had the least amount of habitat on a site‐by‐site basis. Kalamazoo and the northern Detroit sites had the highest levels of multi‐species connectivity across the entire study area based on the least cost paths. These connectivity results have direct applications in urban planning. Kalamazoo, one of the focal urban regions, has implemented a Natural Features Protection (NFP) plan to bolster natural area protections within the city. We compared our connectivity results to the NFP area and show where this plan will have an immediate positive impact and additional areas for potential consideration in future expansions of the protection network. Our results show that conservation opportunities exist within each of the assessed urban areas for maintaining rare species, a key benefit of this multi‐species and multi‐site approach.

## Graphical abstract

We combined several approaches to map habitat and assess connectivity for a diverse set of rare species in seven urban study areas across southern Michigan, USA. Our results show that conservation opportunities exist within each of the assessed urban areas for maintaining rare species, a key benefit of this multi‐species and multi‐site approach.

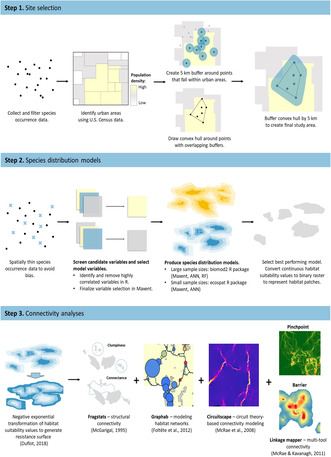

## 1 INTRODUCTION

Global trends in biodiversity largely reflect the pervasive negative impact expanding human populations and resource needs have had on a diverse array of taxa (Crist et al., 2017; Tilman et al., 2017). Humans have reshaped modern landscapes, to the overwhelming detriment of biodiversity (Díaz et al., 2019; Semenchuk et al., 2022). To persist in modern landscapes, species must adapt to mosaics of natural to heavily modified patches that vary in their connectedness and intensity of human use (Díaz et al., 2019). Identifying landscape conservation strategies that can effectively retain remnant biodiversity and minimize biodiversity losses, given the certainty of continued land conversion and use, is a vexing challenge (Hu et al., 2021). Contemporary urban landscapes offer an appropriate landscape context from which to explore such strategies and draw inferences for safeguarding biodiversity in other urbanizing areas.

Studying urban areas can provide detailed information on species distributions, habitat use, and connectivity for future landscape planning oriented around retaining biodiversity in these modified landscapes. Although the negative effects of urbanization on biodiversity are well known (Grimm et al., 2008; Kareiva et al., 2007), urban areas can support appreciable levels of biodiversity (Aronson et al., 2014). Species density in cities depends on factors like urban land cover, vegetative cover, and age of the city, and some (even endemic) biodiversity can be maintained if there exists a network of well‐connected natural habitat patches (Aronson et al., 2014; Beninde et al., 2015). Species responses will vary depending on the taxa present in an individual urban system (Ficetola et al., 2007). These responses can be quite nuanced and scale‐dependent. For example, Garden et al. (2010) found that habitat amount was important to terrestrial reptiles and small mammals but reptiles were more sensitive to forest patch size than mammals, which were more influenced by total forest habitat area. While population‐level responses and threats for individual taxa can be parsed within an urban system to guide focused conservation measures, some habitat management opportunities are more broadly applicable. Threlfall et al. (2017) showed that understory vegetation and native plants were associated with increased occupancy by bats, birds, and several insect groups. As urbanization is predicted to increase, and pristine landscapes become increasingly rare, urban landscapes have the potential to play a critical role in biodiversity conservation (Soanes & Lentini, 2019; Spotswood et al., 2021).

Identifying species that can tolerate and persist in these modified environments informs baseline biodiversity targets for urban conservation planning. Generally, rare vertebrates are more likely to be absent from modified landscapes when they have specialized habitat (Ordeñana et al., 2010) or diet (Evans et al., 2011) requirements, large body size, or home ranges (Bateman et al., 2012), or high incidence of conflict with humans (Riley et al., 2003). However, human settlements (including cities) are often located in or near areas of high primary productivity and high biological diversity (Balmford et al., 2001; Cincotta et al., 2000; Luck, 2007), thus many urban landscapes harbor rare, endemic plants (Schwartz et al., 2002) and animals (Aronson et al., 2014). For example, 30% of Australia's legally protected, threatened plant and animal species occur in cities – a higher percentage than in non‐urban areas (Ives et al., 2016).

Urban landscapes with rare species have higher biodiversity potential, but conservation planning must contend with the challenges of protecting rare species in these human‐dominated landscapes. Because much of the land area is already modified, urban landscapes contain limited potential for expanded habitat protection (Wang et al., 2018), making the protection of existing greenspaces critical. Furthermore, identifying and prioritizing key habitat patches that harbor rare species and corridors that may link those patches can promote natural movement and recolonization (e.g., Ossola et al., 2019), obviating the need for risky and expensive supplemental captive breeding or translocation programs. As urban areas are expected to experience more pronounced temperature changes due to climate change (potentially intensifying effects on urban species, Wilby & Perry, 2006), movement corridors that span urban–rural gradients represent a crucial means of passively facilitating climate‐mediated migration for urban species. Rare species are the most vulnerable to extirpation thereby making them a priority for conservation strategies focused on stemming biodiversity loss, and urban environments should not be overlooked when considering conservation and recovery solutions for these species.

We evaluated patterns of habitat availability and connectivity for a diverse set of rare animal species (two insects, three turtles, two snakes, one bird, and one bat) within four urban regions in Michigan, USA. We selected these species primarily because they are rare (varying from Michigan species of Special Concern to federally listed under the U.S. Endangered Species Act), yet populations still exist in urban areas. Our study objectives considered different aspects of rare species and their habitats in urban environments.

### 1.1 Species‐specific habitat and connectivity patterns

Our primary objective was to explore how this array of species persists in urban environments by mapping available habitat and connectivity. We were interested in habitat and connectivity patterns from a taxonomic perspective, using the aspects of species biology that contribute to rarity in urban environments to find conservation opportunities. Focal species vary in terms of their vagility, habitat specificity, and certain life history traits, and while all are threatened by habitat loss due to urban development, persistence in urban landscapes and sensitivity to its unique threats vary by species. For example, the turtles tend to be less vagile, yet dependent on both aquatic (living) and terrestrial (nesting) habitats, making them especially sensitive to road mortality. We predicted that their distributions would be less limited by suitable wetland or terrestrial habitats and more so by requisite habitat features (e.g., nesting habitat) and barriers to dispersal. The snakes tend to be more specialized in their habitats, whose remnant patches may be highly limited in these landscapes. Therefore, we predicted less available habitat for these reptiles than the more generalist turtle species (Blanding's and Box Turtles). The volant species – bees, birds, and bats – may have greater dispersal capabilities with less resistance to the intervening matrix, and therefore might persist in a network of smaller, more numerous, habitat patches distributed throughout urban landscapes. From a habitat perspective, we predicted the species reliant on grassland/prairie habitat would have the least amount of habitat in urban environments as these habitats are often well‐suited for housing or other developed space.

We believe applying several different approaches for estimating connectivity provides the best opportunity to understand potential responses to urbanization from this set of species. Each urban region we analyzed harbors at least three of the focal species and obtaining several perspectives of landscape permeability should improve our ability to identify connectivity corridors that benefit multiple species. Our specific aims were to determine how fragmented remaining habitats are for these rare species and map potential movement corridors along an urban–rural gradient. We then compared connectivity patterns among co‐occurring species to identify any existing multi‐species linkages.

### 1.2 Protected areas

We assumed protected areas play a prominent role in both habitat availability and promoting movement across these modified landscapes based on our experience working in State Game Areas adjacent to several of these urban regions. We assessed the importance of protected areas across the urban–rural gradient by calculating the total protected area at each study site and estimated the amount of protected habitat for each resident rare species. We predicted that a disproportionate amount of habitat would be located in protected areas and higher connectivity observed in the space peripheral to urban areas at each study site.

### 1.3 Applied case study with Kalamazoo

Our final study objective focused on the Kalamazoo urban region because the city has recently implemented a long‐term program aimed at improving natural area protections within the urban limits (see Section 2 for full description). Here, we were interested in evaluating how an ongoing effort is contributing to connectivity and assessing how our analysis could complement the city's initiative. Urban natural area conservation efforts are most likely to focus on safeguarding greenspaces irrespective of where rare species are found. Therefore, we predicted that the city's protected area plan may not encompass the full extent of available habitat, and that gaps in coverage may be species‐specific. We believe the species‐specific habitat and connectivity results can guide targeted rare species efforts in Kalamazoo and other urban areas with similar programs by identifying key habitat corridors and opportunities for future landscape protections. We aimed to present this case study as a real‐world application of our habitat and connectivity analyses.

## 2 MATERIALS AND METHODS

We selected nine focal species representing several taxonomic groups that are regionally rare (Table 1). Species occurrences were sourced from multiple databases including the Michigan Natural Heritage Database maintained by the Michigan Natural Features Inventory (MNFI), U.S. Fish & Wildlife Service (USFWS), Herp Mapper (Herpmapper, 2022), iNaturalist (www.inaturalist.org), and eBird (www.ebird.org). Data quality control measures included using verified MNFI records wherever possible and iNaturalist Research Grade records. We did not include occurrences that were older than the year 2000. We mapped protected areas by merging all federal, state, county, local, and non‐governmental (NGO) managed areas from an MNFI GIS layer delineating natural areas, waterways, and green spaces. We omitted disturbed greenspaces such as golf courses from our analysis.

**TABLE 1:** Rare focal species, minimum dispersal distances (best estimates of the distances adult individuals are capable of moving between populations), and minimum viable habitat area (smallest patch size capable of supporting a population).

Species	Dispersal distance (km)	References (dispersal)	Minimum viable habitat (ha)	References (habitat)
American Bumble Bee^1^ (*Bombus pensylvanicus*)	10	Kraus et al. (2009), MacPhail et al. (2019)	5	20% threshold of our data
Black and Gold Bumble Bee^1^ (*Bombus auricomus*)	10	Kraus et al. (2009), MacPhail et al. (2019)	4	20% threshold of our data
Blanding's Turtle^1,4^ (*Emydoidea blandingii*)	2	MNFI/NatureServe	10	MNFI/Natureserve
Eastern Box Turtle^1,4^ (*Terrapene carolina carolin*a)	1–2	Laarman (2017), Iglay et al. (2007), Donaldson and Echternacht (2005)	5	Laarman (2017), Kapfer et al. (2013), Donaldson and Echternacht (2005)
Eastern Massasauga^1,4,5^ (*Sistrurus catenatus*)	1–2	MNFI/NatureServe	5	20% threshold of our data
Eastern Foxsnake^2,4^ (*Pantherophis gloydi*)	1.5	Row et al. (2012)	14	20% threshold of our data, Row et al. (2012)
Henslow's Sparrow^3,4^ (*Ammodramus henslowii*)	1	Williams and Boyle (2018), for Grasshoper Sparrow	30	Herkert (2019), Cooper (2012)
Northern Long‐eared Bat^1,4,5^ (*Myotis septentrionalis*)	5	MNFI/NatureServe	61.1	MNFI/NatureServe
Spotted Turtle^2,4^ (*Clemmys gutatta*)	1	MNFI/NatureServe, Chandler et al. (2019), Milam and Melvin (2001), Harding (1997)	5	MNFI/NatureServe, Rasmussen and Litzgus (2010)

*Note*: See Graphab methods for species‐specific details of minimum viable habitat patch size determination. Conservation status is shown for the Michigan state listing as Special Concern^1^, Threatened^2^, Endangered^3^, Midwest Species of Greatest Conservation Need^4^ or federal listing^5^.

**FIGURE 1:**
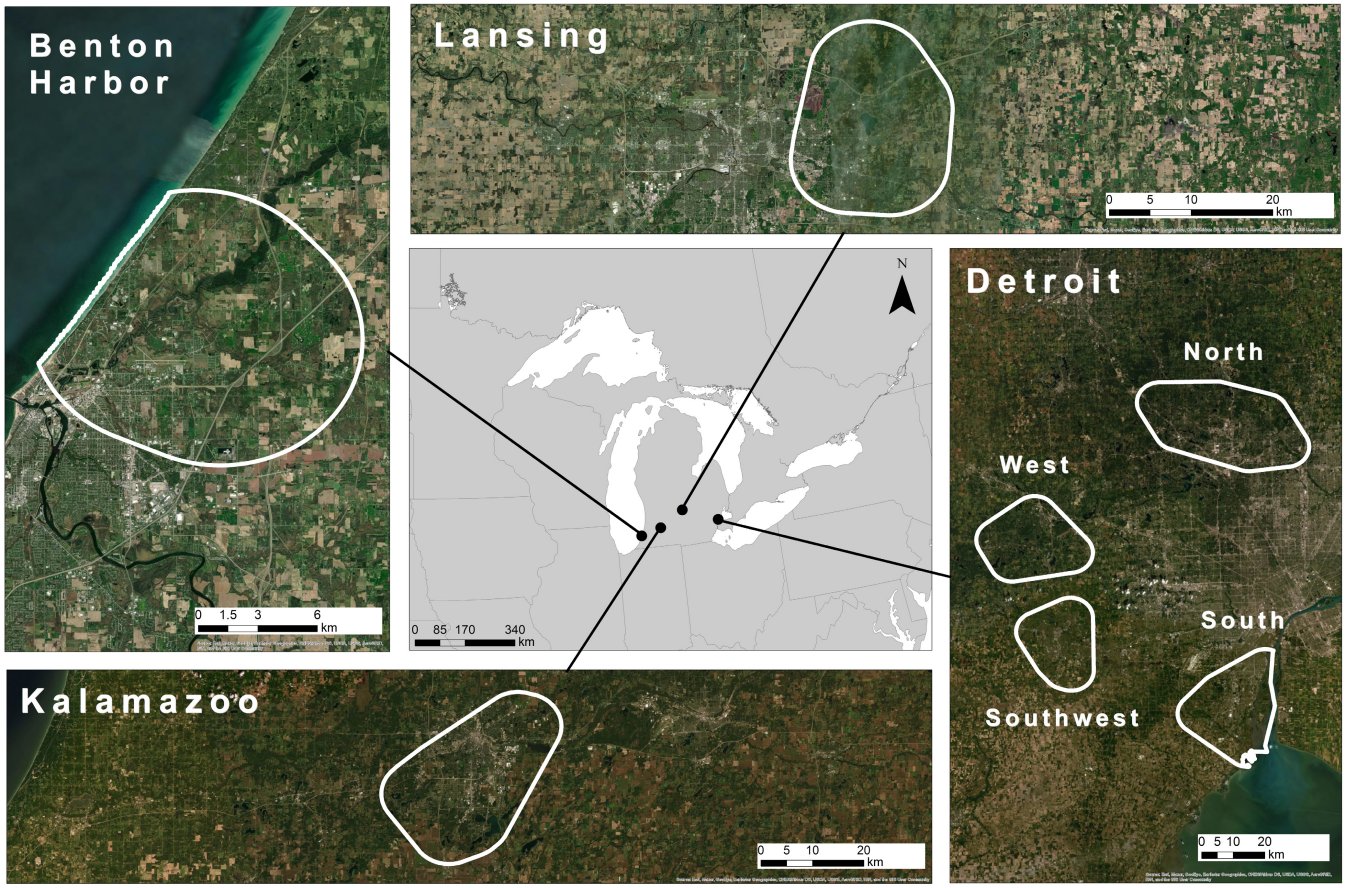
Map of the seven urban study areas across southern Michigan, USA.

We used U.S. Census criteria based on human population and density thresholds for defining urban regions in Michigan. The Census Bureau considers an area with 50,000 or more people as urbanized (Ratcliffe et al., 2016). From a density perspective, census blocks with 1000 people per square mile (ppsm) are considered urban while surrounding blocks that are 500 ppsm may also be considered part of the broader urban area representing residential and other urban associated land use (Ratcliffe et al., 2016). These criteria fit our research aims well as we were most interested in evaluating habitat and protected areas that are embedded within urban areas along a modified urban–rural gradient. Therefore, we treated 500 ppsm areas surrounding 1000 ppsm cores within broader landscapes that exceed 50,000 people as urban in our site selection process. We applied the density threshold at the tract level using available 2020 Census data (https://mtgis‐portal.geo.census.gov/arcgis/apps/MapSeries/index.html?appid=2566121a73de463995ed2b2fd7ff6eb7), selecting 1000 ppsm tracts and all surrounding contiguous 500 ppsm tracts in a Michigan tract‐level shapefile with ArcMap (vers. 10.8). We dissolved the contiguous >500 ppsm tracts for each urban region and then determined if they exceeded 50,000 people. The same conservation issues concerning habitat loss and fragmentation are present in areas with fewer than 50,000 people but our research focus was specifically on the more urban regions in Michigan that still support rare species. We further limited sites based on the number of rare species within the dissolved urban tract boundary, only selecting urban regions with presumed extant populations (with recent occurrence records) for at least three of our nine target species. This focused the analysis on areas with greater potential for supporting multi‐species corridors. Four urban regions (Benton Harbor/St. Joseph/Fair Plain; Kalamazoo; Lansing; Detroit) matched our U.S. census and species criteria (Figure 1).

**FIGURE 2:**
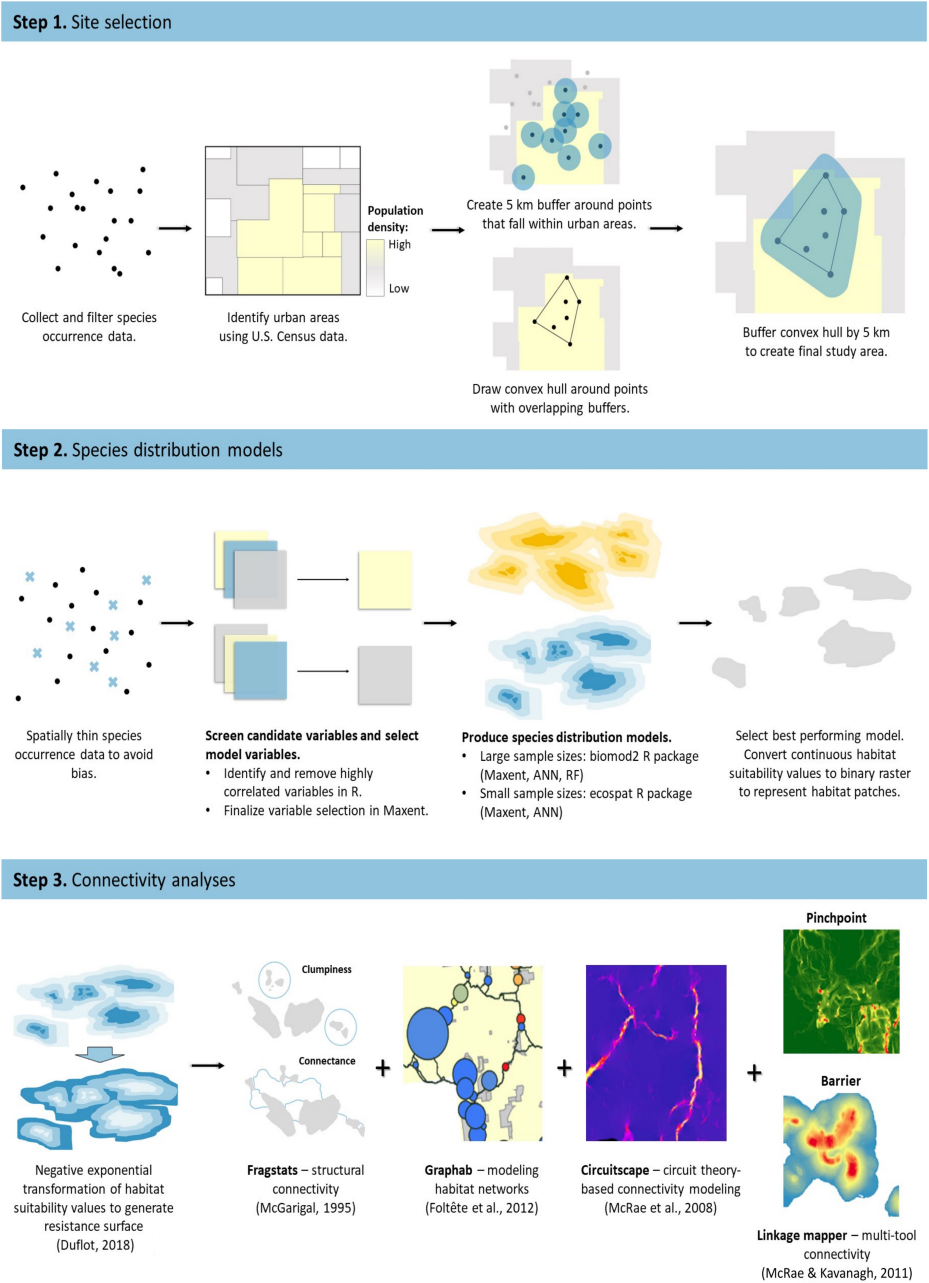
Conceptual diagram describing methods for urban site selection, species distribution models, and connectivity analyses.

We sought to represent connectivity networks that would be biologically relevant and reflect species' distributions within these urban regions and therefore opted not to estimate connectivity throughout the entire urban zone. Our primary criteria for selecting where to assess connectivity within each urban region were for it to be at an appropriate scale for understanding potential movement among habitat patches for this suite of species and where multiple focal species co‐occur. We started the selection process by creating 5 km buffers around each species occurrence within the four urban regions. We used this distance because it encompassed our dispersal estimates for nearly all the focal species (Table 1) and should therefore represent potentially accessible areas within the urban landscape. Our aim was to identify sections of each urban landscape with networks of habitat that support populations of multiple target species. We merged occurrences for all contiguous, overlapping 5 km buffers (<10 km separating each nearest neighbor occurrence). These merged occurrences formed the basis for our connectivity analysis representing areas where multiple rare species co‐occur in close proximity. We created a convex hull (smallest polygon connecting all points) around the merged locations using ArcMap and buffered this by 5 km to create the final analysis area where we generated the connectivity network for each species. The buffered convex hull concentrated the connectivity analysis on the urban landscape immediately surrounding the merged occurrences for multiple species where connectivity information would be most useful. In all cases, these buffers included areas both within and outside our defined urban study areas (Figure 2).

For every urban region aside from Detroit, this process resulted in a single analysis area. Detroit presented several issues that led us to slightly modify our species number threshold of three. The Detroit urban region was the most biologically diverse with occurrences from eight of the nine target species, several of which were found in no other urban site (e.g., American Bumble Bee, Eastern Foxsnake, Northern Long‐eared Bat). The contiguous Detroit urban region is much larger than the other sites and some species have a restricted distribution due to specialized habitat (e.g., Eastern Foxsnake in shoreline and river associated wetlands). Consequently, the habitats and species occurrences were too dispersed for overlapping 5 km buffers to encompass all eight species. Therefore, to maintain consistency in how we defined our analysis areas we lowered the species number threshold to two for creating analysis zones within the Detroit urban region. This permitted multi‐species connectivity analyses for the species only found within the Detroit urban region. There were four separate analysis areas in Detroit (Figure 1; South, Southwest, West, North) with overlapping 5 km buffers for at least two species. After subdividing Detroit our total number of urban analysis areas was seven, with 2–5 species per site (Table 2).

**TABLE 2:** Habitat and connectivity metrics for each species, at each focal urban site, including the percentage of the study area that we classified as habitat for each species, the percentage of that habitat that falls within the urban boundary, the percent of total habitat within protected areas, and within urban protected areas (in parentheses), and the clumpiness and connectance metrics from Fragstats.

Site	Species	Percent habitat	Percent habitat in urban	Percent protected habitat (in urban protected)	Clumpiness	Connectance
Benton Harbor	Blanding's Turtle	16	17.4	15.1 (0.2)	0.9	17.3
	Box Turtle	26	6.8	13.7 (0.02)	0.9	23.3
	Spotted Turtle	16.4	16.4	17.3 (0.4)	0.9	7.5
Kalamazoo	Blanding's Turtle	10.7	65.3	19.0 (15.3)	0.9	4.9
	Box Turtle	34.3	68.7	10.9 (8.0)	0.9	6.3
	Eastern Massasauga	2.6	75.5	21.2 (20.1)	0.8	4.4
	Henslow's Sparrow	3.5	28.5	5.3 (1.5)	0.9	3.0
	Spotted Turtle	12	66.9	17.1 (14.2)	0.9	2.1
Lansing	Black and Gold Bumble Bee	8.6	29.5	16.4 (5.3)	0.7	81.1
	Blanding's Turtle	46.3	46.1	18.6 (7.7)	0.9	7.8
	Eastern Massasauga	8.3	48.2	23.3 (10.9)	0.7	6.9
Detroit South	American Bumble Bee	1.6	61.2	13.8 (7.0)	0.6	72.1
	Eastern Foxsnake	11.3	63.4	35.0 (16.2)	0.7	3.0
Detroit Southwest	Black and Gold Bumble Bee	4	21.2	4.2 (1.0)	0.7	52.6
	Blanding's Turtle	11	41.7	14 (6.8)	0.9	7.2
	Henslow's Sparrow	5	34.5	3 (0.4)	0.9	3.8
	Northern Long‐eared Bat	14.3	46.2	13.8 (7.6)	0.8	20.8
Detroit West	Eastern Massasauga	7	35.3	25.6 (3.1)	0.7	3.7
	Spotted Turtle	35.3	45.2	21.1 (3.1)	0.9	1.7
Detroit North	Black and Gold Bumble Bee	4.8	45.6	19.8 (9.3)	0.7	41.7
	Blanding's Turtle	25.8	63.4	22.6 (12.3)	0.9	3.0
	Eastern Massasauga	4.6	67.4	30.4 (18.0)	0.7	2.5
	Spotted Turtle	25	64.4	22.5 (13.0)	0.9	1.1

The City of Kalamazoo created a Natural Features Protection (NFP) Ordinance and Development Standard to guide development of land on or near areas identified as Natural Features in the City to achieve long‐term protection of natural areas (Kalamazoo Zoning Standards; Natural Features Protection Overlap Standards, https://ecode360.com/34397622). The City's 2025 Master Plan has identified protection of natural features as a community priority during community planning efforts. The Master Plan defines Natural Features as “an area with existing natural features, including creeks, floodplains, stands of large trees, and slope that should be protected through such methods as conservation easements or land acquisition” (Kalamazoo Zoning Standards; Natural Features Protection Overlap Standards, https://ecode360.com/34397622). The ordinance adds additional development standards that require thoughtful design around Natural Features when properties are developed or redeveloped. The NFP Overlay district, a 2183 ha area that was delineated in the City of Kalamazoo to protect Natural Features, was adopted in 2019 and has been successfully implemented since then. We incorporated the NFP by comparing the newly protected areas under this program to the connectivity patterns from the Kalamazoo rare species included in our analysis.

### 2.1 Species distribution models

We developed a Michigan species distribution model (SDM) for each species that was projected across the state so habitat suitability values would be available for each urban region. Prior to developing any SDMs, we spatially thinned each species' occurrence data to reduce the potential for biased results from over‐represented sites (Figure 2). We used ArcMap to select the maximum number of occurrences for each species at a minimum spacing of 1 km and observed how many occurrences remained, then decreased the distance if this resulted in fewer than 20 occurrences (Appendix S1). The 1 km minimum distance prevented any individual population from being overrepresented in the dataset. In some cases, a shorter distance was necessary for species with limited distributions. We used 20 occurrences as a minimum target sample size for producing informative models. The ensemble of small models (ESM) approach applied here has been demonstrated to be effective with even smaller sample sizes (Breiner et al., 2018). Few American Bumble Bee occurrences were available, and we chose to use the Black and Gold Bumble Bee model as surrogate habitat for the closely related American Bumble Bee.

The Eastern Massasauga SDM was developed earlier than the models for the other eight target species as part of an effort to generate habitat suitability data for use by the USFWS in delineating sensitive habitat for this federally Threatened species. The primary differences between the Eastern Massasauga model and those for the other eight species relate to the predictor variables and modeling approach used. These differences are described in more detail below.

We screened a diverse set of candidate variables (30 m resolution) representing different habitat elements including land cover (2016 Coastal Change Analysis Program [C‐CAP]; https://coast.noaa.gov/digitalcoast/tools/lca.html), hydrology, and elevation for each individual SDM (Appendix S1). We derived some predictor variables from land cover classes using the ZonalMetrics toolbox in ArcMap (Adamczyk & Tiede, 2017). The variables for the Eastern Massasauga SDM were based on the same land cover dataset (2016 C‐CAP) but the derived variables did differ in the size of the focal window used for calculating percentage of a given land cover class (e.g., scrub shrub wetland percentage 300 m vs. 5 ha; Appendix S1).

The procedure for variable selection was consistent for both modeling efforts. First, we identified and removed highly correlated variables (>0.75) with the “corSelect” function in the fuzzySim R package (Barbosa, 2015), keeping the variable from each correlated pair that had a better performing model using the default AIC method. We then buffered the thinned occurrence data (10 or 25 km, see details below) to clip the remaining candidate variable rasters for final variable selection in Maxent (Phillips et al., 2006; Phillips & Dudík, 2008). We further evaluated the remaining variables using the jackknife of variable importance and training gain output in Maxent (Cobos et al., 2019), retaining variables that improved model performance based on these metrics. These variables were then used for all model types. The final variable sets for each species with contribution (ecospat) and importance (biomod2) values are available in the Appendix S1.

We used a 10 km buffer for background point selection (10,000 random points) for all species except Henslow's Sparrow and Northern Long‐Eared Bat, which had a 25 km buffer. The background point selection for presence only models should represent accessible area for the modeled species and, despite the low to moderate dispersal distances, we believe that the broader extent buffers are a better match to the scale of habitat selection for these volant species (Gómez‐Ruiz & Lacher, 2016; Monadjem et al., 2021; Virkkala et al., 2022; Zhang et al., 2018). These buffered regions served as the area for background point selection in all models.

We had sufficient Eastern Massasauga occurrences (*n* = 152) to generate SDMs with the R package biomod2 vers 3.3‐7.1 (Thuiller et al., 2009) using Maxent, artificial neural network (ANN), and random forest (RF) model types. The sample sizes for the remaining species were low enough (29–56) that we opted to use a different R package, ecospat (Breiner et al., 2015; Di Cola et al., 2017), that was developed for datasets with few occurrences. Ecospat uses an ESM approach where separate models are produced with each pair of variables before an ensemble is created under a weighting scheme. We used Maxent and ANN for the ecospat ESMs as RF does not perform well in this type of modeling application (Breiner et al., 2018). All biomod2 and ecospat models used 10 evaluation runs of 80% training partitions for model calibration with random splitting. For the Eastern Massasauga biomod2 models, we used the R package wallace (Kass et al., 2018) to determine the Maxent regularization (1–5; increments of 1) and feature sets (Linear [L], Linear + Quadratic [LQ], Linear + Quadratic + Hinge [LQH]) with AICc and internal default biomod2 tuning for the ANN models. All Maxent and ANN ESMs used ecospat internal default tuning. Internal selection for ensembles was used in biomod2 (AUC > 0.7) and ecospat (Somers *D* > 0). We used Area under the Curve (AUC) as the primary model selection metric for the biomod2 models and Boyce Index (Hirzel et al., 2006) implemented in ecospat using the “ecospat.boyce” function for the ESMs to determine what model was used for the connectivity analysis (Appendix S1). Finally, we converted the continuous habitat suitability values from each species SDM to a binary raster of habitat and non‐habitat to represent the distribution of habitat patches. We used the maximum sum of sensitivity and specificity (MSSS) threshold for all ecospat ESM models (equivalent to the maximum true skill statistic [TSS]; Liu et al., 2013). The top Eastern Massasauga biomod2 model was the RF model and the MSSS threshold was too restrictive (i.e., virtually no habitat remained) so a 10th percentile training threshold was used.

### 2.2 Connectivity

There is no single metric or technique that can satisfy all the different criteria for assessing landscape connectivity (Kindlmann & Burel, 2008). Therefore, we applied several methods to target different aspects of connectivity that together offer a more holistic representation of how connected these urban landscapes are for our selected species (Figure 2). Prior to the connectivity analysis we quantified several elements of landscape composition for each urban site's 5 km buffered convex hull: total area (ha), percent urban, percent protected areas overall and within the urban part. Then we calculated percent habitat (based on thresholded SDMs) within the study area, and percent of overall habitat in urban areas, protected areas overall, and in urban protected. For Kalamazoo, we also calculated percent habitat in the NFP. These composition metrics provide useful information about the landscape surrounding our occurrence data.

Our connectivity analysis incorporated four approaches including Fragstats (McGarigal, 1995), Graphab (Foltête et al., 2012), Linkage Mapper (McRae & Kavanagh, 2011), and Circuitscape (Shah & McRae, 2008). Fragstats quantifies landscape composition and metrics related to structural connectivity (landscape configuration; LaPoint et al., 2015) to assess levels of connectivity and fragmentation using habitat patch data. The other approaches go further, representing functional connectivity (organism perspective; LaPoint et al., 2015) as movement potential across the landscape among the structural habitat elements (e.g., patches). Central to this concept is determining an underlying cost or resistance surface to represent how an organism traverses a heterogeneous landscape. One option is to use Euclidean distance, however this is unlikely to adequately portray movement across non‐uniform landscapes (Trainor et al., 2013). Therefore, assigning values to landscape features that match the propensity or difficulty for an organism to pass through them is a preferable alternative to distance alone. Resistance surface development has often used expert opinion where taxon experts assign values to landscape features, but this approach has obvious drawbacks including inaccurate assumptions of organism behavior (Zeller et al., 2012) and limited transferability to different landscapes. Ideally, data from radio telemetry (Trainor et al., 2013) or genetics are available that can be used to derive a resistance surface that reflects real world movement or gene flow (Beninde et al., 2016). When these data are not available, habitat suitability data from SDMs can serve as an underlying resistance surface (Duflot et al., 2018). However, simply inverting the habitat suitability values and assigning low resistance to highly suitable areas has been shown to be ineffective, prompting evaluation of non‐linear responses for transforming habitat suitability to landscape resistance (Keeley et al., 2016). We applied the negative exponential transformation function used by Duflot et al. (2018) to our continuous SDM habitat suitability output. Under this transformation, a resistance surface will assume a resistance value of 1000 when habitat suitability = 0, and 1 when habitat suitability equals or exceeds the suitable habitat threshold (i.e., MSSS for ESMs and 10th percentile for Eastern Massasauga biomod2 model). This approach relies on the assumption that movement is at least partially dependent on habitat suitability. Several studies have shown habitat suitability is not always associated with gene flow (Peterman et al., 2014; Ziółkowska et al., 2016) so alternative movement paths should be considered, however in an urban environment with fewer natural landscape features available, movement is more likely to be focused in greenspace corridors.

Roads were not explicitly included in the SDMs as many occurrences are located near roads, potentially resulting in a false signal, and were therefore not represented in the final resistance surfaces. However, their ubiquity, particularly in these urban landscapes, and well documented chronic negative impacts on populations (Beebee, 2013; Roger et al., 2011; Taylor & Goldingay, 2010) justifies their inclusion in our connectivity analysis. Therefore, we buffered a road line shapefile (U.S. Census Bureau, Geography Division) by 30 m and converted this to a raster, assigning taxon relevant values before mosaicking with each species SDM‐based resistance surface. We used 1000 as the road cost value for the five reptile resistance surfaces based on well‐documented negative road effects on turtles and snakes (Garrah et al., 2015; Paterson et al., 2019; Robson & Blouin‐Demers, 2013). Few studies have examined road impacts for Henslow's Sparrow and Northern Long‐eared Bat specifically, but data from closely related species suggest strong behavioral avoidance of roads but unclear mortality risk (Benítez‐López et al., 2010; Bennett & Zurcher, 2013; Bhardwaj et al., 2021; Cooke et al., 2020). As a result, we assigned a road cost value of 500 for these two species. Finally, research has shown that roadsides can provide suitable habitat for several bee species (Bhattacharya et al., 2003); however, their propensity to cross roads is not well known and bees may follow linear paths tracking habitat instead of moving to the other side (Brebner et al., 2021). Given the potential for habitat creation along roadside margins but clear mortality risk associated with this habitat, we assigned a road cost value of 250 for the bee resistance surface being applied to both species.

### 2.3 Graphab

Graphab uses a graph network approach with least cost paths (LCPs) to assign various connectivity metrics to links and nodes for the landscape of interest. The nodes can be defined as habitat patches from a habitat suitability map. The patches included in the habitat network should be sufficiently large to be viable for individuals to persist within them (Duflot et al., 2018). Therefore, we incorporated any available published data for our study species to base these decisions on (Table 1). In several cases, there were no clear recommendations for minimum viable habitat size due to widely different estimates or a dearth of available data. For these species (Black and Gold/American Bumble Bees; Eastern Massasauga; Eastern Foxsnake) we calculated the sizes of all habitat patches overlapping with SDM occurrence data, then dropped the smallest 20% and compared that patch size to available data to determine if it was ecologically reasonable. This was based on the concept of the 10th percentile threshold as applied to SDMs where areas with suitability values associated with the lowest 10% of occurrence records are removed from consideration as habitat. The assumption is that these records are more likely to be from marginal habitats and therefore not fully representative of higher quality habitat necessary for population persistence. In this case, we used a more conservative cutoff but with the same assumption, that the patch sizes associated with the smallest 20% of occurrence records are more likely to be from marginal habitat fragments.

Duflot et al. (2018) discuss the merits of the Probability of Connectivity (PC) for prioritizing landscape features and the ranking capabilities using the percentage of variation in PC (*d*PC_*k*_; Saura & Pascual‐Hortal, 2007). The *d*PC_*k*_ connector component can show the nodes or linkages essential for maintaining overall connectivity. Calculating this metric requires the use of median dispersal distances. We used reported dispersal distances, which are our best estimates of the distances adult individuals are capable of moving between populations, for all our selected species or closely related surrogates if none existed (Table 1). We also permitted all possible LCPs to be formed regardless of distance for a complete visualization of patch connections in our analysis area.

We used the LCPs from Graphab to create multi‐species connectivity linkages for each site. We converted each LCP to a raster with the same cell size as the SDM variables (30 m) then used Cell Statistics in ArcMap to sum all the rasters per site.

### 2.4 Circuitscape

Circuitscape uses circuit theory to display areas of high and low predicted connectivity in a landscape based on an underlying resistance surface. It can be run between nodes representing habitat patches or populations to compare connectivity levels. In our case, we were more interested in visualizing potential connectivity across the landscape so we used an approach where peripheral nodes are placed outside the study area and current is run between them to avoid the spikes in current that occur at nodes from biasing the interpretation of connectivity patterns in our analysis area (Koen et al., 2012). An advantage of Circuitscape over the two LCP‐based approaches is that it does not result in a single representation of a pathway between two patches, as numerous routes may exist that are all used by the organism of interest.

### 2.5 Linkage Mapper, pinch point, and barrier

Linkage Mapper also maps linkages among habitat patches using LCPs and has two connectivity‐based tools for assessing movement capacity through a landscape. The Barrier Mapper (McRae, 2012a) and Pinchpoint Mapper (McRae, 2012b) tools help reveal aspects of landscape connectivity missing from the other tools. Both of these tools were run after the Linkage Pathways tool in Linkage Mapper. We used the same resistance surfaces with Linkage Mapper with a default corridor width setting and dispersal distances to limit what patches are considered for LCPs. Eastern Massasauga and Eastern Box Turtle have been reported to have a maximum dispersal distance of 2 km so this was used rather than the median distance of 1 km in Graphab where a median distance was called for. Barrier Mapper identifies locations where mitigating a high cost area by creating a corridor or eliminating a barrier could dramatically improve connectivity. The pinch points from the Pinchpoint Mapper are based on running Circuitscape current along LCP corridors and show locations where the current is highly concentrated in narrow corridors. These are therefore potentially vulnerable linkages, as few alternative pathways exist.

### 2.6 Fragstats

We calculated two metrics (clumpiness and connectance) using Fragstats (vers. 4.2; McGarigal et al., 2012) to quantify aspects of the arrangement of habitat patches for species/site combinations. Clumpiness estimates how aggregated patches are on the landscape. Connectance estimates how connected the landscape is based on a species‐specific maximum distance threshold for patches to be considered reachable. An important consideration when comparing landscape metrics is understanding their sensitivity to overall habitat abundance (Wang et al., 2014). Clumpiness is not sensitive to habitat abundance. Connectance is not sensitive to abundance when habitat makes up less than 30% of the landscape, which is true for most of our sites (Wang et al., 2014).

## 3 RESULTS

All species' SDMs had high performance metrics (mean AUC = 0.94 ± 0.04, range: 0.86–1.0; Appendix S1). All figures produced from the connectivity analyses are provided in the Supplementary Figures section. They are organized by site (Benton Harbor: Figures S1–S12, Kalamazoo: Figures S13–S32, Lansing: Figures S33–S44, Detroit South: Figures S45–S52, Detroit Southwest: Figures S53–S68, Detroit West: Figures S69–S76, Detroit North: Figures S77–S92) then grouped by species. Multi‐species connectivity figures for each site are presented at the end (Figures S93–S98).

### 3.1 Kalamazoo

**FIGURE 3:**
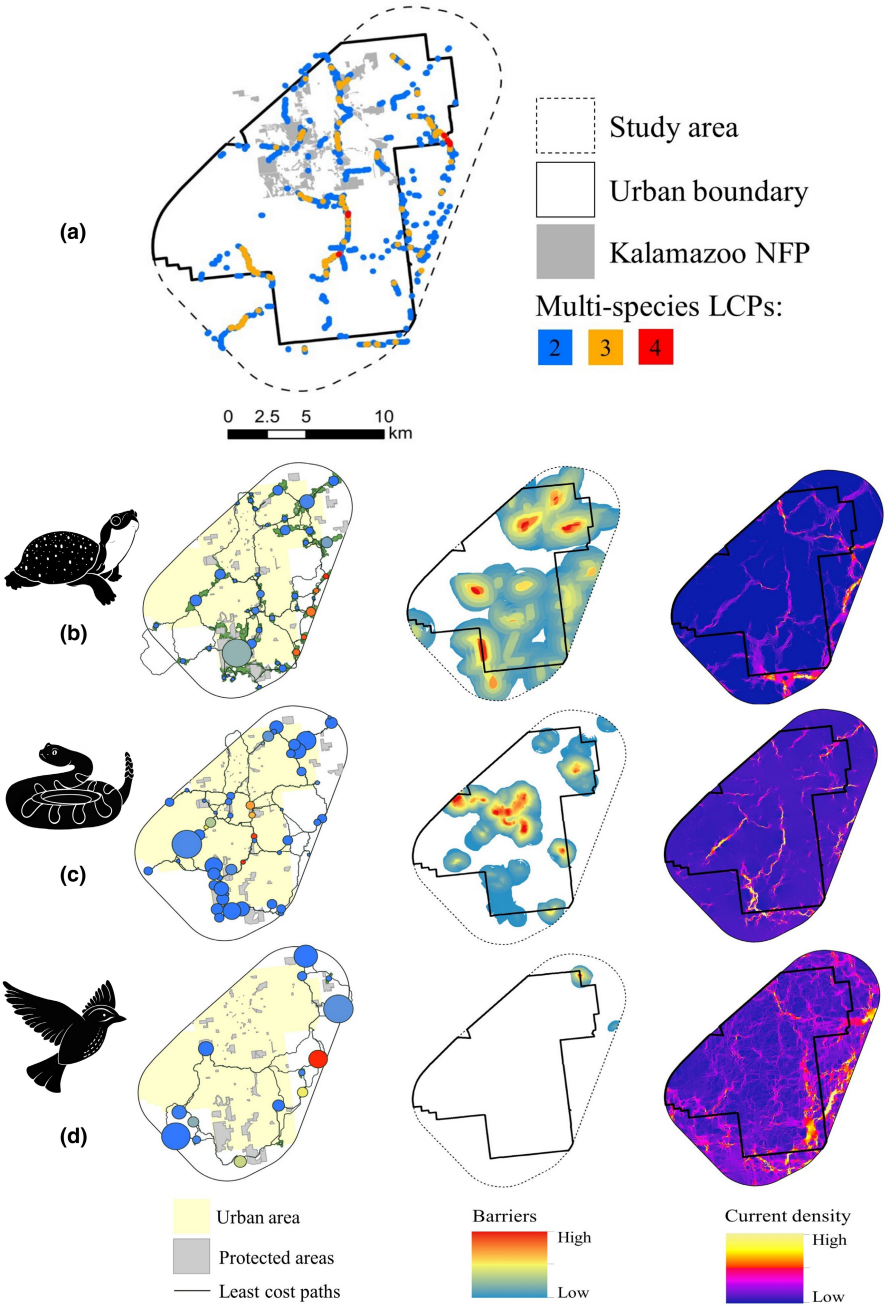
Map of the Kalamazoo study area, urban boundary, and Natural Features Protection initiative land holdings (a) showing the multi‐species least cost paths (LCPs) for two, three, and four species respectively. Individual species connectivity results from Kalamazoo for Blanding's Turtle (b), Eastern Massasauga (c), and Henslow's Sparrow (d) show (from left to right) the Graphab habitat network, barriers impeding connectivity, and Circuitscape current density. The Graphab maps represent patch size as the size of the circle and importance to overall connectivity based on color with warmer colors (orange and red) indicating higher importance.

Kalamazoo has the lowest amount of overall protected habitat (5.9%), but a recent NFP initiative will increase this figure (Table 3). This site has high connectivity potential with several multi‐species corridors running through the urban portion of the study area (Figure 3a). Additionally, despite only 4.4% of protected areas existing within the urban boundary (Table 3), the percentage of species habitat in urban protected areas is the highest at this site for Blanding's Turtle (Figure 3b; 15.3%), Eastern Massasauga (Figure 3c; 20.1%), and Spotted Turtle (14.2%) (Table 2). The percentage of habitat that falls within the Kalamazoo NFP is 4.8% for Blanding's Turtle, 6% for Eastern Box Turtle, 11.7% for Eastern Massasauga, 3.3% for Henslow's Sparrow (Figure 3d), and 8.6% for Spotted Turtle.

Significant barriers exist within the urban space for Blanding's Turtle, Eastern Box Turtle, Eastern Massasauga, and Spotted Turtle (Figures S14, S18, S22 and S30). Numerous pinch points are also present for each of these species (Figures S15, S19, S23 and S31). Henslow's Sparrows may be infrequent visitors with habitat largely located outside the urban area.

Eastern Box Turtle has the most habitat by far at 34.3% (23.5% in urban), and numerous existing connectivity pathways. Blanding's (10.7%) and Spotted (12%) Turtles, by comparison, are much more limited habitat‐wise within the Kalamazoo study area. Connectivity is also much more limited for Blanding's Turtles based on the Circuitscape results (Figure 3b).

Across the Kalamazoo study area, the Graphab results for Blanding's Turtle (Figure 3b), Eastern Massasauga (Figure 3c), and Spotted Turtle (Figure S29) all similarly illustrate the importance of small patches for overall habitat connectivity. The Graphab results also show that the highest priority patches for both Blanding's and Spotted Turtle connectivity occur outside the urban space (Figure 3b; Figure S29).There are movement corridors within the urban space for Eastern Massasauga, boosting connectivity potential for this species but these conservation opportunities also carry potential risks from a human conflict standpoint (Figure 3c). Circuitscape results for both Eastern Massasauga and Spotted Turtle show similar patterns of high current areas within the urban space (Figure 3c; Figure S32). These species co‐occur in many Michigan habitats. Most of the species have highly aggregated habitat, as illustrated by their clumpiness values (ranging from 0.88 to 0.94), aside from Eastern Massasaugas whose habitat is slightly more dispersed (0.76). Clumpiness values were similar across all sites for these five species.

### 3.2 Benton Harbor

The Benton Harbor study area's urban space has the lowest potential for wildlife movement of any examined site (Figures S4, S8 and S12). However, the non‐urban space is highly connected for each of the three turtle species (Blanding's, Eastern Box, and Spotted) found here. Benton Harbor is the smallest site by area (8678.0 ha), percentage of urban space (25.1%), and percentage of protected urban space (0.7%) within the analysis area (Table 3). Small sections of suitable habitat exist at the urban margin for each turtle species and most habitat is outside the urban boundary (Table 2).

The connectivity measures show that movement potential is limited in the urban space with few habitat patches separated by long paths of least resistance (LCPs; Figures S1, S5 and S9) and barriers present for each species within the urban space (Figures S2, S6 and S10). The multi‐species LCP based connectivity map suggests the shared connectivity is restricted to links crossing the northern section of the urban space (Figure S93). However, the Circuitscape results show that each turtle species has high movement potential along the riparian areas that border the urban boundary (Figures S4, S8 and S12). The similar patterns in Circuitscape current flow for each species indicate that these are critical linkages from a multi‐species connectivity perspective. These linkages also appear to be broad as the pinch point analysis shows few narrow corridors outside the urban areas (Figures S3, S7 and S11). The Fragstats results also suggest the habitat bordering the urban boundary and outside the urban space is highly connected as the connectance values for each species are the highest compared to any other site where they occur (Table 2).

### 3.3 Lansing

The Lansing study area is the second smallest site by area (20,321.5 ha; Table 3) with about half the site within the urban boundary (51.7%). The Lansing study area has a high percentage of overall habitat, and habitat within the urban area for Blanding's Turtle (46.3% overall; 21.4% urban) and Eastern Massasauga (8.3% overall; 4.0% urban) relative to the other sites. Although, the percentage of habitat in urban protected areas is about half of what it is in Kalamazoo, for both species. For Eastern Massasauga, a North–South barrier (Figure S42) separates the existing urban habitat patches (Figure S41). Likewise, for Black and Gold Bumble Bee, many barriers exist in the northeastern section of the study area, especially within the northern urban section. There are a significant number of pinch points for both Black and Gold Bumble Bee and Eastern Massasauga indicating that movement could be highly constrained over a broad area (Figures S35 and S43). Conversely, the considerable area of Blanding's Turtle habitat provides many avenues for dispersal (Figures S37 and S40). The Fragstats results for Black and Gold Bumble Bee show similar clumpiness across Lansing and the other two sites (Detroit North and Southwest) but a higher amount of habitat connectance in Lansing (Table 2).

### 3.4 Detroit South

Detroit South is the second largest study area (39,898.7 ha) with a considerable urban portion (64.5%). This is the only site that harbors Eastern Foxsnake, with 35% of this snake's predicted habitat falling within protected areas. This represents the highest percentage of predicted habitat within protected areas of any of our study species. Eastern Foxsnake connectivity is largely restricted to the coastline and several riparian areas (Figures S49 and S52). American Bumble Bee has high habitat connectance despite more dispersed habitat patches relative to other species (clumpiness = 0.58), likely resulting from their relatively long (10 km) dispersal capability. However, American Bumble Bee habitat availability is low and thus dispersal opportunities are limited in this urban landscape (Figure S48). Significant dispersal barriers are spread throughout the central part of the study area and the existing corridors are narrow (Figures S46 and S47).

### 3.5 Detroit Southwest

The Detroit Southwest site has a unique assemblage of species with one representative from all taxonomic groups in the analysis and the only site with Northern Long‐eared Bat occurrences (Table 2). Habitat for all species largely exists in the periphery of the study area, outside the urban center. Blanding's Turtle and Northern Long‐eared Bat have many barriers and pinch points around the periphery indicating that there may be limited potential for movement across this site. As with other sites, Henslow's Sparrow has few linkages outside the periphery of the study area. Detroit Southwest has the lowest percentage of habitat in protected areas for Black and Gold Bumble Bees (4.2%), Blanding's Turtles (14%), and Henslow's Sparrows (3%) of any site in which they occur.

### 3.6 Detroit West

The Detroit West site has the highest percentage of protected area (15.9%) of any site but the second lowest percentage (2.9%) of protected area within the urban boundary (Table 3). The protected areas are largely immediately adjacent to the urban boundary and are important from a connectivity perspective based on Graphab results (Figures S69 and S73) and percentage of habitat in protected areas for both Eastern Massasauga (25.6%) and Spotted Turtle (21.1%). Interestingly, there is very little direct overlap in the multi‐species linkages for these two species, despite sharing similar habitats in many areas (Figure S97). Eastern Massasauga habitat is mostly located outside the urban area (Figure S69) and with a considerable number of barriers inside the urban area, movement will likely be highly restricted.

**TABLE 3:** Summary of focal study sites including the total study site area (ha), the percent of site area that falls within the urban boundary, the percent area that is protected, and the percent of protected areas that fall within the urban boundary (parentheses).

Site	Total area (ha)	% urban	% protected areas (in urban)
Benton Harbor	8678.00	25.1	7.7 (0.7)
Kalamazoo	39,103.26	66	5.9 (4.4)
Lansing	20,321.46	51.7	13.0 (5.6)
Detroit S	39,898.65	64.5	11.8 (5.5)
Detroit SW	29,777.77	59.7	8.6 (5.2)
Detroit W	37,767.95	52	15.9 (2.9)
Detroit N	61,619.58	72.7	13.7 (8.4)

### 3.7 Detroit North

The Detroit North site is the largest site (61,619.6 ha), with the highest percentage of urban area (72.7%), and the highest percentage of urban protected area (8.4%) of any site. Non‐urban protected areas are important for providing habitat at this site with the highest percentage of habitat in protected areas for all four species (Black and Gold Bumble Bee = 19.8%, Blanding's Turtle = 22.6%, Eastern Massasauga = 30.4%, Spotted Turtle = 22.5%) occurring here. Multi‐species linkages extend throughout the study area (Figure S98).

## 4 DISCUSSION

Integrating multiple connectivity tools into a single analysis provides a range of useful information that can be incorporated into local conservation planning. We observed distinct differences in rare species connectivity patterns across these urban environments reflecting the unique nature of these landscapes and their associated species. A promising finding was moderate to high percentages (~40%–75%) of habitat within the urban analysis areas for many of the assessed species; however, major connectivity corridors tended to fall outside of the urban boundaries. Thus, the primary challenge for promoting rare species persistence in these landscapes will be maintaining habitat corridors not only within the urban areas but from the surrounding landscapes as well (Morrison & Boyce, 2009).

Riparian habitats have been recognized as critical components of well‐connected landscapes (Fremier et al., 2015). When left intact, these dendritic networks of largely linear habitat features tend to preserve linkages between lowlands and headwaters, but also across the aquatic and terrestrial boundary (Beier, 2012). Furthermore, road underpasses associated with aquatic habitat features may enable some species to avoid risky road crossings (Glista et al., 2009). Our Circuitscape and pinchpoint results support the importance of riparian habitats for connectivity of wildlife (especially aquatic species) in highly disturbed urban landscapes as well, as these may be the only remaining linkages between fragmented greenspaces. Protecting these riparian corridors therefore becomes critical for connectivity of urban wildlife.

### 4.1 Species‐specific habitat and connectivity patterns

The three turtle species consistently had among the highest amount of habitat as percentage of the landscape across study sites. The urban landscapes with populations of these rare turtle species are therefore retaining wetlands (Blanding's and Spotted Turtles) and forest/edge (Eastern Box Turtle) habitats. Collectively, turtles are one of the most at‐risk vertebrate groups (Lovich et al., 2018) making this a promising finding. With abundant habitat, conservation planning can therefore focus on existing population‐level threats. The mortality risk posed by roads for turtles, particularly nesting females, is well documented (Gibbs & Shriver, 2002; Howell & Seigel, 2019; Steen & Gibbs, 2004) making road mitigation along LCPs a priority conservation action for these species. Importantly, our analysis did not evaluate suitable nesting areas or any demographic data, therefore extant urban populations could have skewed sex ratios and age classes (i.e., older male turtles) with little recruitment.

On a ‐site‐by‐site basis, the Eastern Massasauga and Eastern Foxsnake had a comparable percentage of habitat in urban areas to the two semi‐aquatic turtles in the analysis (Blanding's and Spotted Turtles) whereas the bee species and Henslow's Sparrow were consistently lower than other species (Table 2). This is likely due to Eastern Massasauga preference for wetland‐associated grassland habitat and wetland retention in the urban areas in which this species persists. Similarly, a majority of the remaining marsh/emergent wetland habitat used by Eastern Foxsnakes at the Detroit South site is within the urban boundary. Habitat amount for Black and Gold and American Bumble Bees, Eastern Massasauga, and Henslow's Sparrow were collectively the lowest as percentage of landscape. Grassland and prairie habitats represent some of the rarest ecosystems in North American landscapes (Samson & Knopf, 1994), and managing fragments left in urban environments (via, for example, prescribed fire) is challenging (McLaughlin et al., 2014). There were differences in percentage of habitat in urban areas for these grassland and prairie associated species.

The *d*PC_*k*_ connector metric from Graphab enabled cross‐site comparisons of patch importance to observe trends in overall habitat network connectivity. Importantly, this analysis showed that larger habitat patches (patch size represented by the size of the circle in Graphab figures) are often not the most important from a network connectivity perspective. This was observed across taxa in landscapes with more numerous habitat patches over a broader extent (Kalamazoo‐Blanding's Turtle, Eastern Massasauga; Detroit North‐Black and Gold Bumble Bee). Larger habitat patches were the most important for maintaining connectivity in landscapes where patches were spatially restricted and a disproportionate amount of habitat was associated with a single large patch (Benton Harbor‐all three turtle species; Detroit South‐Eastern Foxsnake; Detroit Southwest‐Black and Gold Bumble Bee). In these situations, the high priority habitat patch was located outside the urban boundary.

The general trend in multi‐species connectivity from a shared LCP perspective was fewer and shorter linkages at sites with fewer species, as would be expected (Figures S93–S98). However, there were differences between sites with the same number of species such as Benton Harbor and Lansing (three species) and Detroit Southwest and Detroit North (four species). In both cases, the site with more similar species, Benton Harbor (the three turtle species) and Detroit North (two turtles and Eastern Massasauga), had substantially more shared LCP linkages (Figures S93 and S98). Detroit North and Kalamazoo had extensive multi‐species linkages relative to the other sites (Figure 3a; Figure S98). The similar patterns likely reflect the shared life histories of some species (e.g., the aquatic turtles) and demonstrate that multiple species are relying on the same remnant urban patches of greenspace. Collectively, the reptile species in Detroit North and Kalamazoo have a higher percentage of habitat within the urban boundaries compared to other sites while Black and Gold Bumble Bee and Henslow's Sparrow were outliers at each site. This is largely due to these two sites having the highest percentage of urban area (Table 3) but also importantly shows how urban areas can retain habitat for rare reptiles.

The Circuitscape results enabled site‐wide assessments of all potential movement pathways, an important advantage over LCP based analyses (McRae et al., 2008). At several sites, predicted movement corridors indicated by high current density in the Circuitscape maps overlap among species despite not showing up in our multi‐species LCPs. In these situations, broader corridors may have less cell‐to‐cell overlap when combining LCPs but are still multi‐species linkages and represent a weakness of combining LCPs at a finer cell resolution like we do here (30 m). For example, even though the multi‐species LCPs for Detroit North show abundant shared LCPs (Figure S98), there are numerous areas of high connectivity shown in the Circuitscape maps for each species (See Figures S80, S84, S88 and S92). Conservation planning for this site could incorporate both tools. The multi‐species LCPs show priority corridors for conservation as shared optimal pathways for several taxa, while the Circuitscape maps enable greater flexibility (i.e., multiple potential corridors) for identifying protected areas for conservation planning.

### 4.2 Protected areas

Protected areas do appear to play an important role in providing habitat for rare species relative to their landscape footprint. For example, over one‐third (35%) of Eastern Foxsnake habitat from the Detroit South site was found in protected areas that make up 11.8% of that assessed area. Although this was not universal, as observed with some species (Henslow's Sparrow) and sites (Benton Harbor and Detroit Southwest) (Tables 2 and 3). The importance of protected areas was particularly evident for the species more associated with less modified natural areas such as Eastern Massasauga and Spotted Turtle. These were the two species found at the Detroit West site, which has the most protected habitat but second lowest percentage of urban protected (Table 3). The lack of urban protected areas likely contributes to the low percentage of habitat within the urban boundary (Table 2) for both Eastern Massasauga and Spotted Turtle relative to other sites (aside from Benton Harbor for Spotted Turtle).

In addition to the amount of habitat they protect, we can assess the importance of protected areas by observing their relationship to priority habitat patches for overall connectivity from the Graphab analysis. High priority patches in protected areas were observed for several taxa at Benton Harbor (see Figures S1, S5 and S9), Detroit West (see Figures S69 and S73), and Detroit North (see Figures S77, S81, S85 and S89), illustrating that the types of habitats within protected areas and their position within the broader landscape influence their relevance to rare species connectivity.

Finally, information from urban areas with high rare species diversity but lower amounts of protected habitat can be provided to land conservancy groups to guide land acquisition and restoration efforts if high priority habitats remain. For example, the Detroit Southwest site had high taxonomic diversity for our evaluated species, and lower percentages of habitat in protected areas relative to other sites. While protecting habitat from development is the first step, protected habitat in urban landscapes can become degraded without continuous management to restore and maintain critical habitat structure (Le Roux et al., 2014). Management and maintenance of high‐quality habitat in these urban protected areas may be particularly challenging if processes (e.g., prescribed burning, canopy senescence and wood decay) pose hazards to humans (Le Roux et al., 2014). Conversely, restoration of urban protected areas brings the added benefit of a community‐based opportunity to connect people and nature (Standish et al., 2013).

### 4.3 Applied case study with Kalamazoo

**FIGURE 4:**
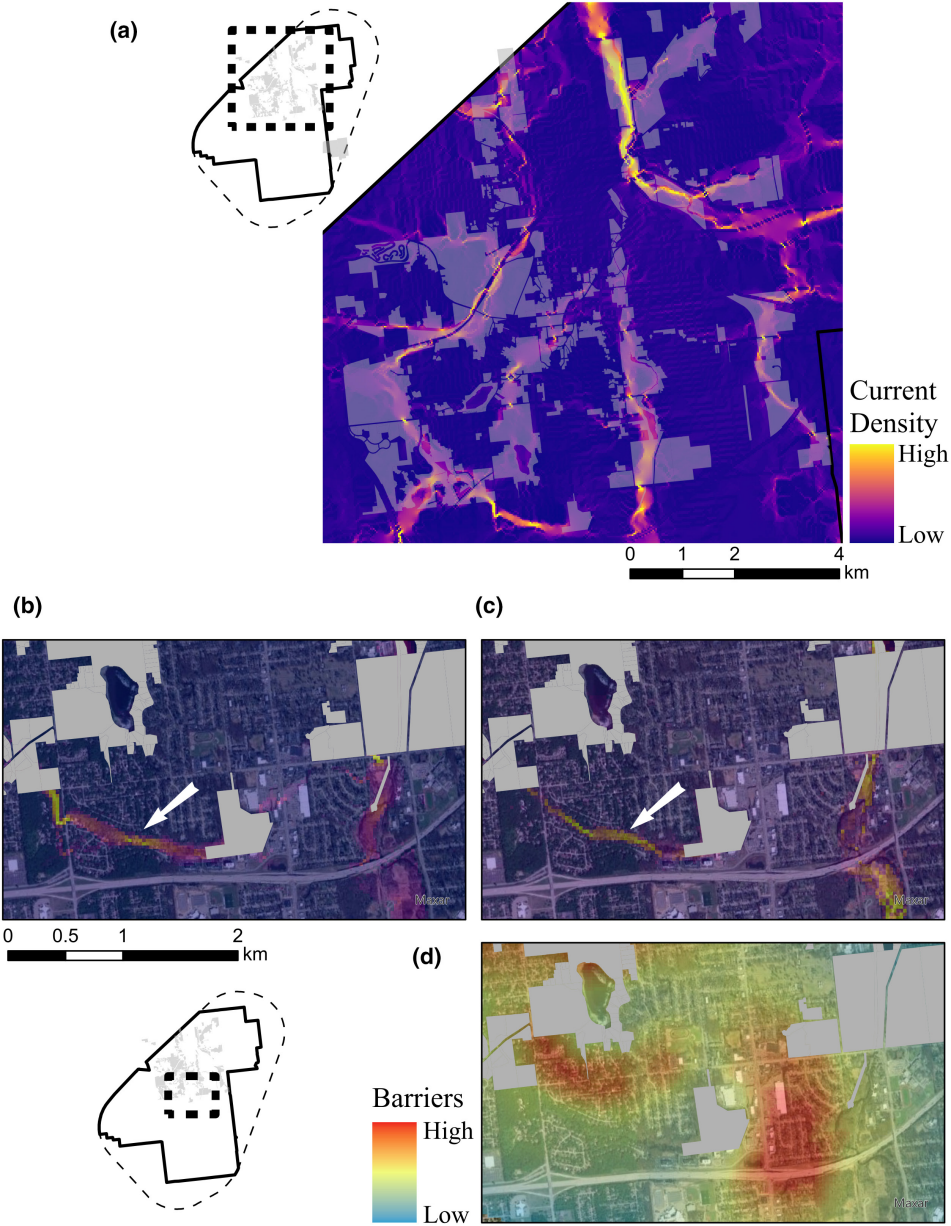
Map of the Kalamazoo Natural Features Protection (NFP) land holdings within our Kalamazoo study area with Spotted Turtle current density displayed underneath (a) showing overlap between protected areas and predicted movement corridors for this species. The southern edge of the NFP has a disjunct parcel adjacent to two riparian corridors (East–West and North–South). This parcel is shown with current density maps for Blanding's Turtle (b) and Eastern Massasauga (c), a white arrow in both maps identifies an unprotected stretch of the East–West riparian area that has high movement potential for both species. The parcel is also shown in relation to a barrier map for Eastern Massasauga (d). The barrier in red indicates that reducing the cost associated with the area between the parcel and the North–South riparian corridor would dramatically improve connectivity between habitat patches. This is a residential area; however, the unprotected East–West corridor represents more realistic and functional option for connecting the disjunct parcel to the broader protected network.

The Kalamazoo NFP demonstrates how greenspace initiatives will benefit rare species in urban environments (Figure 4a). Several multi‐species corridors are contained within the protected areas (Figure 3a). Identifying and protecting any greenspaces within urban areas will have a multitude of benefits including rare species habitat protection as protected habitats do not need to be large to boost connectivity (Wintle et al., 2019). The primary added benefit of species‐focused connectivity mapping is revealing the linkages that are most likely to be used by rare species thereby providing information that can guide future conservation planning. For example, the southern edge of the NFP area has several protected parcels with gaps of no protected status on either side of one disjunct parcel and riparian corridors running East–West and North–South (Figure 4a–d). Current density maps for Blanding's Turtle and Eastern Massasauga show that the gap on the left side contains a suitable movement corridor for both species along the East–West riparian area (Figure 4b,c). The Eastern Massasauga barrier to the right of the disjunct parcel indicates that the overlapping residential area represents a high‐cost area separating areas of suitable habitat (East–West and North–South riparian corridors, Figure 4c). This information helps identify where to prioritize future conservation efforts (e.g., East–West riparian area) and reveals the limits to enhancing connectivity when barrier removal is not possible.

### 4.4 Future directions

Our connectivity assessments were focused on where rare species occur within urban environments and the surrounding landscape, thus our analyses are not intended to appraise habitat and connectivity patterns city‐wide. These approaches could be applied to any of the selected urban areas from a city perspective rather than the taxon‐specific one used here, assigning generalized resistance values to land cover types to model connectivity of greenspaces. More species‐focused assessments that target population‐level data (e.g., demography; genetics) in urban environments will be needed as pervasive immovable barriers in urban spaces will invariably limit movement from surrounding landscapes.

Our approach for estimating connectivity relied on several assumptions, starting with the ESM SDMs that were based on few occurrences due to the rarity of the selected species. We obtained good model metrics for our ESMs but better models would be produced with a larger dataset representing a broader range of habitat conditions. Future research could investigate how models based on different sample sizes or geographic extents reflect occupied habitat in urban areas. We also assumed that animal movement would be related to habitat suitability. This has not been investigated for any of our focal taxa and it would be useful to establish this link when attempting to model connectivity in other landscapes. Finally, we did not incorporate any behavioral component into our methods but animals are unlikely to react similarly to all urban features (e.g., different types of roads); therefore, more detailed investigations of how to assign taxon‐specific resistance values for urban connectivity modeling would improve the accuracy of resulting connectivity networks.

Assessing multi‐species habitat and connectivity in urban landscapes, using these techniques, is increasingly important considering the threat of climate change and the high extinction debts these relatively recently fragmented landscapes may soon pay (Soga & Koike, 2013; Tilman et al., 1994). Identifying and protecting critical habitat patches and linkages will promote biodiversity in urban landscapes by providing refuge from heat islands and ensuring the potential for species to move under future climate scenarios. Urban planners and conservation managers may therefore improve the likelihood of persistence of rare species in these landscapes, while meeting the socio‐ecological goal of connecting people and nature.

## AUTHOR CONTRIBUTIONS

**Eric M. McCluskey:** Conceptualization (lead); formal analysis (lead); methodology (lead); writing – original draft (lead); writing – review and editing (lead). **Faith C. Kuzma:** Methodology (equal); visualization (lead). **Helen D. Enander:** Conceptualization (supporting); data curation (lead); methodology (equal). **Ashley Cole‐Wick:** Conceptualization (supporting); writing – original draft (supporting); writing – review and editing (equal). **Michela Coury:** Methodology (supporting); visualization (supporting). **David L. Cuthrell:** Conceptualization (supporting); data curation (supporting); writing – review and editing (supporting). **Caley Johnson:** Data curation (supporting); methodology (supporting); visualization (supporting). **Marianne Kelso:** Methodology (supporting); visualization (supporting); writing – review and editing (supporting). **Yu Man Lee:** Conceptualization (supporting); data curation (equal). **Diana Methner:** Methodology (supporting); visualization (supporting); writing – review and editing (supporting). **Logan Rowe:** Conceptualization (supporting); data curation (equal); writing – review and editing (supporting). **Alyssa Swinehart:** Methodology (supporting); visualization (supporting); writing – review and editing (supporting). **Jennifer A. Moore:** Conceptualization (equal); visualization (equal); writing – original draft (equal); writing – review and editing (lead).

## CONFLICT OF INTEREST STATEMENT

The authors declare no conflicts of interest.

## Supporting information


Figures S1–S98

Appendix S1

## Data Availability

We provide additional information and data pertaining to our SDMs and connectivity analyses in the Appendix S1 and Figures S1–S98. We are unable to provide georeferenced occurrence data used for most of the analyses in this manuscript due to the threats both illegal collection and indiscriminate killing pose to several of the rare species included in this study. The Michigan Department of Natural Resources and U.S. Fish & Wildlife Service have both expressed concern over the release of any sensitive species occurrence data.
