# Supplementary material for: Assessing habitat connectivity of rare species to inform urban conservation planning
Source: Ecol Evol. 2024 Mar 4;14(3):e11105. doi: 10.1002/ece3.11105 (PMC10912553; doi:10.1002/ece3.11105)
Supplement: Supplementary file 1 — Figures S1–S98 [file ECE3-14-e11105-s002.zip › ece31105-sup-0001-FigureS1-S48.pdf]

**Supplementary Figure 1**. Habitat network map for Blanding's Turtles (*Emydoidea blandingii*) in Benton Harbor site showing habitat patch size as the size of the circle and importance of the patch in maintaining overall network connectivity with warming colors (orange and red) indicating highest importance.

**Supplementary Figure 2.** Map of locations where removal of a barrier (red areas) would improve connectivity for Blanding's Turtles (*Emydoidea blandingii*) in the Benton Harbor site.

**Supplementary Figure 3.** Map of areas with narrow linkages (yellow and red areas) where Blanding's Turtles (*Emydoidea blandingii*) would have limited movement options in Benton Harbor site making them important corridors to maintain.

**Supplementary Figure 4.** Current density map for Blanding's Turtles (*Emydoidea blandingii*) in Benton Harbor site. Areas with higher current density are predicted to represent better movement corridors.

# S1

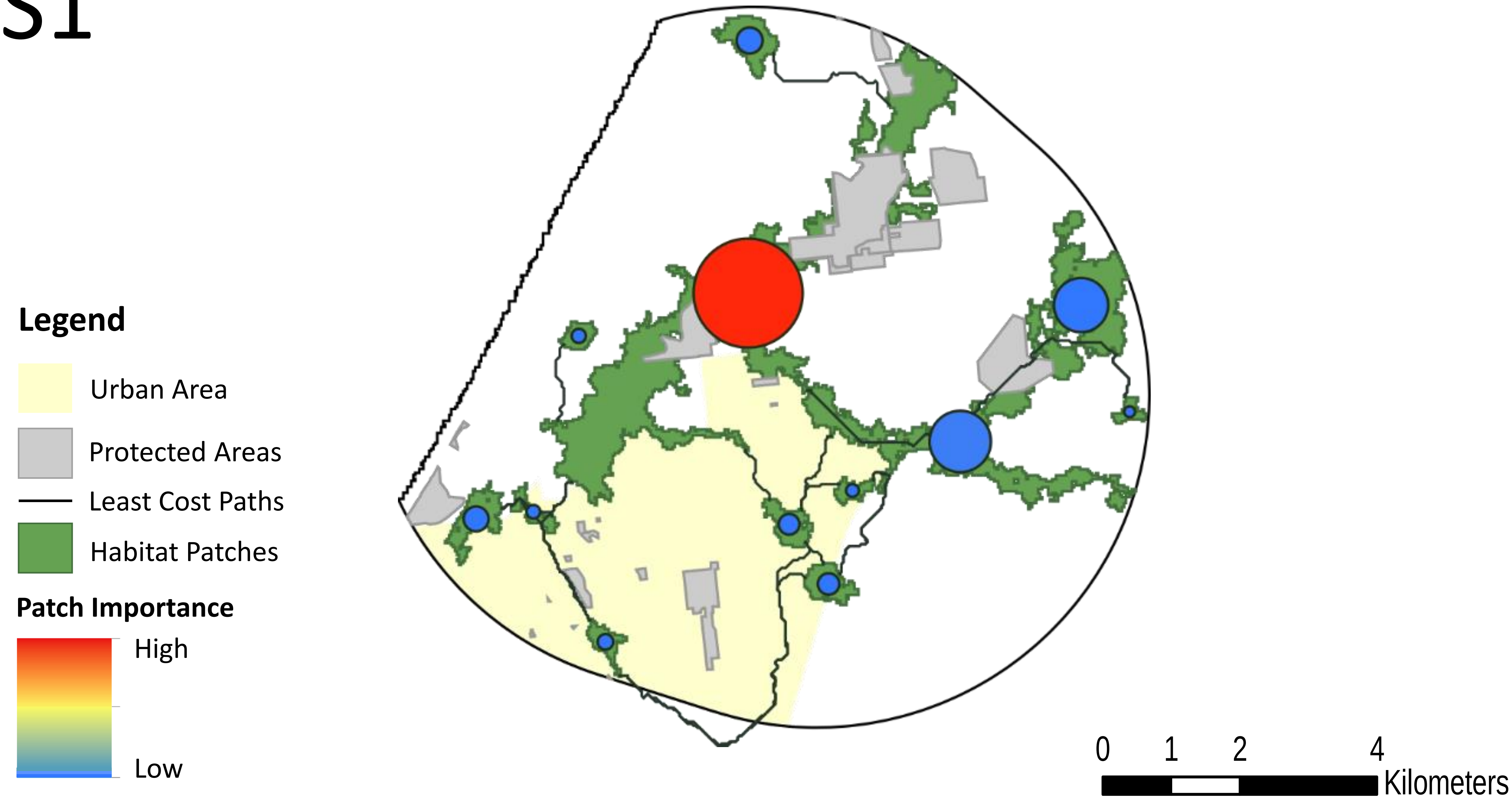
Legend
Urban Area
Protected Areas
Least Cost Paths
Habitat Patches
Patch Importance
High
Low
0
1
2
4
Kilometers

# S2

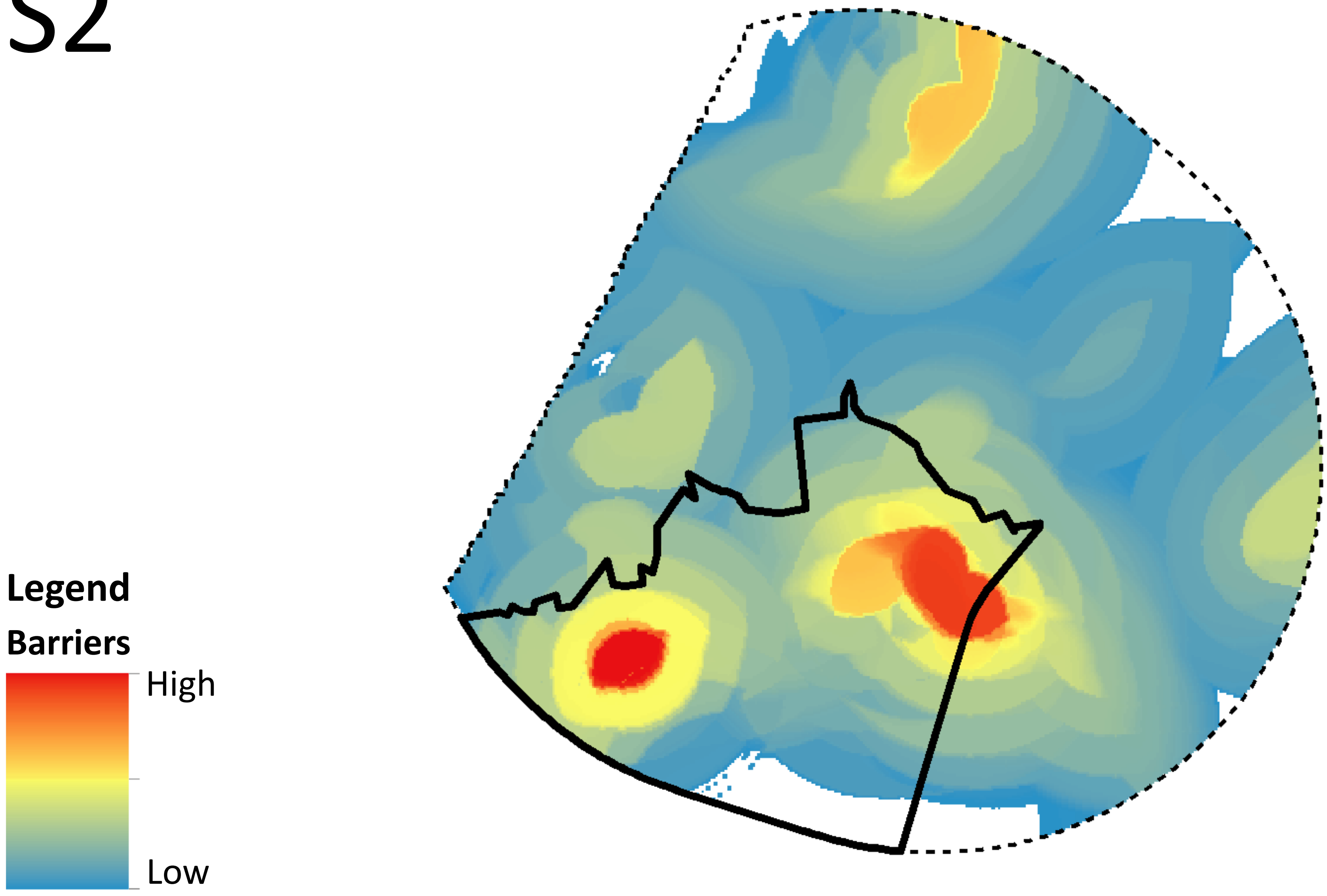

S3

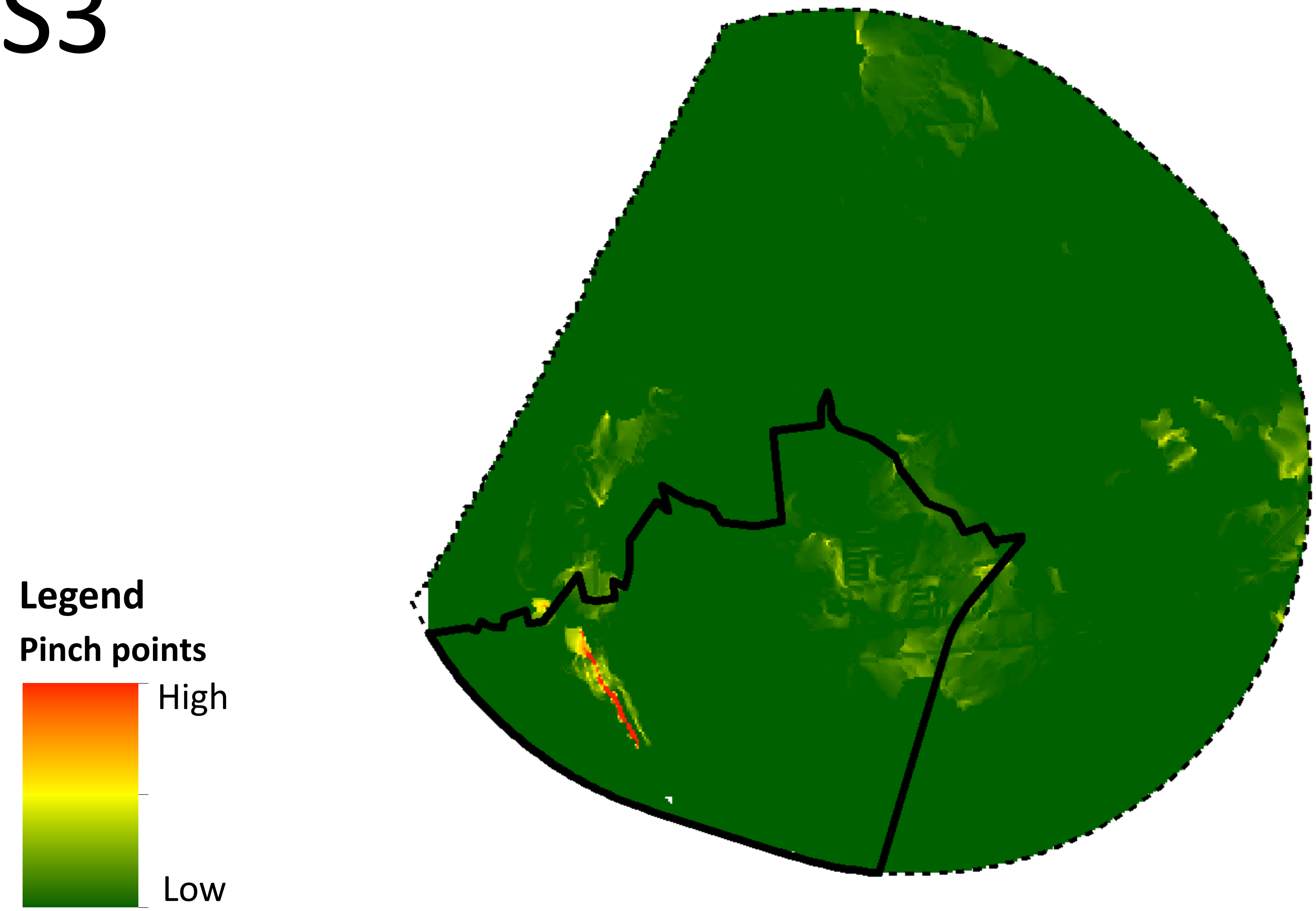

# S4

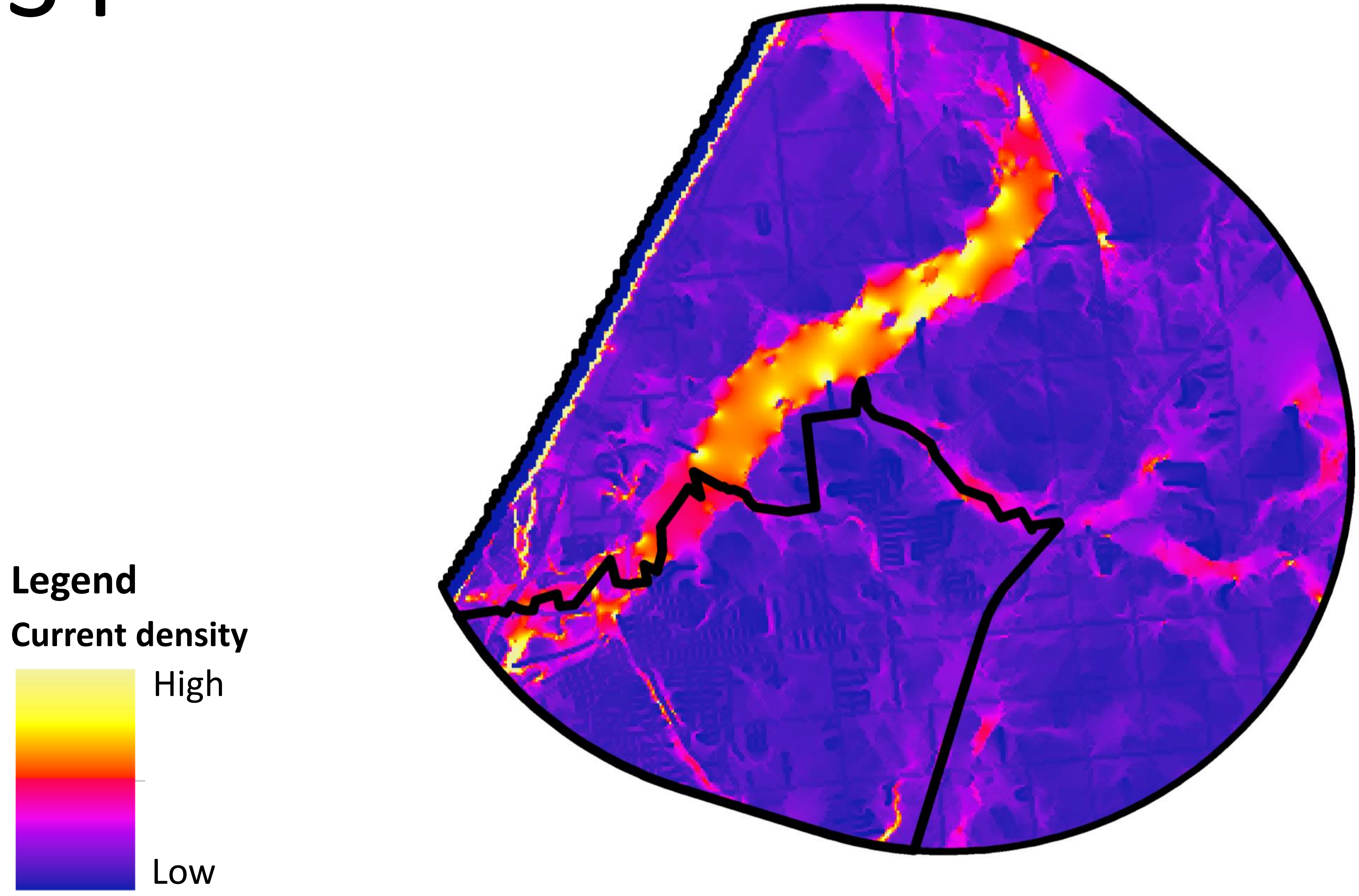

**Supplementary Figure 5**. Habitat network map for Eastern Box Turtles (*Terrapene carolina carolina*) in Benton Harbor site showing habitat patch size as the size of the circle and importance of the patch in maintaining overall network connectivity with warming colors (orange and red) indicating highest importance.

**Supplementary Figure 6.** Map of locations where removal of a barrier (red areas) would improve connectivity for Eastern Box Turtles (*Terrapene carolina carolina*) in the Benton Harbor site.

**Supplementary Figure 7.** Map of areas with narrow linkages (yellow and red areas) where Eastern Box Turtles (*Terrapene carolina carolina*) would have limited movement options in Benton Harbor site making them important corridors to maintain.

**Supplementary Figure 8.** Current density map for Eastern Box Turtles (*Terrapene carolina carolina*) in Benton Harbor site. Areas with higher current density are predicted to represent better movement corridors.

# S5

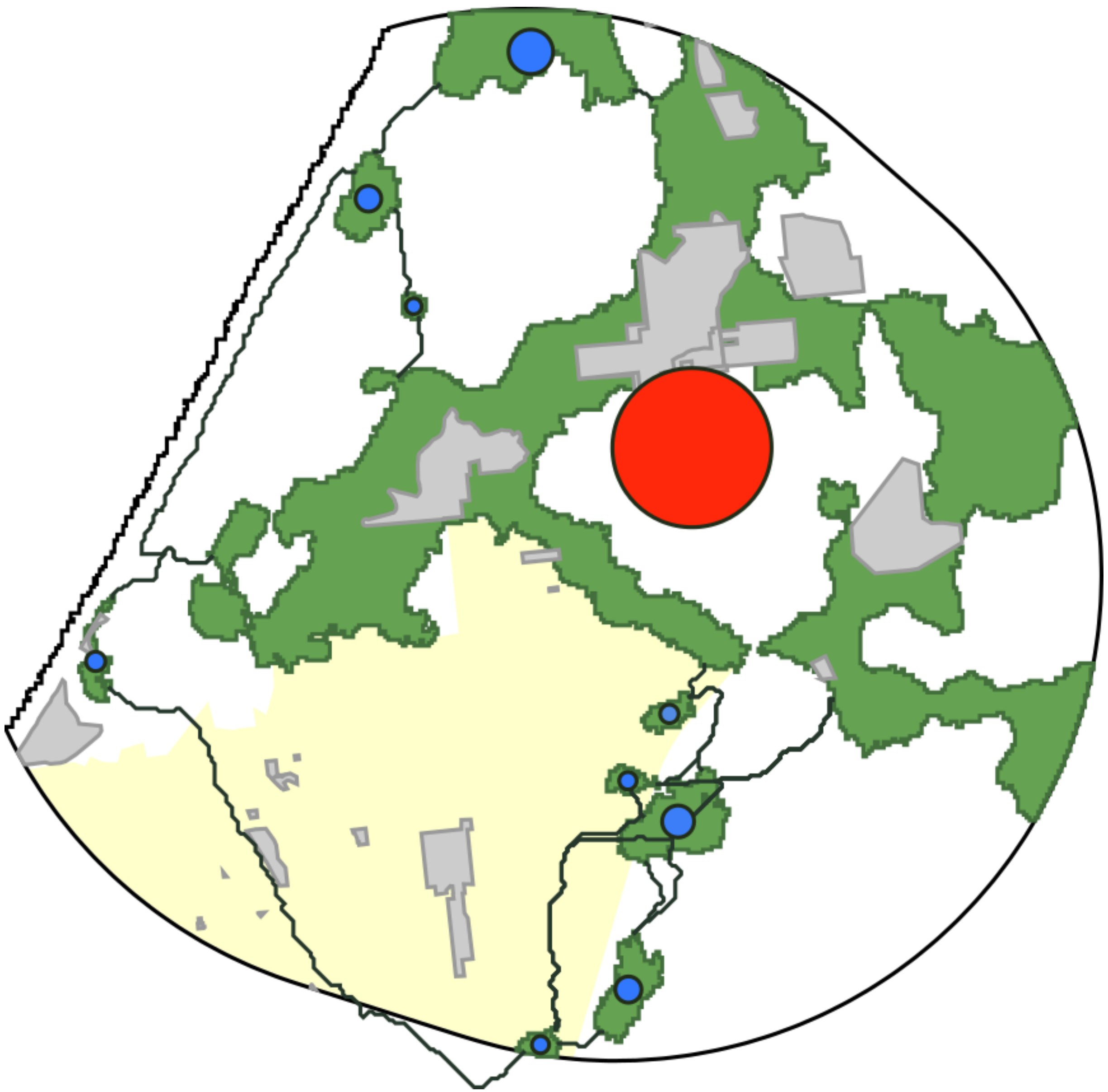

# S6

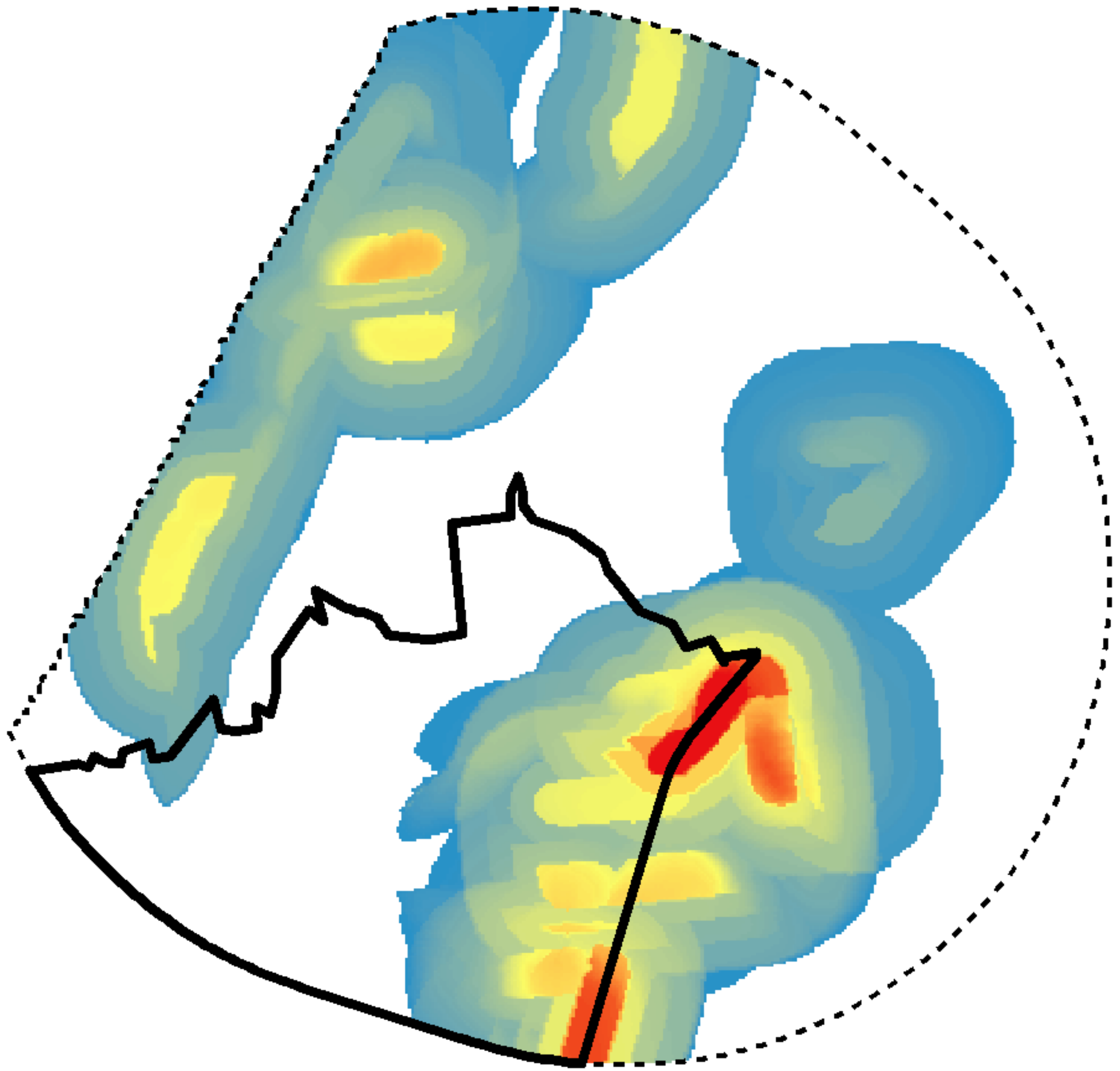

# S7

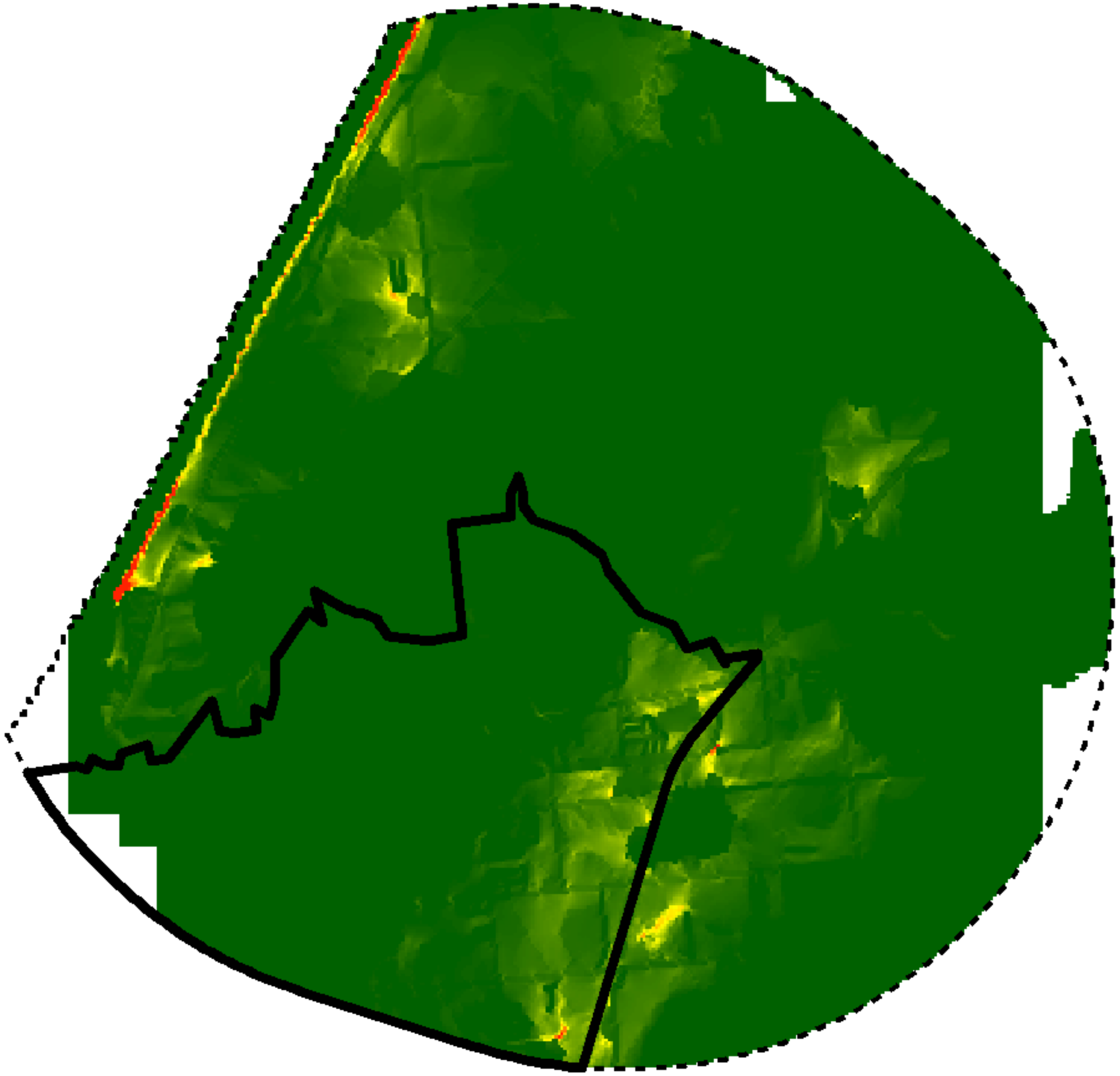

S8

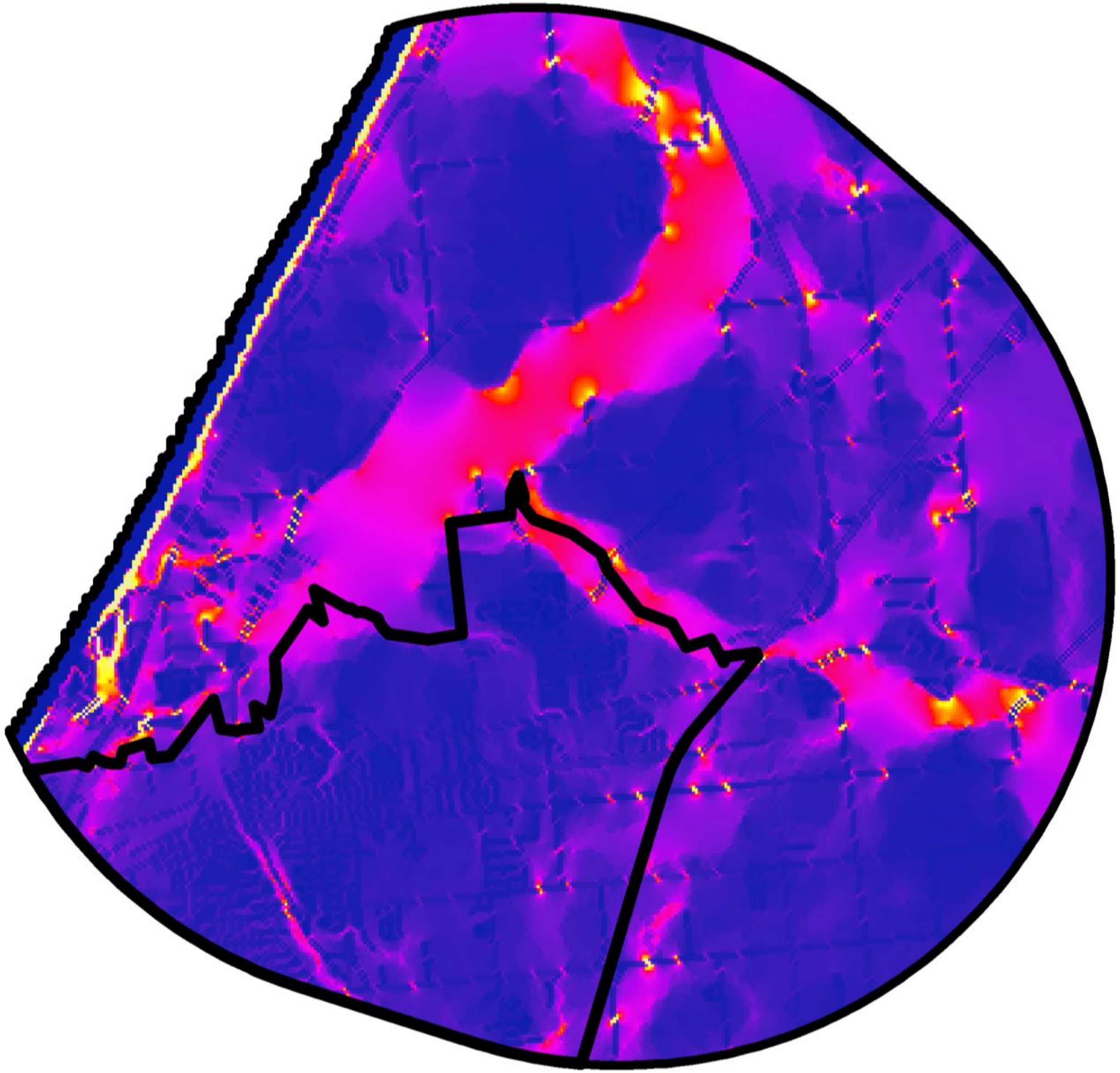

**Supplementary Figure 9**. Habitat network map for Spotted Turtles (*Clemmys guttata*) in Benton Harbor site showing habitat patch size as the size of the circle and importance of the patch in maintaining overall network connectivity with warming colors (orange and red) indicating highest importance.

**Supplementary Figure 10.** Map of locations where removal of a barrier (red areas) would improve connectivity for Spotted Turtles (*Clemmys guttata*) in the Benton Harbor site.

**Supplementary Figure 11.** Map of areas with narrow linkages (yellow and red areas) where Spotted Turtles (*Clemmys guttata*) would have limited movement options in Benton Harbor site making them important corridors to maintain.

**Supplementary Figure 12.** Current density map for Spotted Turtles (*Clemmys guttata*) in Benton Harbor site. Areas with higher current density are predicted to represent better movement corridors.

# S9

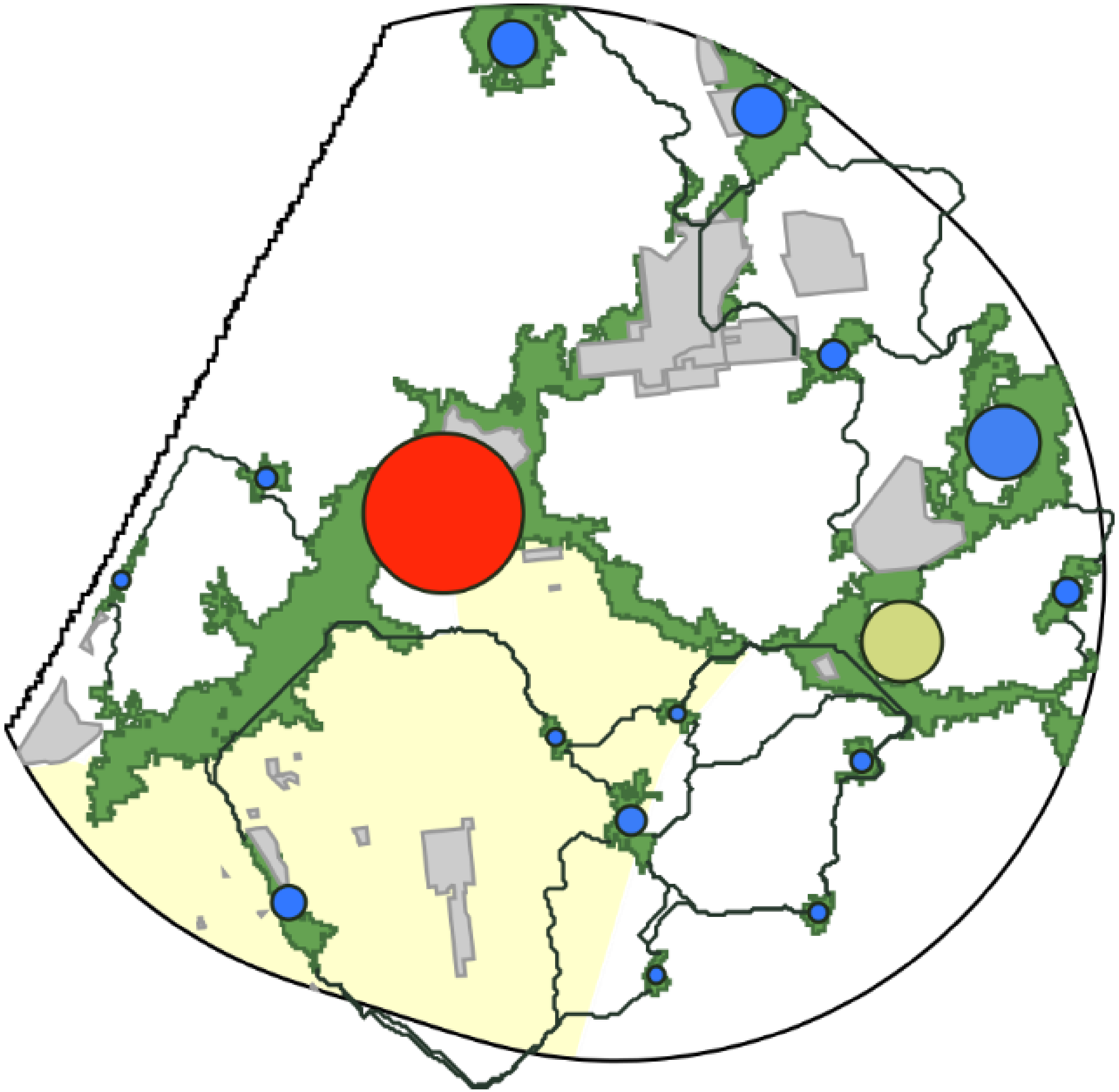

S10

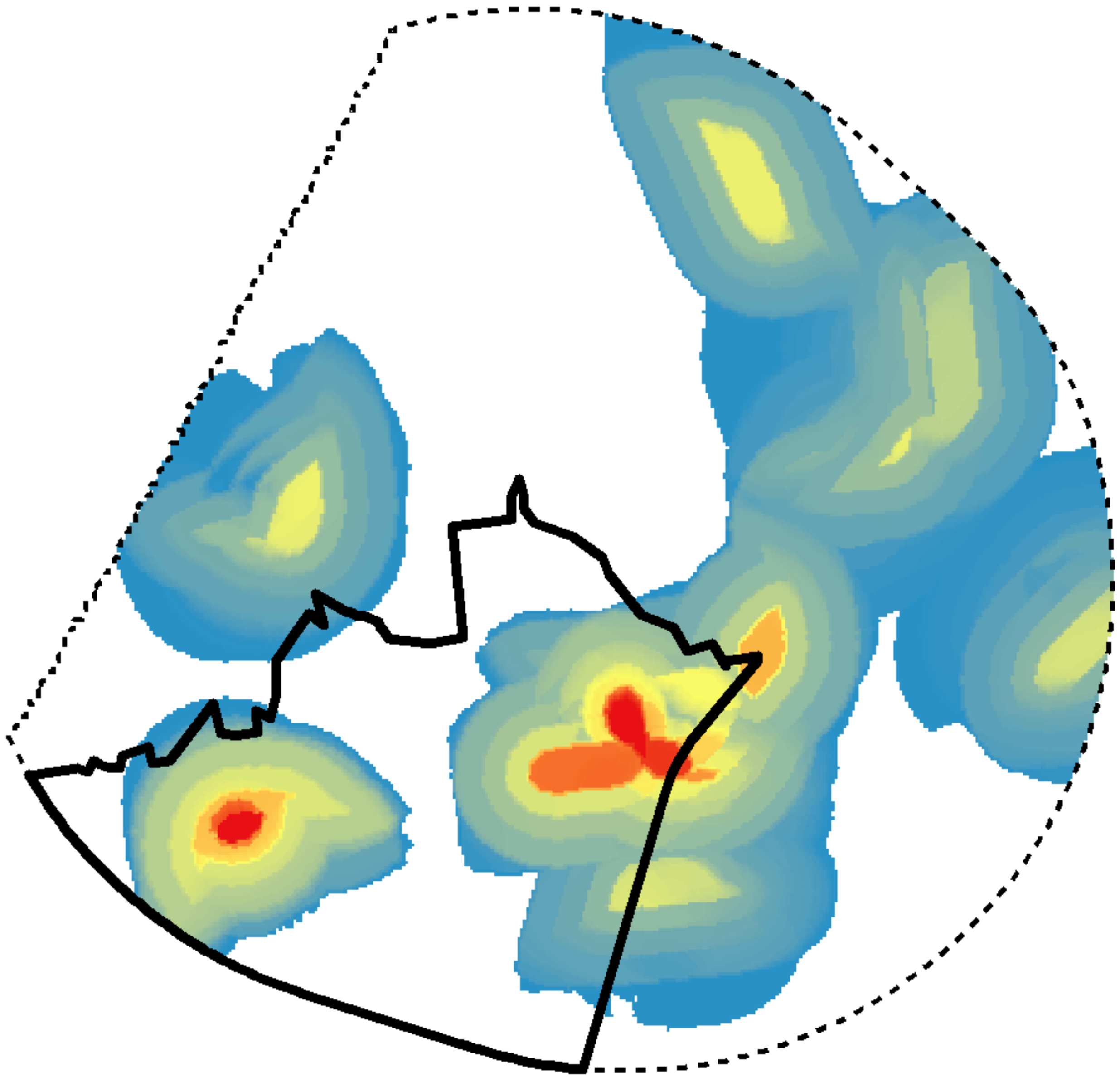

S11

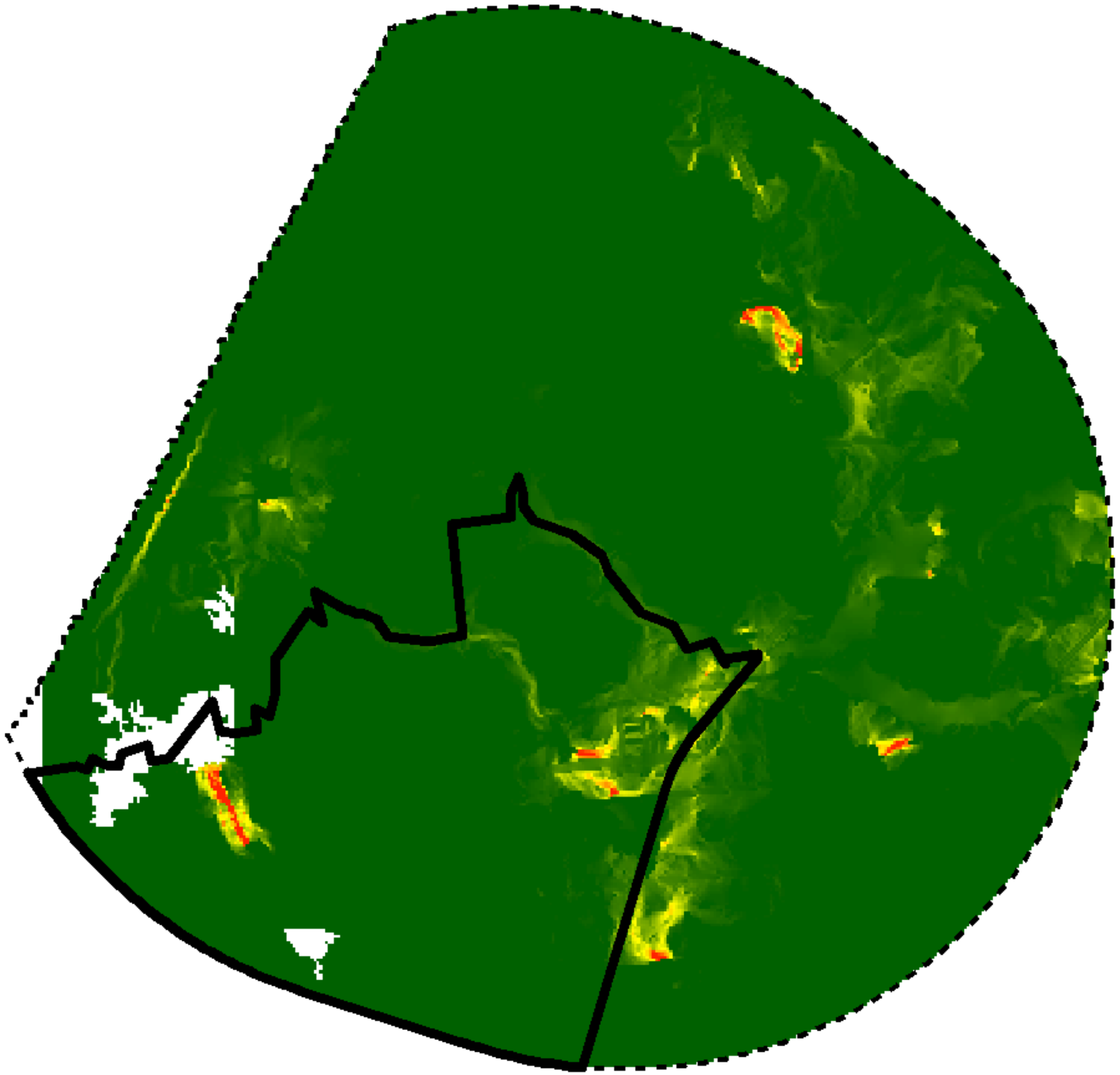

# S12

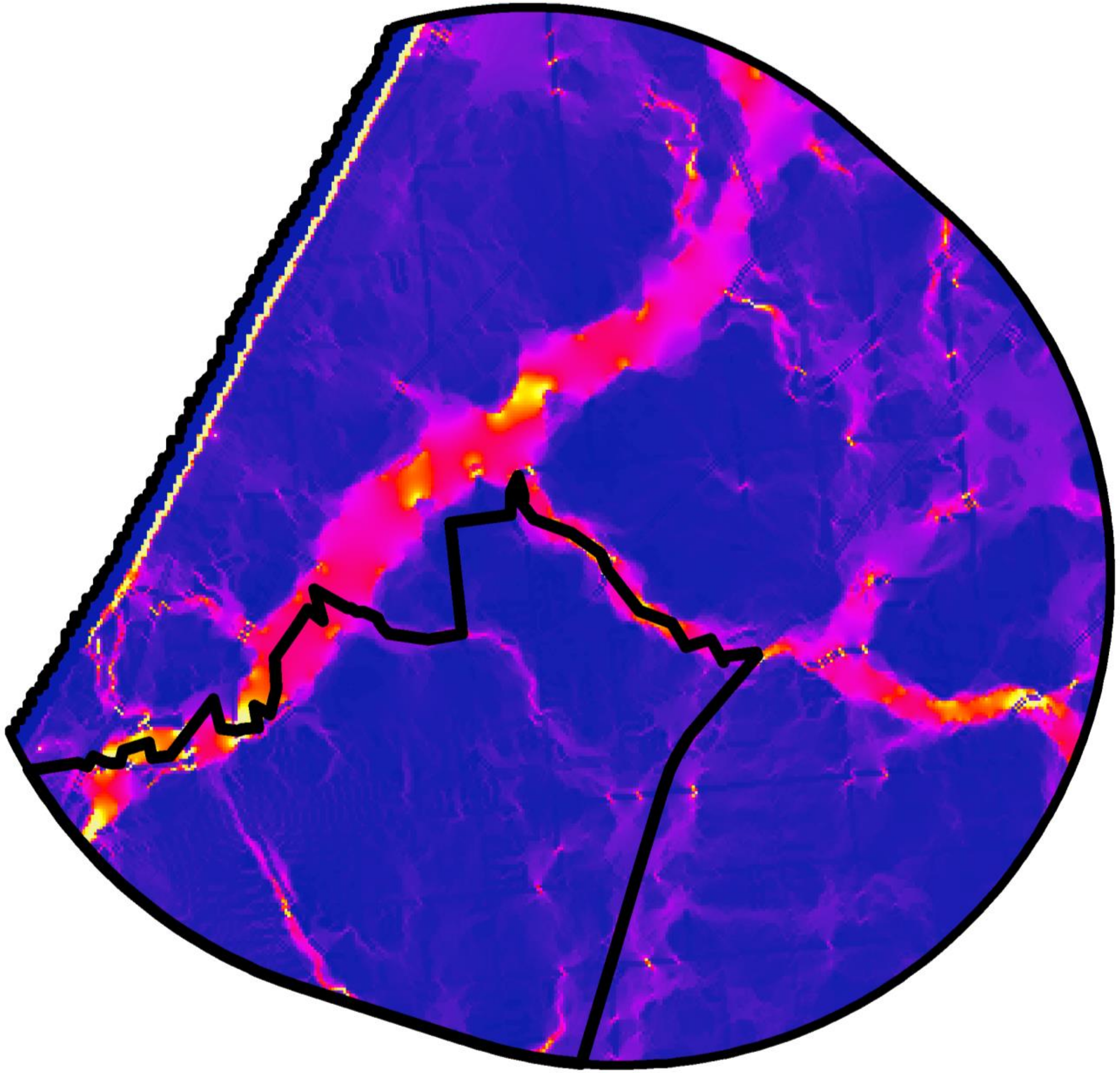

**Supplementary Figure 13**. Habitat network map for Blanding's Turtles (*Emydoidea blandingii*) in Kalamazoo site showing habitat patch size as the size of the circle and importance of the patch in maintaining overall network connectivity with warming colors (orange and red) indicating highest importance.

**Supplementary Figure 14.** Map of locations where removal of a barrier (red areas) would improve connectivity for Blanding's Turtles (*Emydoidea blandingii*) in the Kalamazoo site.

**Supplementary Figure 15.** Map of areas with narrow linkages (yellow and red areas) where Blanding's Turtles (*Emydoidea blandingii*) would have limited movement options in Kalamazoo site making them important corridors to maintain.

**Supplementary Figure 16.** Current density map for Blanding's Turtles (*Emydoidea blandingii*) in Kalamazoo site. Areas with higher current density are predicted to represent better movement corridors.

# S13

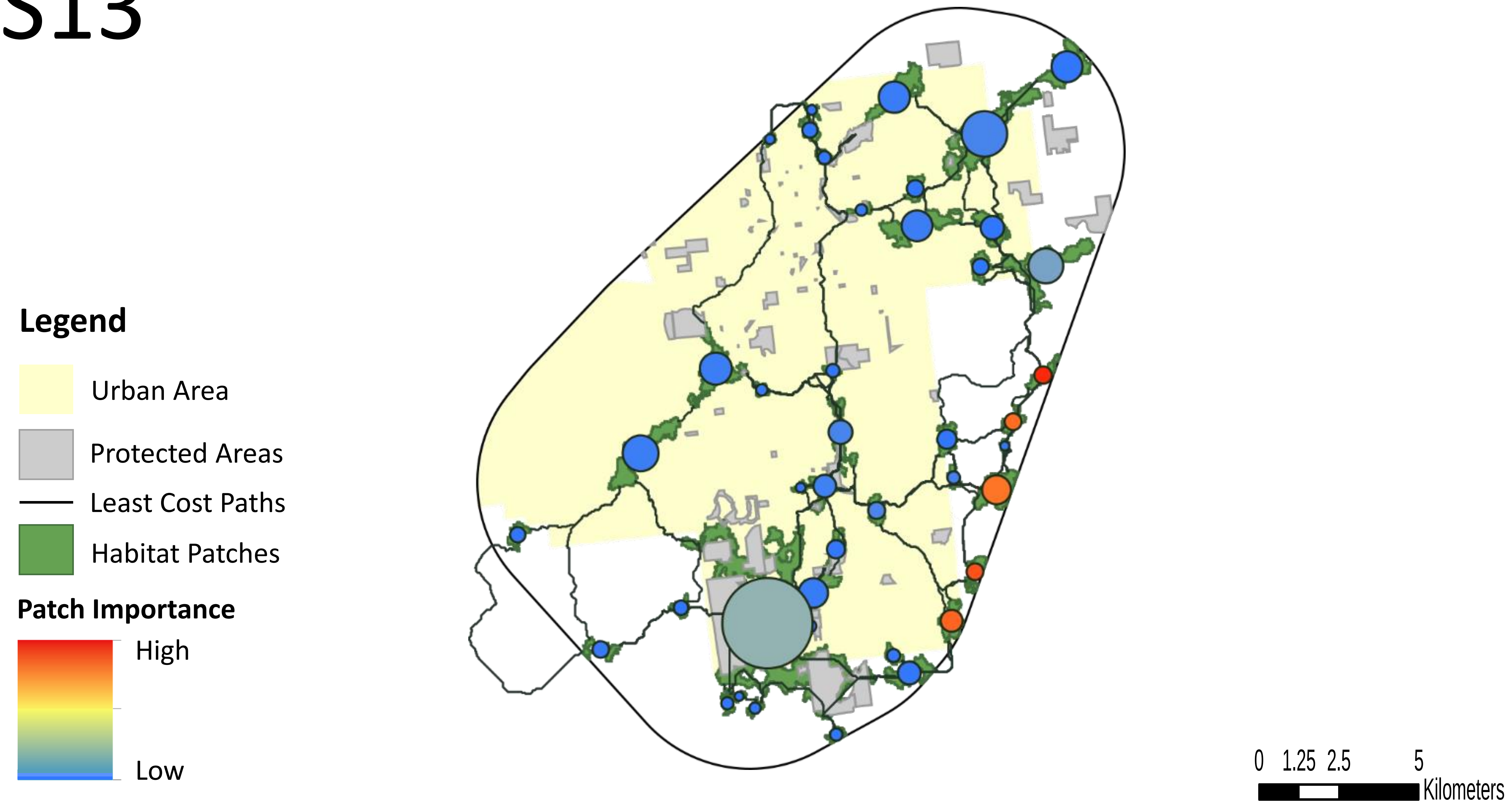
Legend
Urban Area
Protected Areas
Least Cost Paths
Habitat Patches
Patch Importance
High
Low
0 1.25 2.5 5
Kilometers

# S14

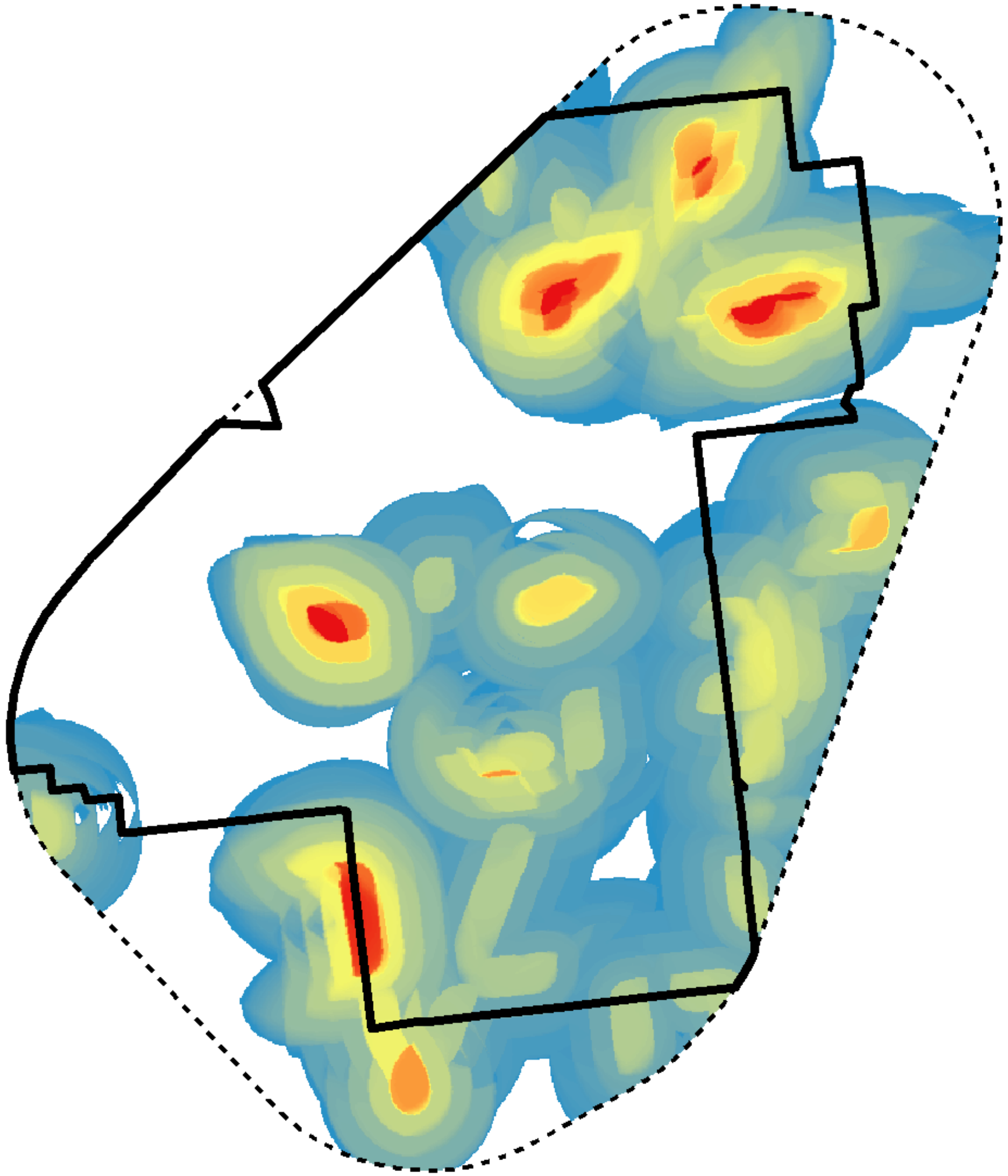

**Legend**

**Barriers**

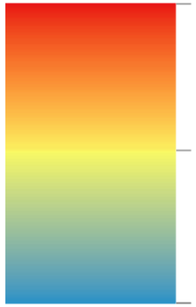

High

Low

# S15

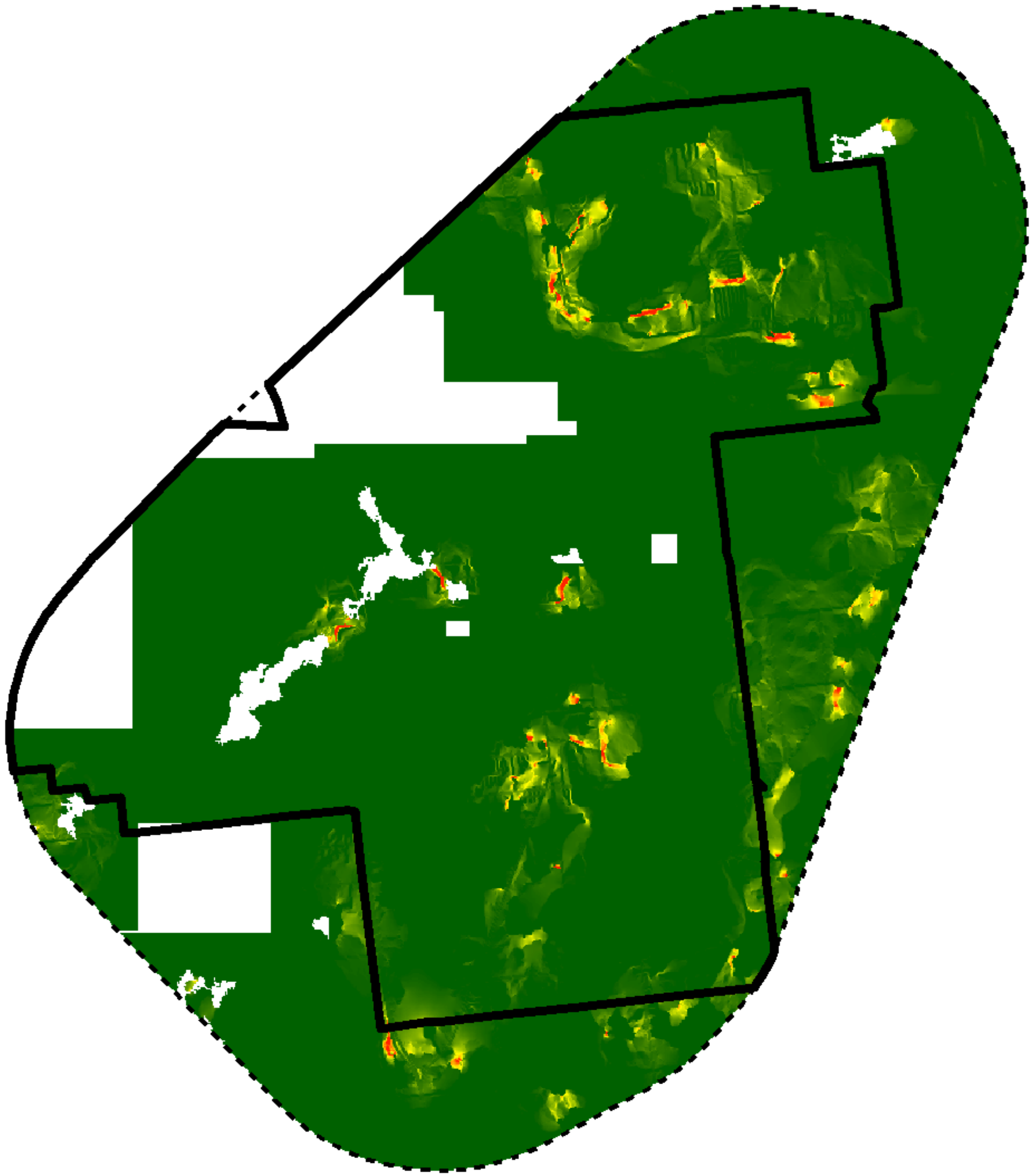

**Legend**

**Pinch points**

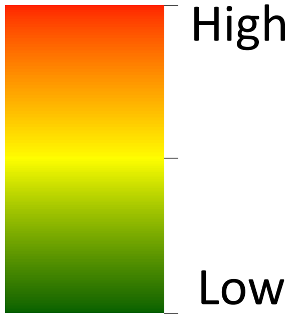

# S16

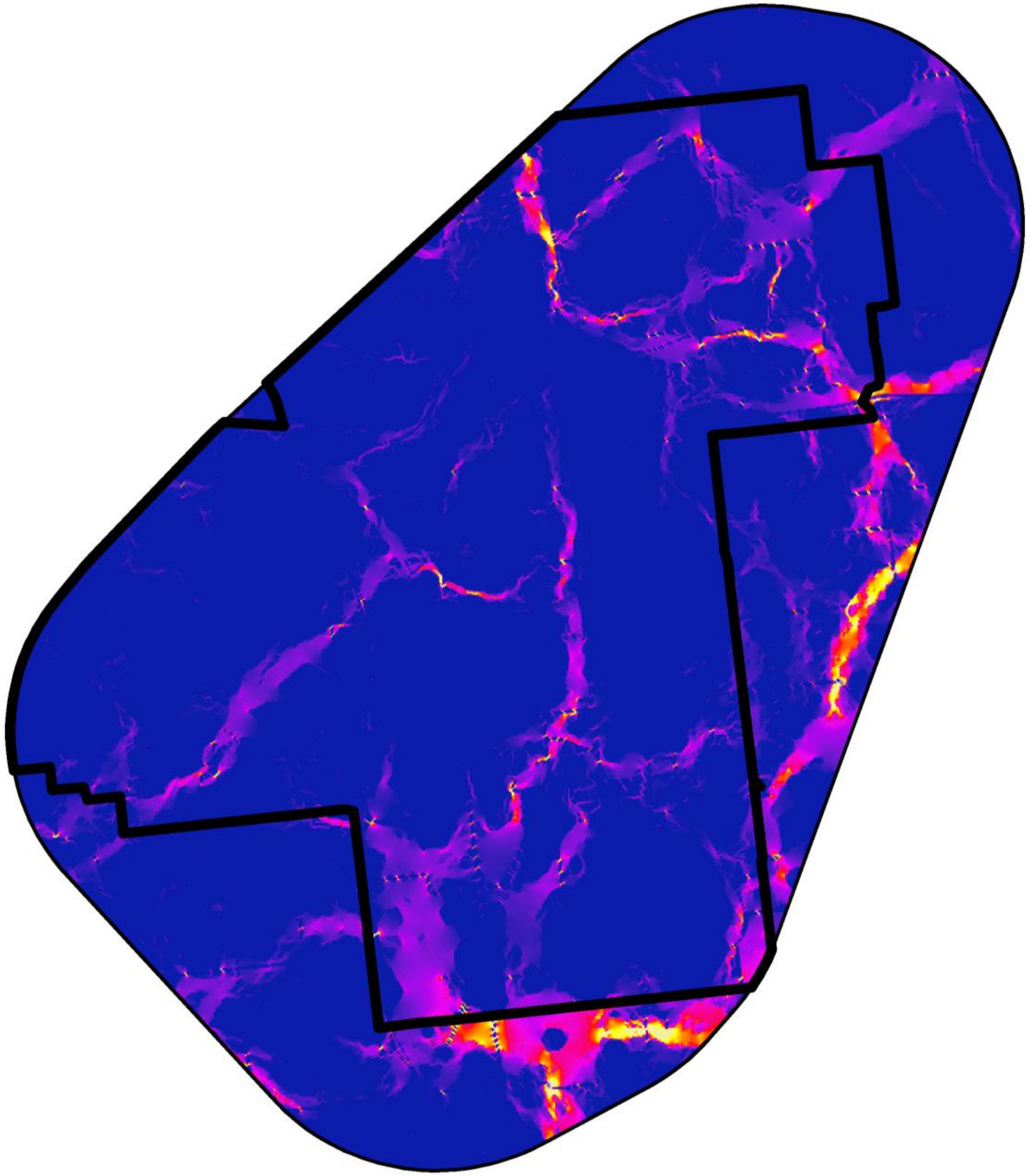

**Legend**

**Current density**

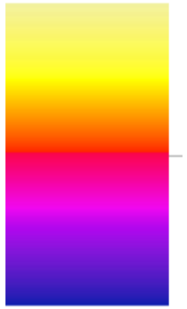

**Supplementary Figure 17**. Habitat network map for Eastern Box Turtles (*Terrapene carolina carolina*) in Kalamazoo site showing habitat patch size as the size of the circle and importance of the patch in maintaining overall network connectivity with warming colors (orange and red) indicating highest importance.

**Supplementary Figure 18.** Map of locations where removal of a barrier (red areas) would improve connectivity for Eastern Box Turtles (*Terrapene carolina carolina*) in the Kalamazoo site.

**Supplementary Figure 19.** Map of areas with narrow linkages (yellow and red areas) where Eastern Box Turtles (*Terrapene carolina carolina*) would have limited movement options in Kalamazoo site making them important corridors to maintain.

**Supplementary Figure 20.** Current density map for Eastern Box Turtles (*Terrapene carolina carolina*) in Kalamazoo site. Areas with higher current density are predicted to represent better movement corridors.

# S17

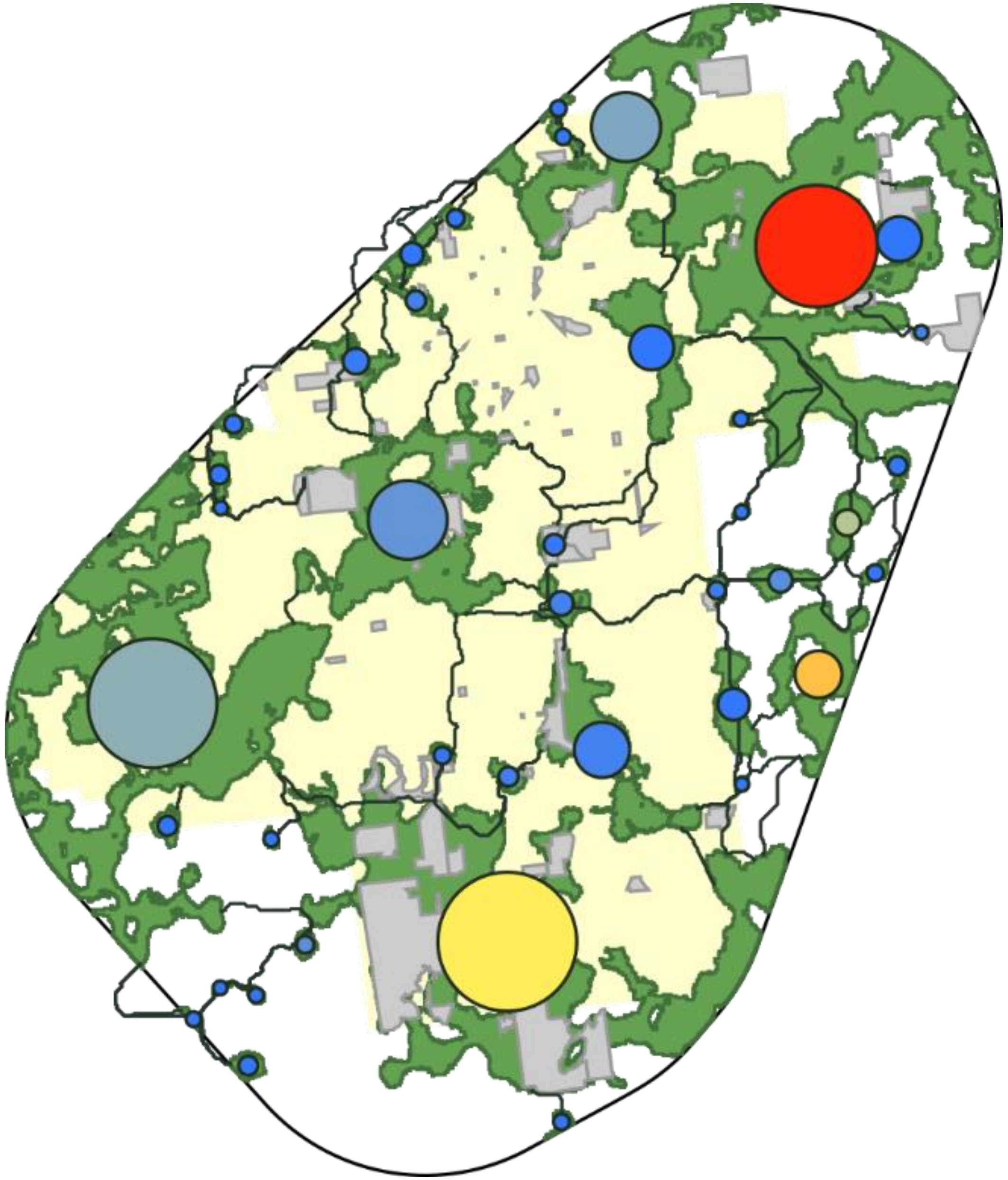

S18

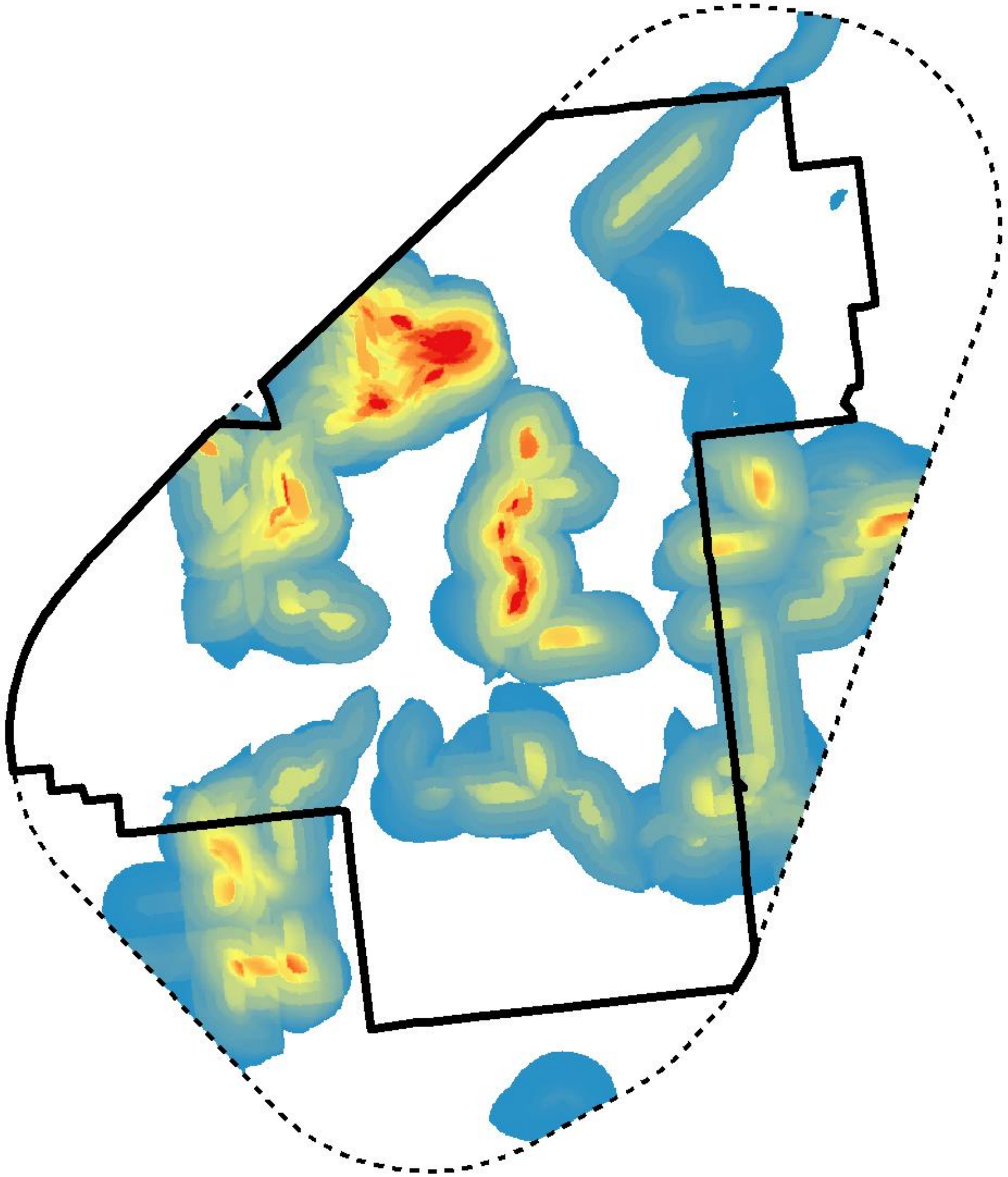

S19

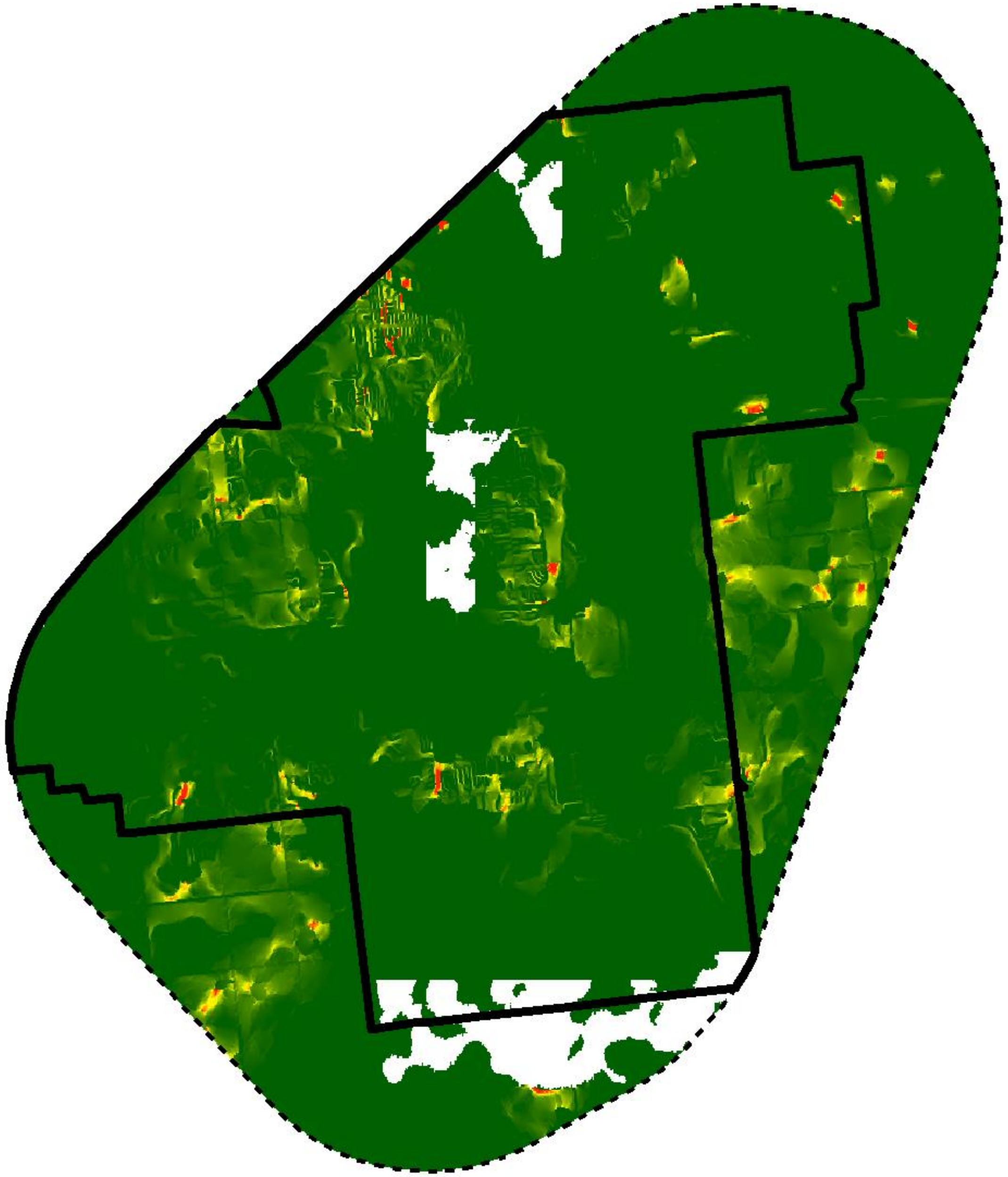

# S20

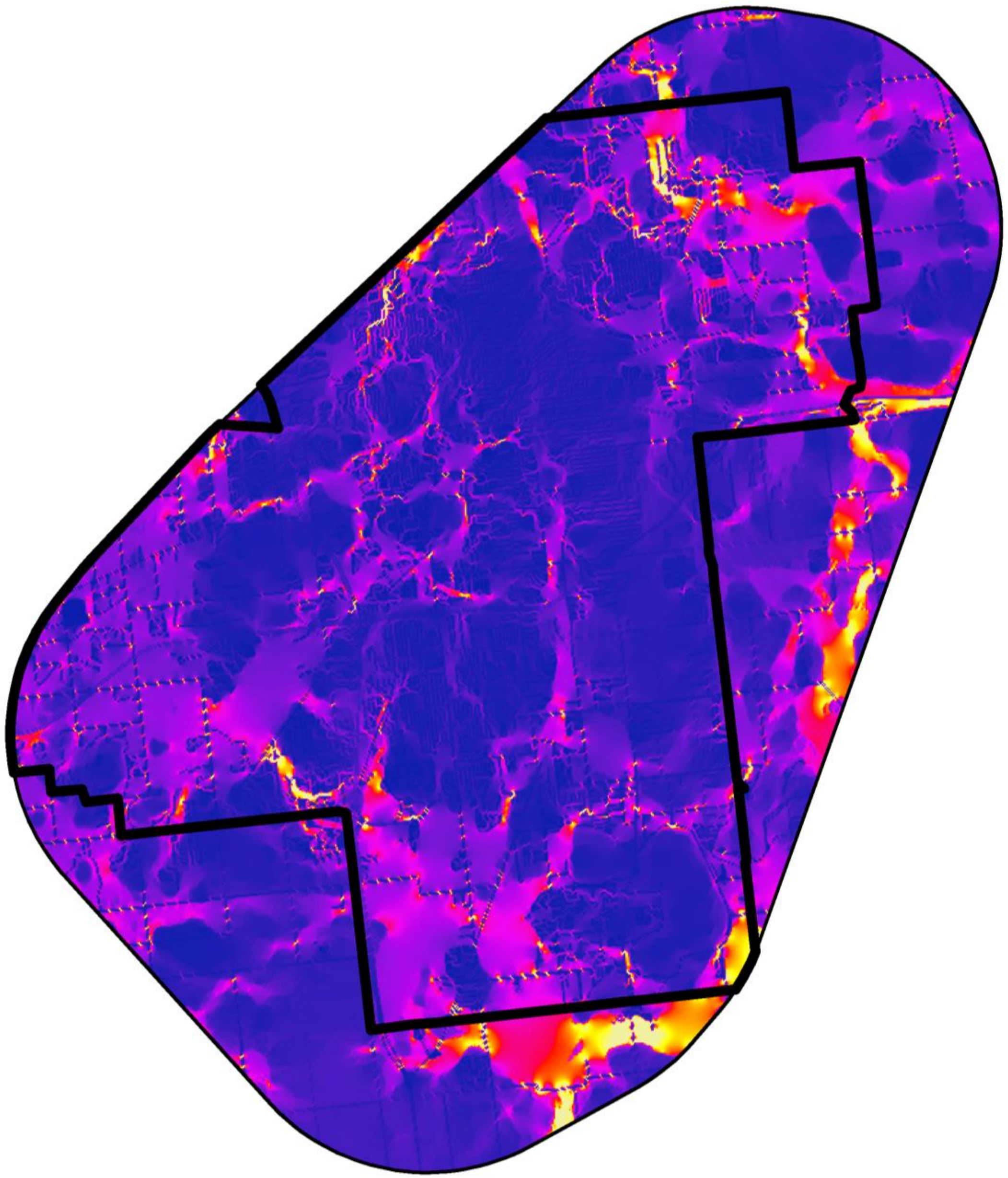

**Supplementary Figure 21**. Habitat network map for Eastern Massasauga Rattlesnakes (*Sistrurus catenatus*) in Kalamazoo site showing habitat patch size as the size of the circle and importance of the patch in maintaining overall network connectivity with warming colors (orange and red) indicating highest importance.

**Supplementary Figure 22.** Map of locations where removal of a barrier (red areas) would improve connectivity for Eastern Massasauga Rattlesnakes (*Sistrurus catenatus*) in the Kalamazoo site.

**Supplementary Figure 23.** Map of areas with narrow linkages (yellow and red areas) where Eastern Massasauga Rattlesnakes (*Sistrurus catenatus*) would have limited movement options in Kalamazoo site making them important corridors to maintain.

**Supplementary Figure 24.** Current density map for Eastern Massasauga Rattlesnakes (*Sistrurus catenatus*) in Kalamazoo site. Areas with higher current density are predicted to represent better movement corridors.

# S21

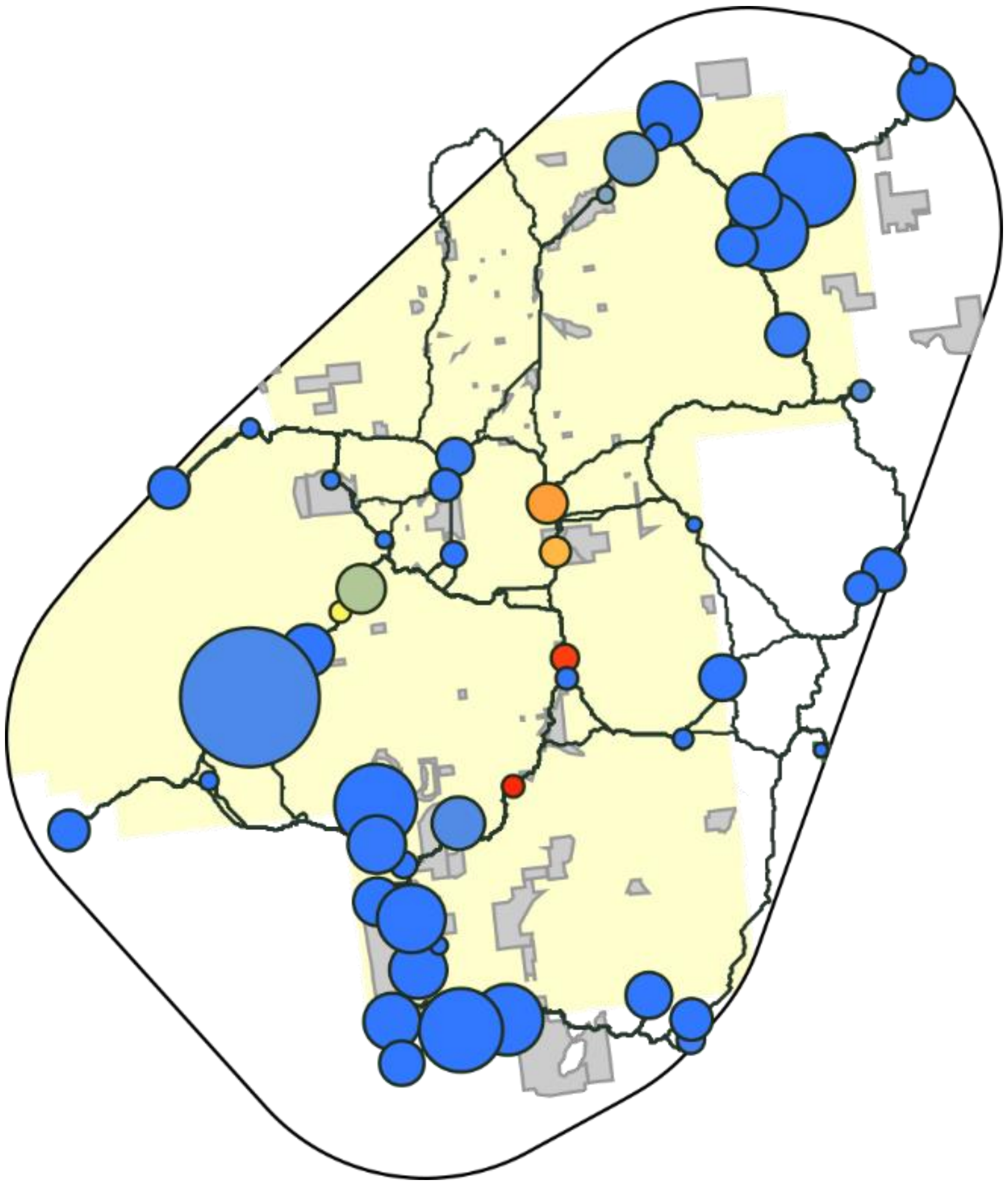

# S22

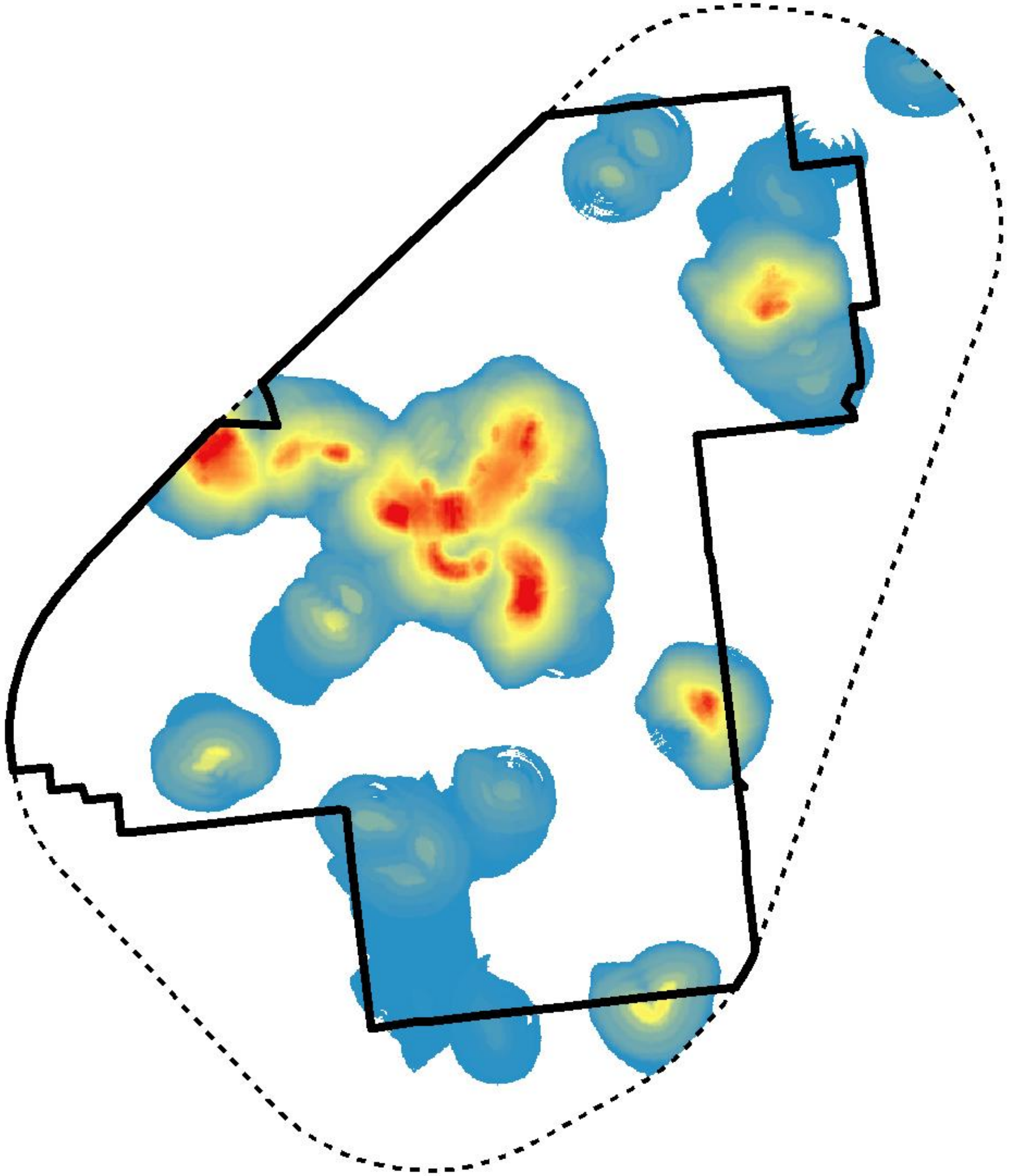

# S23

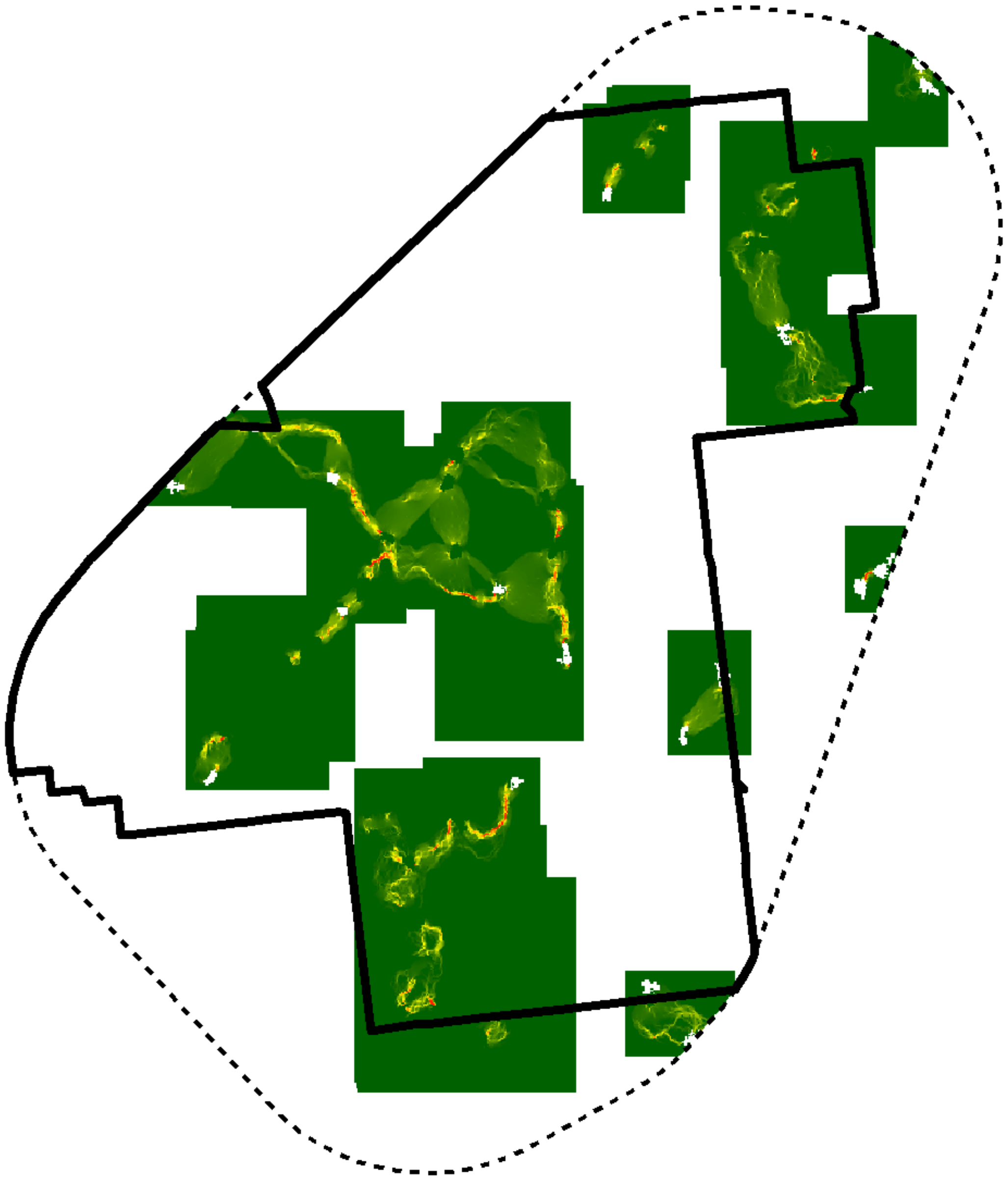

# S24

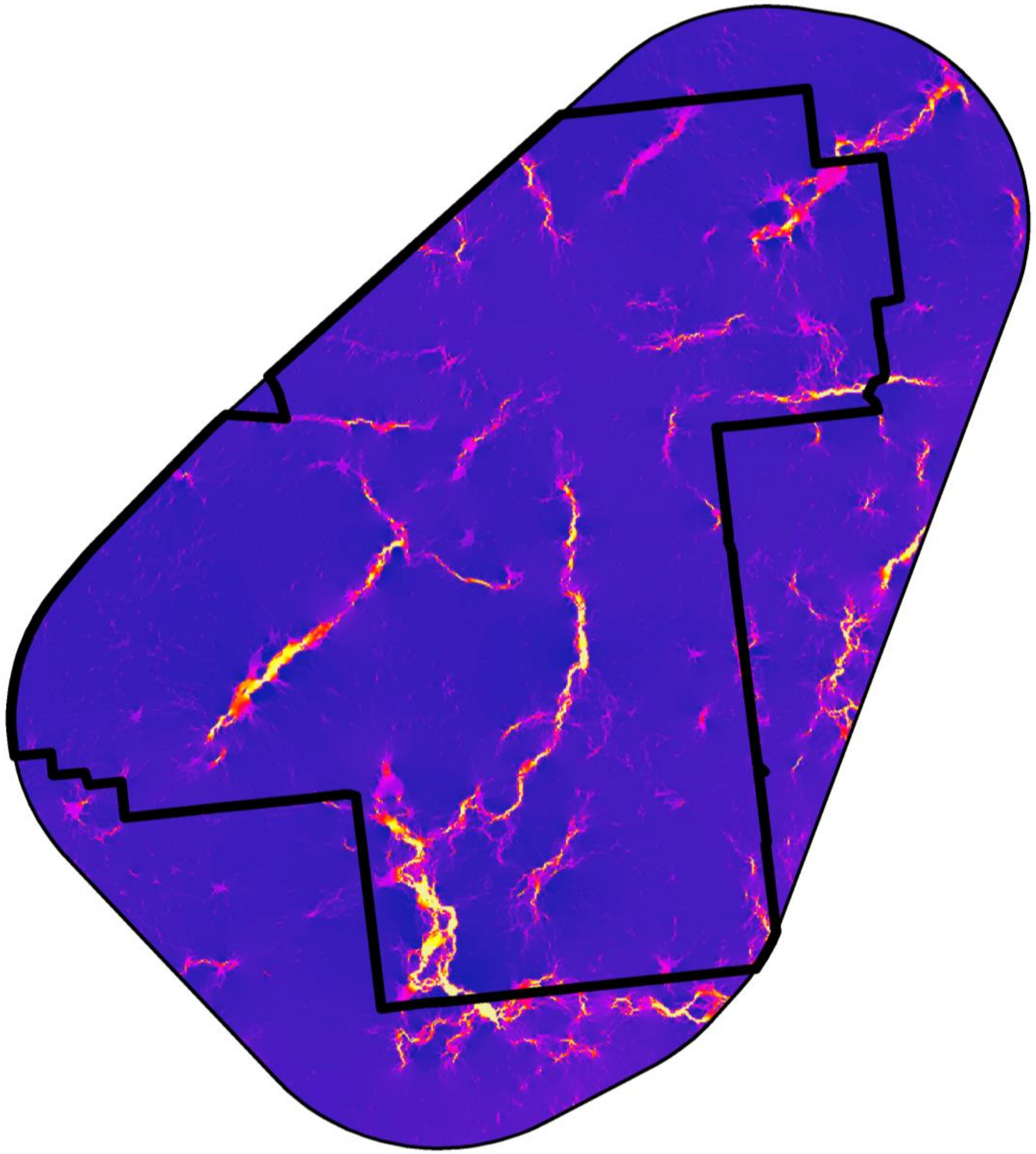

**Supplementary Figure 25**. Habitat network map for Henslow's Sparrows (*Ammodramus henslowii*) in Kalamazoo site showing habitat patch size as the size of the circle and importance of the patch in maintaining overall network connectivity with warming colors (orange and red) indicating highest importance.

**Supplementary Figure 26.** Map of locations where removal of a barrier (red areas) would improve connectivity for Henslow's Sparrows (*Ammodramus henslowii*) in the Kalamazoo site.

**Supplementary Figure 27.** Map of areas with narrow linkages (yellow and red areas) where Henslow's Sparrows (*Ammodramus henslowii*) would have limited movement options in Kalamazoo site making them important corridors to maintain.

**Supplementary Figure 28.** Current density map for Henslow's Sparrows (*Ammodramus henslowii*) in Kalamazoo site. Areas with higher current density are predicted to represent better movement corridors.

S25

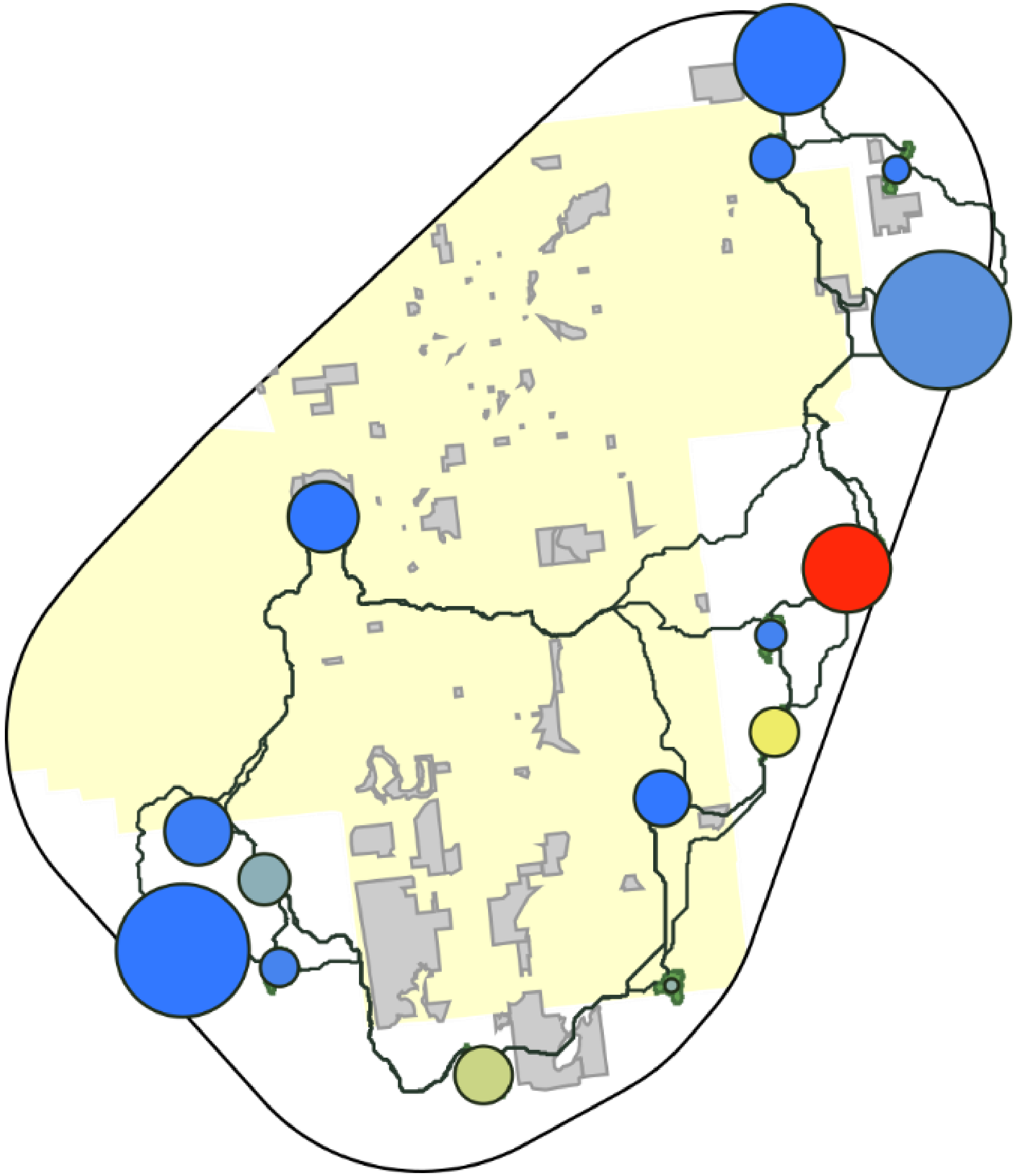

# S26

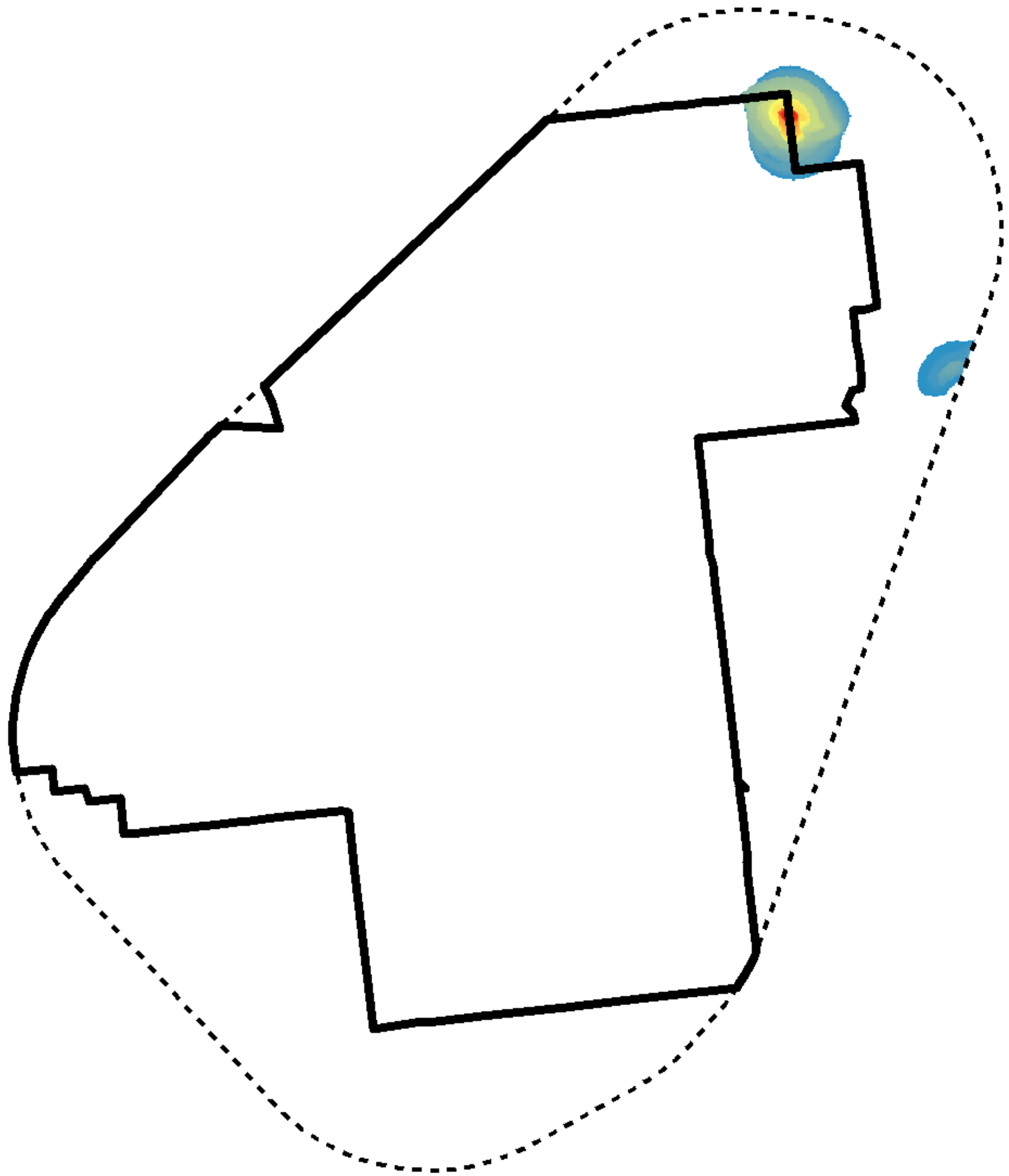

# S27

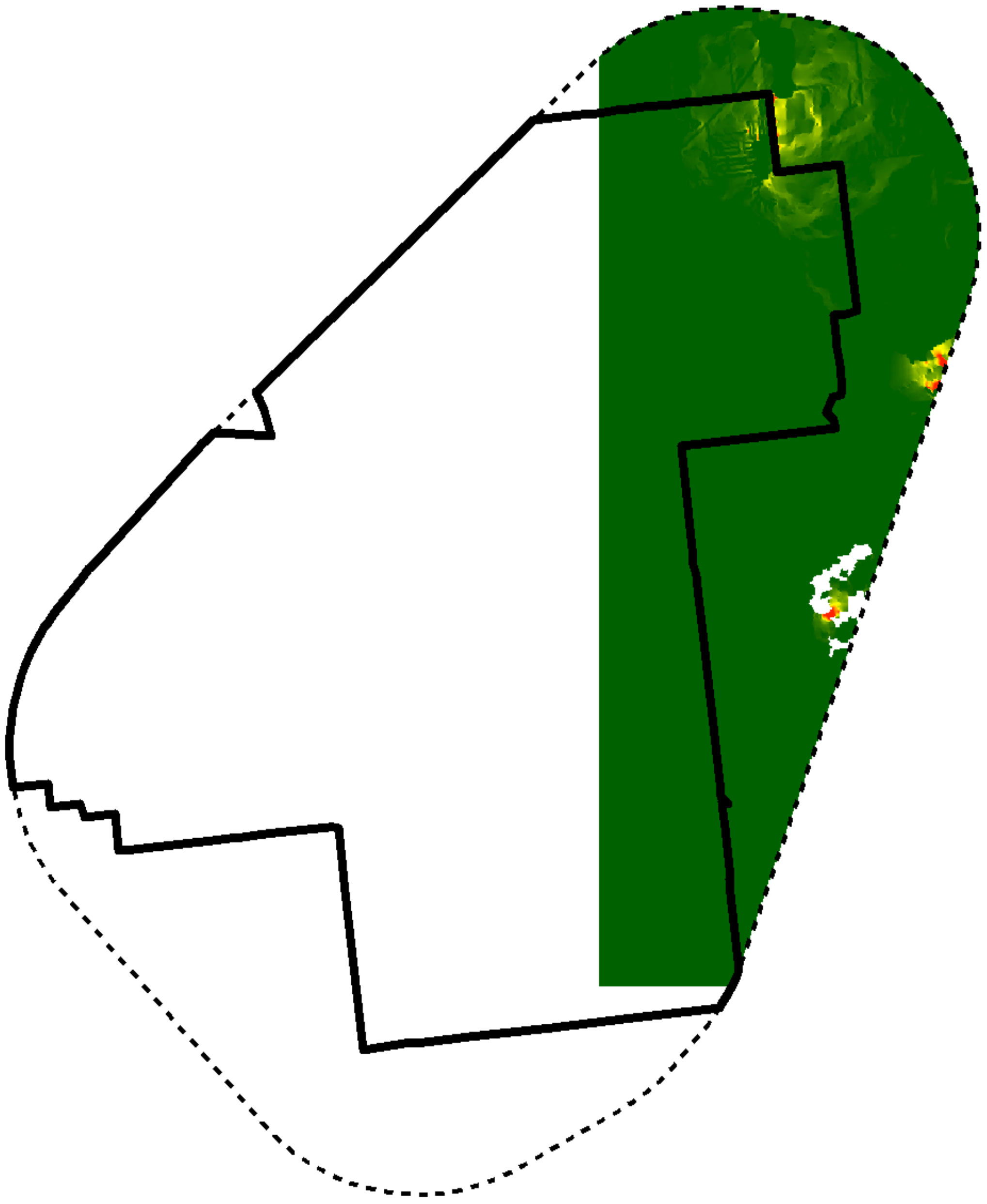

# S28

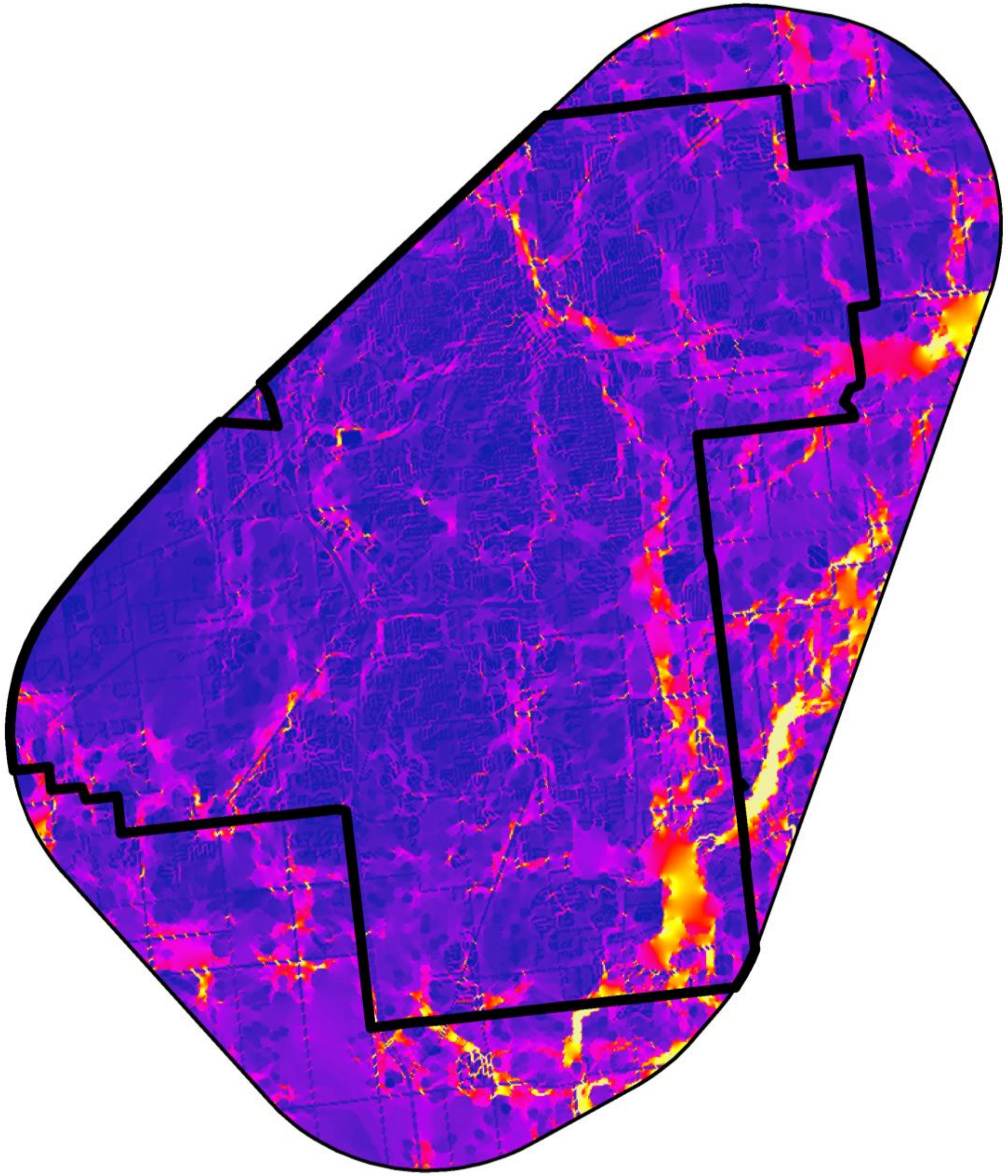

**Supplementary Figure 29**. Habitat network map for Spotted Turtles (*Clemmys guttata*) in Kalamazoo site showing habitat patch size as the size of the circle and importance of the patch in maintaining overall network connectivity with warming colors (orange and red) indicating highest importance.

**Supplementary Figure 30.** Map of locations where removal of a barrier (red areas) would improve connectivity for Spotted Turtles (*Clemmys guttata*) in the Kalamazoo site.

**Supplementary Figure 31.** Map of areas with narrow linkages (yellow and red areas) where Spotted Turtles (*Clemmys guttata*) would have limited movement options in Kalamazoo site making them important corridors to maintain.

**Supplementary Figure 32.** Current density map for Spotted Turtles (*Clemmys guttata*) in Kalamazoo site. Areas with higher current density are predicted to represent better movement corridors.

# S29

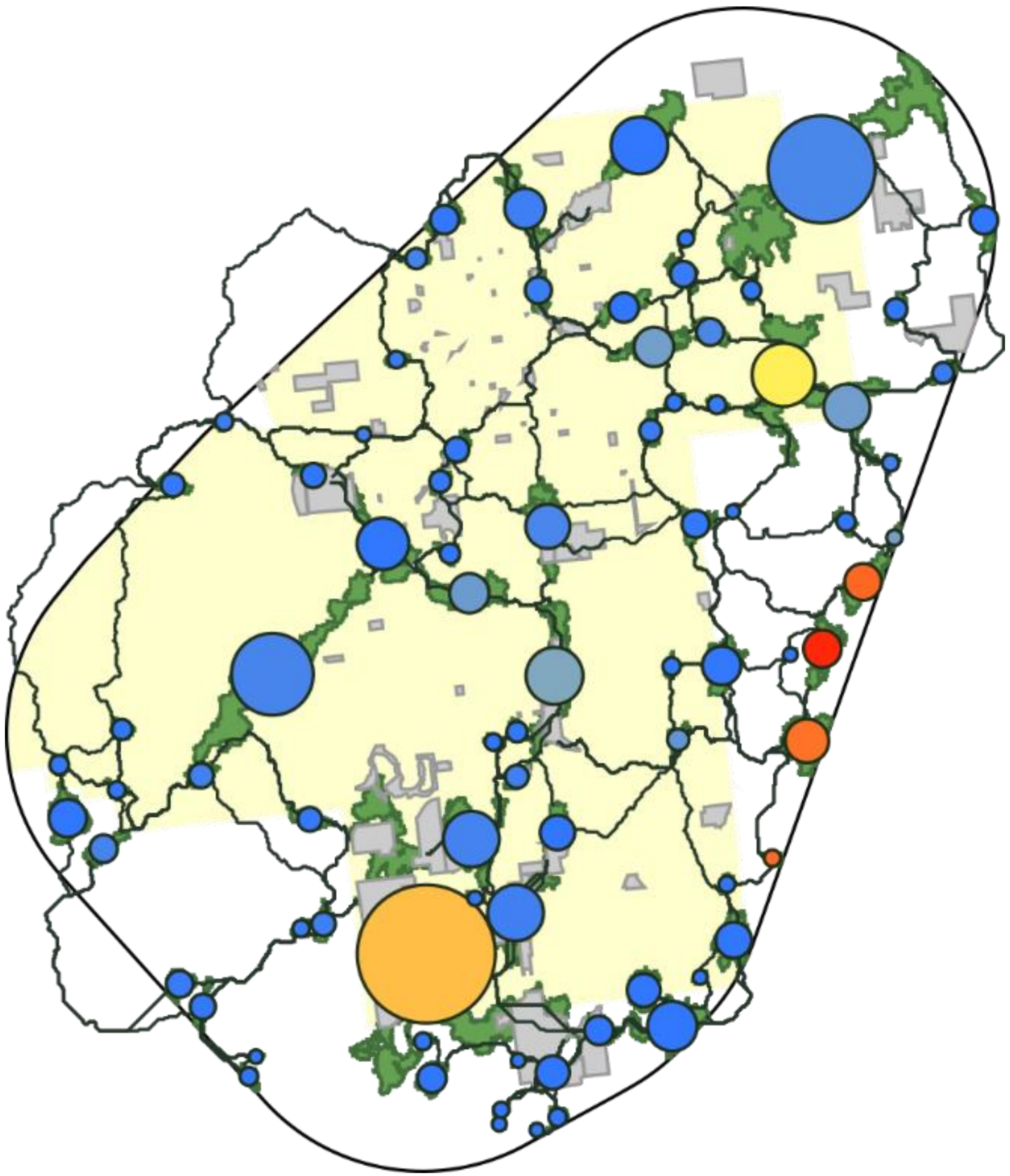

S30

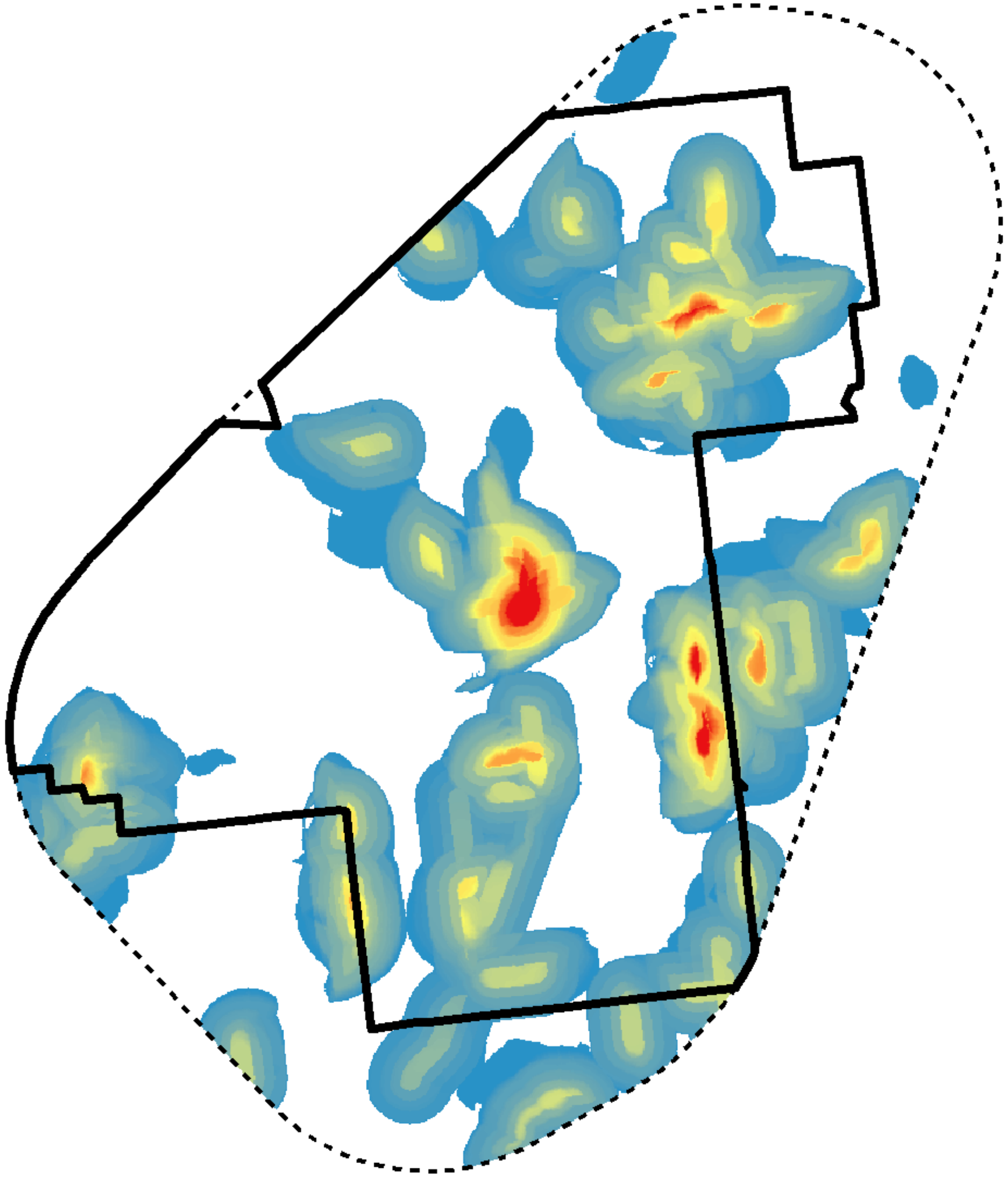

# S31

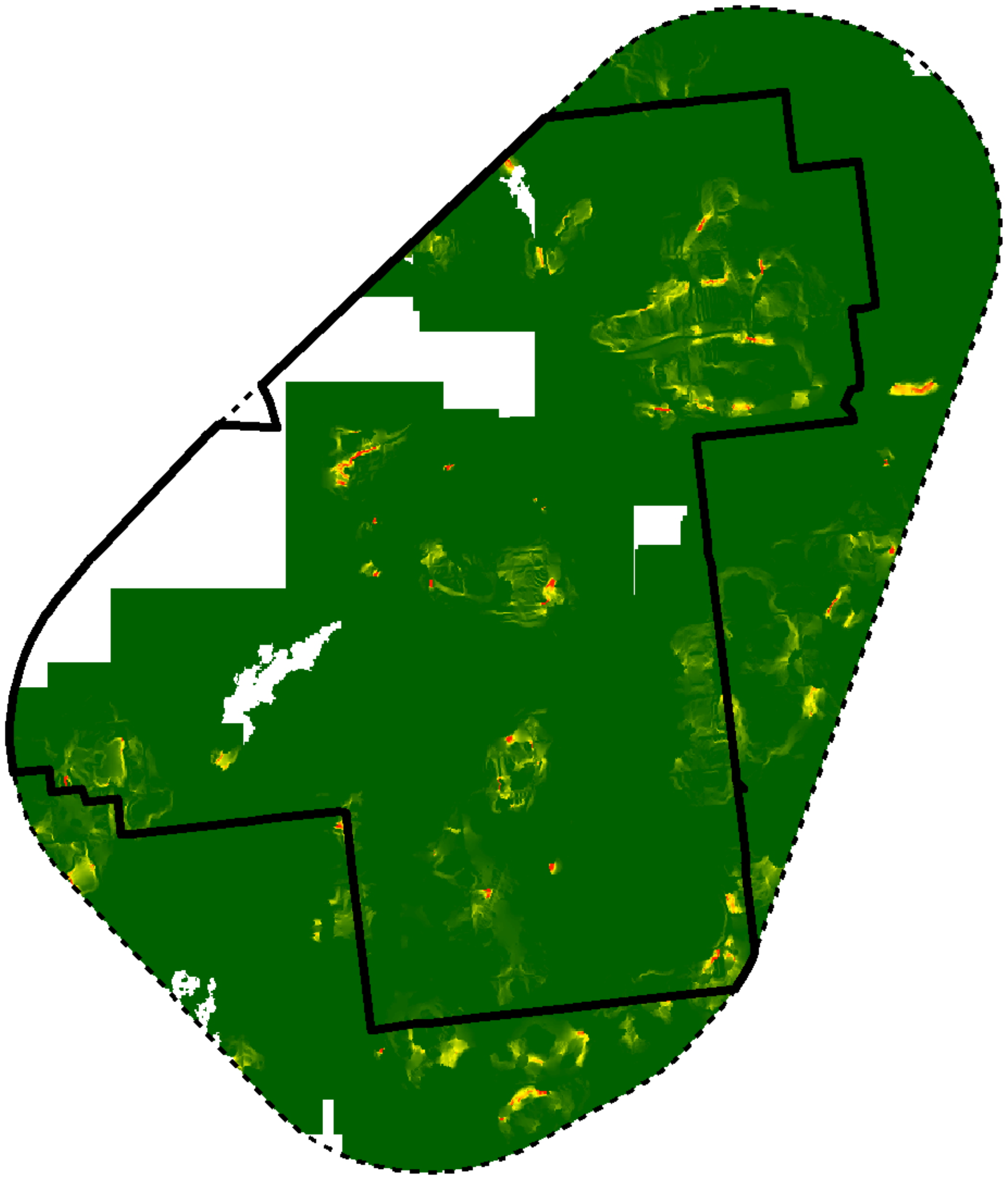

S32

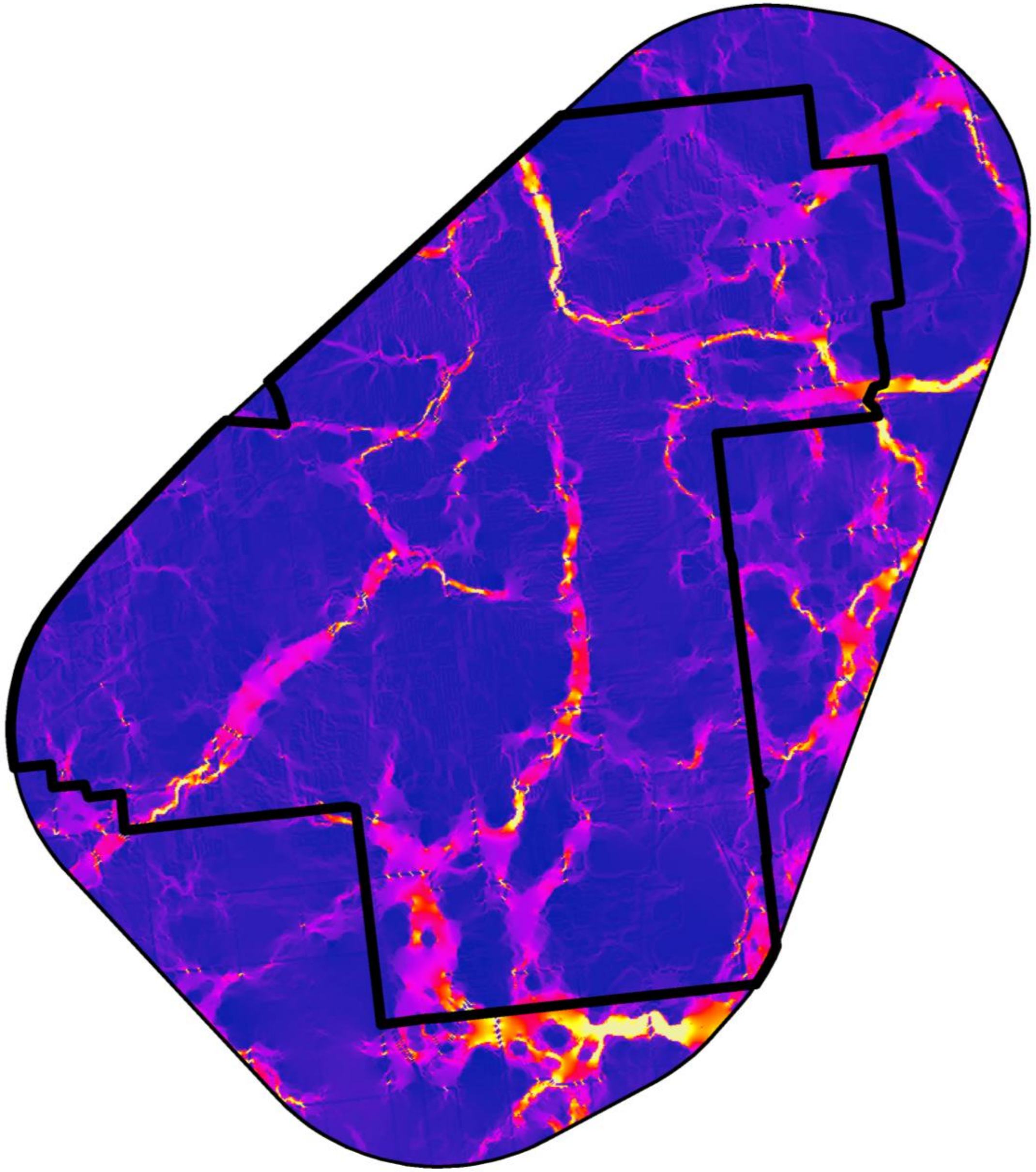

**Supplementary Figure 33**. Habitat network map for Black and Gold Bumble Bees (*Bombus auricomus*) in Lansing site showing habitat patch size as the size of the circle and importance of the patch in maintaining overall network connectivity with warming colors (orange and red) indicating highest importance.

**Supplementary Figure 34.** Map of locations where removal of a barrier (red areas) would improve connectivity for Black and Gold Bumble Bees (*Bombus auricomus*) in the Lansing site.

**Supplementary Figure 35.** Map of areas with narrow linkages (yellow and red areas) where Black and Gold Bumble Bees (*Bombus auricomus*) would have limited movement options in Lansing site making them important corridors to maintain.

**Supplementary Figure 36.** Current density map for Black and Gold Bumble Bees (*Bombus auricomus*) in Lansing site. Areas with higher current density are predicted to represent better movement corridors.

# S33

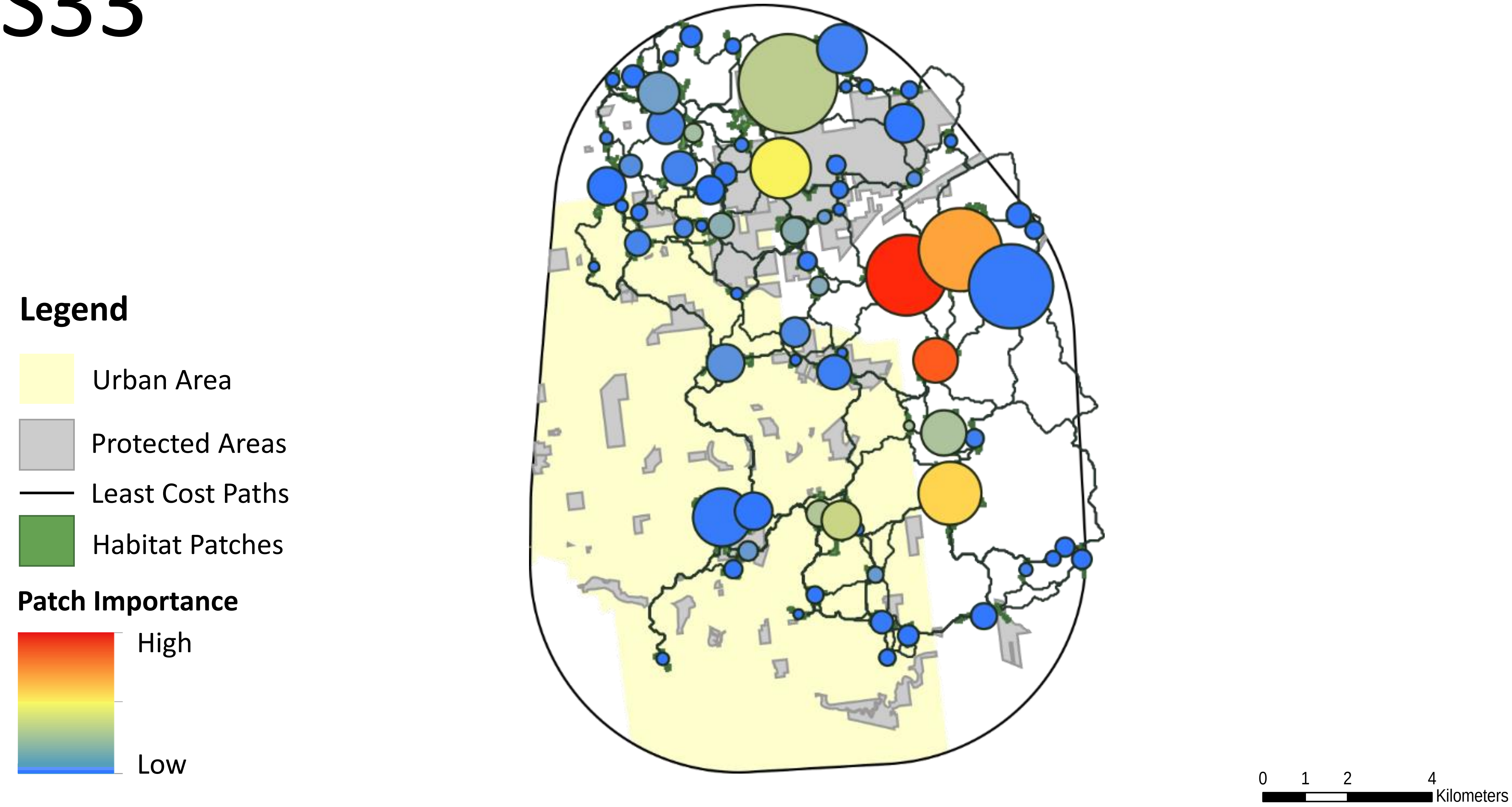

Legend
Urban Area
Protected Areas
Least Cost Paths
Habitat Patches
Patch Importance
High
Low
0
1
2
4
Kilometers

# S34

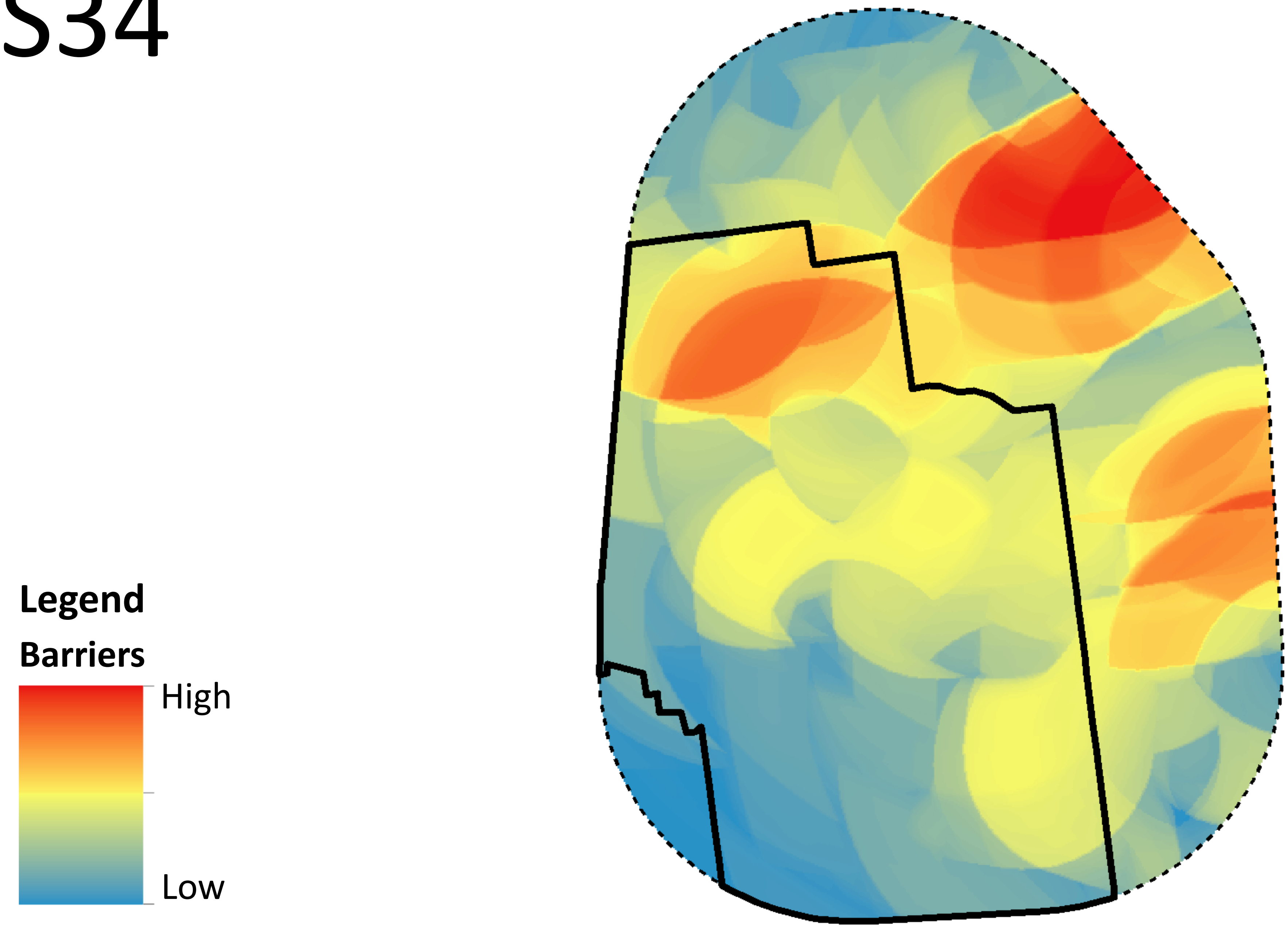

# S35

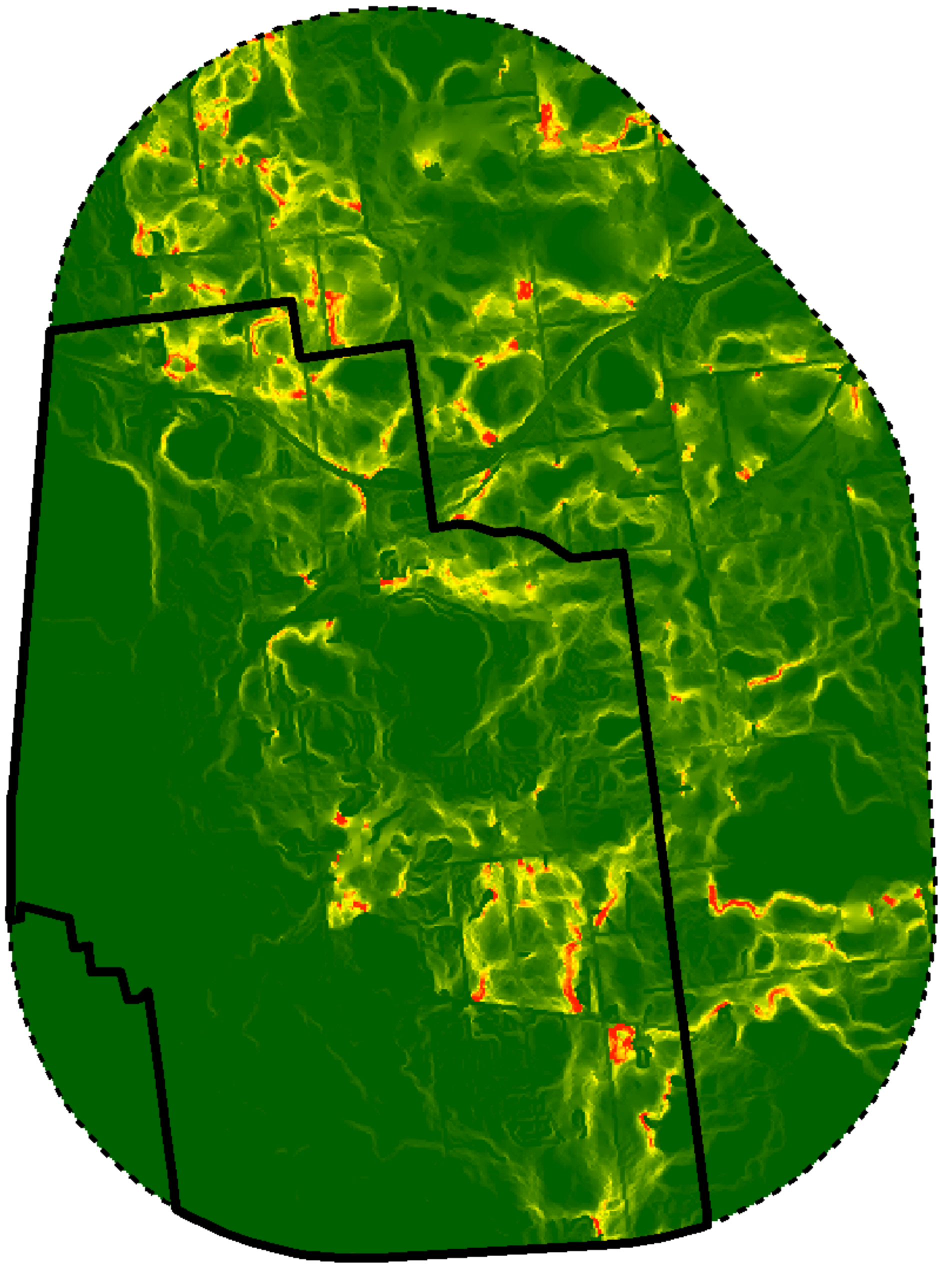

**Legend**

**Pinch points**

High

Low

# S36

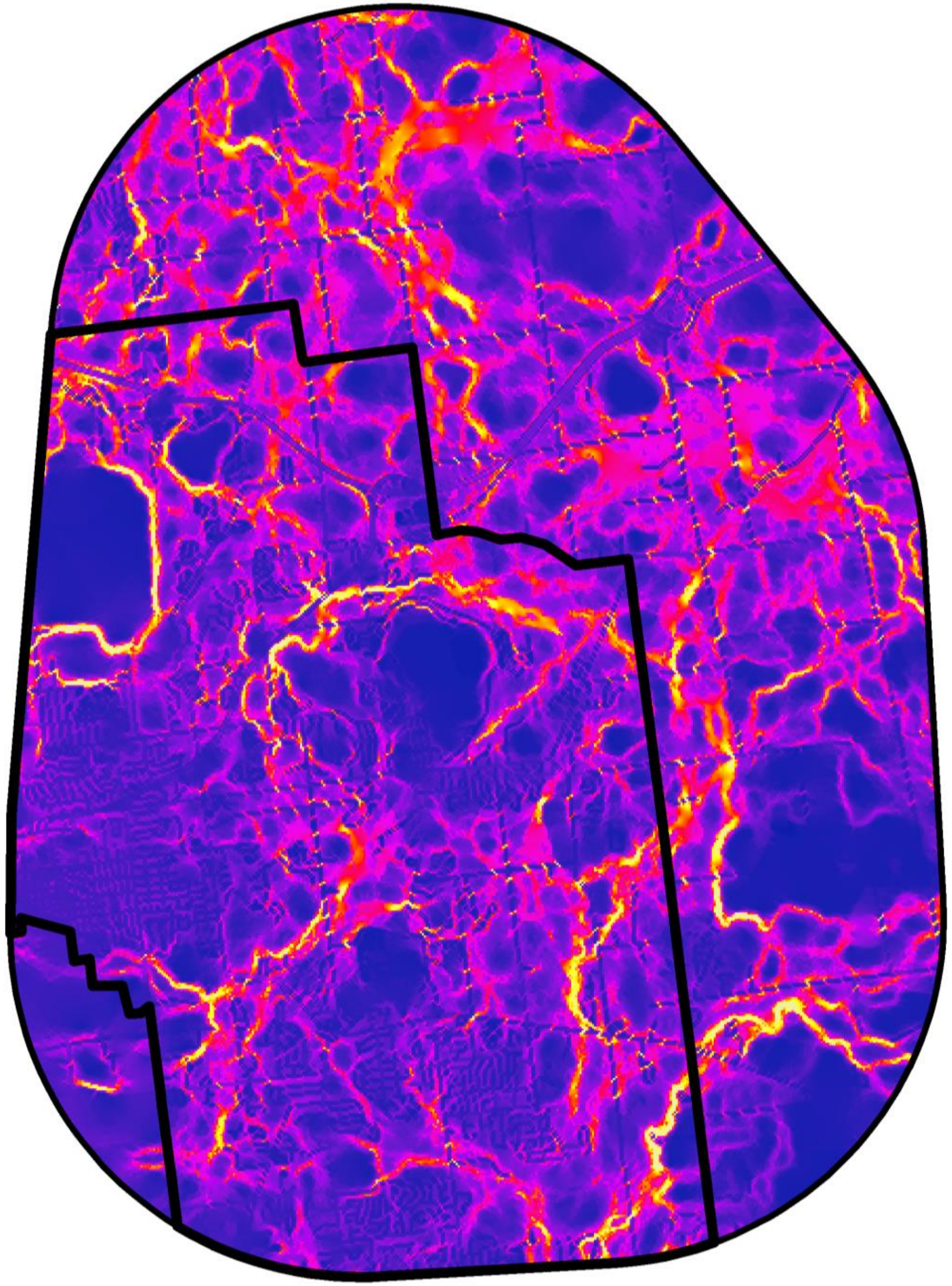

**Legend**

**Current density**

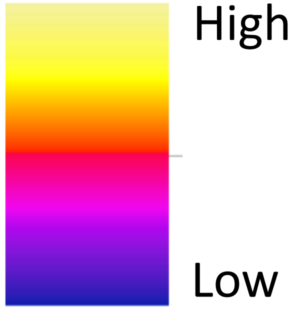

**Supplementary Figure 37**. Habitat network map for Blanding's Turtles (*Emydoidea blandingii*) in Lansing site showing habitat patch size as the size of the circle and importance of the patch in maintaining overall network connectivity with warming colors (orange and red) indicating highest importance.

**Supplementary Figure 38.** Map of locations where removal of a barrier (red areas) would improve connectivity for Blanding's Turtles (*Emydoidea blandingii*) in the Lansing site.

**Supplementary Figure 39.** Map of areas with narrow linkages (yellow and red areas) where Blanding's Turtles (*Emydoidea blandingii*) would have limited movement options in Lansing site making them important corridors to maintain.

**Supplementary Figure 40.** Current density map for Blanding's Turtles (*Emydoidea blandingii*) in Lansing site. Areas with higher current density are predicted to represent better movement corridors.

S37

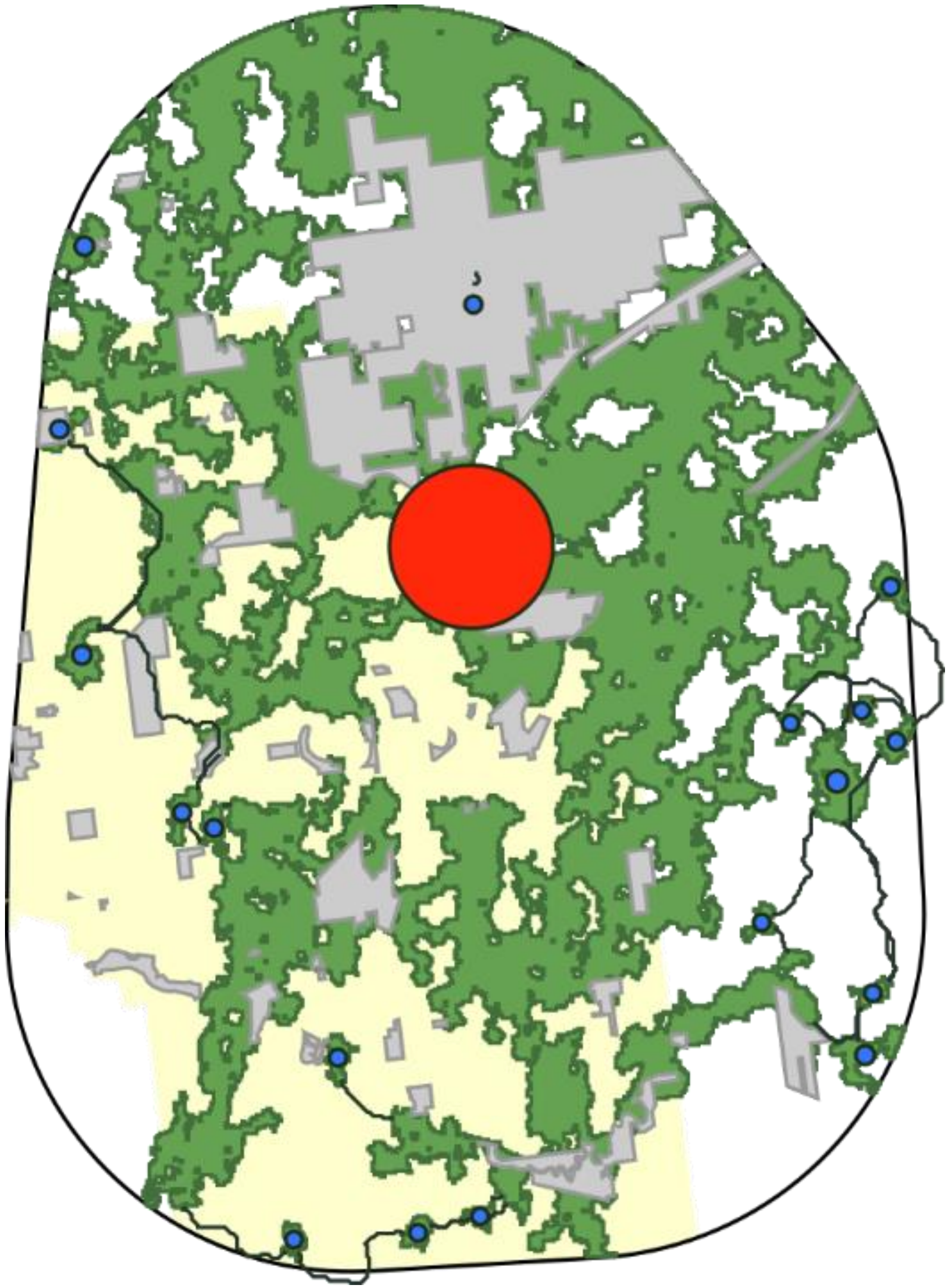

# S38

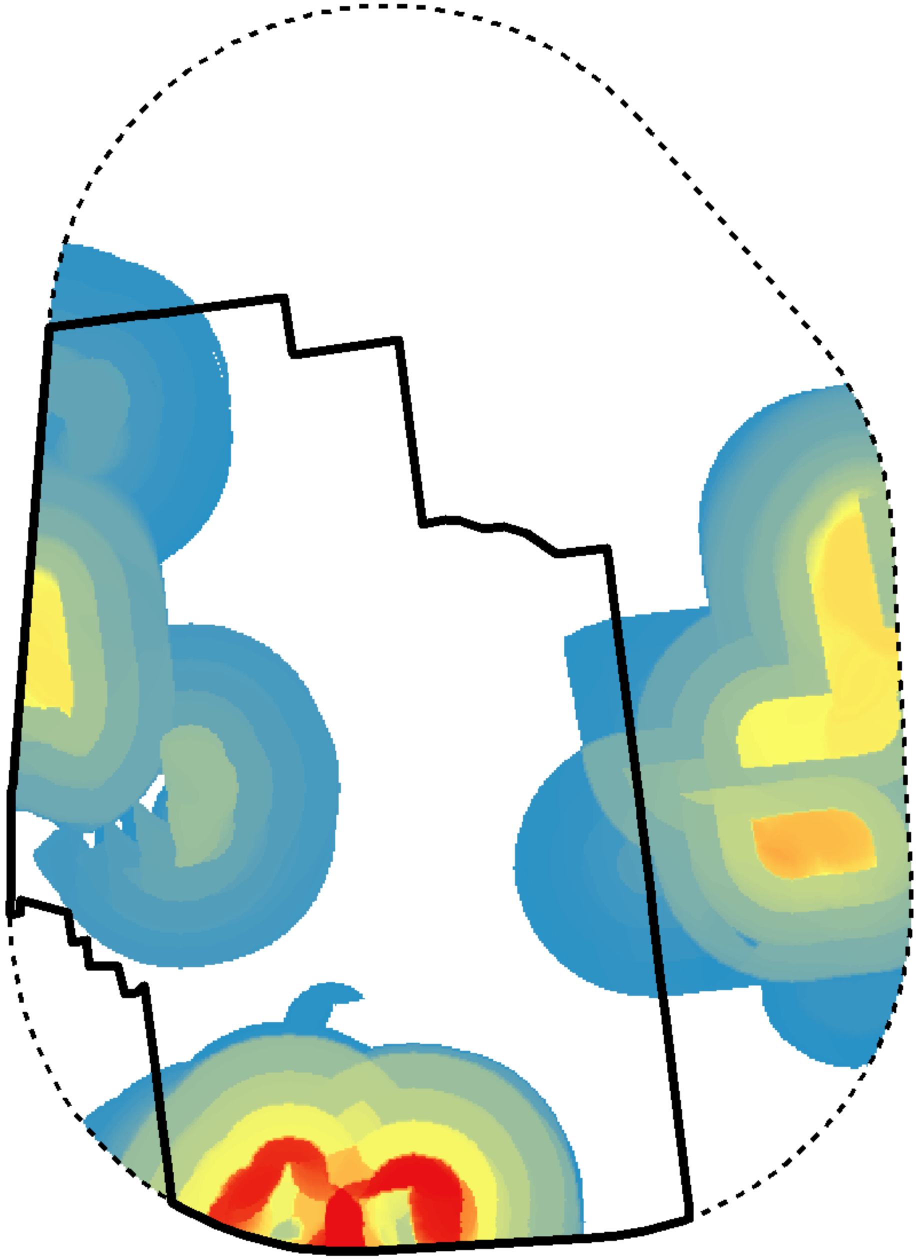

# S39

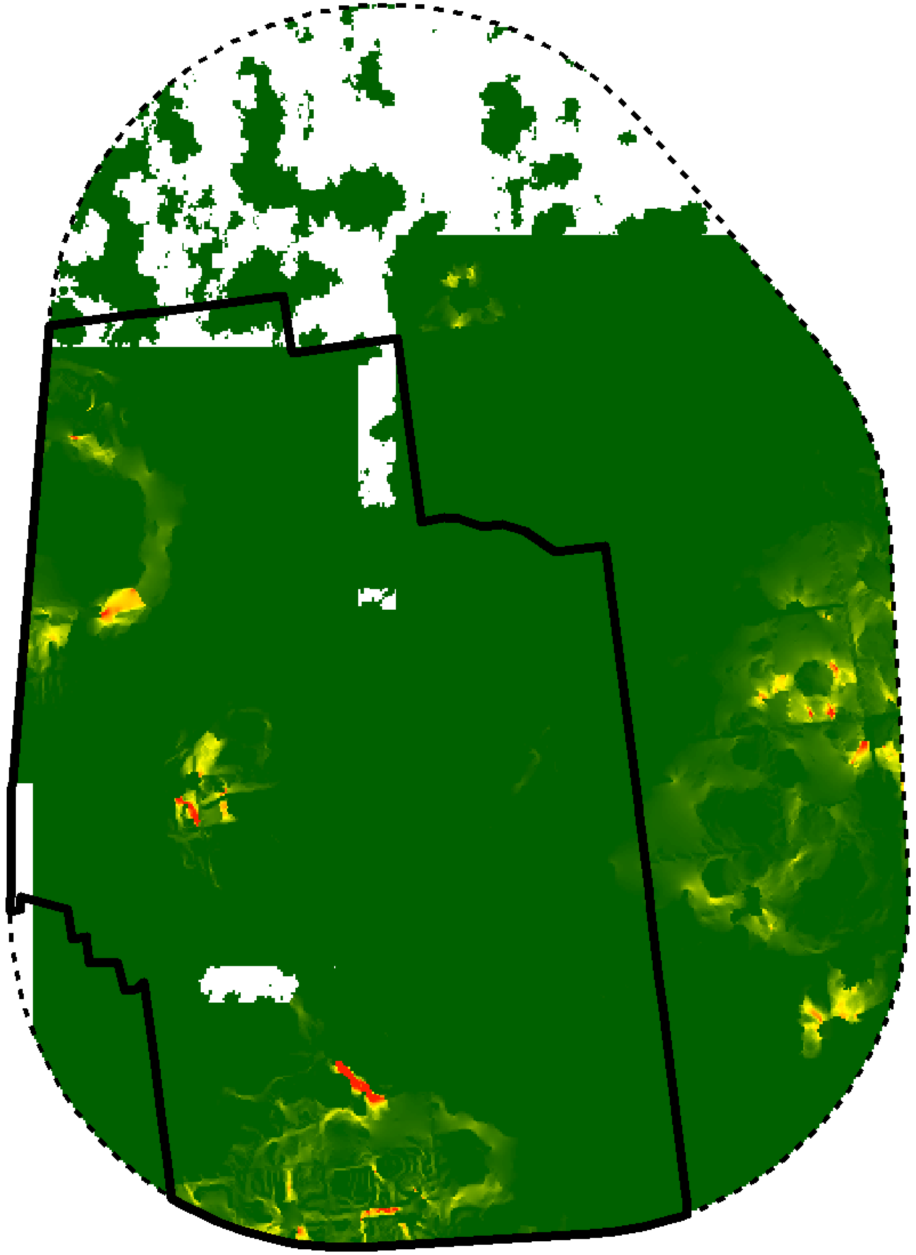

# S40

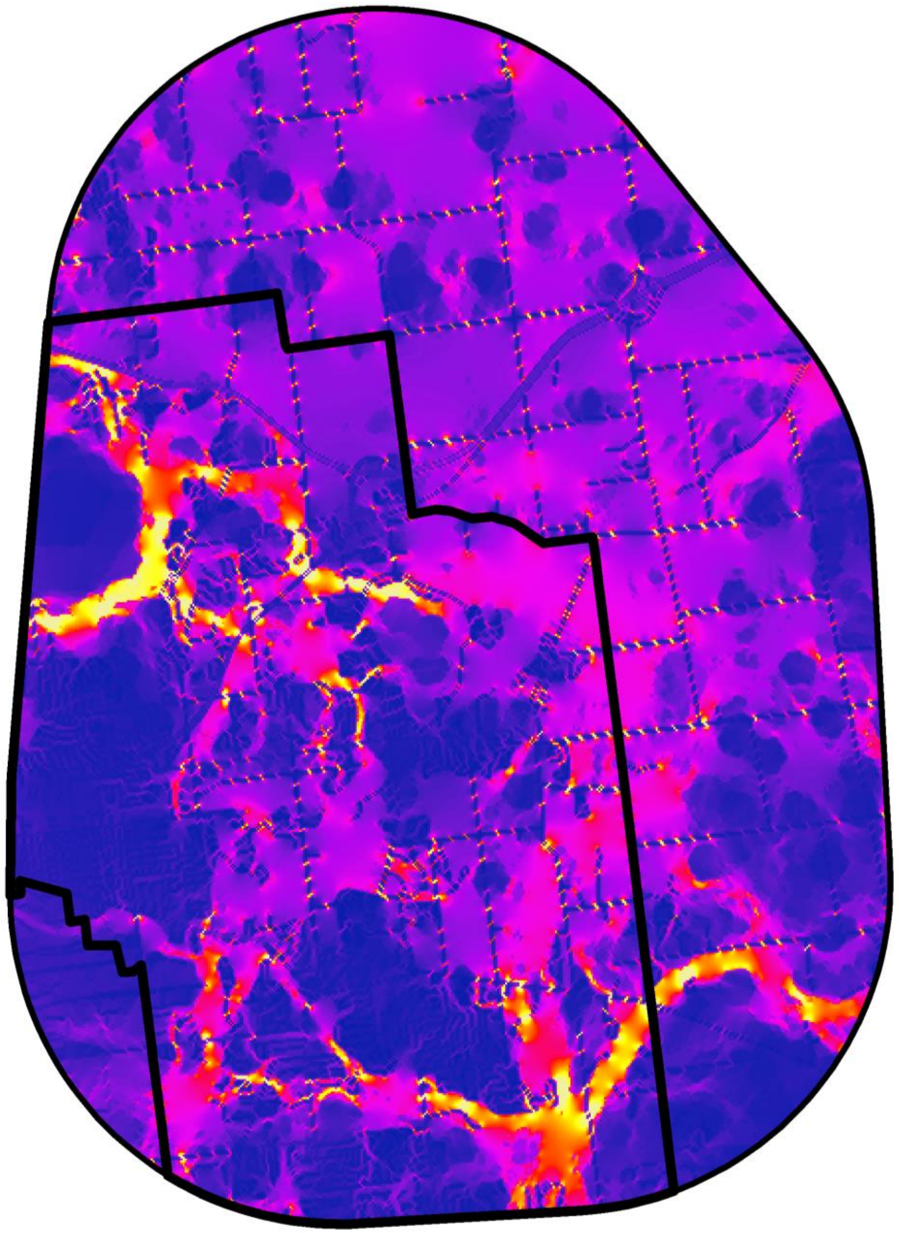

**Supplementary Figure 41**. Habitat network map for Eastern Massasauga Rattlesnakes (*Sistrurus catenatus*) in Lansing site showing habitat patch size as the size of the circle and importance of the patch in maintaining overall network connectivity with warming colors (orange and red) indicating highest importance.

**Supplementary Figure 42.** Map of locations where removal of a barrier (red areas) would improve connectivity for Eastern Massasauga Rattlesnakes (*Sistrurus catenatus*) in the Lansing site.

**Supplementary Figure 43.** Map of areas with narrow linkages (yellow and red areas) where Eastern Massasauga Rattlesnakes (*Sistrurus catenatus*) would have limited movement options in Lansing site making them important corridors to maintain.

**Supplementary Figure 44.** Current density map for Eastern Massasauga Rattlesnakes (*Sistrurus catenatus*) in Lansing site. Areas with higher current density are predicted to represent better movement corridors.

S41

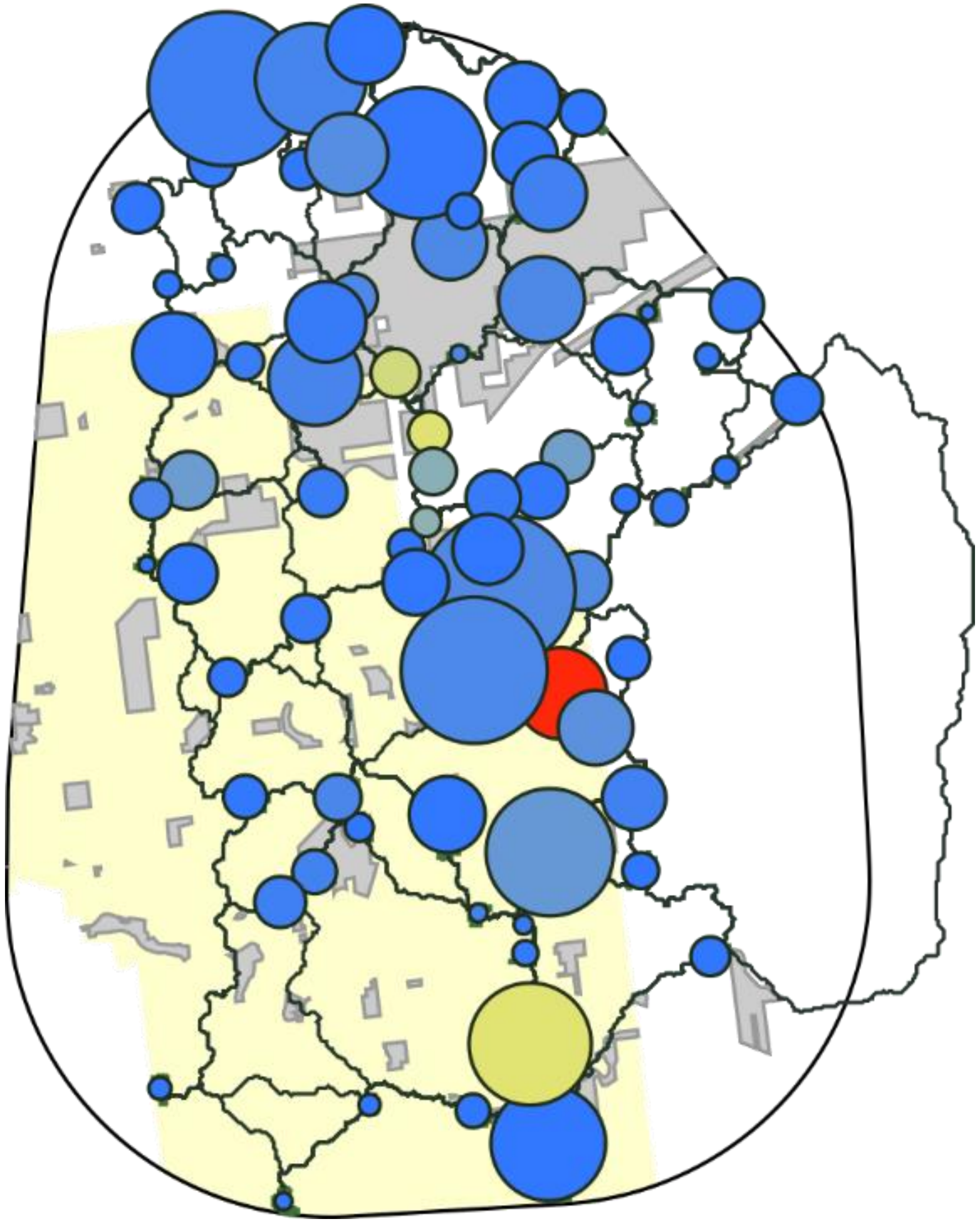

# S42

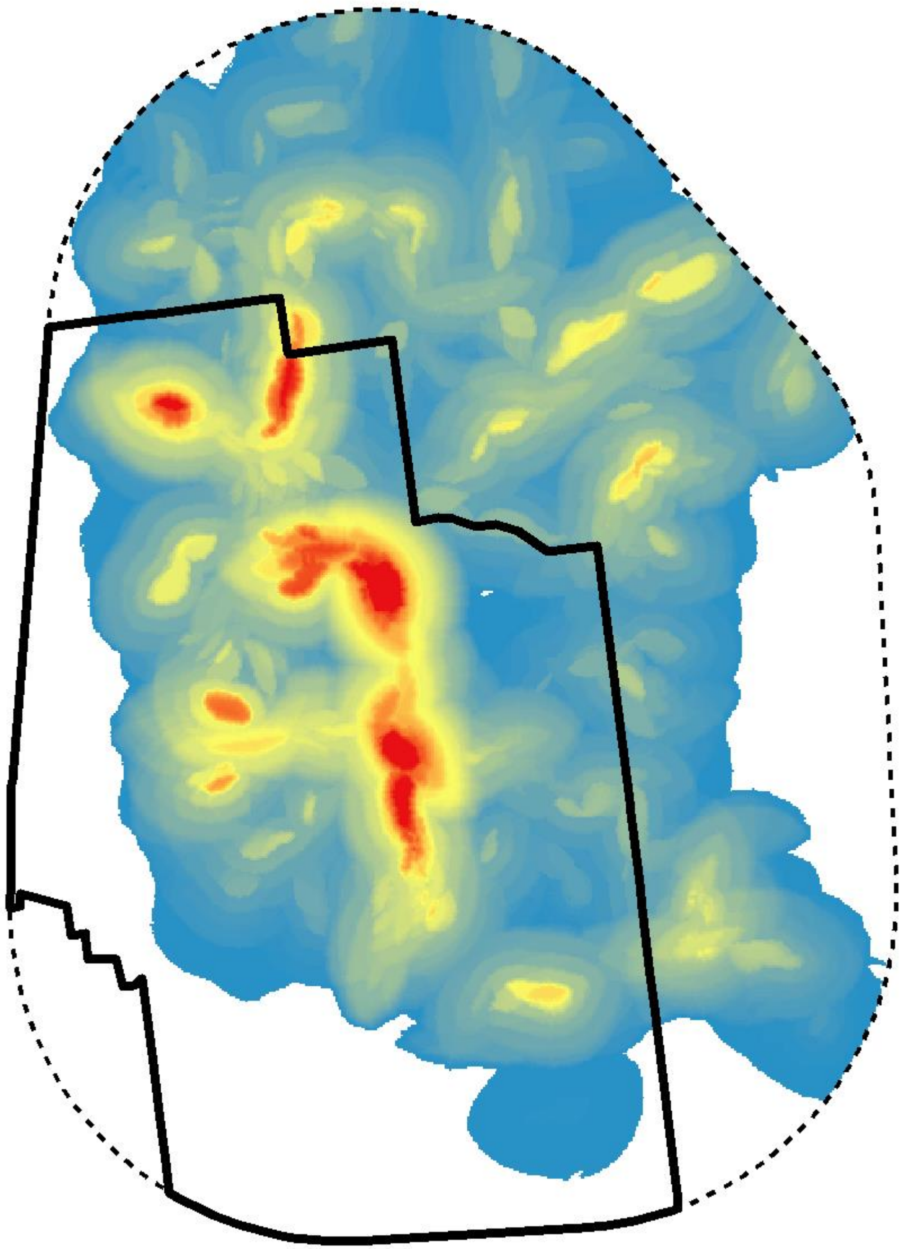

S43

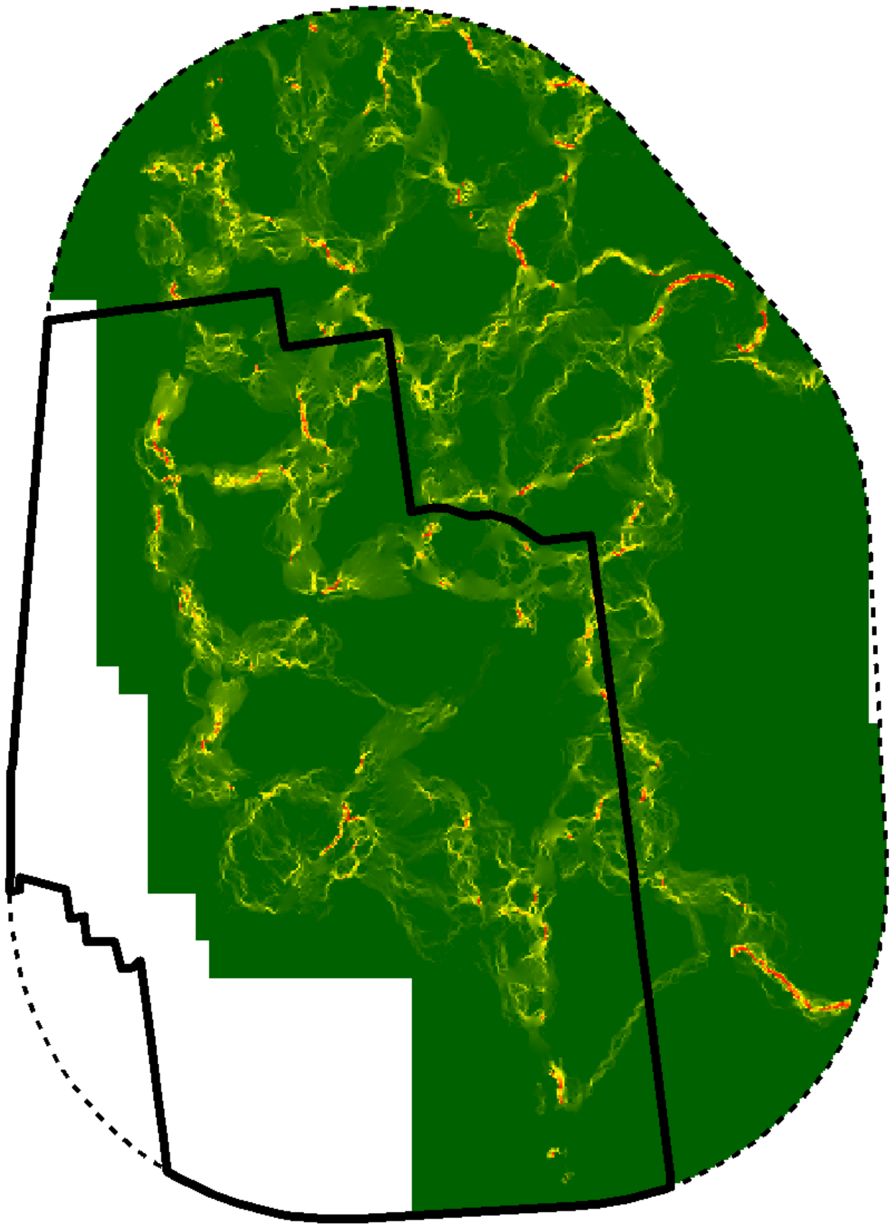

# S44

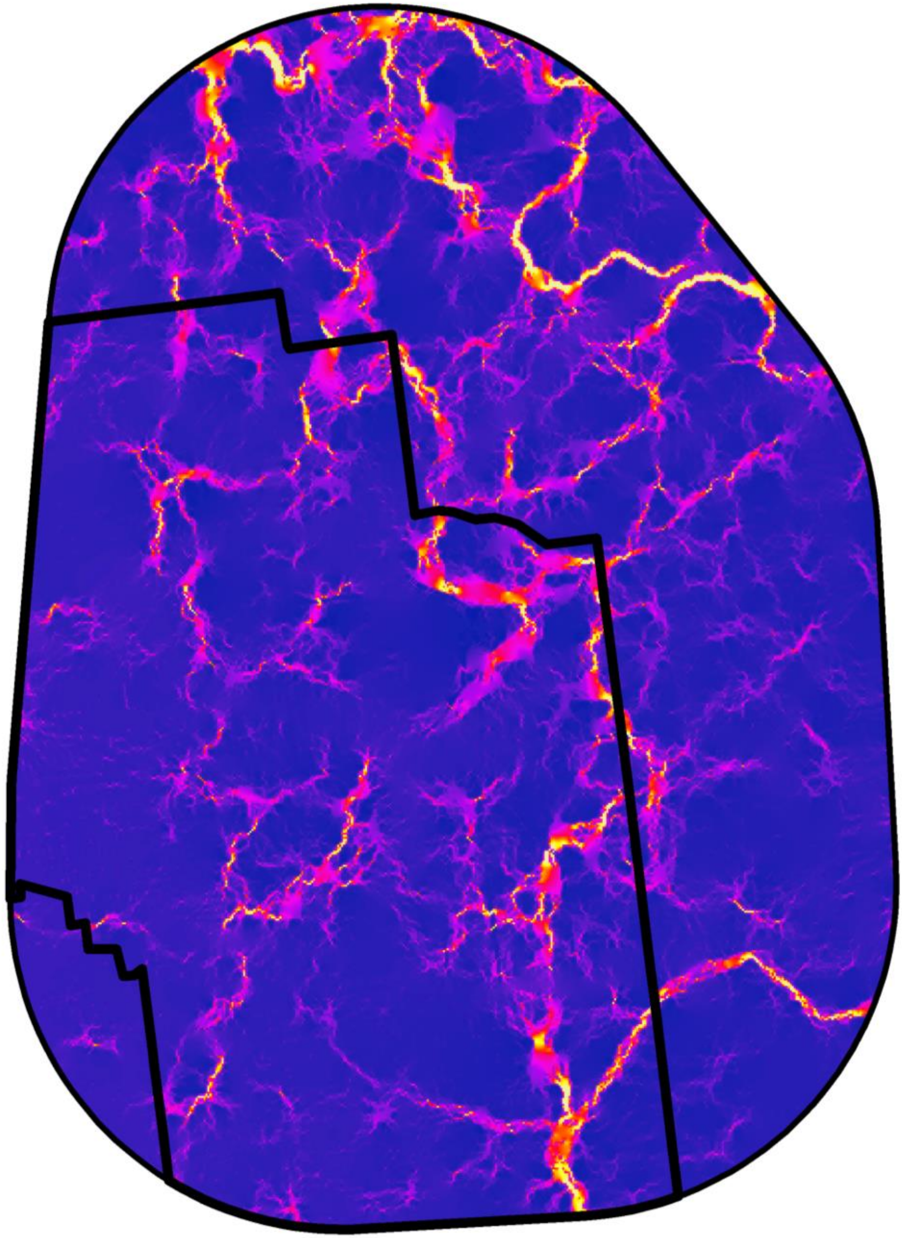

**Supplementary Figure 45**. Habitat network map for American Bumble Bees (*Bombus pensylvanicus*) in Detroit South site showing habitat patch size as the size of the circle and importance of the patch in maintaining overall network connectivity with warming colors (orange and red) indicating highest importance.

**Supplementary Figure 46.** Map of locations where removal of a barrier (red areas) would improve connectivity for American Bumble Bees (*Bombus pensylvanicus*) in the Detroit South site.

**Supplementary Figure 47.** Map of areas with narrow linkages (yellow and red areas) where American Bumble Bees (*Bombus pensylvanicus*) would have limited movement options in Detroit South site making them important corridors to maintain.

**Supplementary Figure 48.** Current density map for American Bumble Bees (*Bombus pensylvanicus*) in Detroit South site. Areas with higher current density are predicted to represent better movement corridors.

# S45

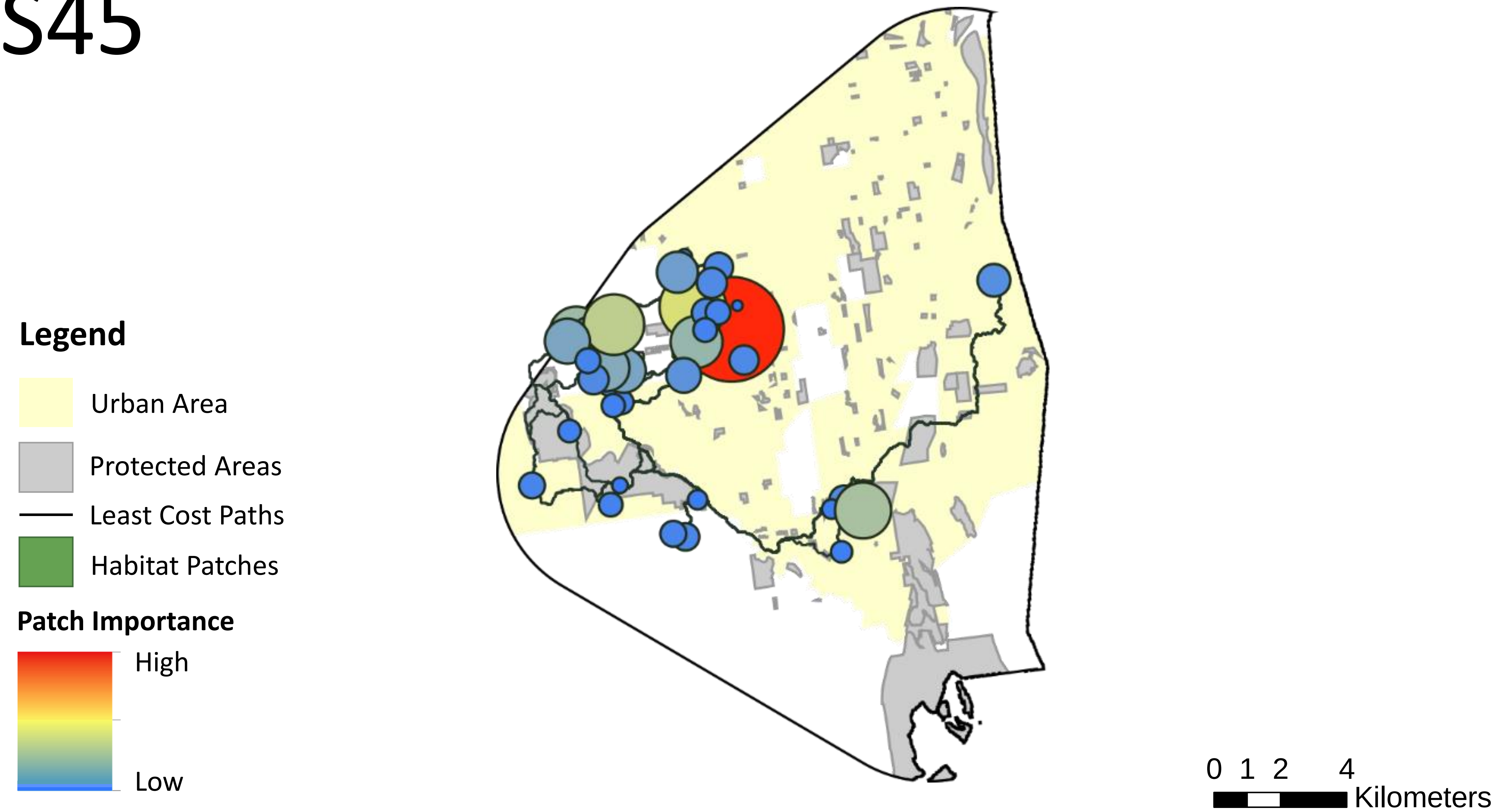
Legend
Urban Area
Protected Areas
Least Cost Paths
Habitat Patches
Patch Importance
High
Low
0 1 2 4
Kilometers

# S46

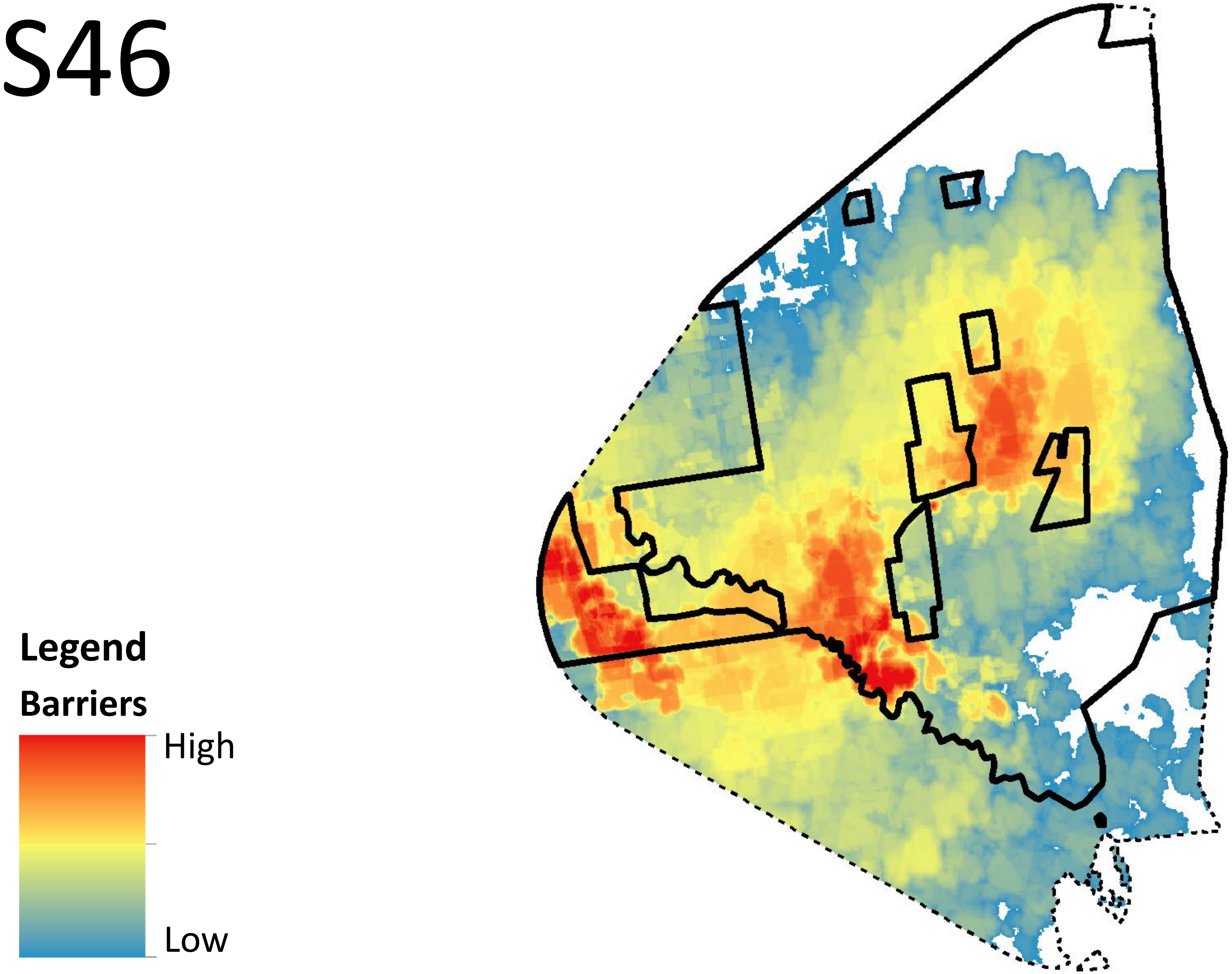

# S47

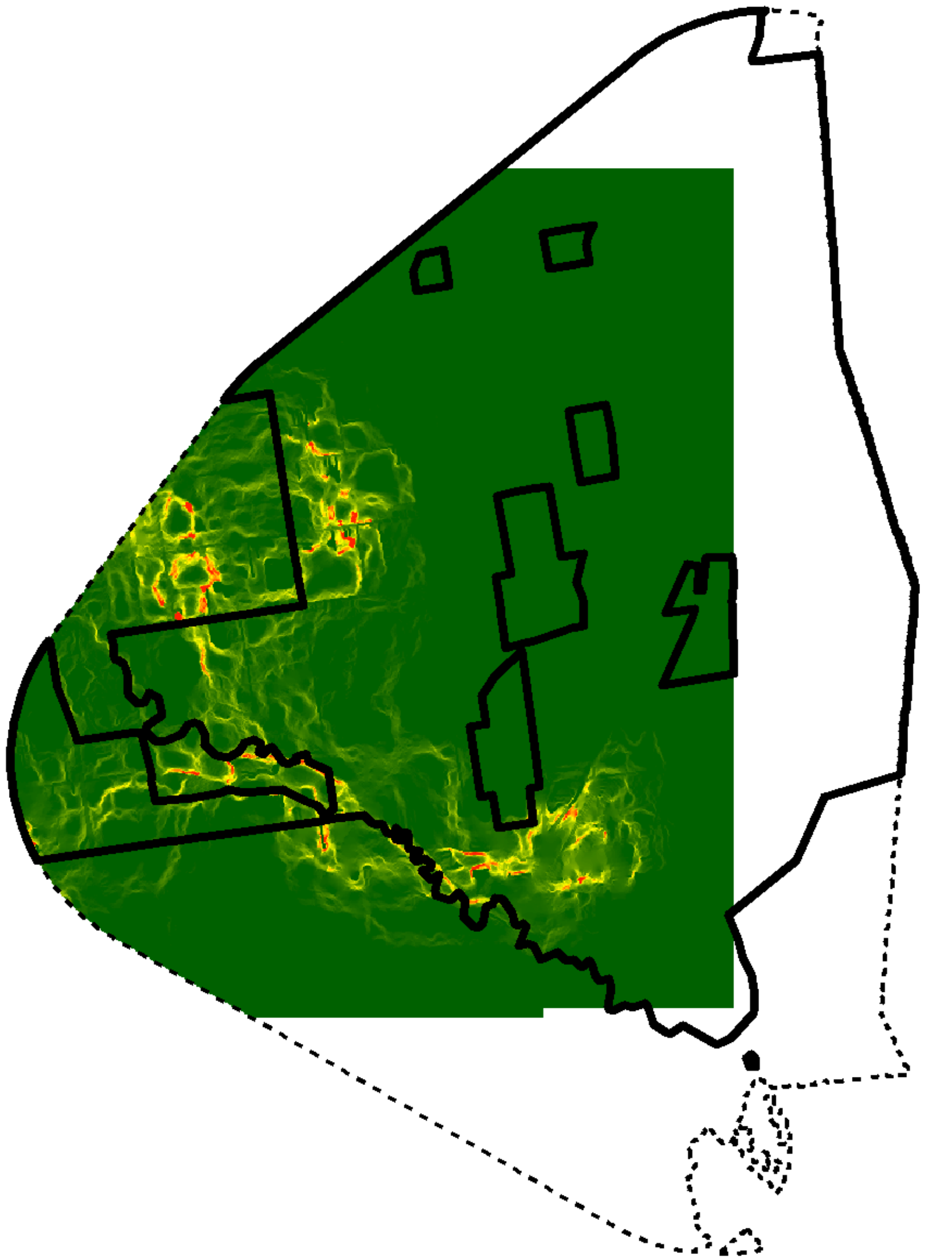

**Legend**

**Pinch points**

High

Low

# S48

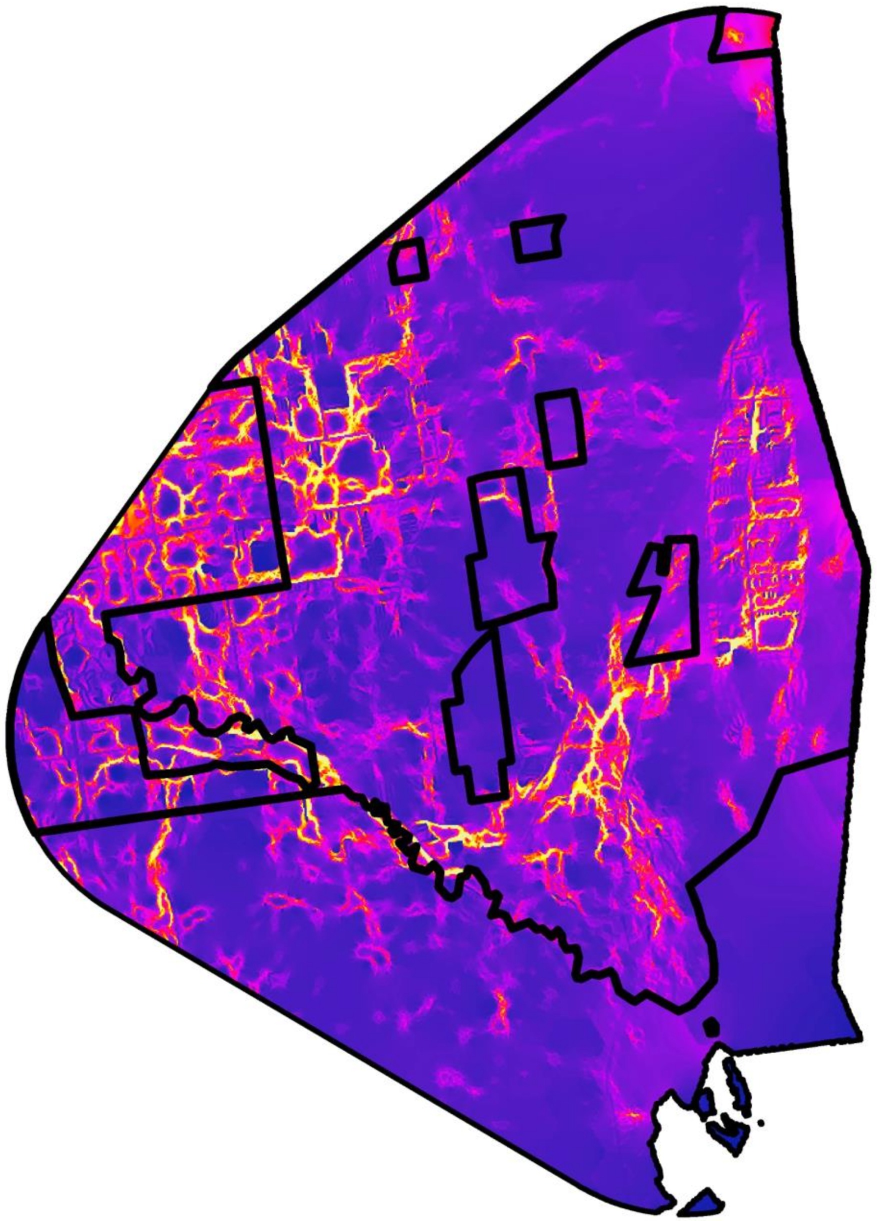

**Legend**

**Current density**

High

Low
