# Supplementary material for: Assessing habitat connectivity of rare species to inform urban conservation planning
Source: Ecol Evol. 2024 Mar 4;14(3):e11105. doi: 10.1002/ece3.11105 (PMC10912553; doi:10.1002/ece3.11105)

Supplementary Figure 49. Habitat network map for Eastern Foxsnakes (*Pantherophis gloydi*) in Detroit South site showing habitat patch size as the size of the circle and importance of the patch in maintaining overall network connectivity with warming colors (orange and red) indicating highest importance.

Supplementary Figure 50. Map of locations where removal of a barrier (red areas) would improve connectivity for Eastern Foxsnakes (*Pantherophis gloydi*) in the Detroit South site.

Supplementary Figure 51. Map of areas with narrow linkages (yellow and red areas) where Eastern Foxsnakes (*Pantherophis gloydi*) would have limited movement options in Detroit South site making them important corridors to maintain.

Supplementary Figure 52. Current density map for Eastern Foxsnakes (*Pantherophis gloydi*) in Detroit South site. Areas with higher current density are predicted to represent better movement corridors.

S49

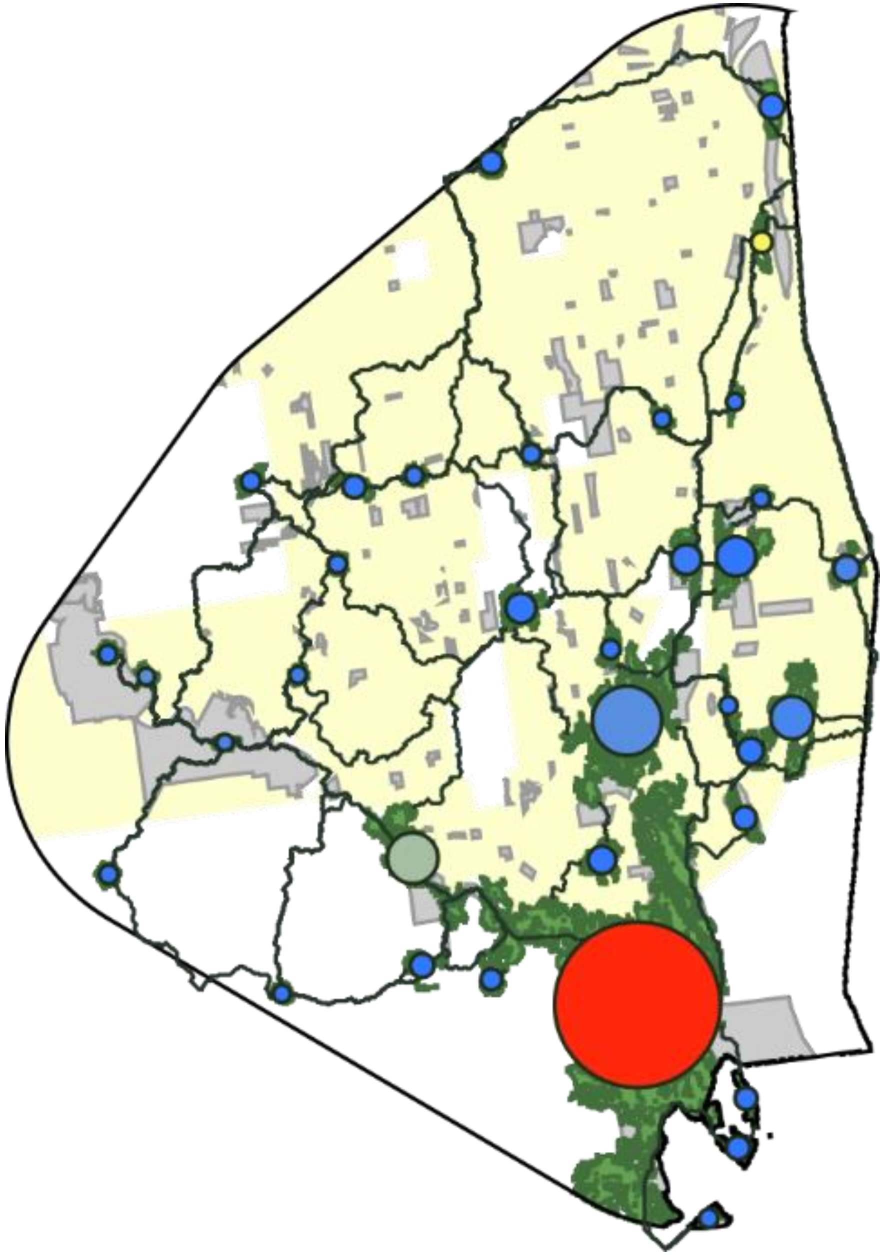

S50

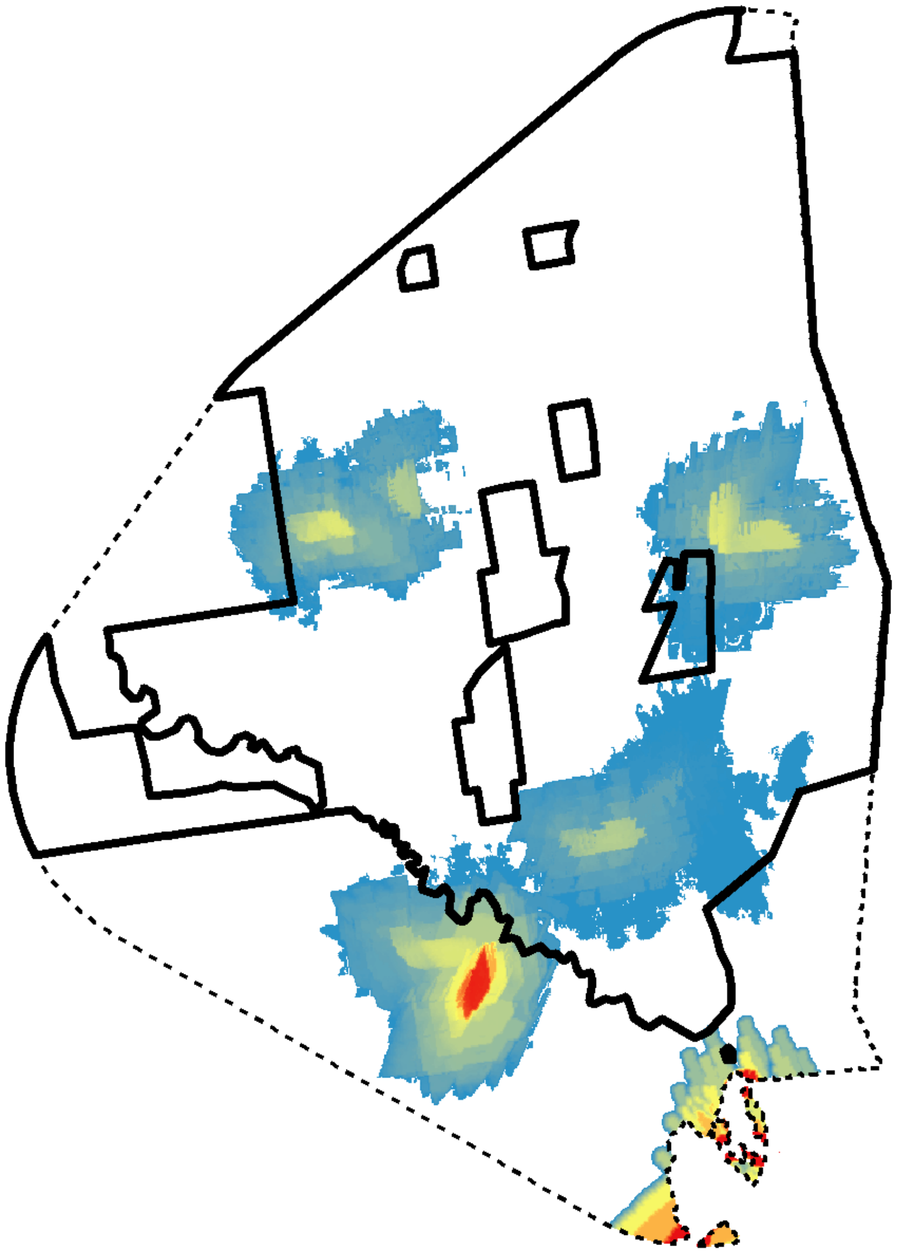

S51

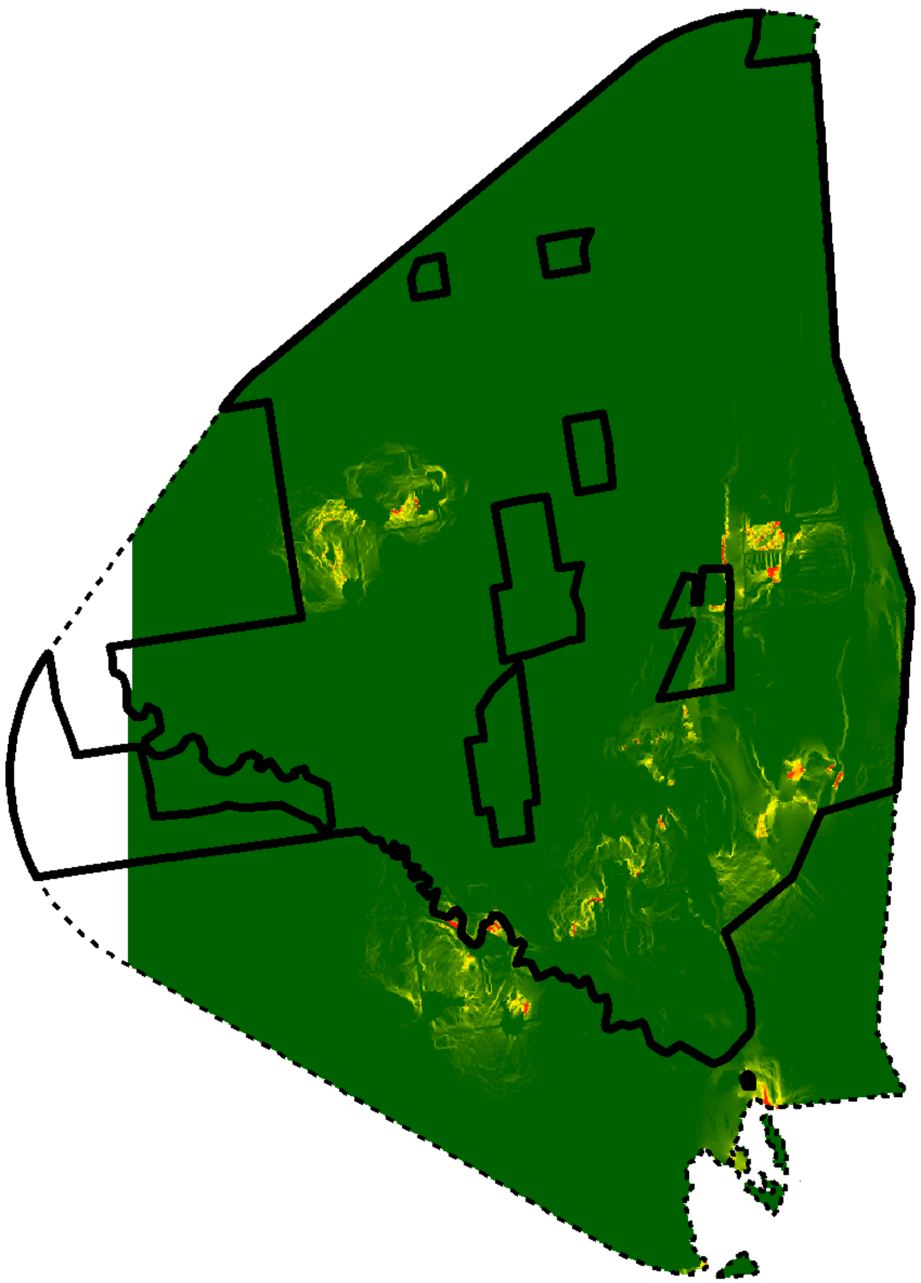

S52

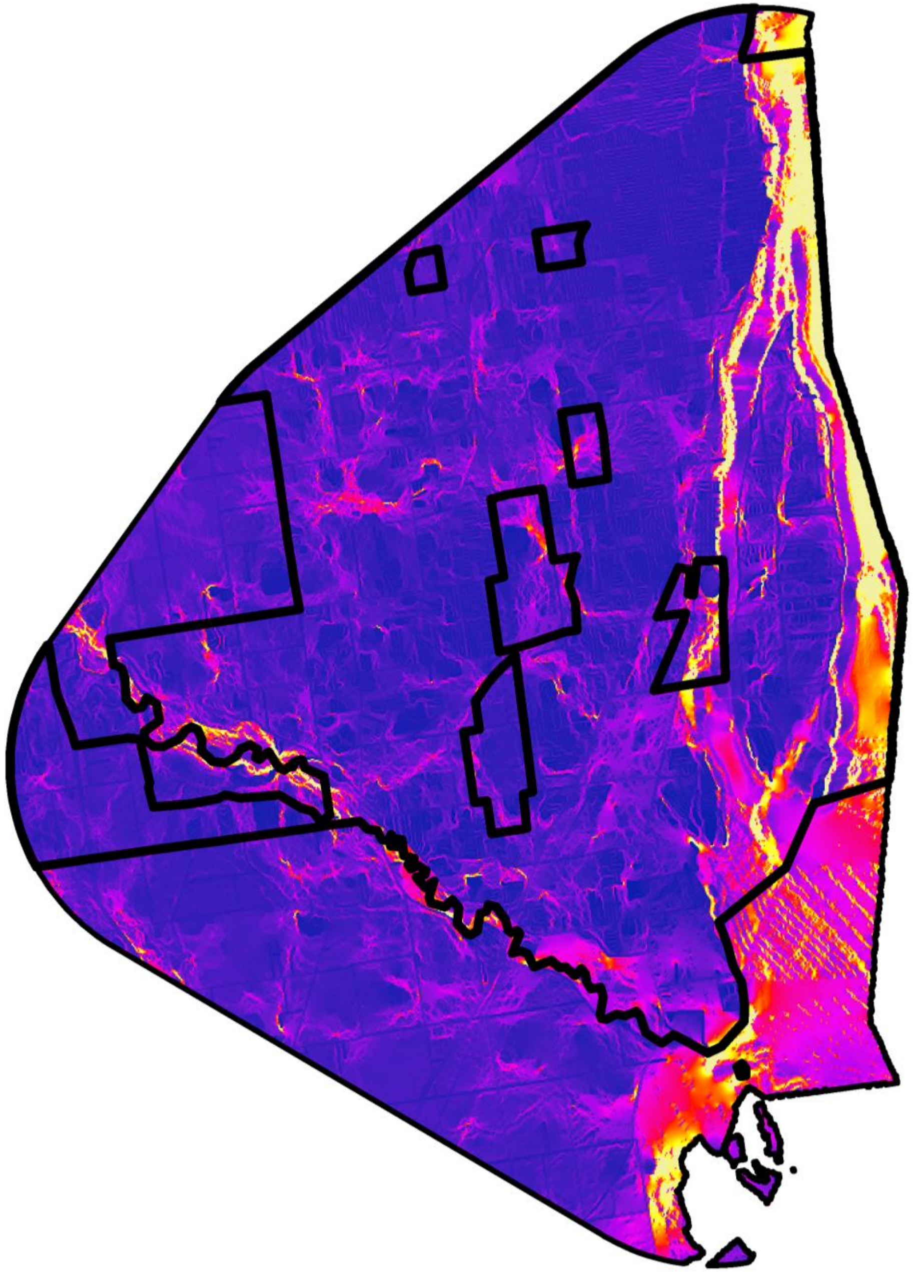

Supplementary Figure 53. Habitat network map for Black and Gold Bumble Bees (*Bombus auricomus*) in Detroit Southwest site showing habitat patch size as the size of the circle and importance of the patch in maintaining overall network connectivity with warming colors (orange and red) indicating highest importance.

Supplementary Figure 54. Map of locations where removal of a barrier (red areas) would improve connectivity for Black and Gold Bumble Bees (*Bombus auricomus*) in the Detroit Southwest site.

Supplementary Figure 55. Map of areas with narrow linkages (yellow and red areas) where Black and Gold Bumble Bees (*Bombus auricomus*) would have limited movement options in Detroit Southwest site making them important corridors to maintain.

Supplementary Figure 56. Current density map for Black and Gold Bumble Bees (*Bombus auricomus*) in Detroit Southwest site. Areas with higher current density are predicted to represent better movement corridors.

S53

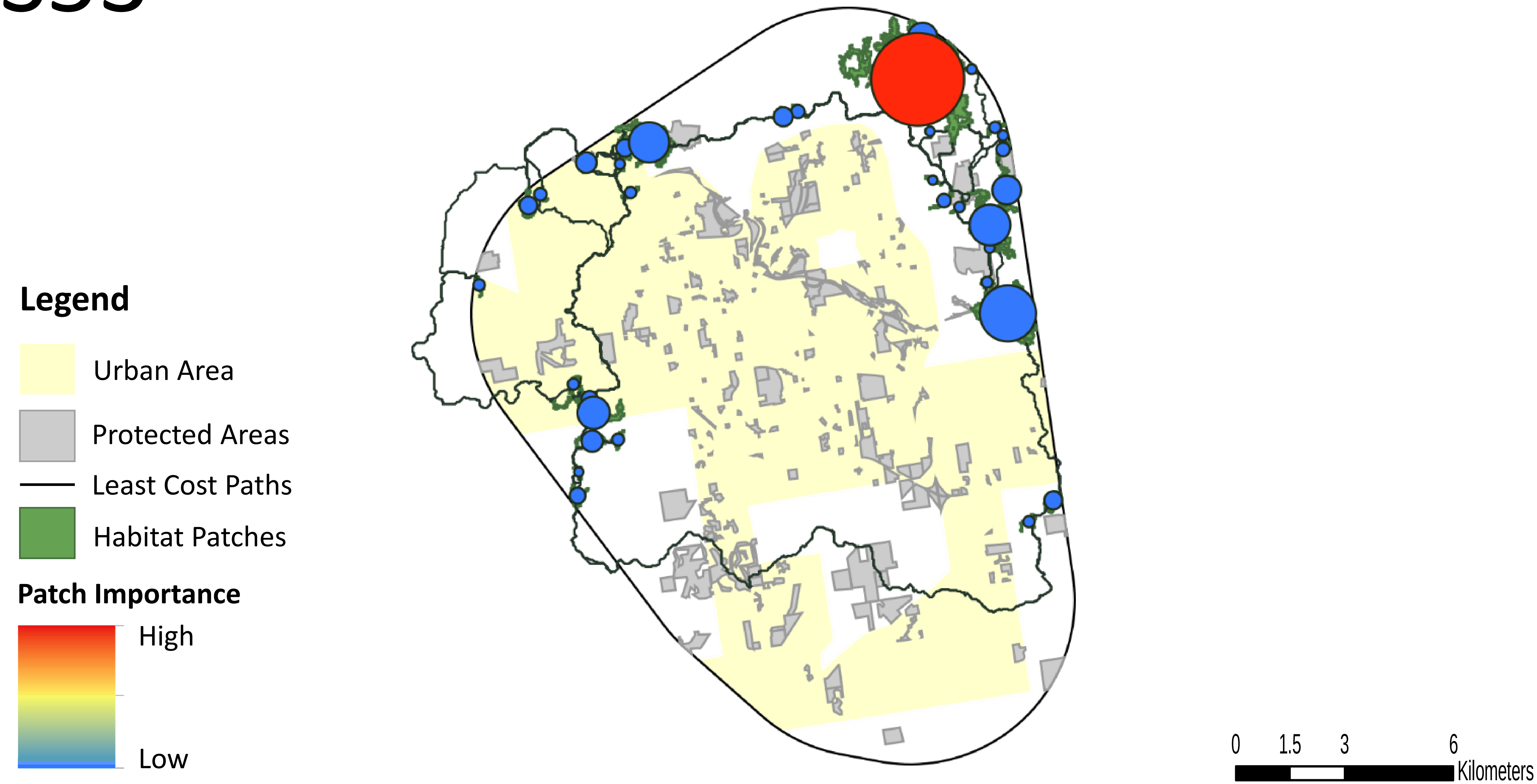
Legend
Urban Area
Protected Areas
Least Cost Paths
Habitat Patches
Patch Importance
High
Low
0
1.5
3
6
Kilometers

S54

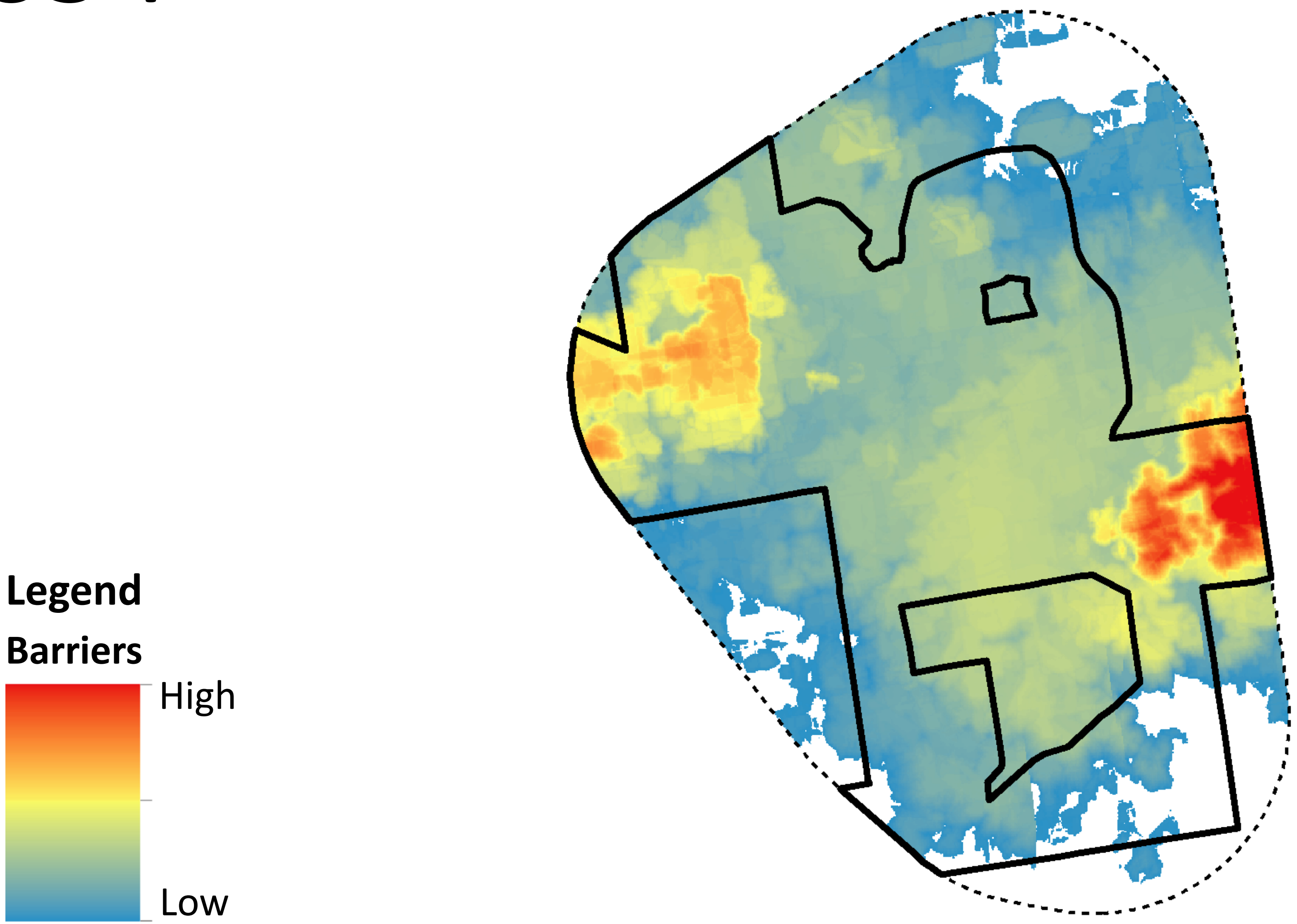

S55

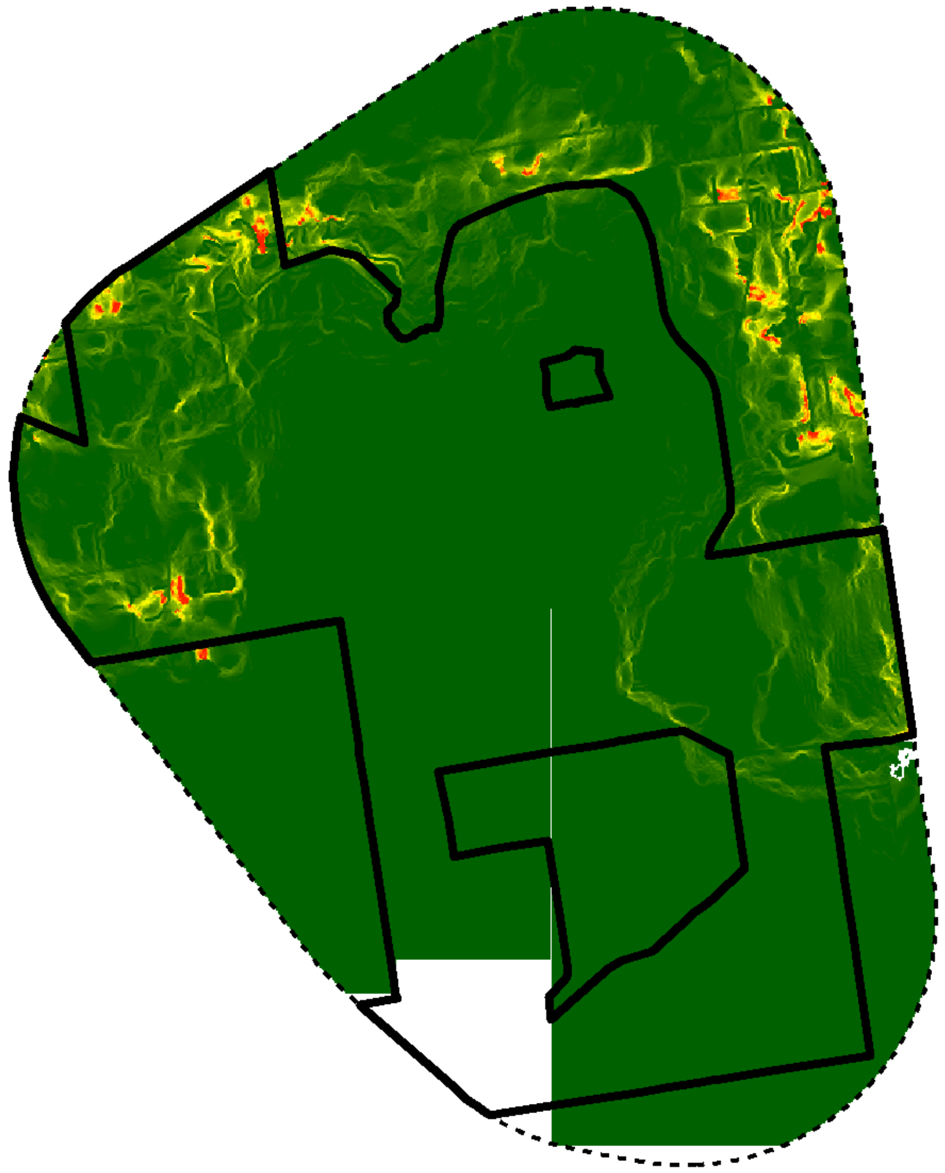

Legend

Pinch points

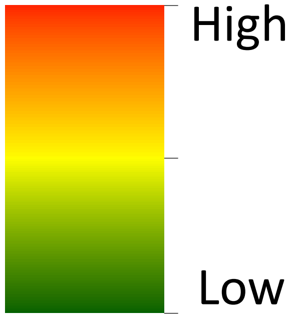

S56

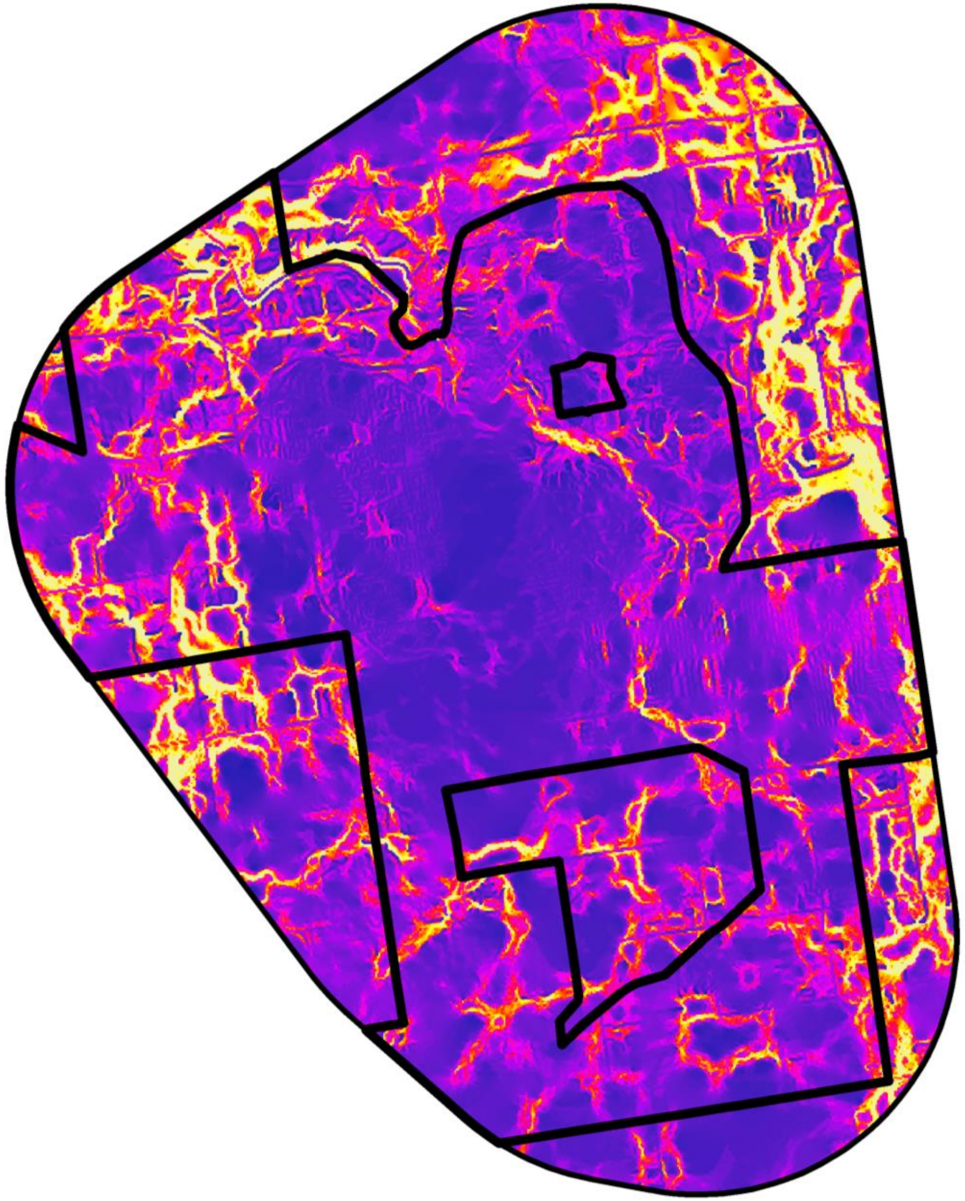

Legend

Current density

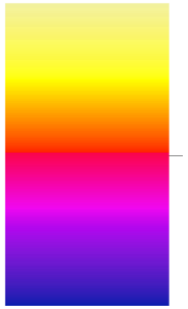

Supplementary Figure 57. Habitat network map for Blanding's Turtles (*Emydoidea blandingii*) in Detroit Southwest site showing habitat patch size as the size of the circle and importance of the patch in maintaining overall network connectivity with warming colors (orange and red) indicating highest importance.

Supplementary Figure 58. Map of locations where removal of a barrier (red areas) would improve connectivity for Blanding's Turtles (*Emydoidea blandingii*) in the Detroit Southwest site.

Supplementary Figure 59. Map of areas with narrow linkages (yellow and red areas) where Blanding's Turtles (*Emydoidea blandingii*) would have limited movement options in Detroit Southwest site making them important corridors to maintain.

Supplementary Figure 60. Current density map for Blanding's Turtles (*Emydoidea blandingii*) in Detroit Southwest site. Areas with higher current density are predicted to represent better movement corridors.

S57

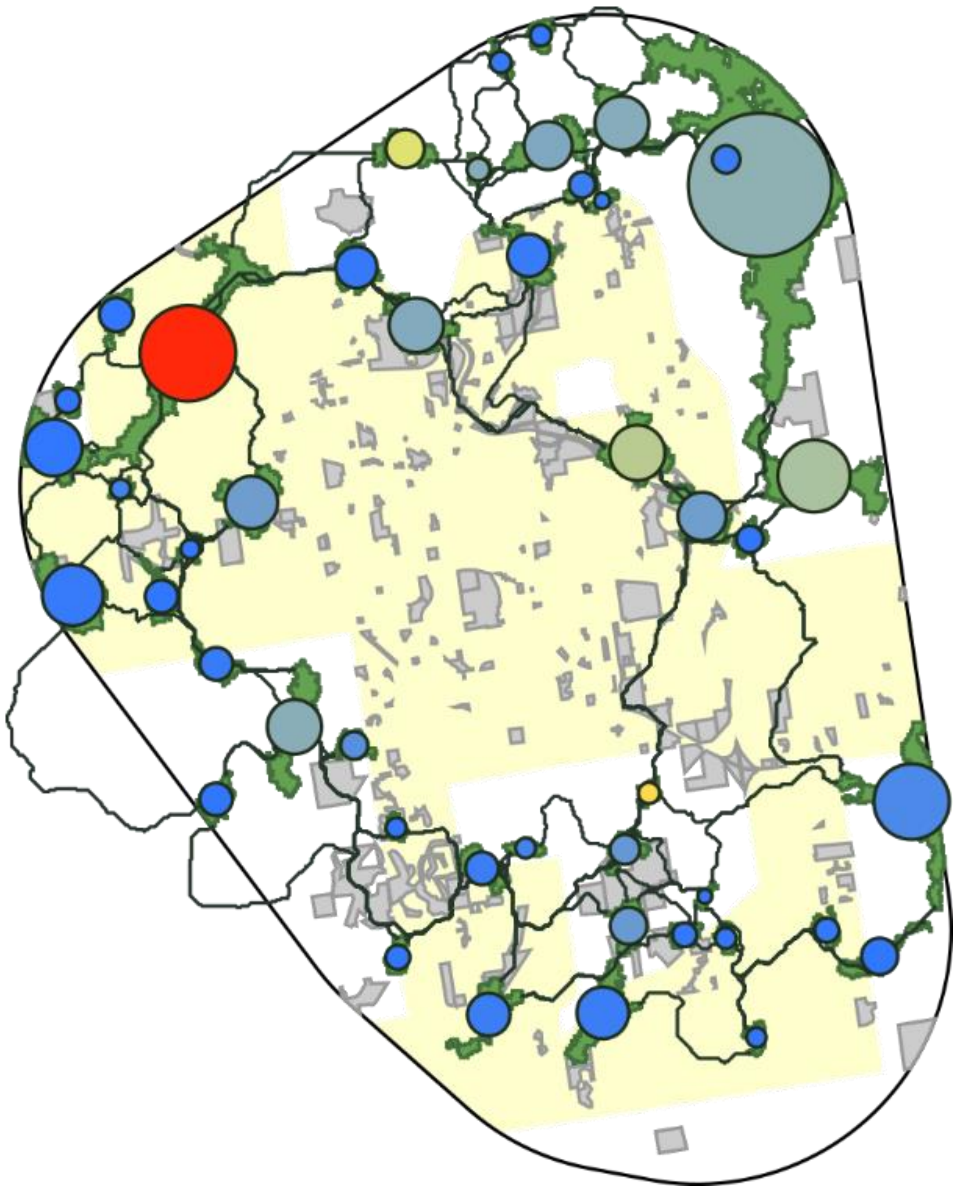

S58

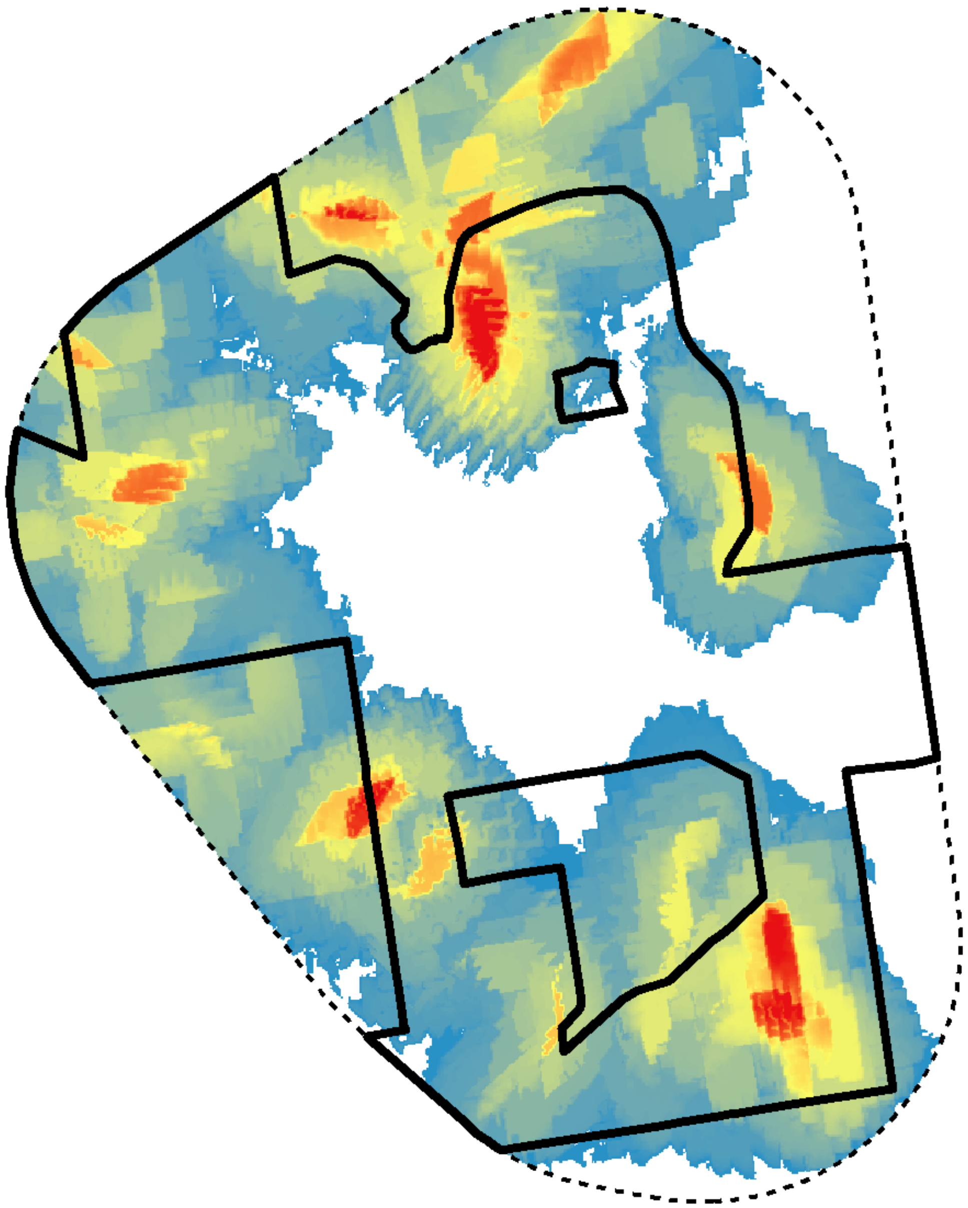

S59

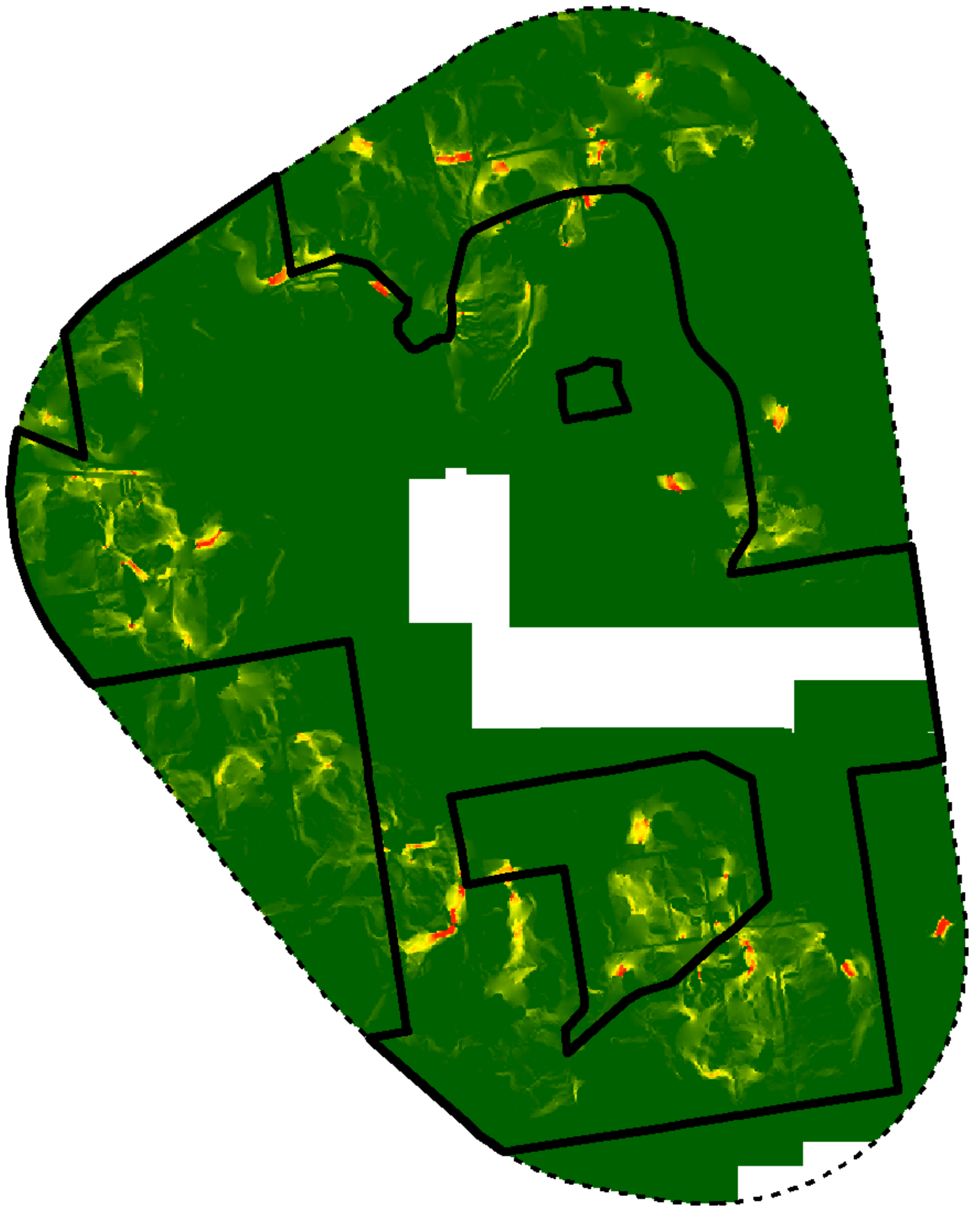

S60

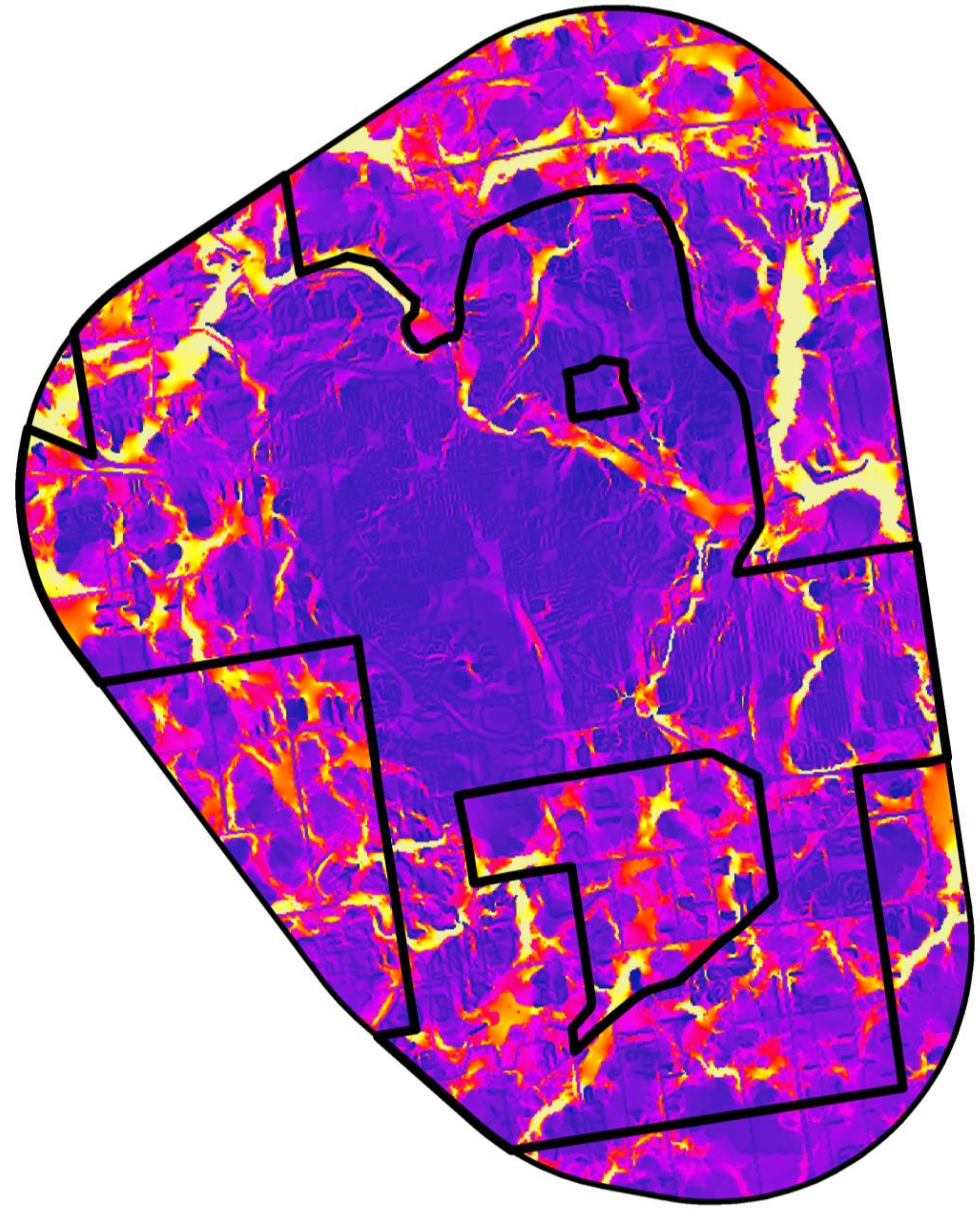

Supplementary Figure 61. Habitat network map for Henslow’s sparrows (*Ammodramus henslowii*) in Detroit Southwest site showing habitat patch size as the size of the circle and importance of the patch in maintaining overall network connectivity with warming colors (orange and red) indicating highest importance.

Supplementary Figure 62. Map of locations where removal of a barrier (red areas) would improve connectivity for Henslow’s sparrows (*Ammodramus henslowii*) in the Detroit Southwest site.

Supplementary Figure 63. Map of areas with narrow linkages (yellow and red areas) where Henslow’s sparrows (*Ammodramus henslowii*) would have limited movement options in Detroit Southwest site making them important corridors to maintain.

Supplementary Figure 64. Current density map for Henslow’s sparrows (*Ammodramus henslowii*) in Detroit Southwest site. Areas with higher current density are predicted to represent better movement corridors.

S61

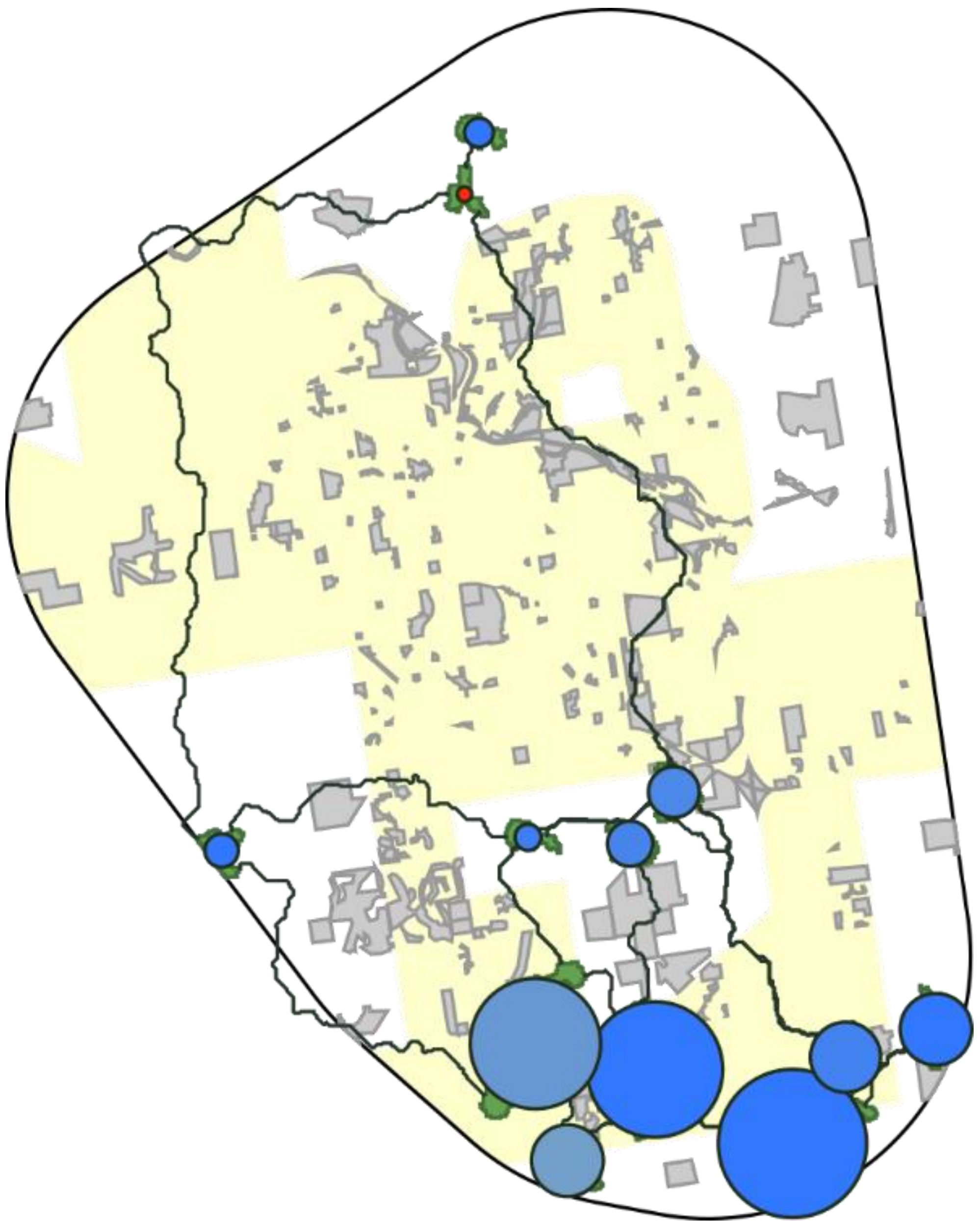

S62

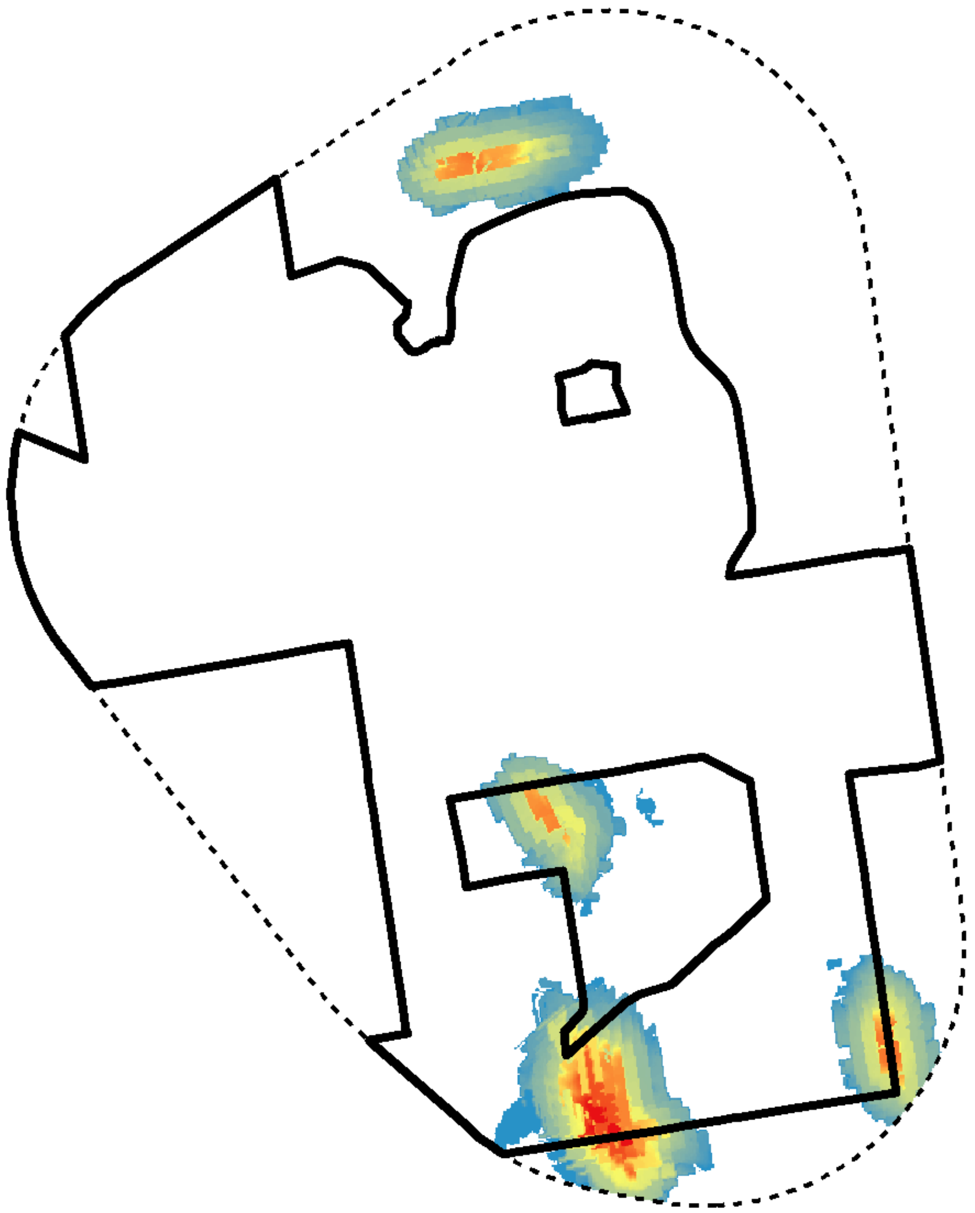

S63

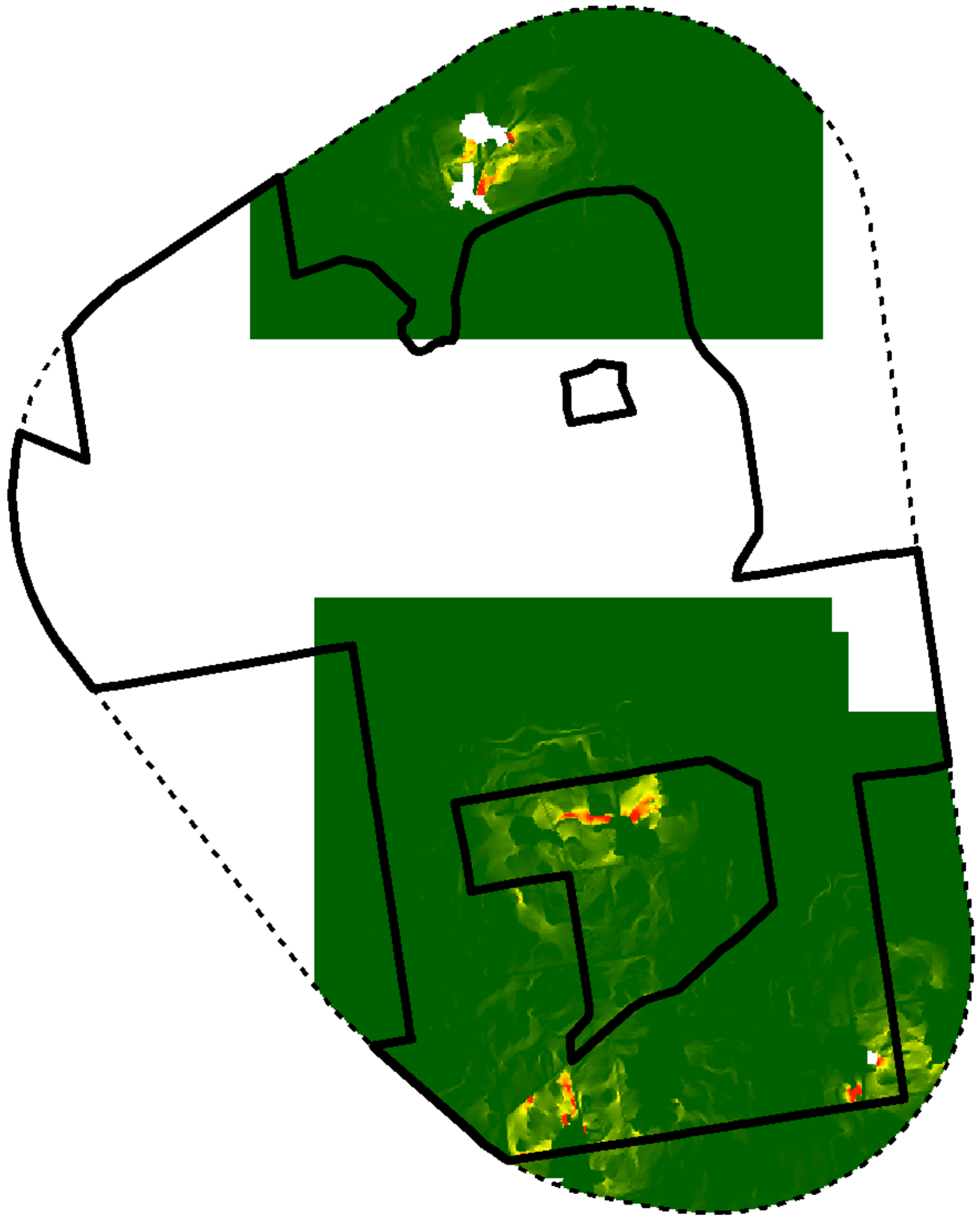

S64

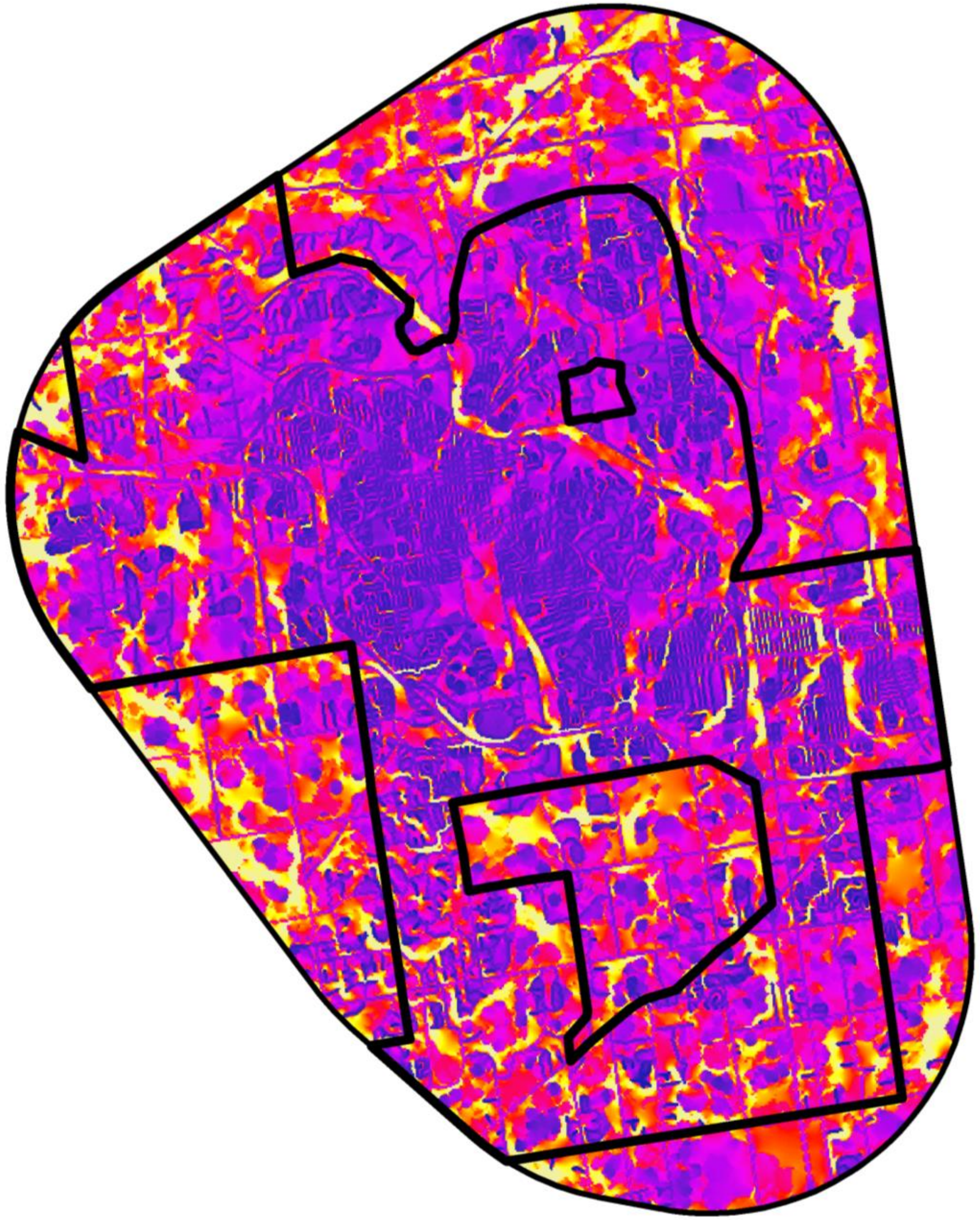

Supplementary Figure 65. Habitat network map for Northern long-eared bats (*Myotis septentrionalis*) in Detroit Southwest site showing habitat patch size as the size of the circle and importance of the patch in maintaining overall network connectivity with warming colors (orange and red) indicating highest importance.

Supplementary Figure 66. Map of locations where removal of a barrier (red areas) would improve connectivity for Northern long-eared bats (*Myotis septentrionalis*) in the Detroit Southwest site.

Supplementary Figure 67. Map of areas with narrow linkages (yellow and red areas) where Northern long-eared bats (*Myotis septentrionalis*) would have limited movement options in Detroit Southwest site making them important corridors to maintain.

Supplementary Figure 68. Current density map for Northern long-eared bats (*Myotis septentrionalis*) in Detroit Southwest site. Areas with higher current density are predicted to represent better movement corridors.

S65

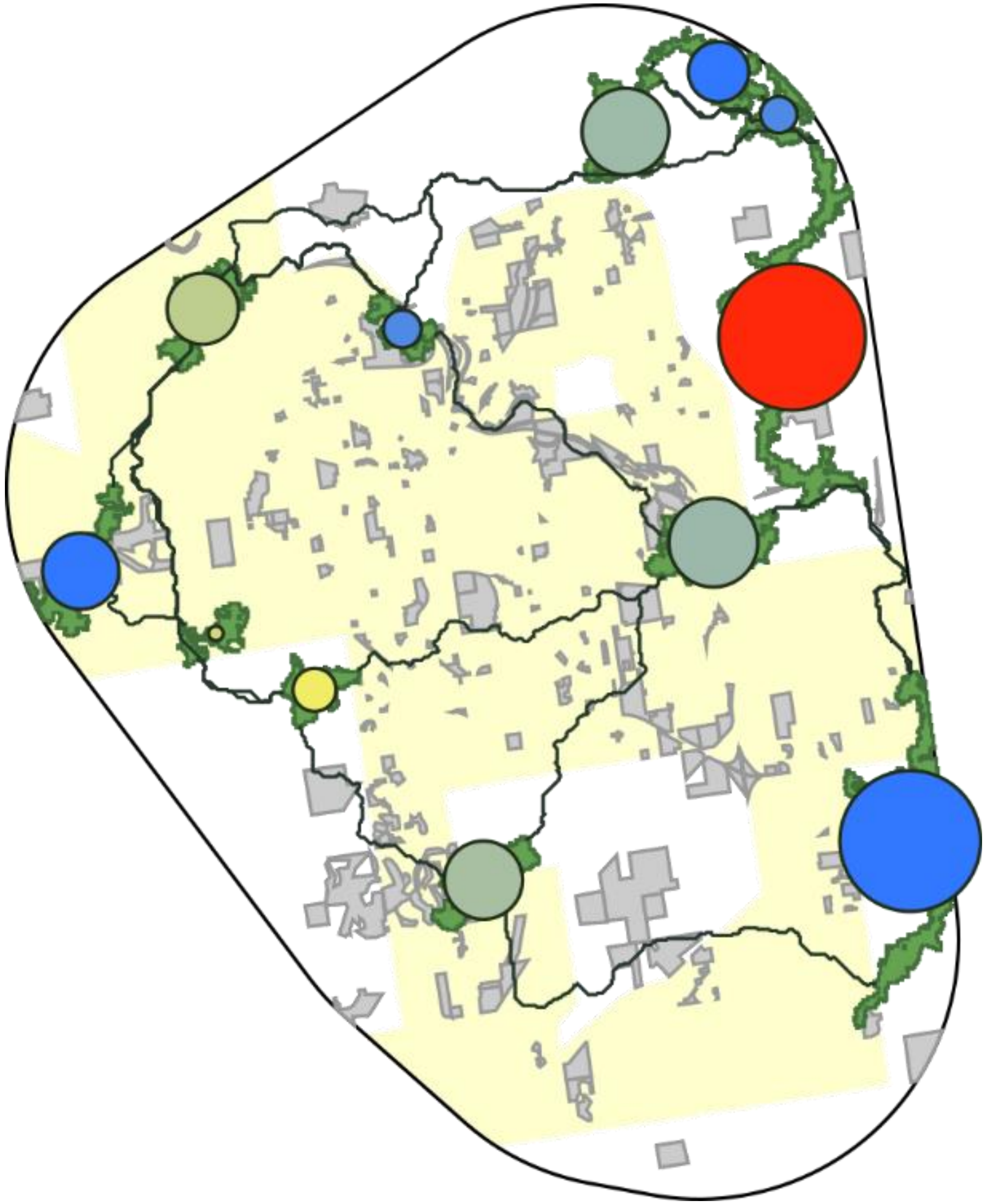

S66

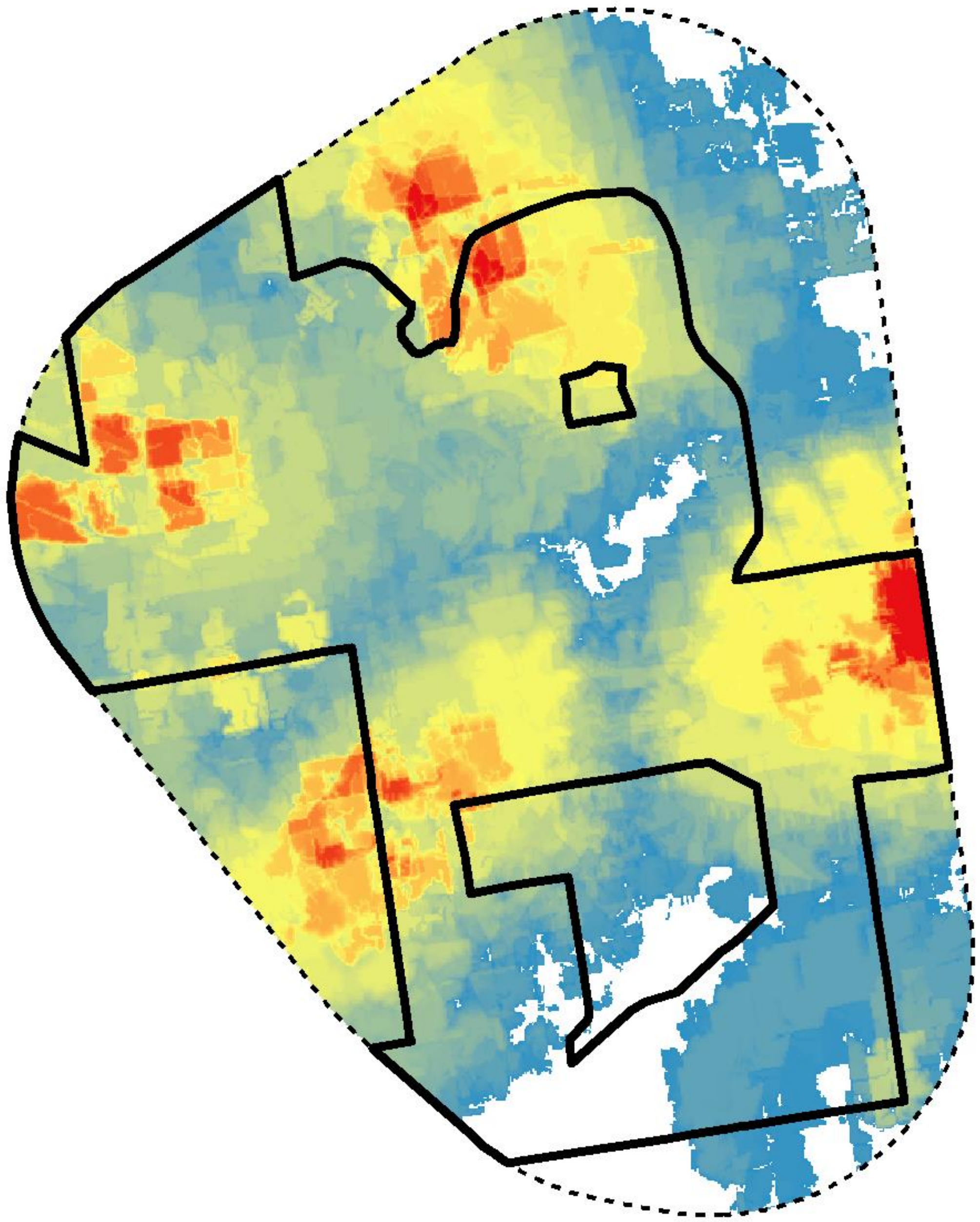

S67

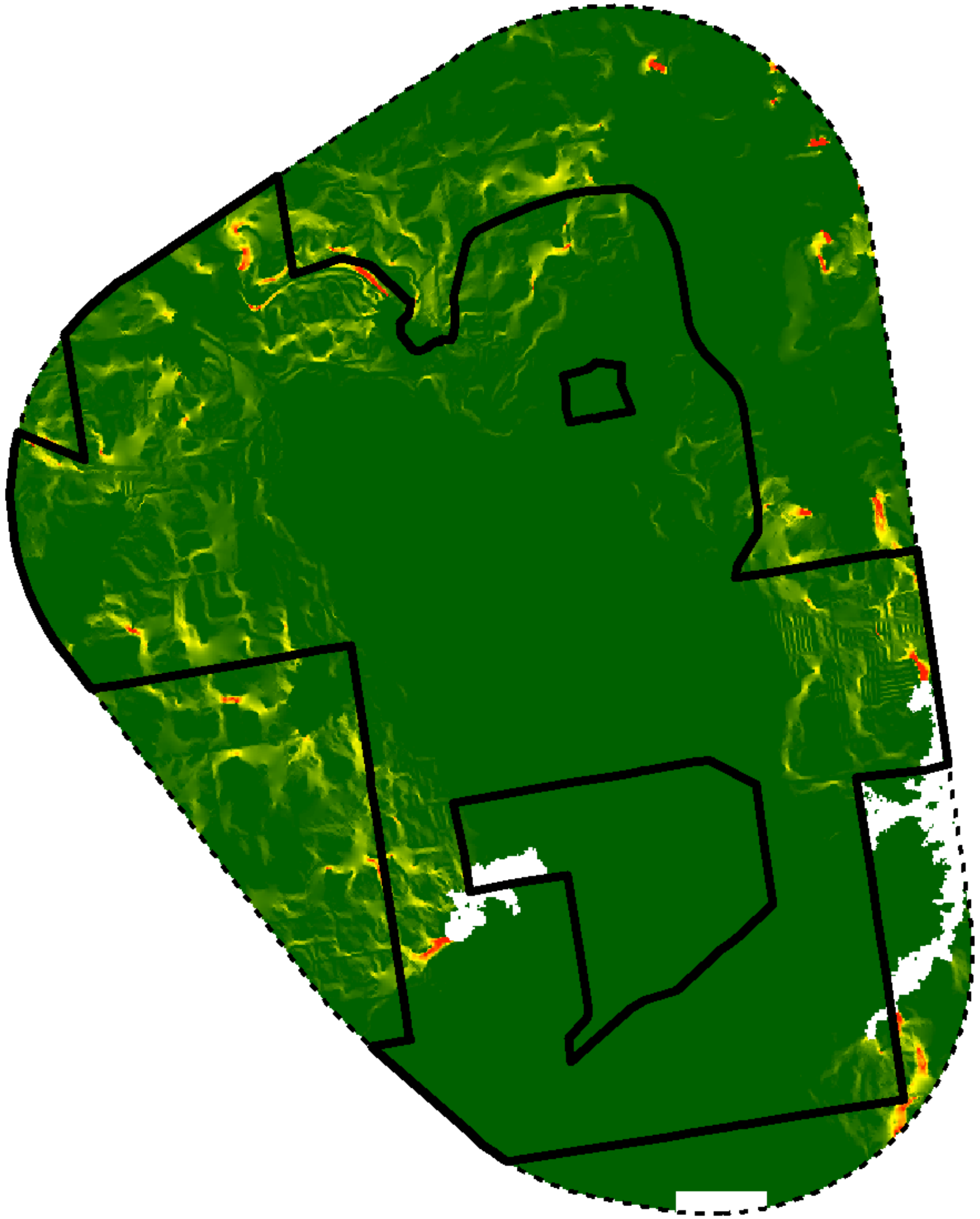

S68

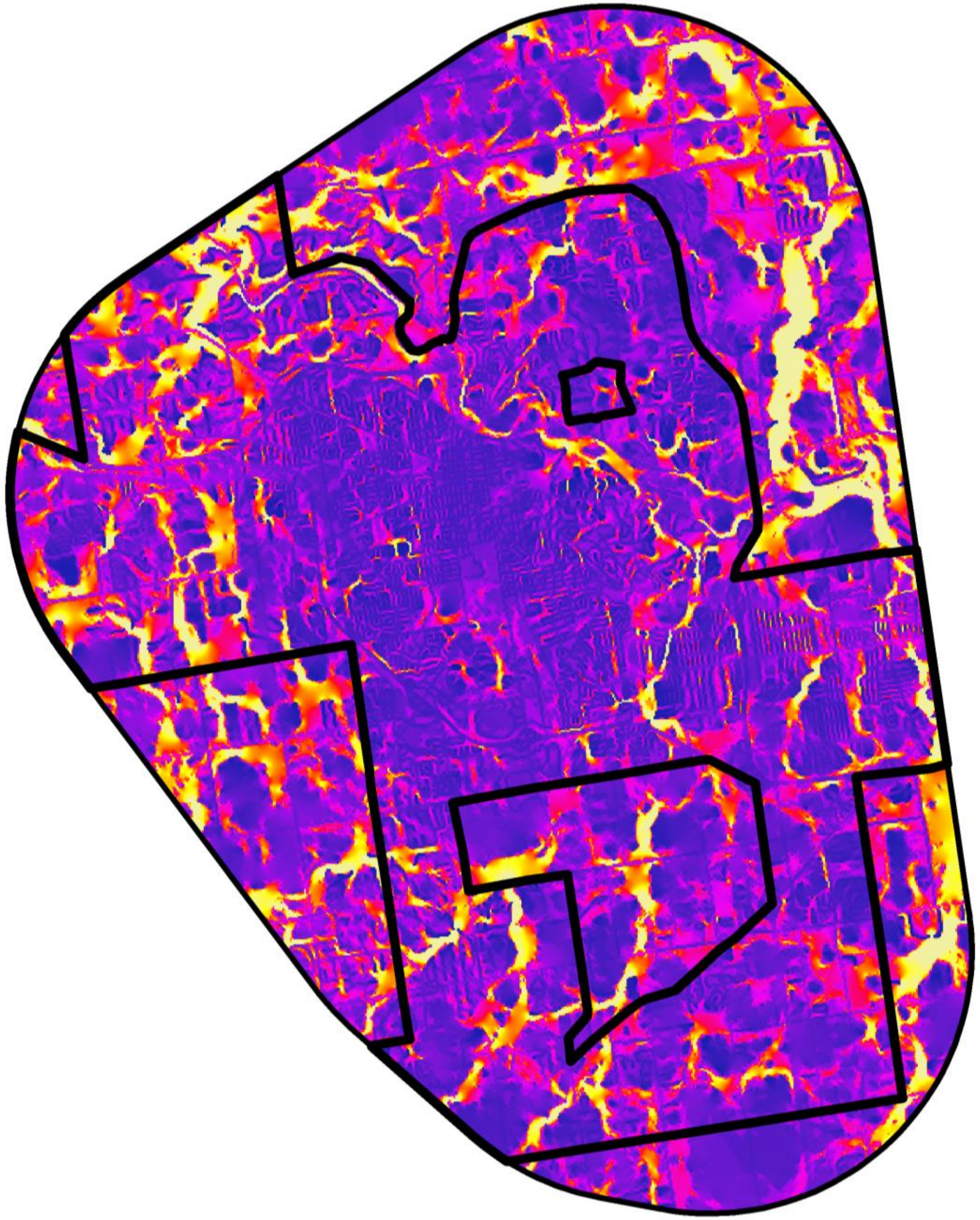

Supplementary Figure 69. Habitat network map for Eastern Massasauga Rattlesnakes (*Sistrurus catenatus*) in Detroit West site showing habitat patch size as the size of the circle and importance of the patch in maintaining overall network connectivity with warming colors (orange and red) indicating highest importance.

Supplementary Figure 70. Map of locations where removal of a barrier (red areas) would improve connectivity for Eastern Massasauga Rattlesnakes (*Sistrurus catenatus*) in the Detroit West site.

Supplementary Figure 71. Map of areas with narrow linkages (yellow and red areas) where Eastern Massasauga Rattlesnakes (*Sistrurus catenatus*) would have limited movement options in Detroit West site making them important corridors to maintain.

Supplementary Figure 72. Current density map for Eastern Massasauga Rattlesnakes (*Sistrurus catenatus*) in Detroit West site. Areas with higher current density are predicted to represent better movement corridors.

S69

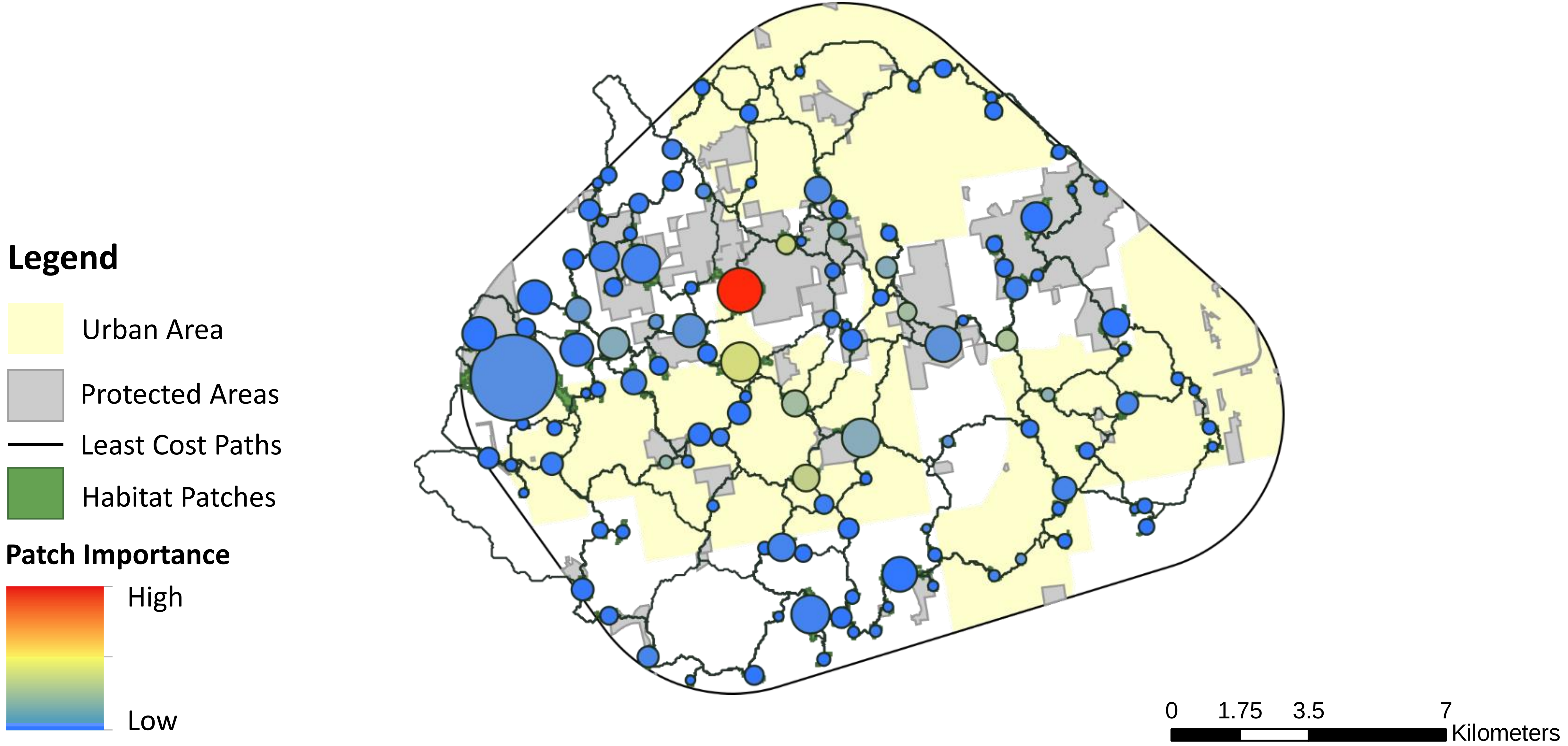
Legend
Urban Area
Protected Areas
Least Cost Paths
Habitat Patches
Patch Importance
High
Low
0
1.75
3.5
7
Kilometers

S70

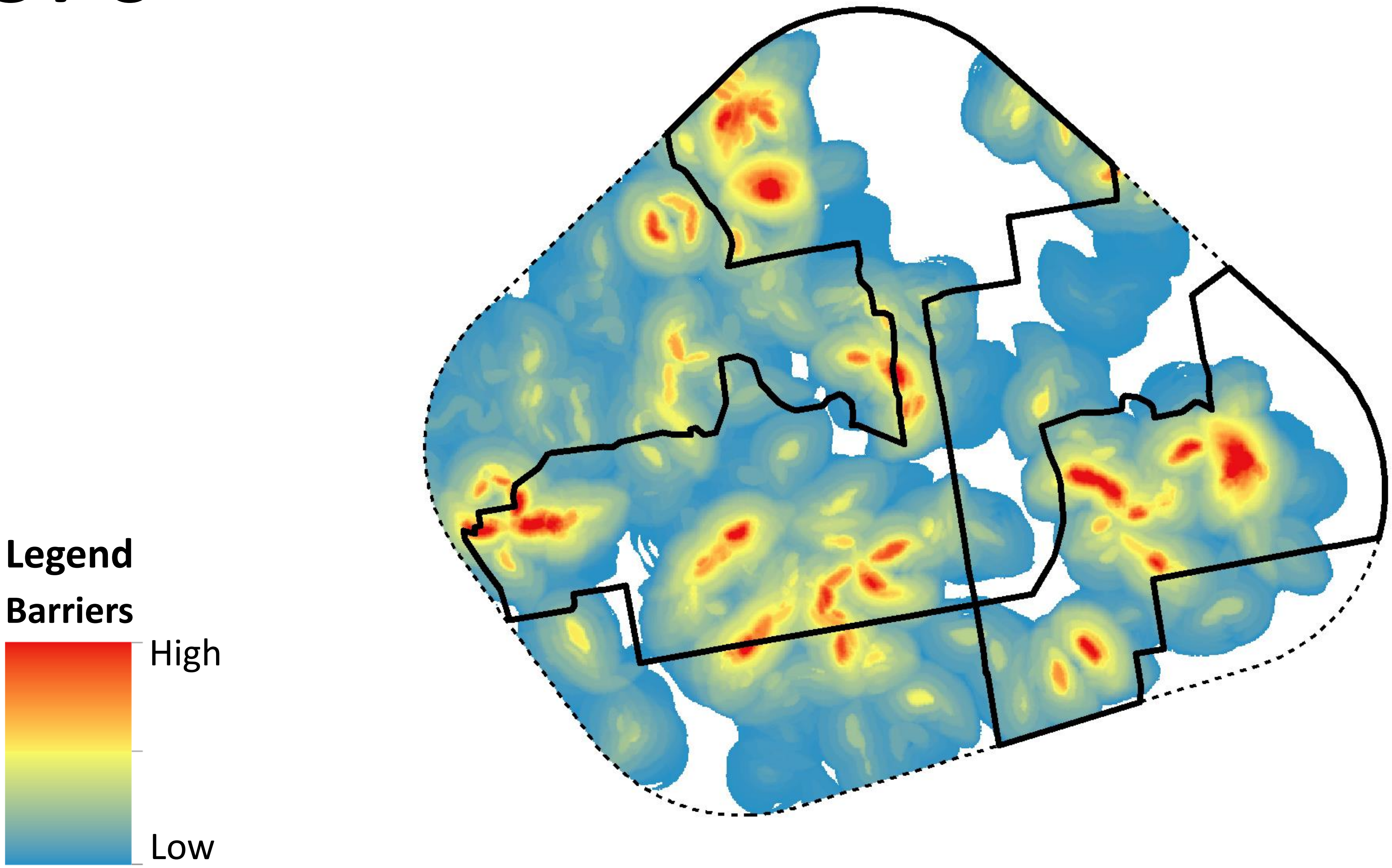

S71

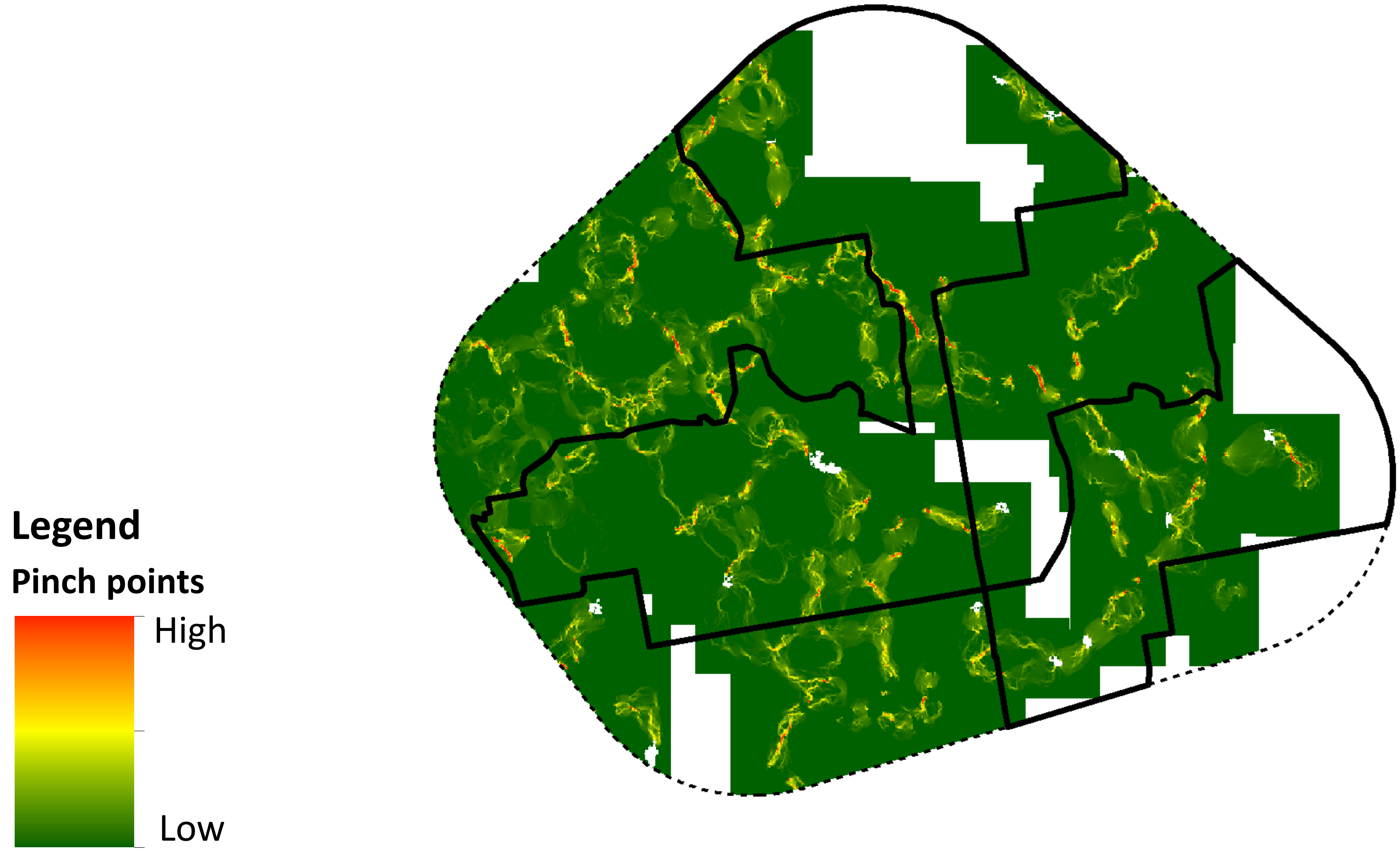

S72

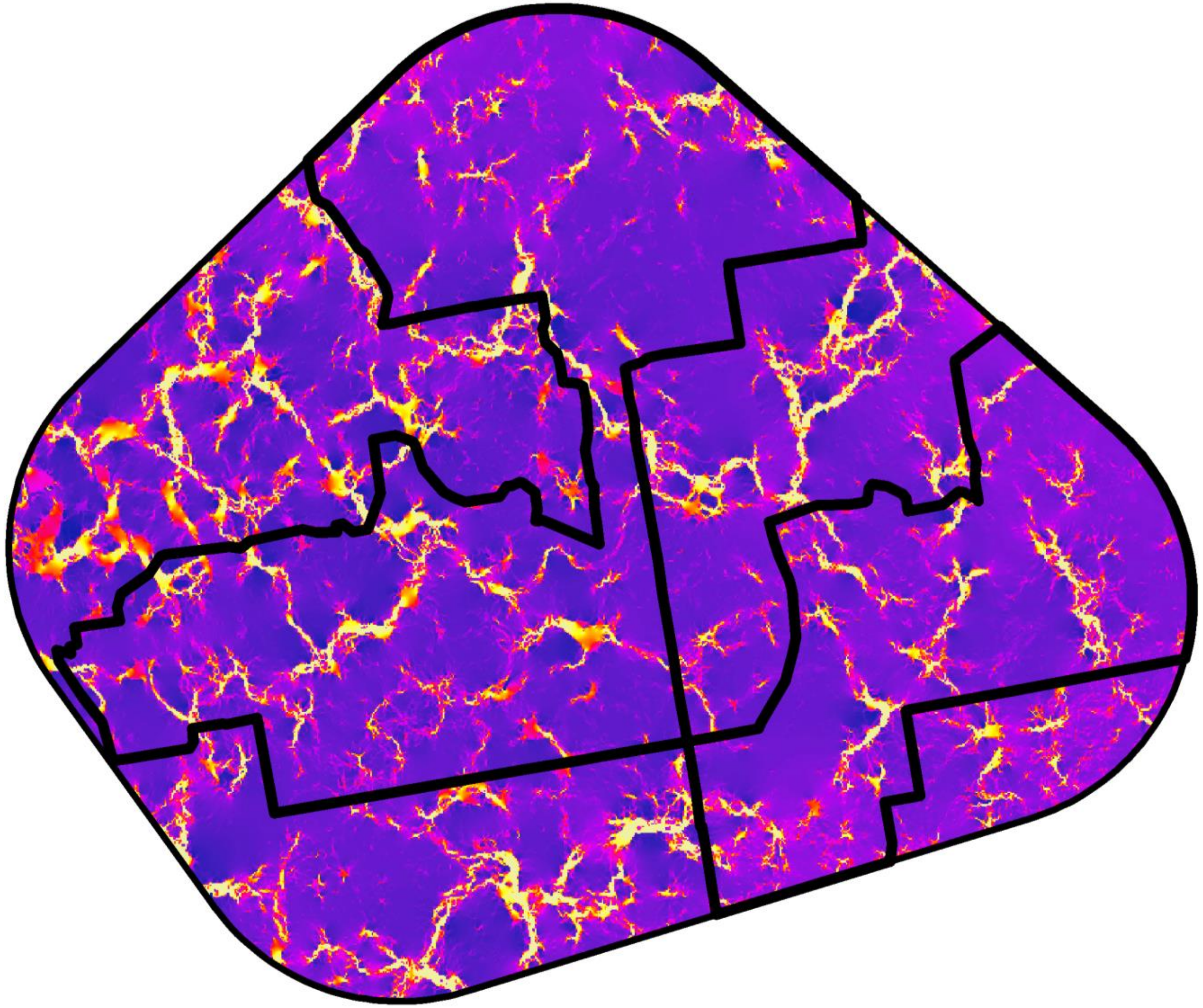

Legend

Current density

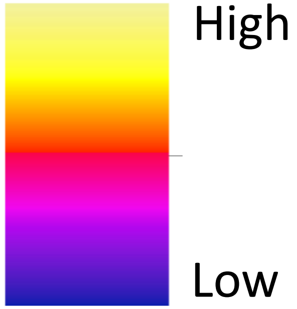

Supplementary Figure 73. Habitat network map for Spotted Turtles (*Clemmys guttata*) in Detroit West site showing habitat patch size as the size of the circle and importance of the patch in maintaining overall network connectivity with warming colors (orange and red) indicating highest importance.

Supplementary Figure 74. Map of locations where removal of a barrier (red areas) would improve connectivity for Spotted Turtles (*Clemmys guttata*) in the Detroit West site.

Supplementary Figure 75. Map of areas with narrow linkages (yellow and red areas) where Spotted Turtles (*Clemmys guttata*) would have limited movement options in Detroit West site making them important corridors to maintain.

Supplementary Figure 76. Current density map for Spotted Turtles (*Clemmys guttata*) in Detroit West site. Areas with higher current density are predicted to represent better movement corridors.

S73

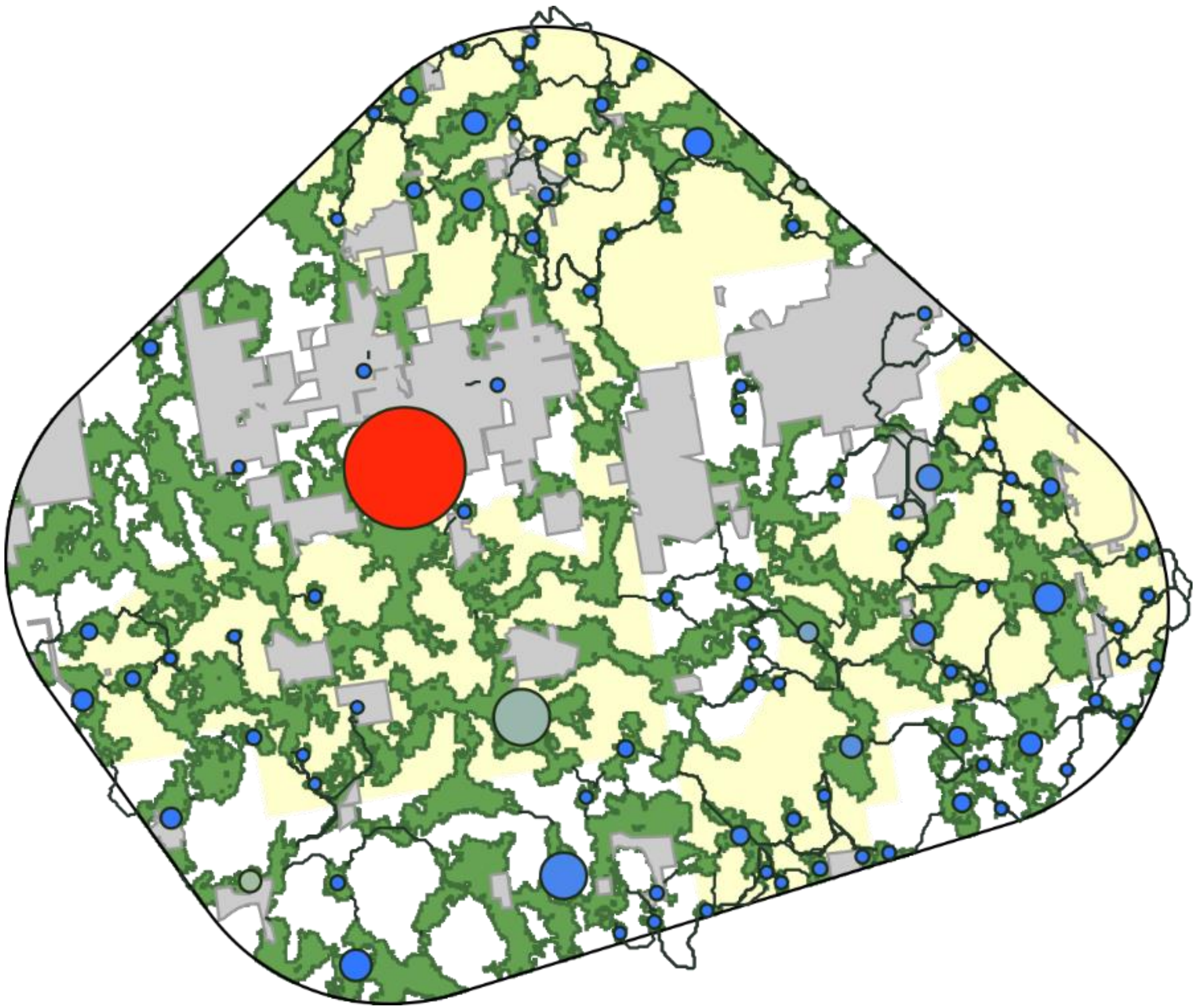

S74

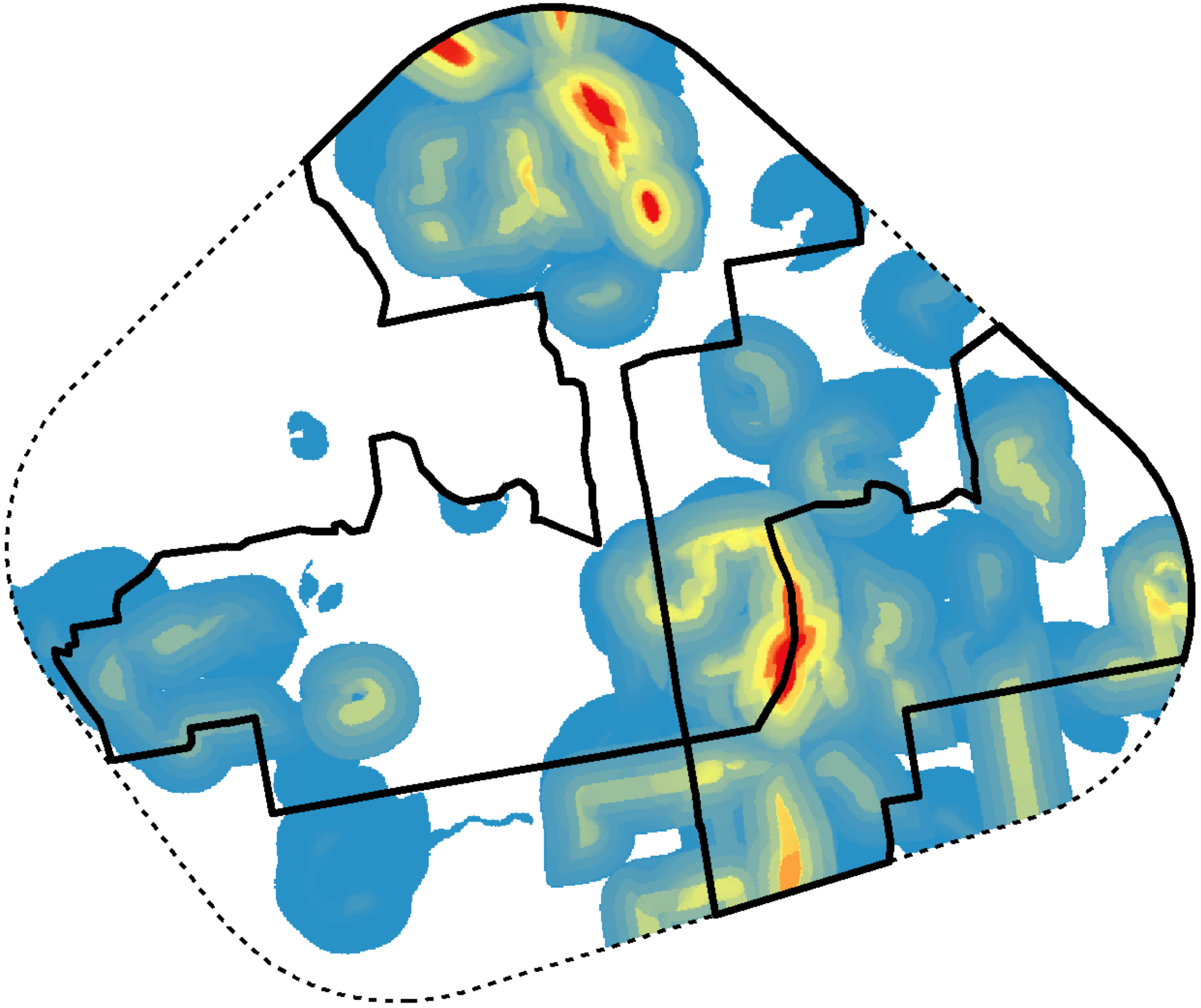

S75

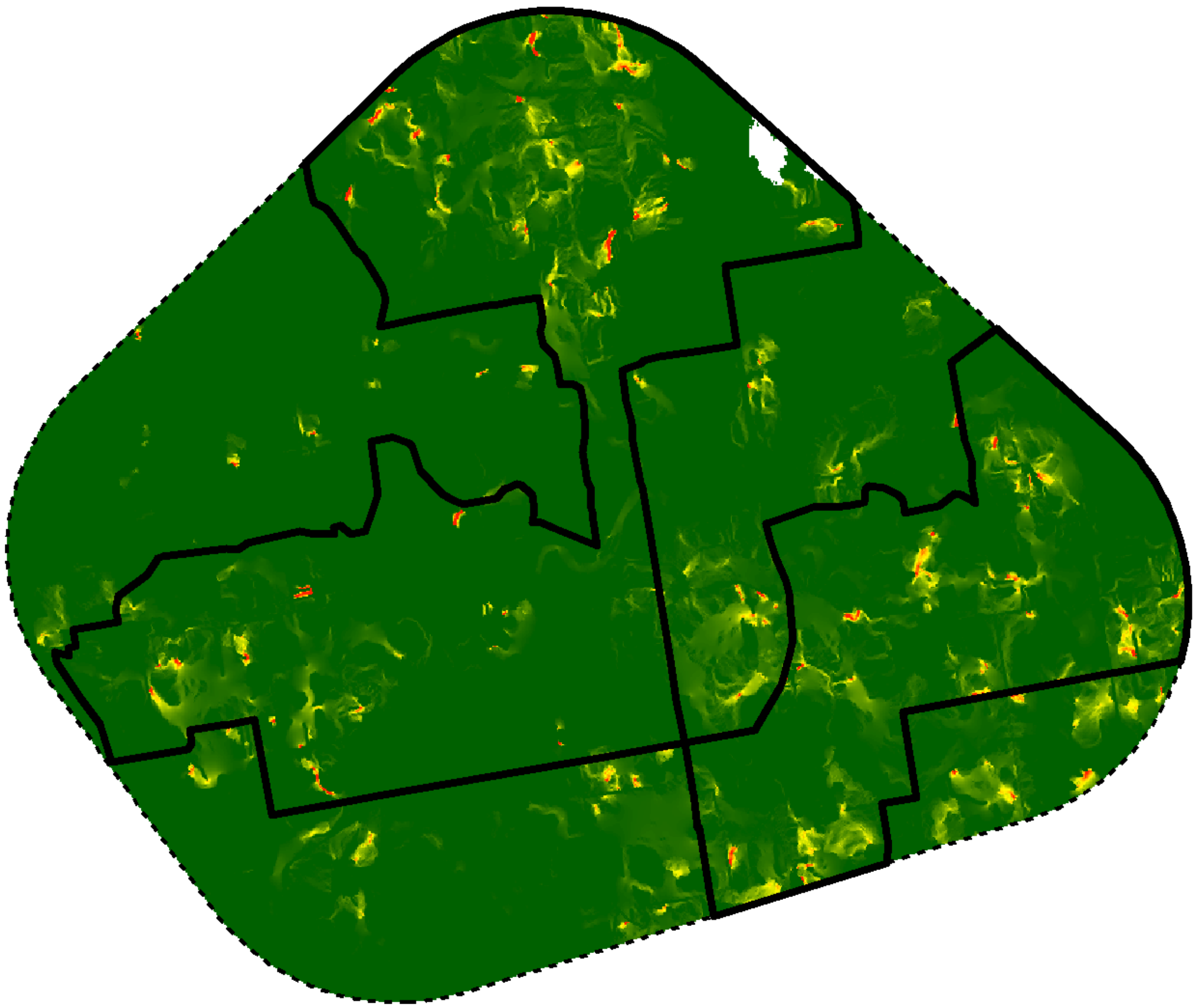

S76

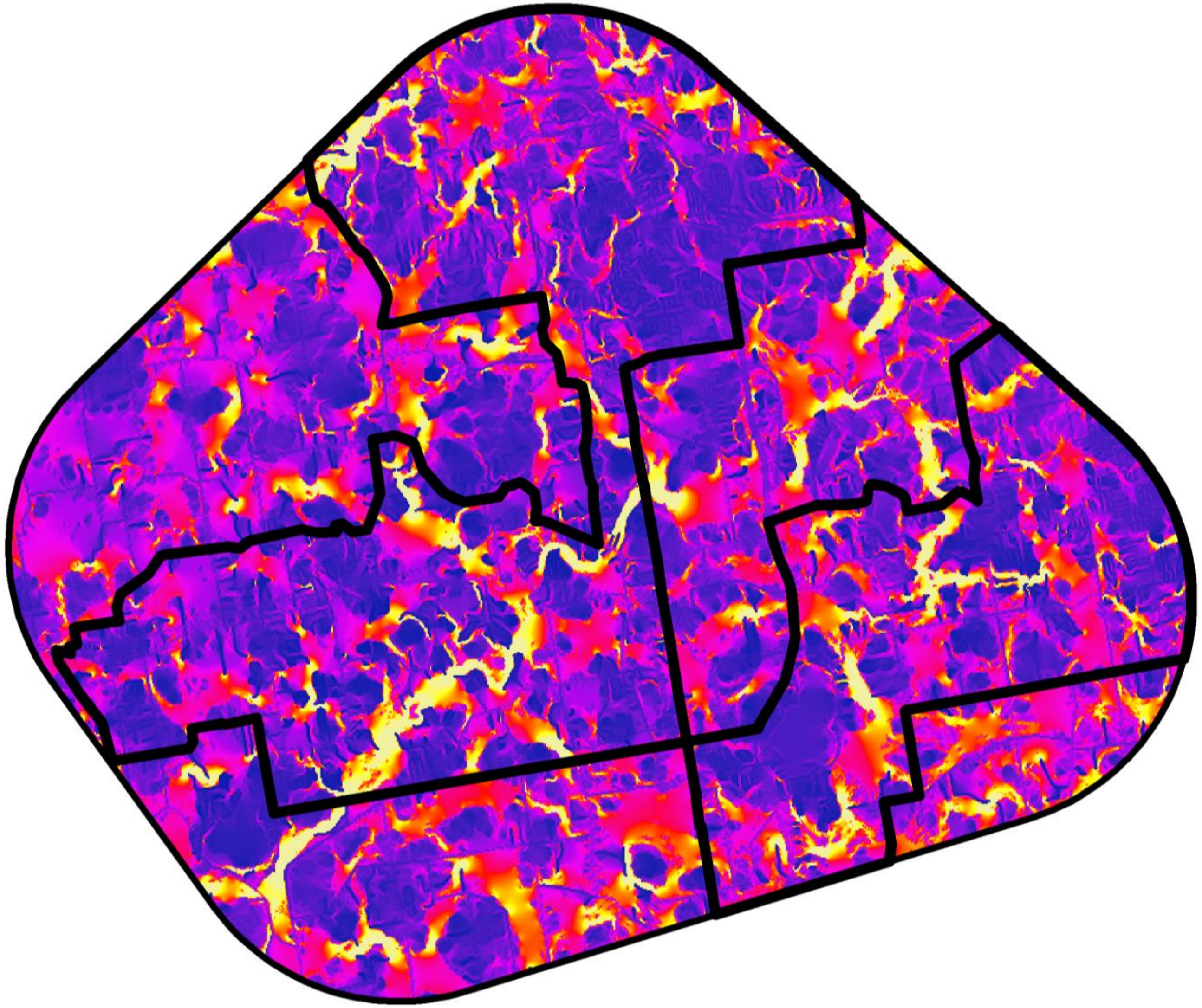

Supplementary Figure 77. Habitat network map for Black and Gold Bumble Bees (*Bombus auricomus*) in Detroit North site showing habitat patch size as the size of the circle and importance of the patch in maintaining overall network connectivity with warming colors (orange and red) indicating highest importance.

Supplementary Figure 78. Map of locations where removal of a barrier (red areas) would improve connectivity for Black and Gold Bumble Bees (*Bombus auricomus*) in the Detroit North site.

Supplementary Figure 79. Map of areas with narrow linkages (yellow and red areas) where Black and Gold Bumble Bees (*Bombus auricomus*) would have limited movement options in Detroit North site making them important corridors to maintain.

Supplementary Figure 80. Current density map for Black and Gold Bumble Bees (*Bombus auricomus*) in Detroit North site. Areas with higher current density are predicted to represent better movement corridors.

S77

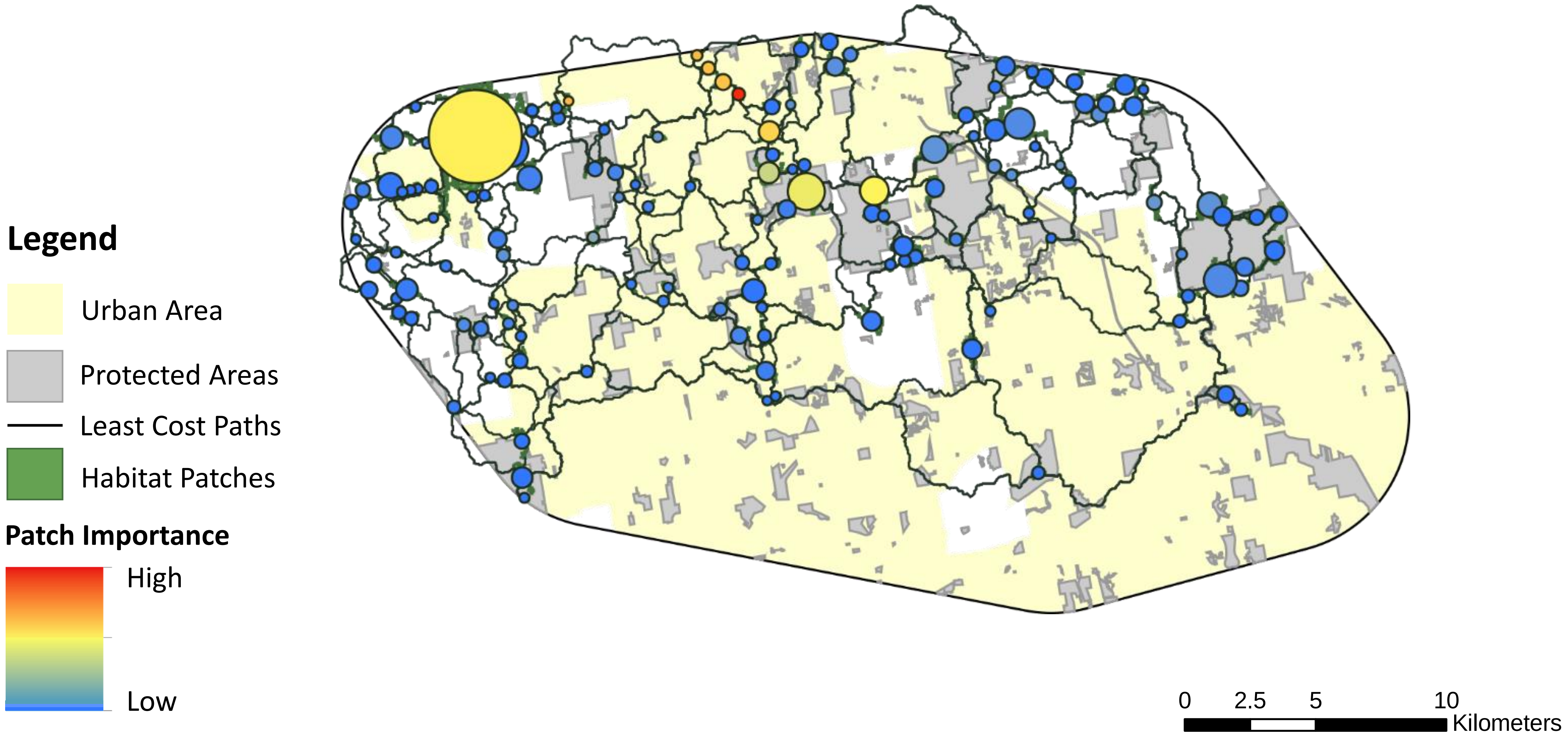
Legend
Urban Area
Protected Areas
Least Cost Paths
Habitat Patches
Patch Importance
High
Low
0 2.5 5 10
Kilometers

S78

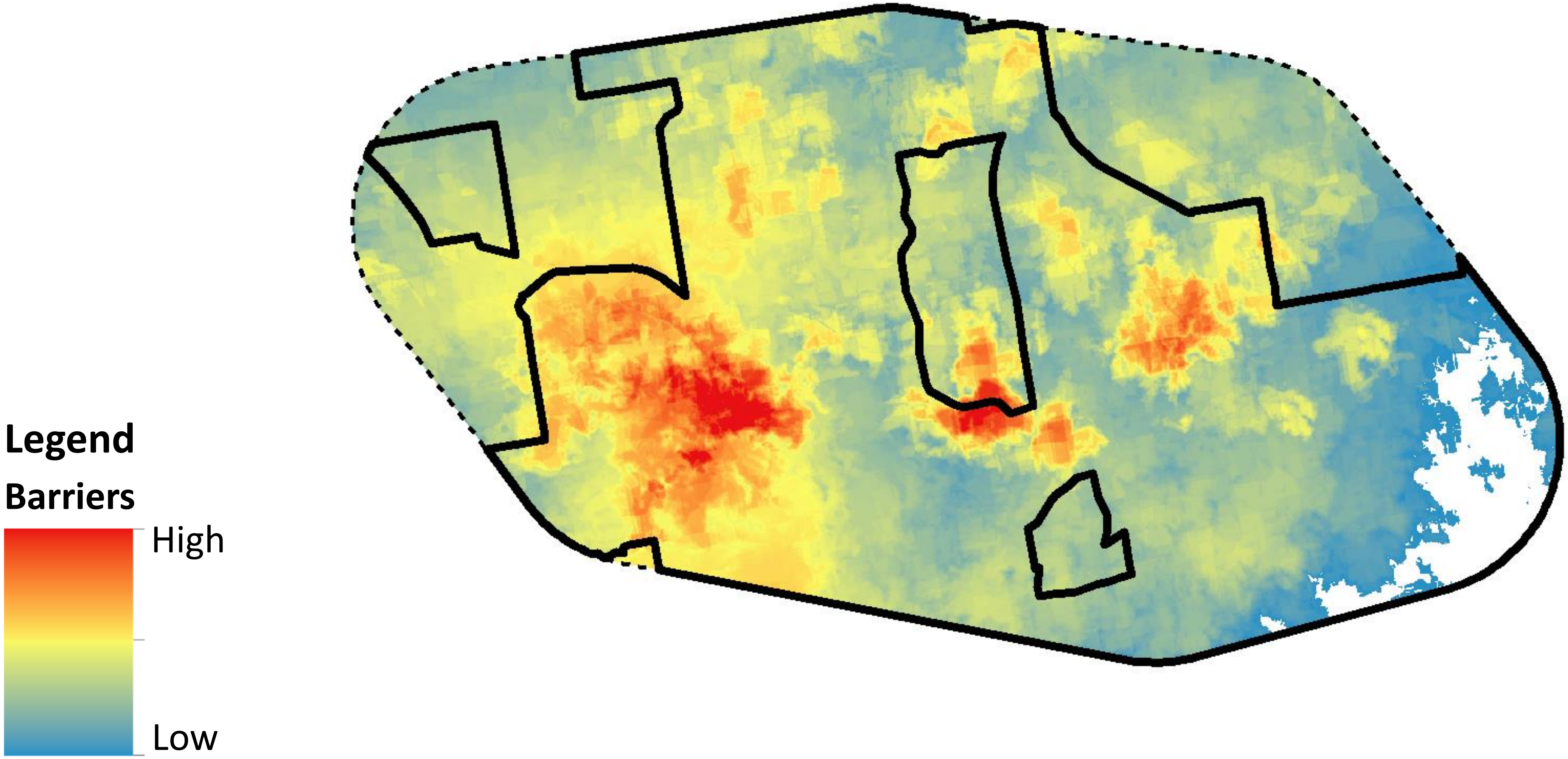

S79

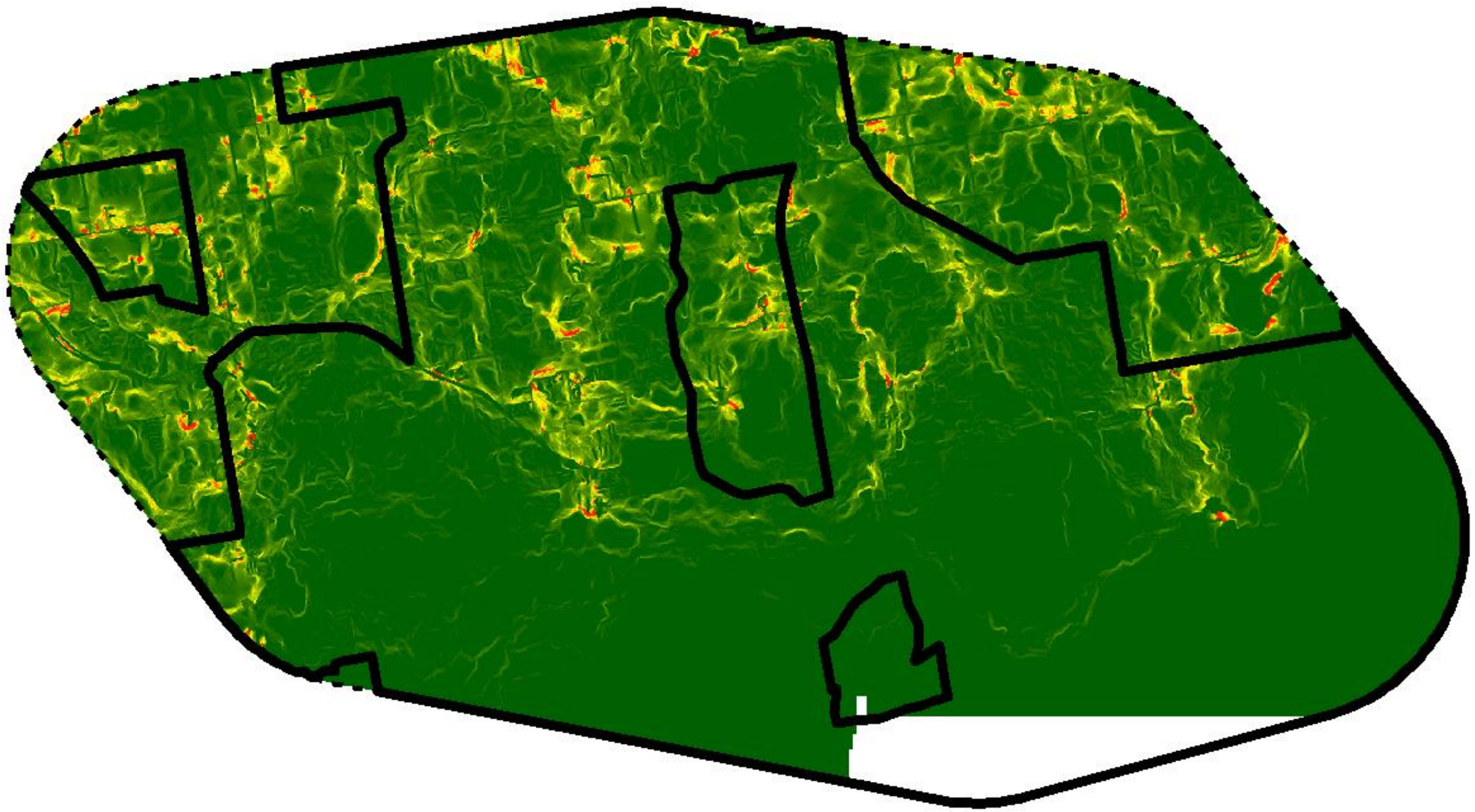

Legend

Pinch points

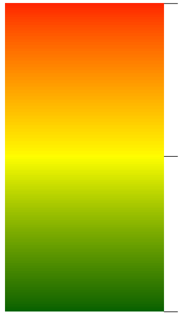

S80

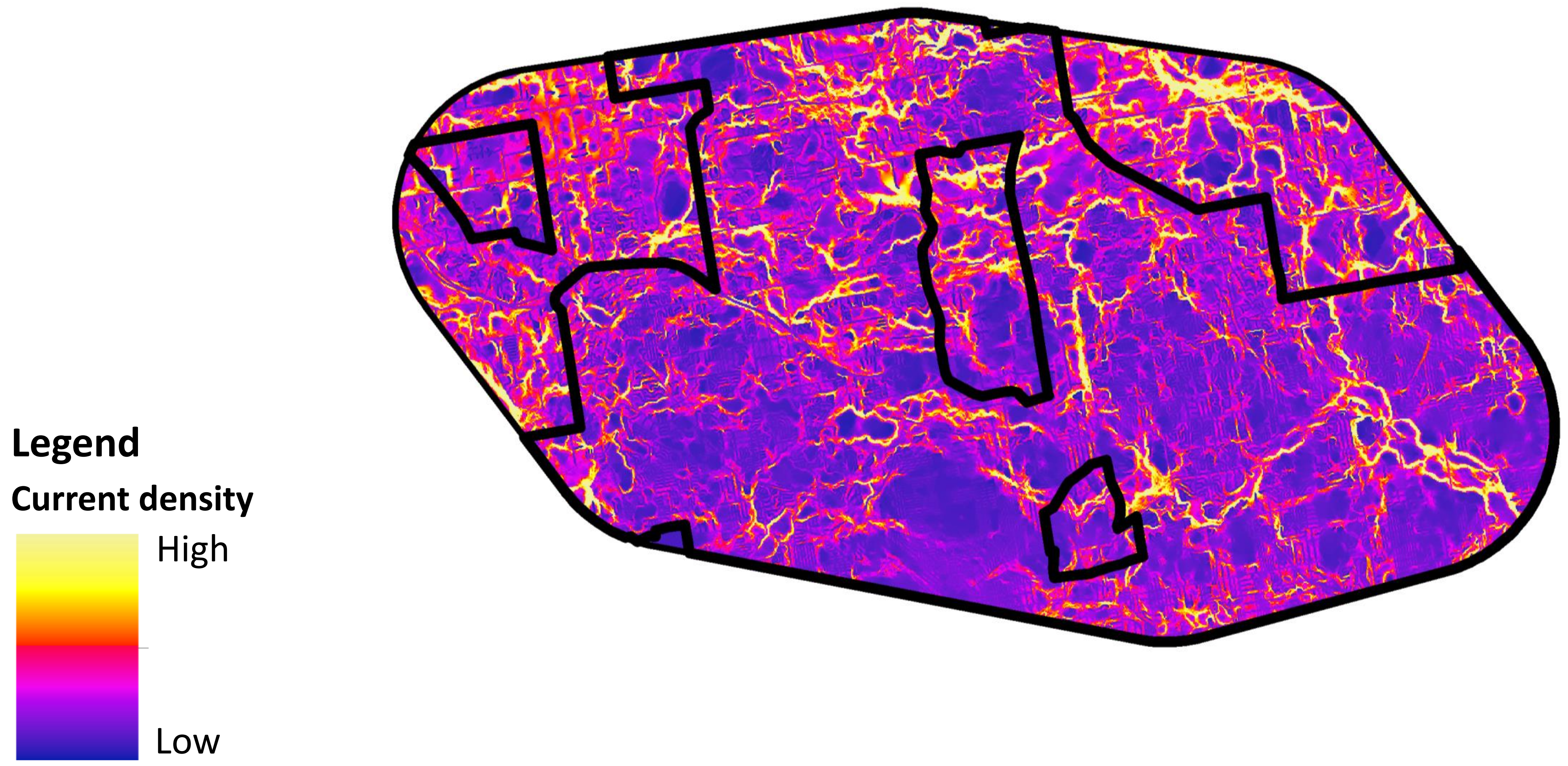

Supplementary Figure 81. Habitat network map for Blanding's Turtles (*Emydoidea blandingii*) in Detroit North site showing habitat patch size as the size of the circle and importance of the patch in maintaining overall network connectivity with warming colors (orange and red) indicating highest importance.

Supplementary Figure 82. Map of locations where removal of a barrier (red areas) would improve connectivity for Blanding's Turtles (*Emydoidea blandingii*) in the Detroit North site.

Supplementary Figure 83. Map of areas with narrow linkages (yellow and red areas) where Blanding's Turtles (*Emydoidea blandingii*) would have limited movement options in Detroit North site making them important corridors to maintain.

Supplementary Figure 84. Current density map for Blanding's Turtles (*Emydoidea blandingii*) in Detroit North site. Areas with higher current density are predicted to represent better movement corridors.

S81

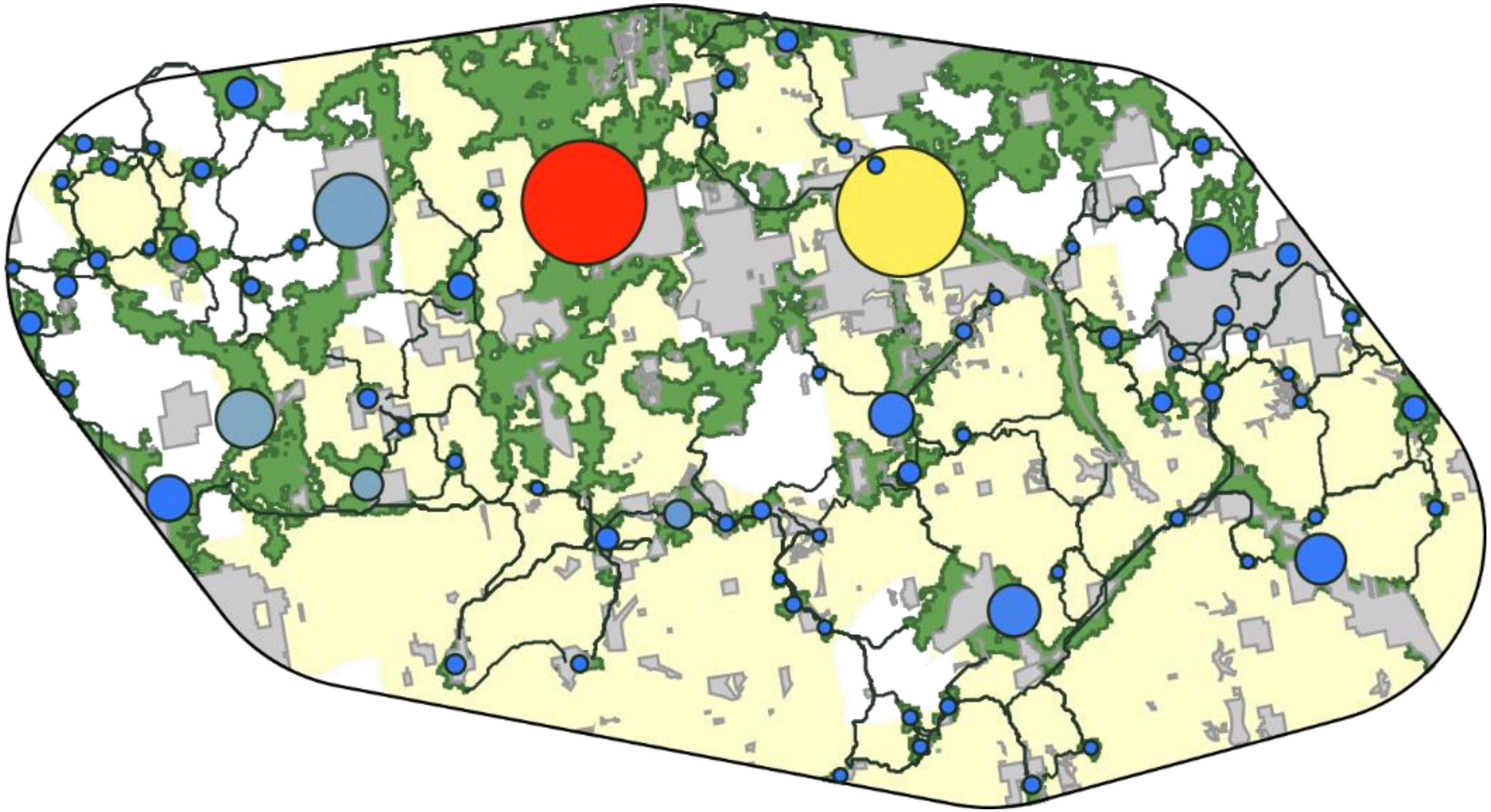

S82

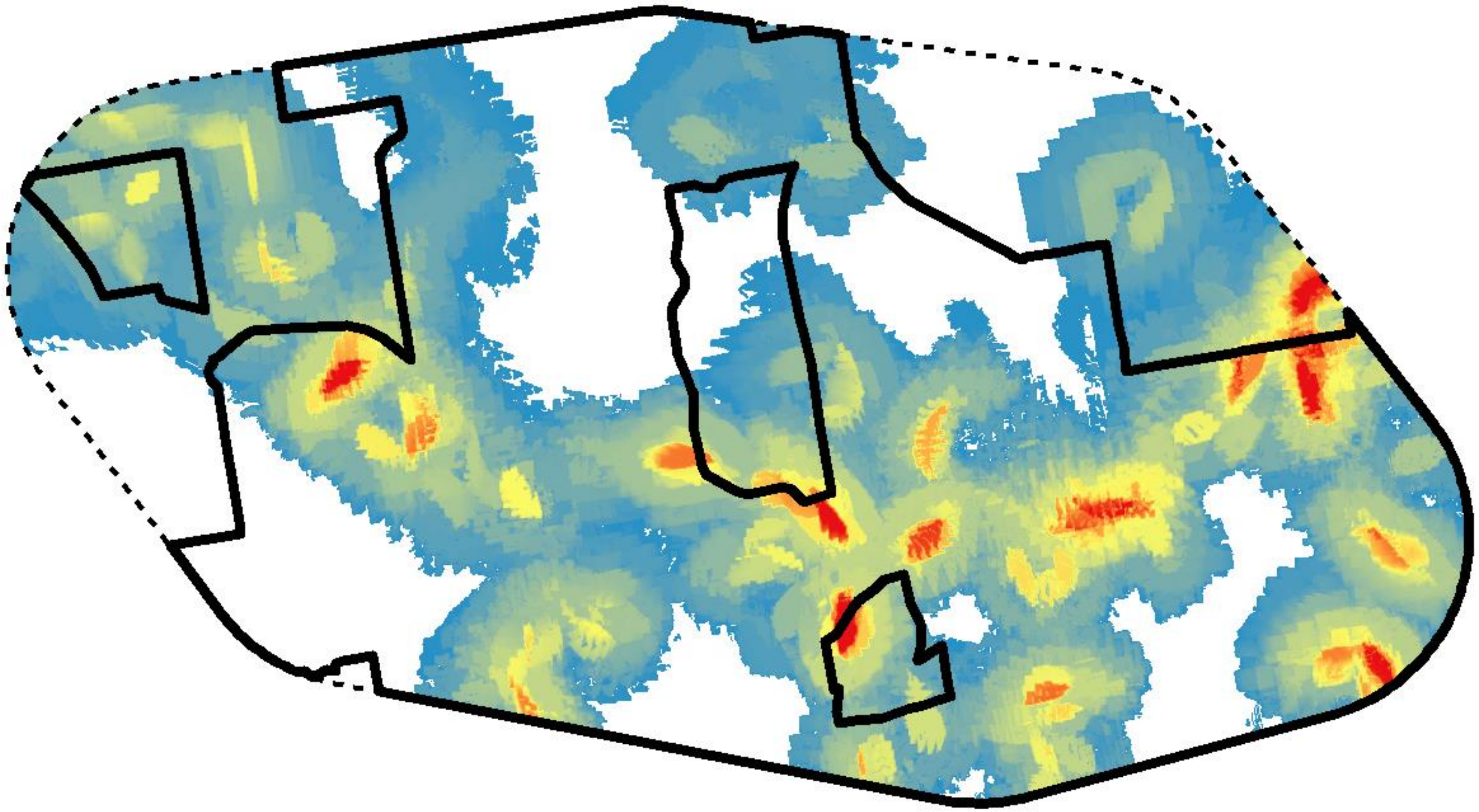

S83

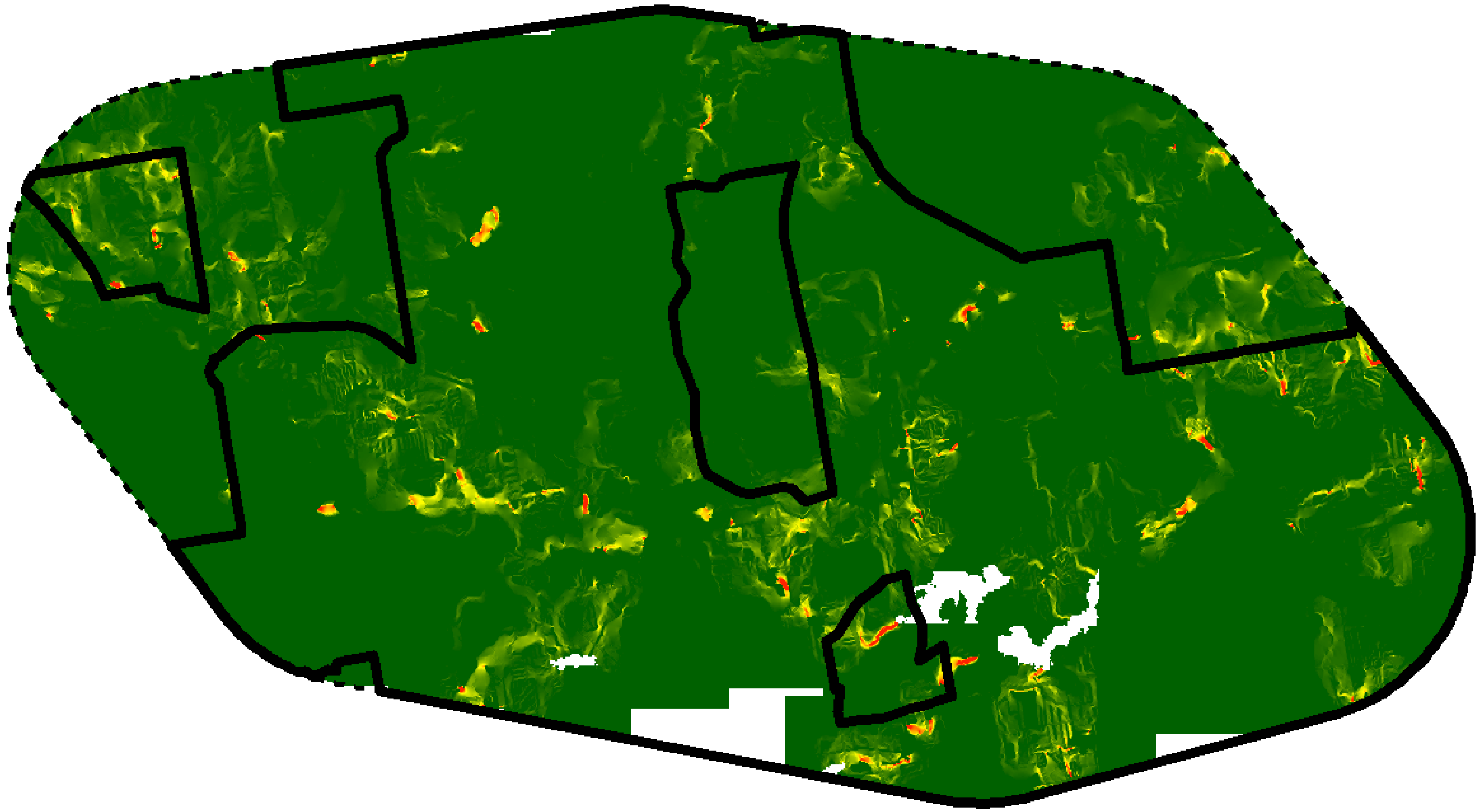

S84

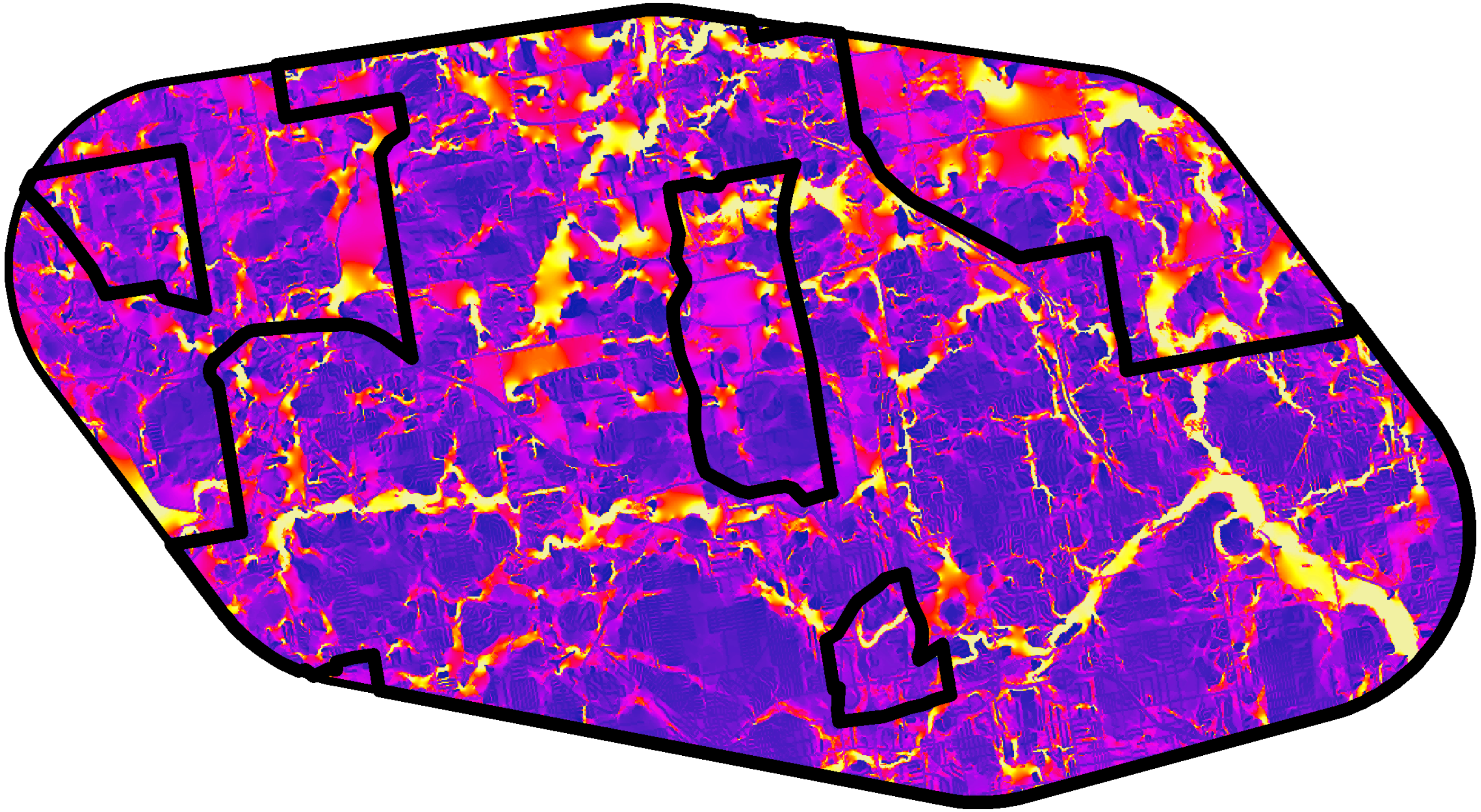

Supplementary Figure 85. Habitat network map for Eastern Massasauga Rattlesnakes (*Sistrurus catenatus*) in Detroit North site showing habitat patch size as the size of the circle and importance of the patch in maintaining overall network connectivity with warming colors (orange and red) indicating highest importance.

Supplementary Figure 86. Map of locations where removal of a barrier (red areas) would improve connectivity for Eastern Massasauga Rattlesnakes (*Sistrurus catenatus*) in the Detroit North site.

Supplementary Figure 87. Map of areas with narrow linkages (yellow and red areas) where Eastern Massasauga Rattlesnakes (*Sistrurus catenatus*) would have limited movement options in Detroit North site making them important corridors to maintain.

Supplementary Figure 88. Current density map for Eastern Massasauga Rattlesnakes (*Sistrurus catenatus*) in Detroit North site. Areas with higher current density are predicted to represent better movement corridors.

S85

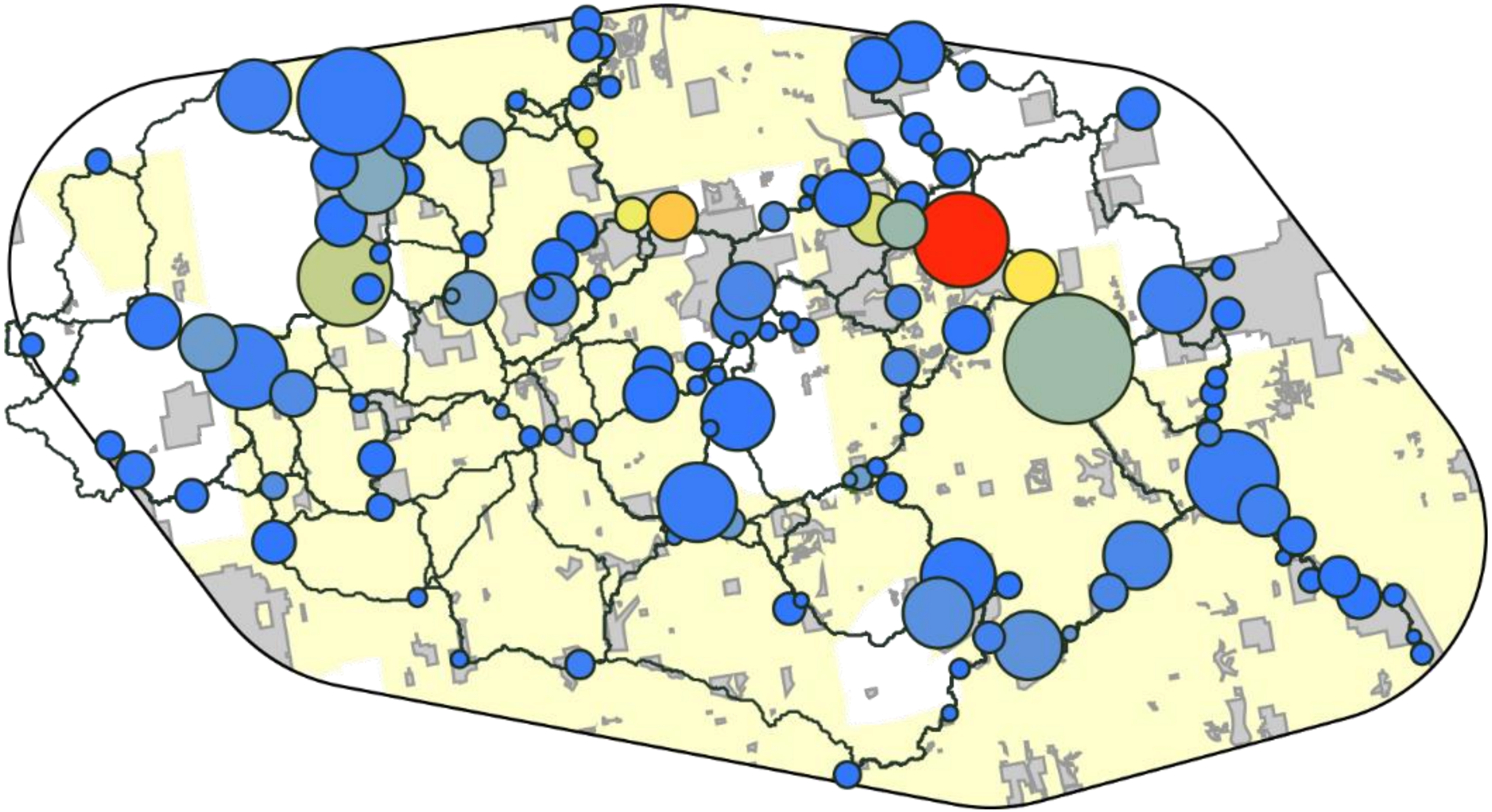

S86

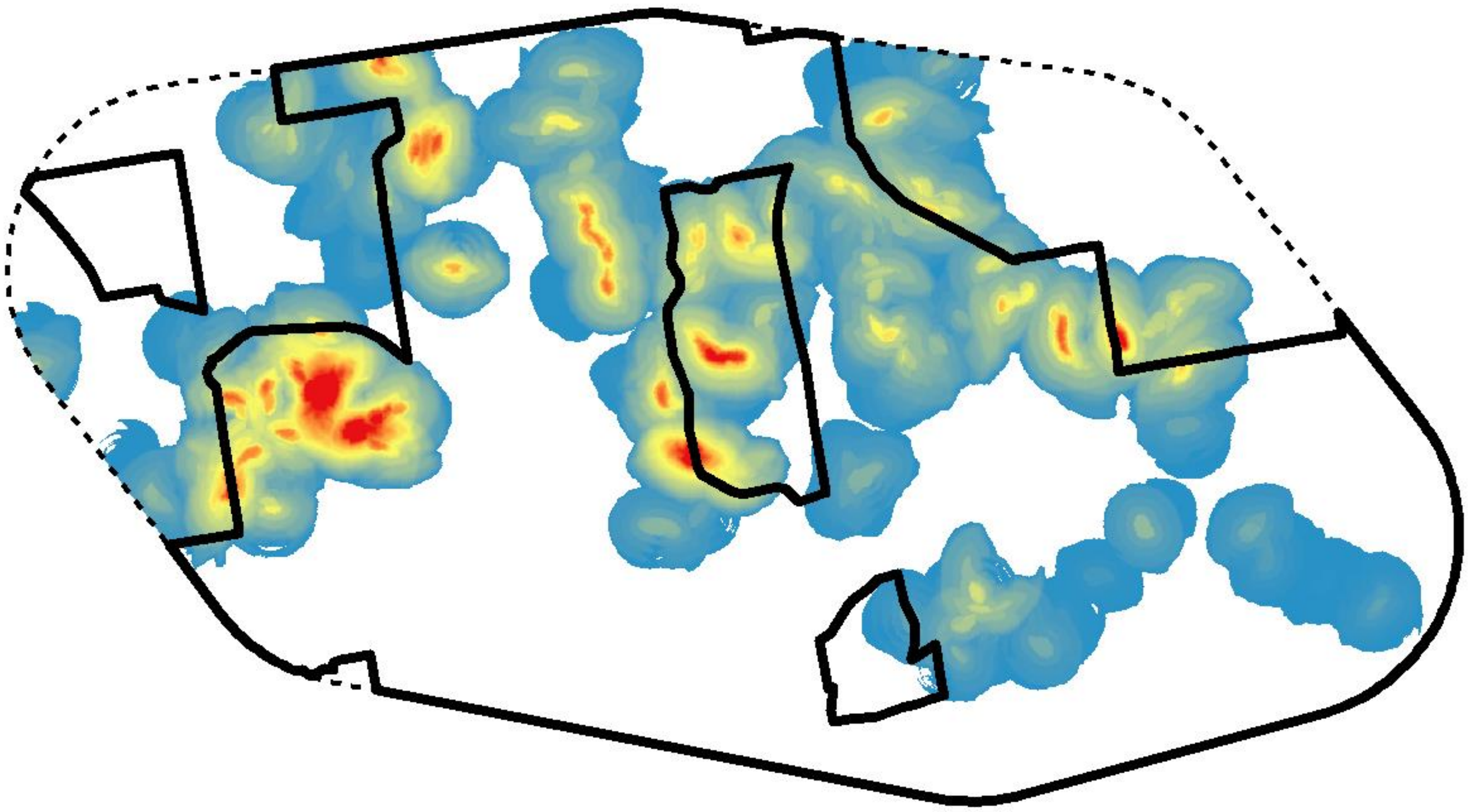

S87

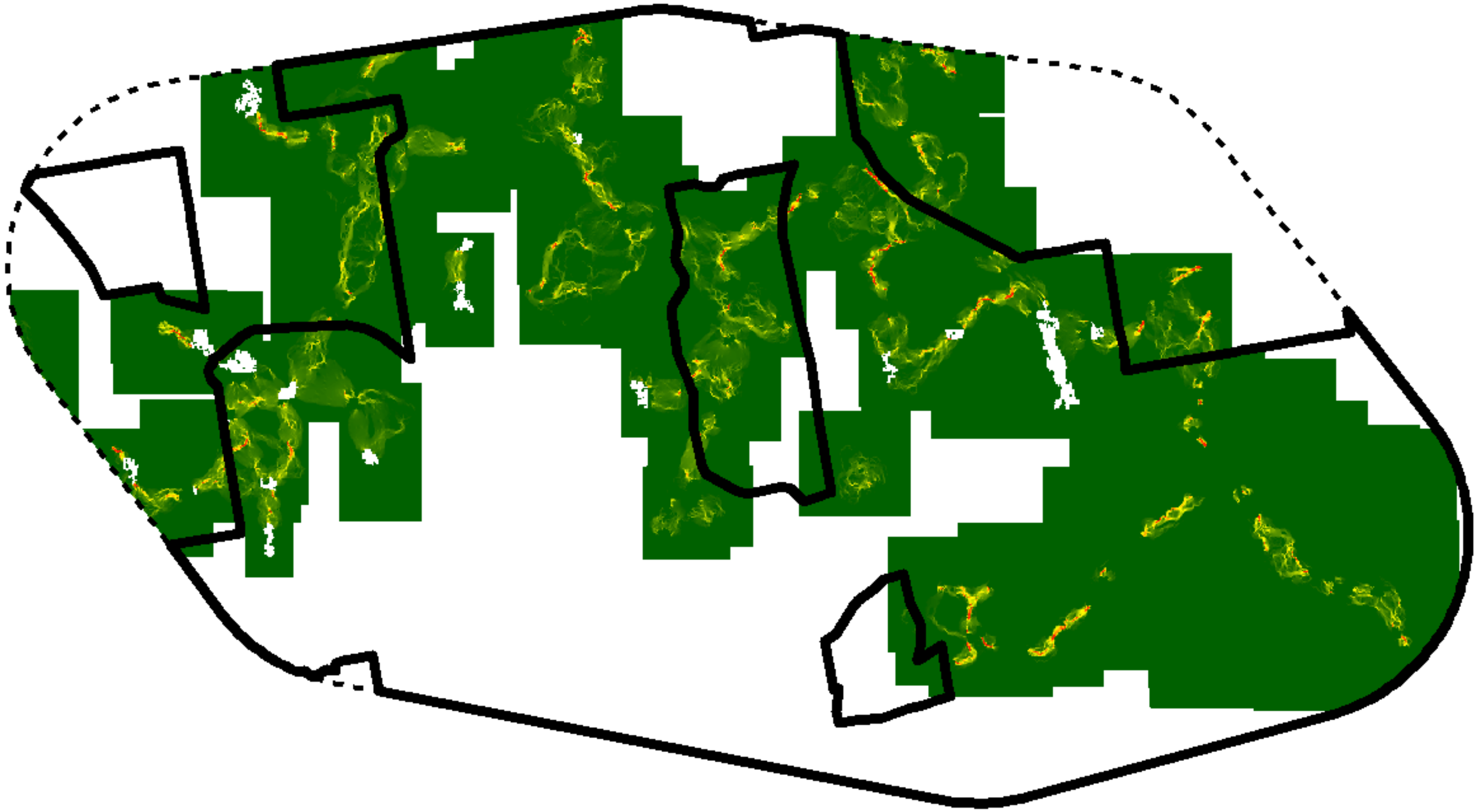

S88

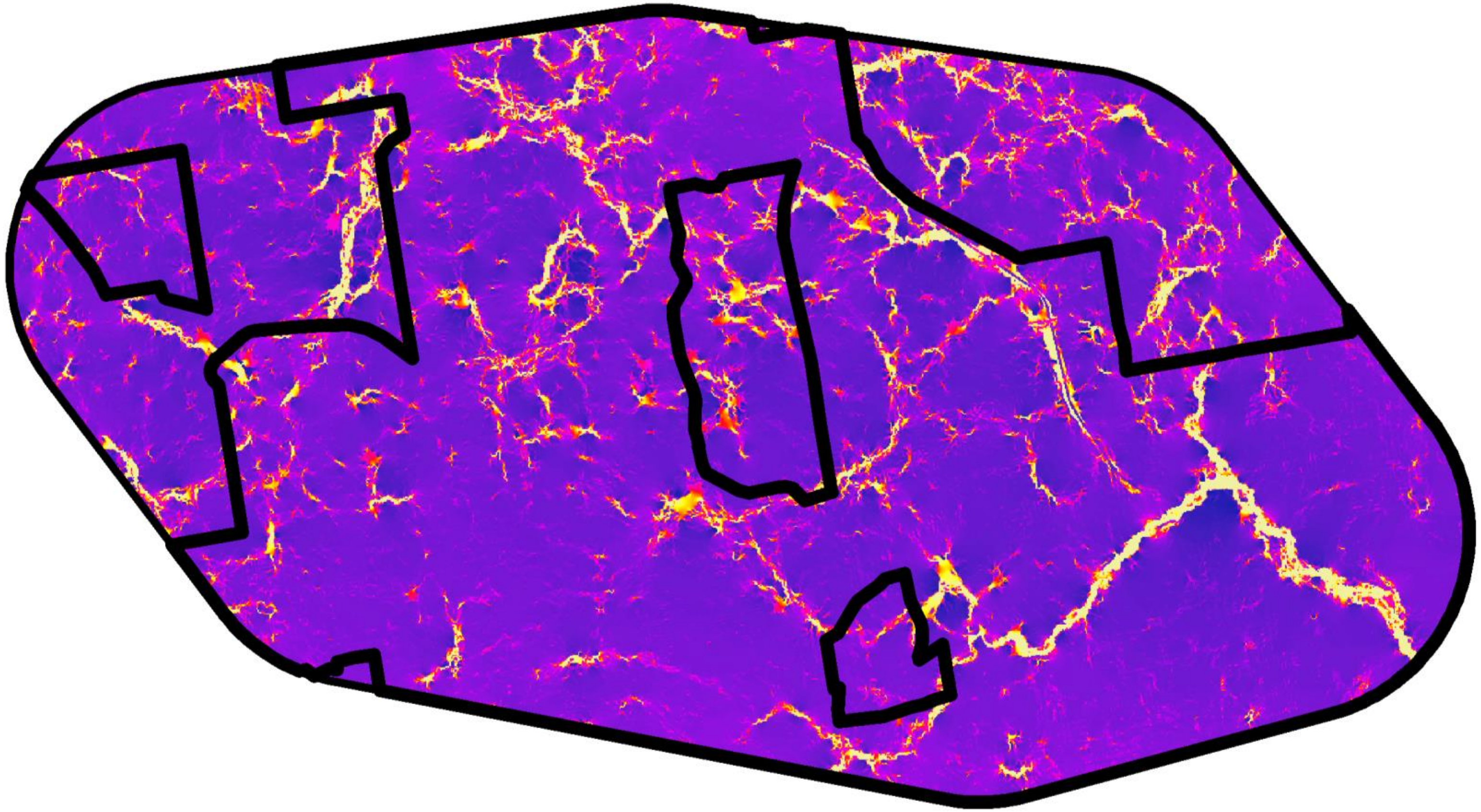

Supplementary Figure 89. Habitat network map for Spotted Turtles (*Clemmys guttata*) in Detroit North site showing habitat patch size as the size of the circle and importance of the patch in maintaining overall network connectivity with warming colors (orange and red) indicating highest importance.

Supplementary Figure 90. Map of locations where removal of a barrier (red areas) would improve connectivity for Spotted Turtles (*Clemmys guttata*) in the Detroit North site.

Supplementary Figure 91. Map of areas with narrow linkages (yellow and red areas) where Spotted Turtles (*Clemmys guttata*) would have limited movement options in Detroit North site making them important corridors to maintain.

Supplementary Figure 92. Current density map for Spotted Turtles (*Clemmys guttata*) in Detroit North site. Areas with higher current density are predicted to represent better movement corridors.

S89

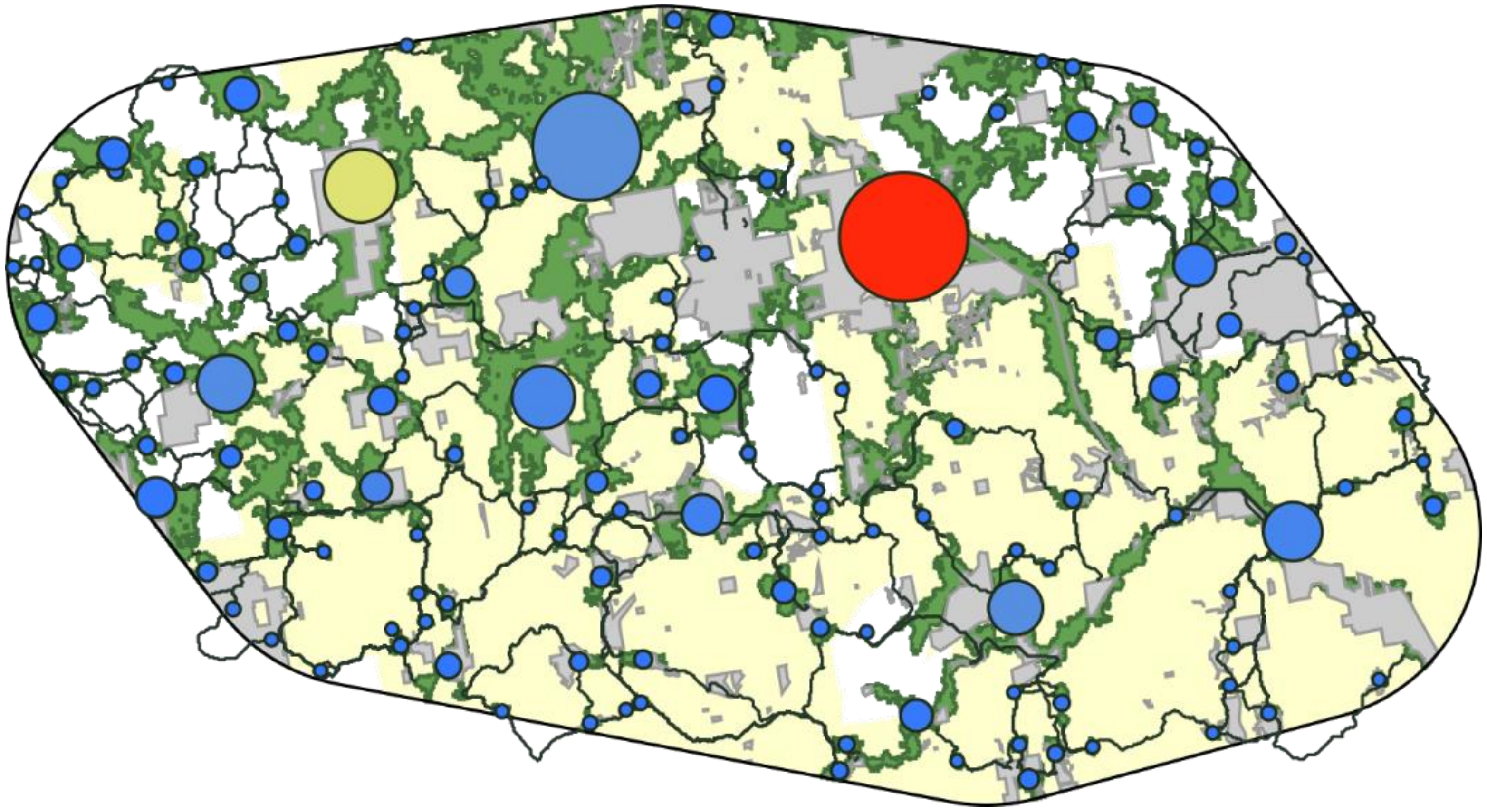

S90

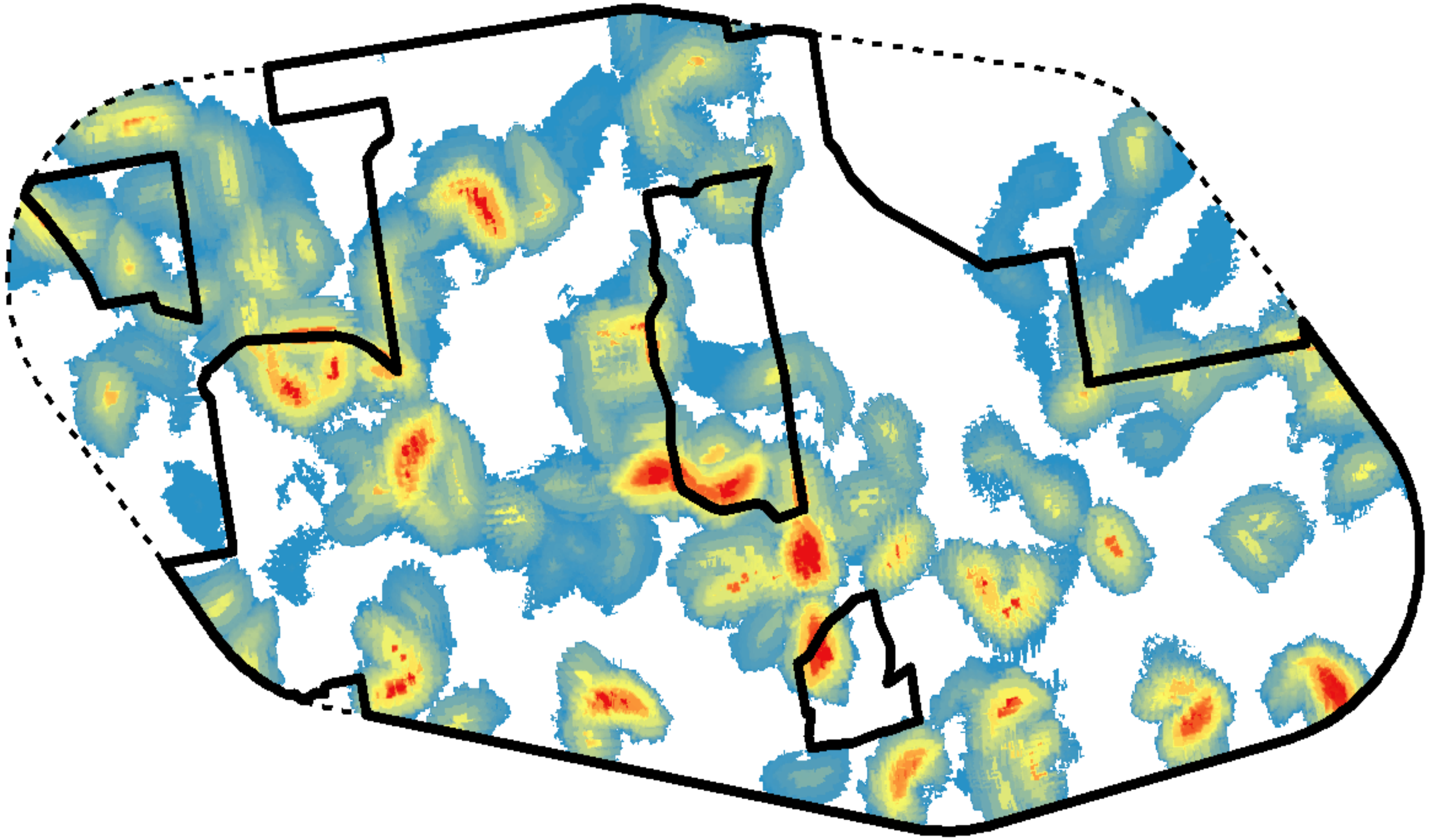

S91

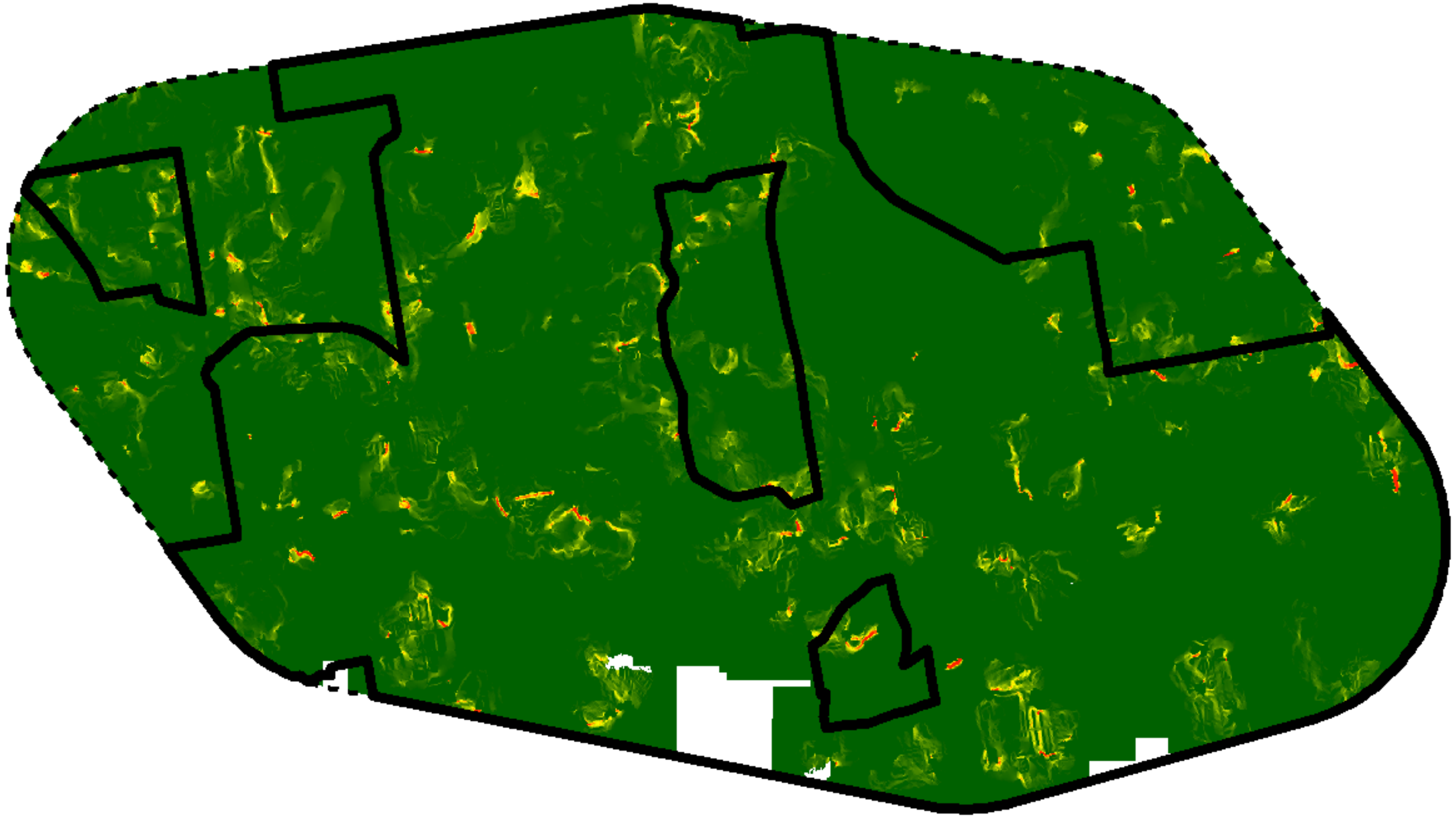

S92

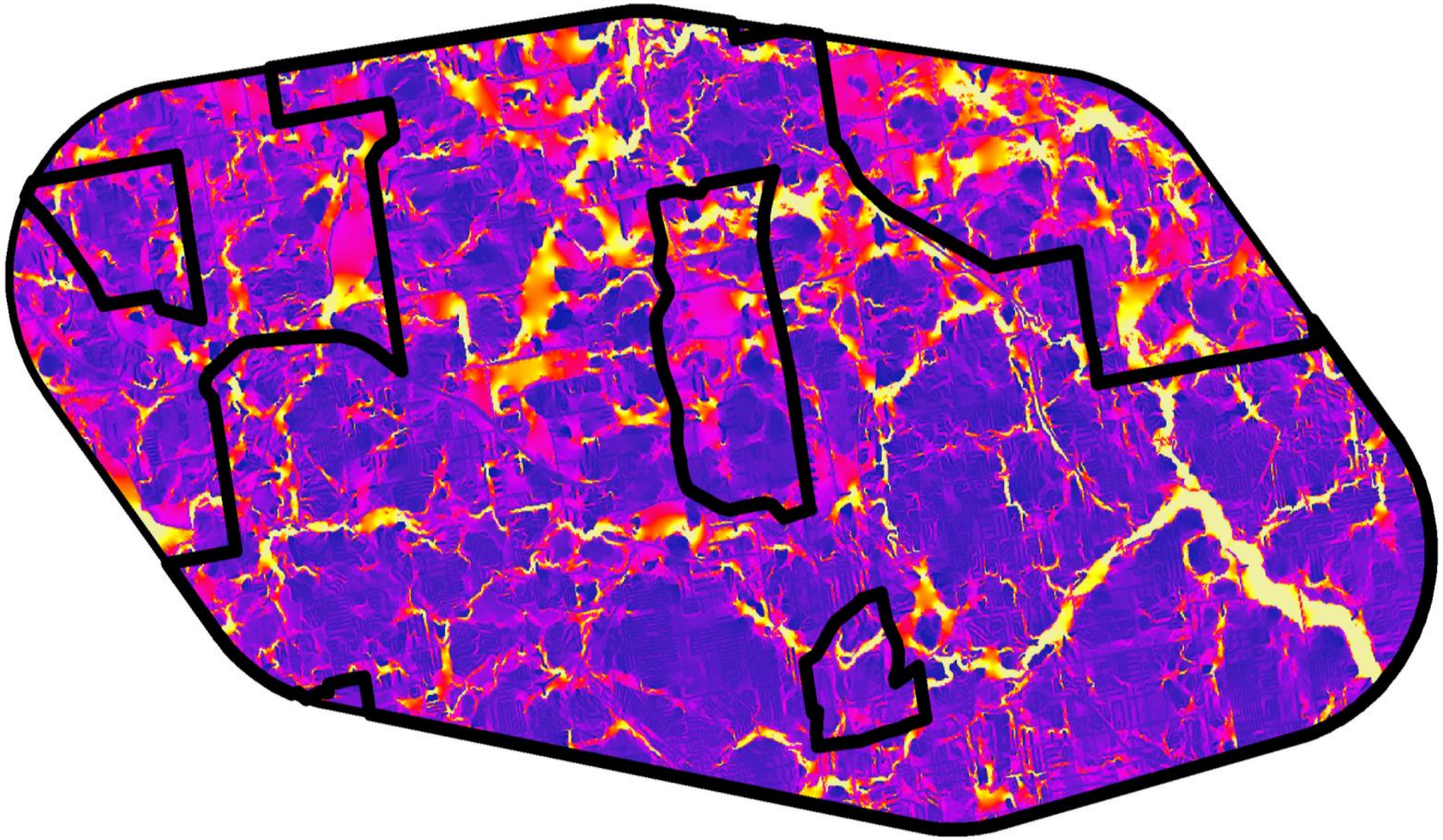

Supplementary Figure 93. Multi-species least cost path (LCP) map for Benton Harbor showing the LCP overlap for two and three species respectively.

Supplementary Figure 94. Multi-species least cost path (LCP) map for Lansing showing the LCP overlap for two species.

Supplementary Figure 95. Multi-species least cost path (LCP) map for Detroit South showing the LCP overlap for two species.

Supplementary Figure 96. Multi-species least cost path (LCP) map for Detroit Southwest showing the LCP overlap for two and three species respectively.

Supplementary Figure 97. Multi-species least cost path (LCP) map for Detroit West showing the LCP overlap for two species.

Supplementary Figure 98. Multi-species least cost path (LCP) map for Detroit North showing the LCP overlap for two, three, and four species respectively.

S93

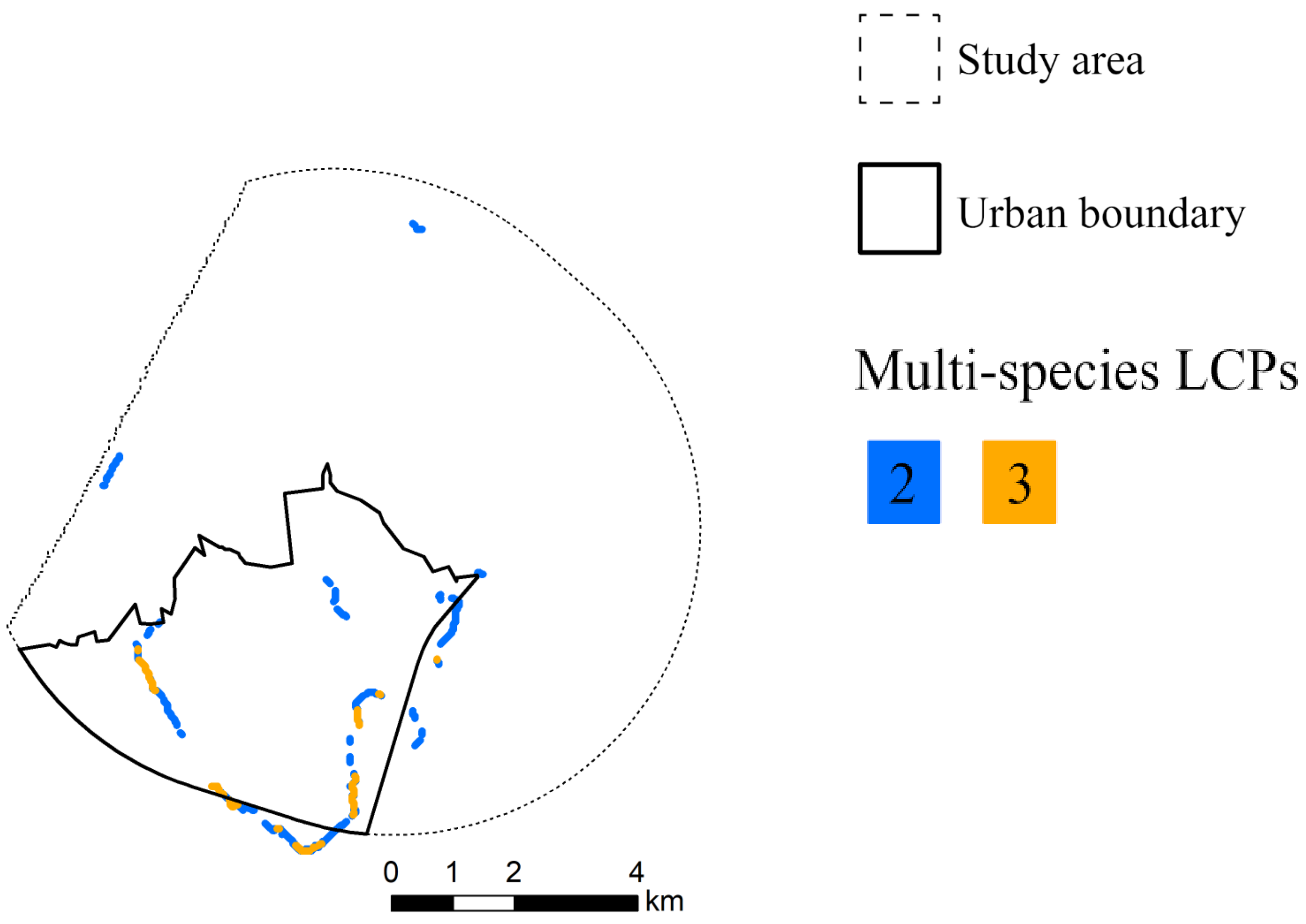

S94

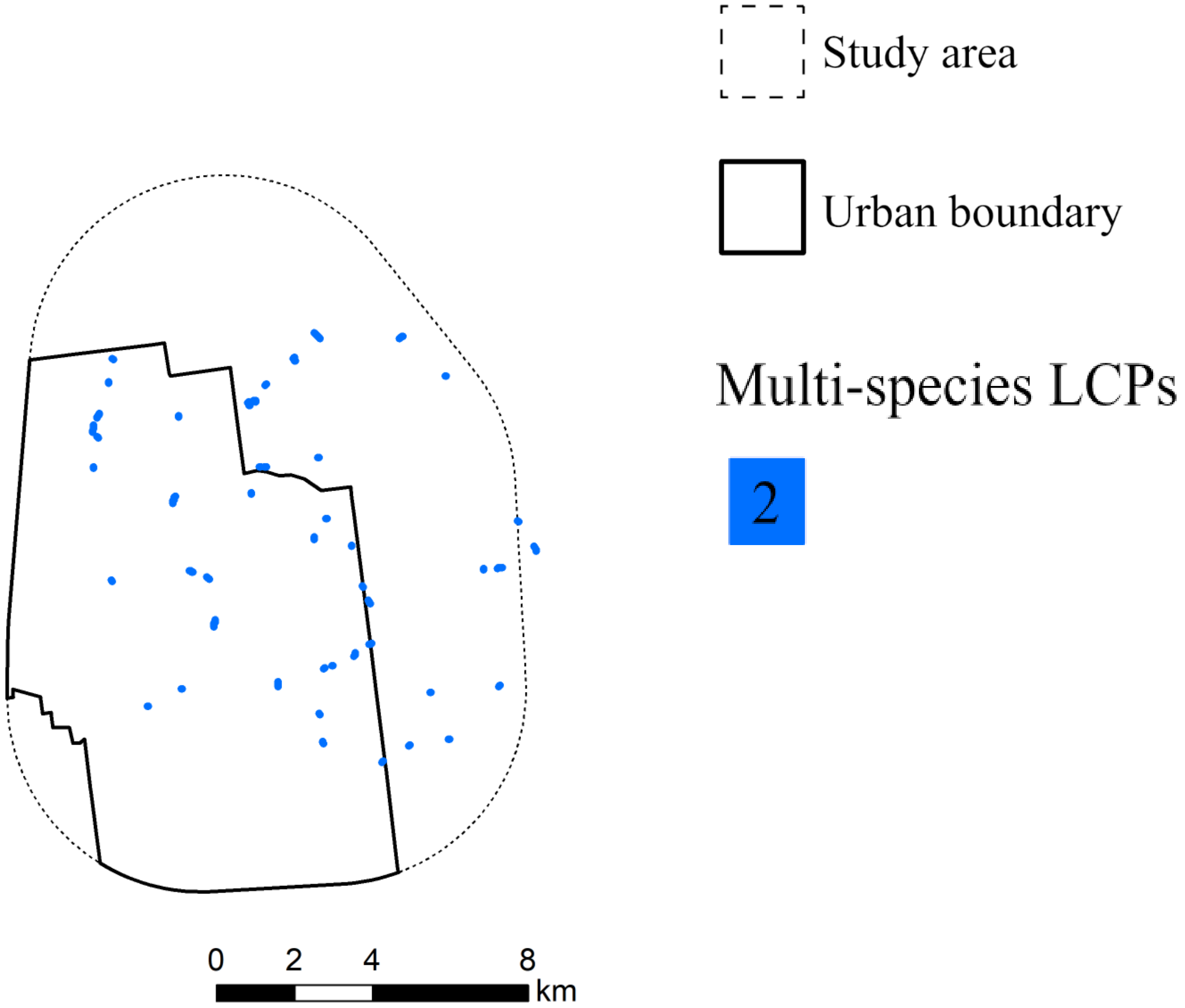

S95

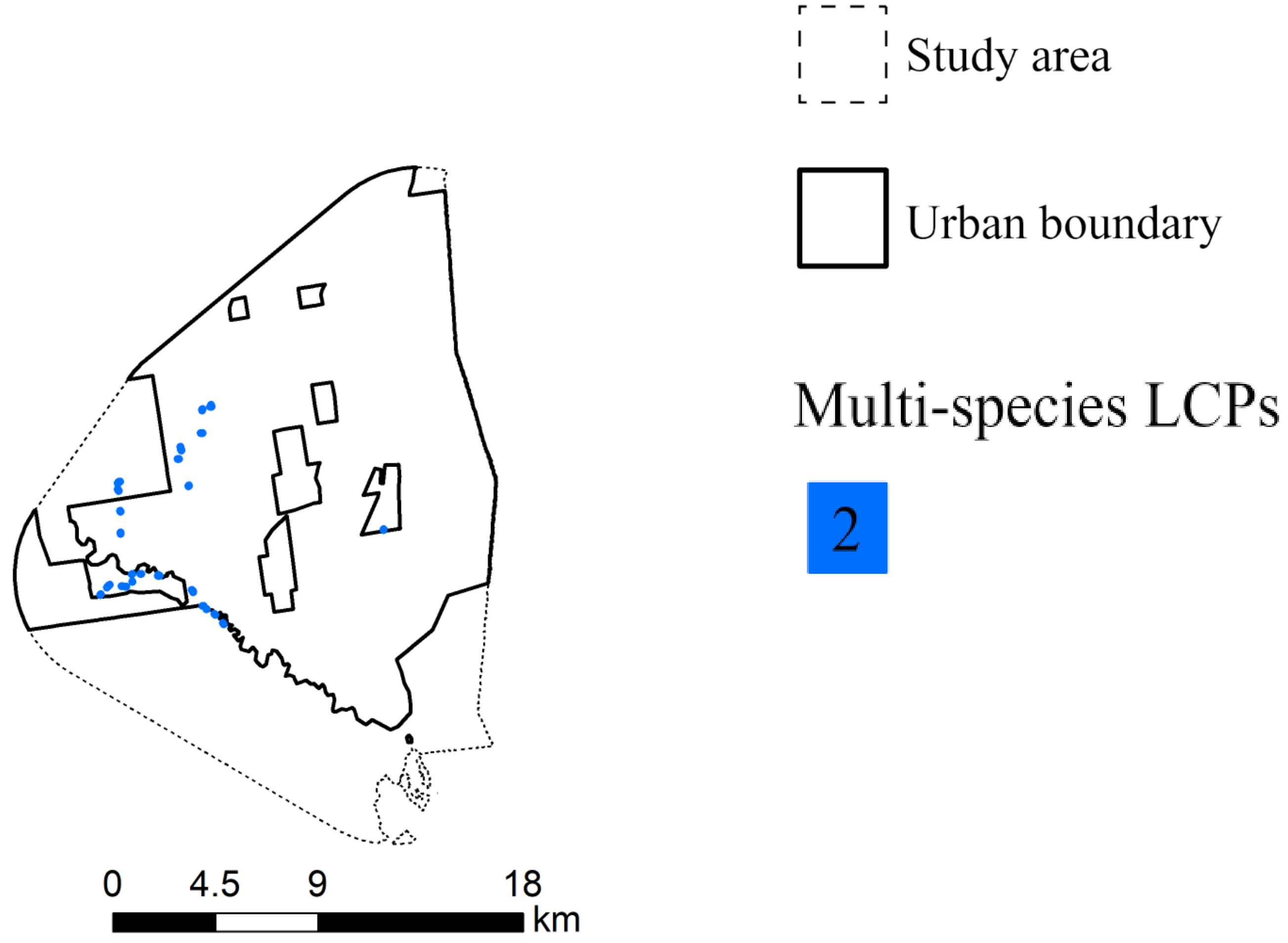

S96

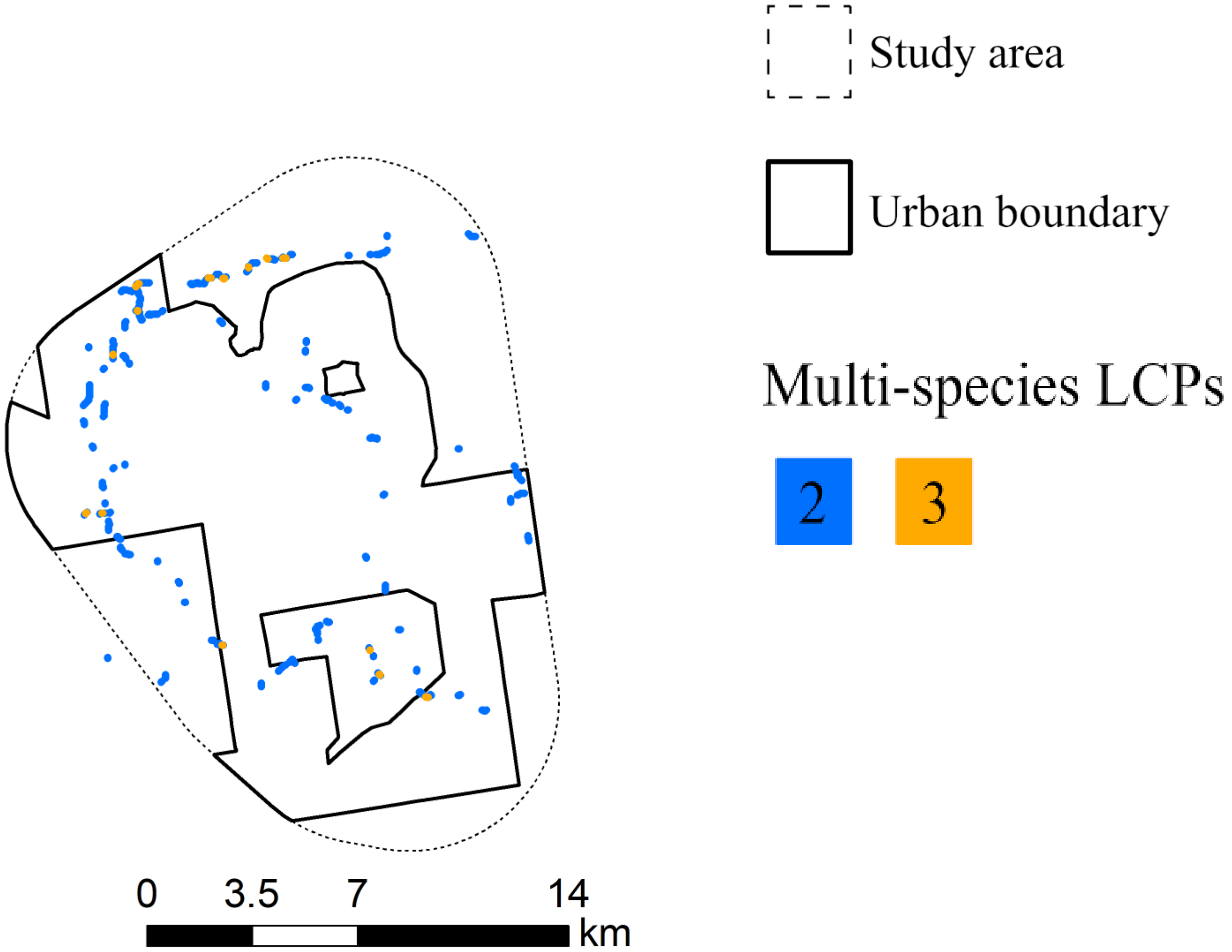

S97

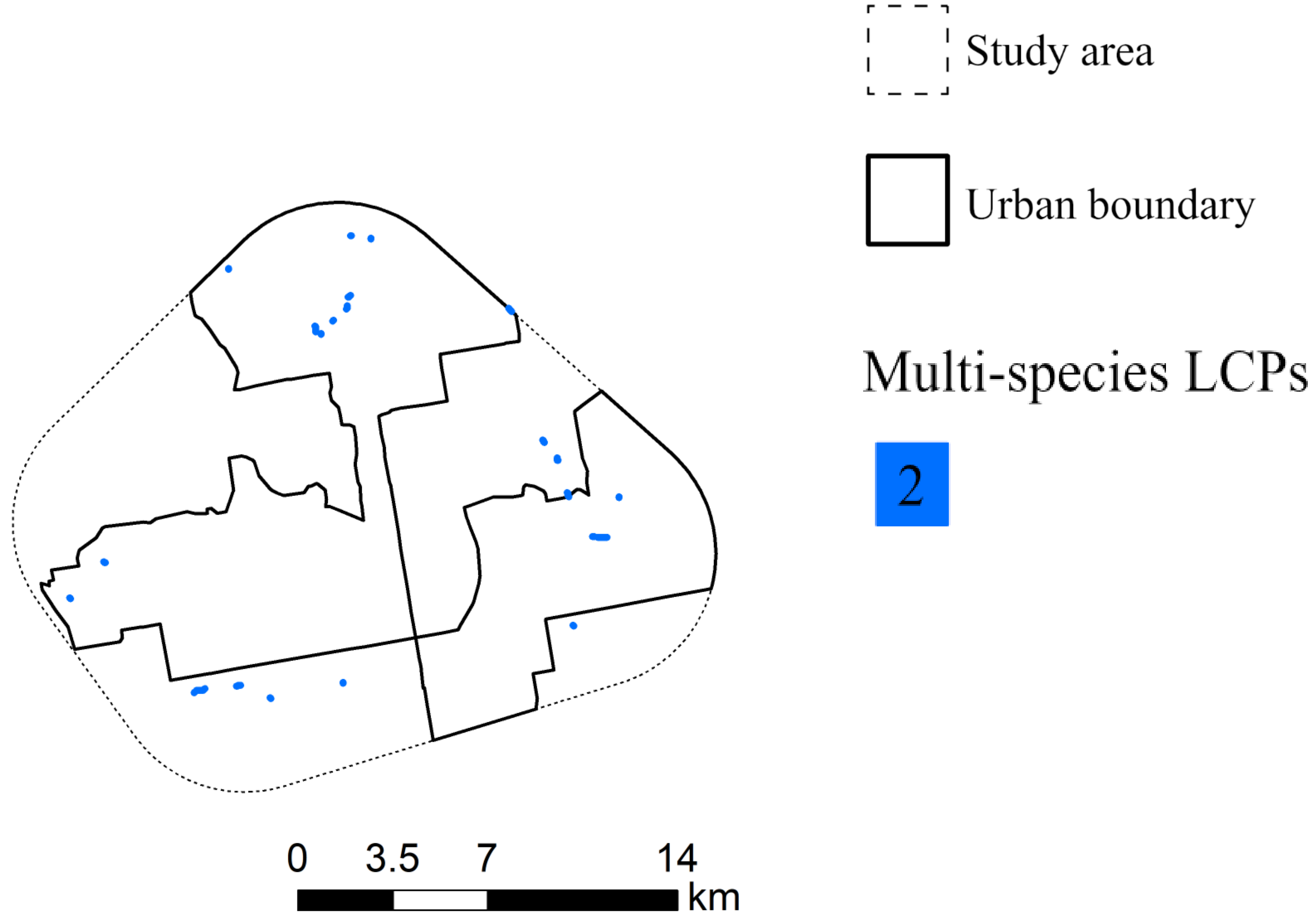

S98

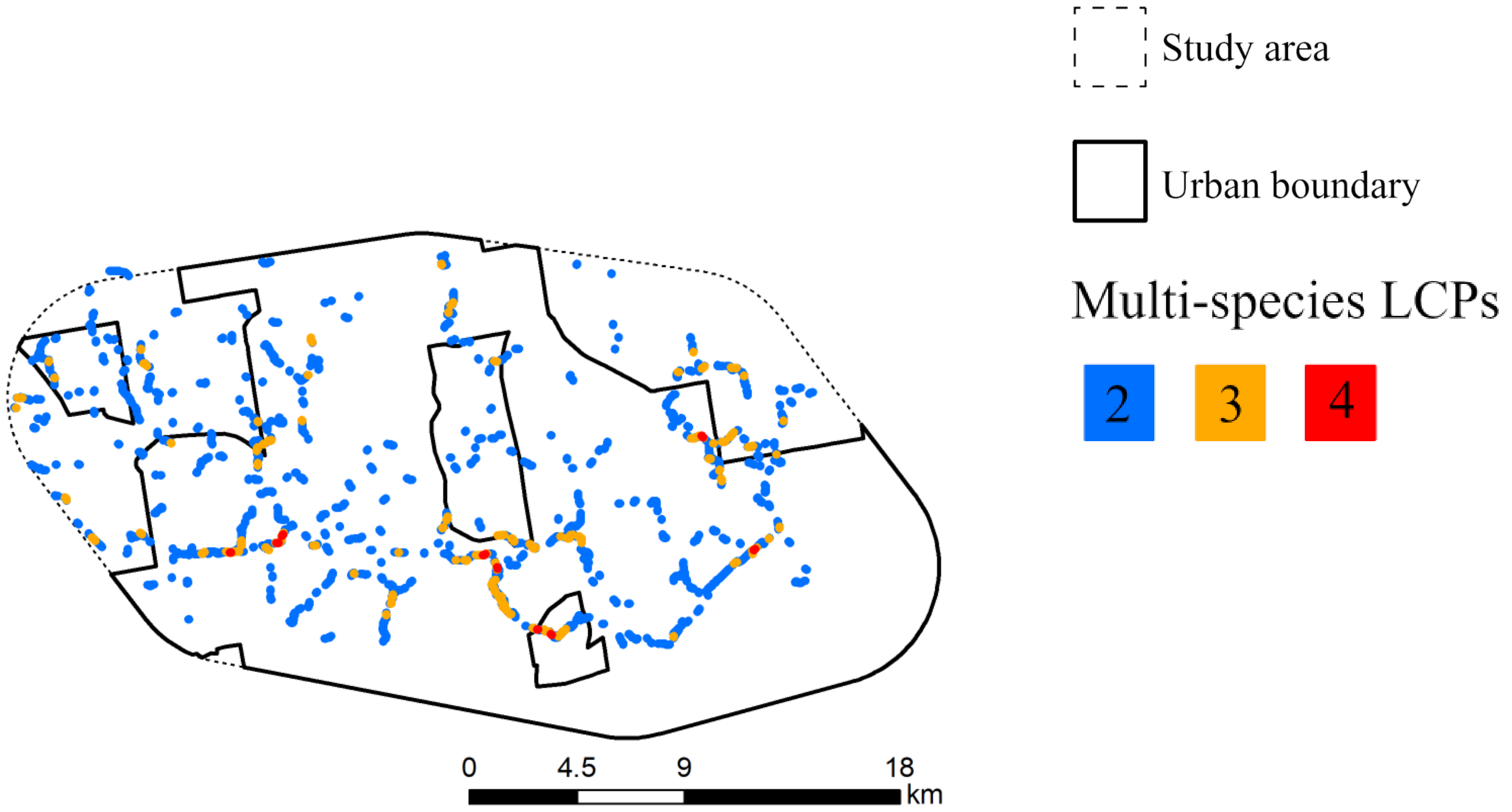

Supplement: Supplementary file 1 — Figures S1–S98 [file ECE3-14-e11105-s002.zip › ece31105-sup-0002-FigureS49-S98.pdf]
