# Supplementary material for: Assessing habitat connectivity of rare species to inform urban conservation planning
Source: Ecol Evol. 2024 Mar 4;14(3):e11105. doi: 10.1002/ece3.11105 (PMC10912553; doi:10.1002/ece3.11105)
Supplement: Supplementary file 2 — Appendix S1 [file ECE3-14-e11105-s001.docx]

Table 1. Model metrics for each final species distribution model (SDM) including number of occurrences included in model (N), SDM type, scores from several metrics (Boyce Index, Area under the curve (AUC), SomersD, true skill statistic (TSS)), and the maximum sum of sensitivity and specificity (MSSS) threshold used for ecospat models.

| Species | N | SDM Type | Boyce Index | AUC | SomersD | TSS | MSSS Threshold |
| --- | --- | --- | --- | --- | --- | --- | --- |
| American/Black and Gold Bumble Bee | 29 | ANN | 1.00 | 0.98 | 0.96 | 0.87 | 0.57 |
|  |  | Maxent | 0.96 | 0.89 | 0.78 | 0.66 | 0.58 |
|  |  |  |  |  |  |  |  |
| Blanding’s Turtle | 49 | ANN | 0.95 | 0.90 | 0.80 | 0.66 | 0.49 |
|  |  | Maxent | 0.93 | 0.86 | 0.72 | 0.59 | 0.46 |
|  |  |  |  |  |  |  |  |
| Eastern Box Turtle | 45 | ANN | 0.86 | 0.92 | 0.83 | 0.70 | 0.52 |
|  |  | Maxent | 0.97 | 0.85 | 0.70 | 0.55 | 0.43 |
|  |  |  |  |  |  |  |  |
| Eastern Massasauga | 152 | ANN |  | 0.88 |  | 0.61 |  |
|  |  | Maxent |  | 0.85 |  | 0.56 |  |
|  |  | RF |  | 1.00 |  | 0.99 |  |
|  |  |  |  |  |  |  |  |
| Eastern Foxsnake | 29 | ANN | 0.94 | 0.97 | 0.94 | 0.84 | 0.48 |
|  |  | Maxent | 0.94 | 0.92 | 0.84 | 0.70 | 0.47 |
|  |  |  |  |  |  |  |  |
| Henslow's Sparrow | 34 | ANN | 0.93 | 0.94 | 0.89 | 0.74 | 0.46 |
|  |  | Maxent | 0.97 | 0.88 | 0.76 | 0.61 | 0.62 |
|  |  |  |  |  |  |  |  |
| Northern Long-eared Bat | 56 | ANN | 0.99 | 0.86 | 0.73 | 0.59 | 0.53 |
|  |  | Maxent | 0.99 | 0.83 | 0.66 | 0.52 | 0.50 |
|  |  |  |  |  |  |  |  |
| Spotted Turtle | 45 | ANN | 0.95 | 0.92 | 0.84 | 0.68 | 0.48 |
|  |  | Maxent | 0.82 | 0.89 | 0.78 | 0.62 | 0.35 |

Table 2. Candidate predictor variables screened for use in the ensemble of small models implemented in ecospat.

| **Type** | **Scale** | **Source** |
| --- | --- | --- |
|  |  |  |
| 10 Class Canopy mean | 100 m | NLCD Canopy Cover |
|  | 200 m |  |
|  |  |  |
| Canopy Standard Deviation | 50 m | NLCD Canopy Cover |
|  | 200 m |  |
|  | 500 m |  |
|  |  |  |
| Canopy range | 100 m | NLCD Canopy Cover |
|  | 200 m |  |
|  | 500 m |  |
|  |  |  |
| Canopy x Canopy STD | 500 m | NLCD Canopy Cover |
|  |  |  |
|  |  |  |
| **Soil** |  | SSURGO |
| **Distance Variables** |  |  |
| Poorly drained soil |  |  |
|  |  |  |
|  |  |  |
| **Digital Elevation Model** |  |  |
| Elevation |  |  |
| Topographic Position Index | 500 m |  |
|  | 1 km |  |
| Compound Topographic Index | |  |
|  |  |  |
| **Land Cover** |  |  |
| Percentage Variables |  |  |
| Agriculture | 25 ha | CCAP; Zonal Metrics |
|  | 50 ha |  |
|  | 75 ha |  |
|  | 100 ha |  |
|  | 150 ha |  |
|  |  |  |
|  |  |  |
| Emergent Wetland | 5 ha | CCAP; Zonal Metrics |
|  | 10 ha |  |
|  | 25 ha |  |
|  | 50 ha |  |
|  | 75 ha |  |
|  | 100 ha |  |
|  | 150 ha |  |
|  | 200 ha |  |
|  | 250 ha |  |
|  | 500 ha |  |
|  | 750 ha |  |
|  | 2000 ha |  |
|  |  |  |
| Forest | 5 ha | CCAP; Zonal Metrics |
|  | 10 ha |  |
|  | 25 ha |  |
|  | 50 ha |  |
|  | 75 ha |  |
|  | 100 ha |  |
|  | 150 ha |  |
|  | 200 ha |  |
|  | 250 ha |  |
|  | 500 ha |  |
|  | 2000 ha |  |
|  |  |  |
| Forest Connectance | 100 ha | CCAP; Zonal Metrics |
|  | 500 ha |  |
|  |  |  |
| Forested wetland | 5 ha | CCAP; Zonal Metrics |
|  | 10 ha |  |
|  | 25 ha |  |
|  | 50 ha |  |
|  | 75 ha |  |
|  | 100 ha |  |
|  | 150 ha |  |
|  | 200 ha |  |
|  | 250 ha |  |
|  | 500 ha |  |
|  | 2000 ha |  |
|  |  |  |
| Grassland | 25 ha | CCAP; Zonal Metrics |
|  | 50 ha |  |
|  | 75 ha |  |
|  | 100 ha |  |
|  | 150 ha |  |
|  |  |  |
| Shannon Habitat Diversity | 25 ha | CCAP; Zonal Metrics |
|  | 100 ha |  |
|  | 500 ha |  |
|  |  |  |
| Pasture | 25 ha | CCAP; Zonal Metrics |
|  | 50 ha |  |
|  | 75 ha |  |
|  | 100 ha |  |
|  | 150 ha |  |
|  |  |  |
| Scrub Shrub | 5 ha | CCAP; Zonal Metrics |
|  | 10 ha |  |
|  | 25 ha |  |
|  | 50 ha |  |
|  | 75 ha |  |
|  | 100 ha |  |
|  | 150 ha |  |
|  | 200 ha |  |
|  | 250 ha |  |
|  | 500 ha |  |
|  | 2000 ha |  |
|  |  |  |
| Scrub Shrub Wetland | 5 ha | CCAP; Zonal Metrics |
|  | 10 ha |  |
|  | 25 ha |  |
|  | 50 ha |  |
|  | 75 ha |  |
|  | 100 ha |  |
|  | 150 ha |  |
|  | 200 ha |  |
|  | 250 ha |  |
|  | 500 ha |  |
|  | 2000 ha |  |
|  |  |  |
| Wetland Connectance | 500 ha | CCAP; Zonal Metrics |
|  | 1000 ha |  |
|  |  |  |
| Shannon Wetland Diversity | 10 ha | CCAP; Zonal Metrics |
|  | 25 ha |  |
|  | 250 ha |  |
|  | 500 ha |  |
|  | 1000 ha |  |
|  |  |  |
|  |  |  |
|  |  |  |
| **Distance Variables** |  |  |
| Open Water |  | NLCD |
|  |  |  |
|  |  |  |

Table 3. Candidate predictor variables screened for use in the biomd2 Eastern Massasauga models.

| **Type** | **Scale** | **Source** |
| --- | --- | --- |
| **Forest** |  |  |
|  |  |  |
| Canopy |  | NLCD Canopy Cover |
| 4 Class Canopy |  | NLCD Canopy Cover |
| 10 Class Canopy mean | 100 m | NLCD Canopy Cover |
|  | 200 m |  |
|  |  |  |
| Canopy Standard Deviation | 50 m | NLCD Canopy Cover |
|  | 200 m |  |
|  | 500 m |  |
| Canopy range | 100 m |  |
|  | 200 m |  |
|  | 500 m |  |
|  |  |  |
|  |  |  |
| **Percentage Variables** |  |  |
| Forest Disturbance All | 150 m | NLCD Science Products |
|  | 300 m |  |
|  | 1500 m |  |
|  | 3000 m |  |
| Forest Disturbance Within 0_10 Years | 300 m | NLCD Science Products |
|  | 1500 m |  |
| Forest Disturbance Within 10_20 Years | 300 m | NLCD Science Products |
|  | 1500 m |  |
| Forest Disturbance Within 20_30 Years | 300 m | NLCD Science Products |
|  | 1500 m |  |
| **Distance Variables** |  |  |
| Forest Disturbance All |  | NLCD Science Products |
| Forest Disturbance Within 0_10 Years |  | NLCD Science Products |
| Forest Disturbance Within 10_20 Years |  | NLCD Science Products |
| Forest Disturbance Within 20_30 Years |  | NLCD Science Products |
|  |  |  |
| **Land Cover** |  |  |
| **Percentage Variables** |  |  |
| Agriculture | 300 m | CCAP |
|  | 1500 m |  |
| Coniferous Forest | 150 m | CCAP |
|  | 300 m |  |
|  | 750 m |  |
|  | 1500 m |  |
|  | 3 km |  |
| Deciduous Forest | 150 m | CCAP |
|  | 300 m |  |
|  | 750 m |  |
|  | 1500 m |  |
|  | 3 km |  |
| All Forest | 150 m | CCAP |
|  | 300 m |  |
|  | 750 m |  |
|  | 1500 m |  |
|  | 3 km |  |
| Emergent Wetland | 300 m | CCAP |
|  | 1500 m |  |
| Forested Wetland | 300 m | CCAP |
|  | 1500 m |  |
| Scrub Wetlands | 300 m | CCAP |
|  | 1500 m |  |
| All Wetlands | 300 m |  |
|  | 1500 m |  |
| **Distance Variables** |  |  |
| Open Water |  | NLCD |
| Wetlands |  | NWI |
| Roads |  | Tiger Line |
|  |  |  |
|  |  |  |
|  |  |  |
|  |  |  |
| **Soil** |  | SSURGO |
| Hydric rating |  |  |
| Max hydric rating within distance | 100 m |  |
|  | 500 m |  |
| Mean hydric rating within distance | 100 m |  |
|  | 500 m |  |
|  |  |  |
| **Distance Variables** |  |  |
| Poorly drained soil |  |  |
|  |  |  |
| **Percentage Variables** |  |  |
| Poorly drained soil | 150 m |  |
|  | 300 m |  |
|  | 750 m |  |
|  | 1500 m |  |
|  | 3 km |  |
|  |  |  |
| **Digital Elevation Model** |  |  |
| Elevation |  |  |
| Topographic Position Index | 500 m |  |
|  | 1 km |  |
| Compound Topographic Index |  |  |
| Roughness | 100 m |  |
|  | 500 m |  |
|  | 1 km |  |
|  |  |  |
| **Wetland Potential** |  | NOAA |
| Average Wetness Potential | 100 m |  |
|  | 500 m |  |
|  | 1 km |  |
| Majority Wetland Value | 100 m |  |
|  | 500 m |  |
|  | 1 km |  |
|  |  |  |
| **Distance Variables** |  |  |
| Wetland potential highest non-open water rating (8) |  |  |

Table 4. Final variables selected for each species distribution model (SDM) with accompanying biological justification for inclusion in final SDM.

| Species | Model Variables | Justification |
| --- | --- | --- |
| American/Black and Gold Bumble Bee | Canopy 10 class mean 100 m | Intermediate canopy cover was associated with habitat. Potential for more plant diversity. |
|  | Canopy std 200 | More variation in canopy was associated with habitat. Potential for more plant diversity. |
|  | Emergent wetland 150 ha | Emergent wetlands are used for foraging activities |
|  | Forest 200 ha | Both species are known to forage along forest edges |
|  | Forest wetland 2000 ha | Higher percentage of forested wetland was associated with better habitat, could reflect more natural areas in general |
|  | Scrub shrub 150 ha | Higher percentage of scrub shrub was associated with better habitat, could reflect more natural areas in general |
| Blanding’s Turtle | Emergent wetland 50 ha | Species is known to use emergent wetland habitats |
|  | Forest 500 ha | Turtles make frequent terrestrial movements through forest habitats |
|  |  |  |
|  | Forest wetland 250 ha | Species is known to use forested wetland habitats |
|  | Simpsons diversity habitat 25 ha | Habitat was associated with more habitat heterogeneity |
|  | Scrub shrub 100 ha | Species is known to use scrub shrub habitats |
|  | Scrub wetland 5 ha | Species is known to use scrub wetland habitats |
|  | Topographic position index 1 km | Lower values indicate lower elevation areas where their preferred wetland habitats are likely to be present |
| Eastern Box Turtle | Canopy x Canopy std 500 m | Intermediate levels of product of canopy and canopy std were best. Species uses edge habitat. |
|  | Forest 25 ha | Species is known to use forest habitats for foraging and overwintering |
|  | Forest wetland (5 ha, 75 ha, 250 ha) | Species is known to use forested wetland habitats for foraging and thermoregulation |
|  | Scrub wetland 25 ha | Species is known to use scrub wetlands for foraging and thermoregulation |
|  | Topographic position index 1 km | Lower values indicate lower elevation areas where their preferred wetland habitats are likely to be present |
| Eastern Massasauga | 10 class canopy mean value within 200 m | Intermediate canopy provides open areas for basking/foraging with some cover |
|  | Topographic position index 1 km | Lower values indicate lower elevation areas where their preferred wetland habitats are likely to be present |
|  | Canopy range 200 m | Higher range of canopy is associated with open areas with dispersed trees/shrubs that offer cover |
|  | Poorly drained soil percentage (150 m, 750 m) | Indicative of the wetland habitats these snakes occupy |
|  | Scrub wetland percentage 300 m | Species is known to use scrub wetland habitats |
| Eastern Foxsnake | Compound topographic index | Species frequently uses drier sections of wetland habitats |
|  | Distance to open water | Fox snakes typically occur near water |
|  | Elevation | Species is found in lower elevation areas where wetlands are more prevalent |
|  | Emergent wetland 25 ha | Species is known to inhabit emergent wetland habitats |
|  | Simpsons diversity wetlands 25 ha | Higher diversity of wetland types increases foraging opportunities |
|  | Topographic position index 500 m | Species frequently uses terrestrial areas in wetland habitats |
| Henslow’s Sparrow | Agriculture 25 ha | Species is known to occur in open field habitats |
|  | Distance to open water | Shorter distance to water can provide foraging opportunities |
|  | Forest 5 ha | Species is associated with less forest |
|  | Forest wetland 5 ha | Species is associated with less forested wetland |
|  | Pasture 25 ha | Species is known to use pasture habitats |
|  | Scrub wetland (5 ha, 750 ha) | Species uses scrub habitats for the dense cover they provide |
| Northern Long-eared Bat | Canopy 10 class mean 100 m | More canopy provides roosting habitat |
|  | Canopy range 500 m | More range in canopy creates habitat heterogeneity |
|  | Emergent wetland 50 ha | Flight and foraging activities are often concentrated over wetlands |
|  | Forest wetland 5 ha | Flight and foraging activities are often concentrated over wetlands |
|  | Simpsons diversity wetlands (25 ha, 1000 ha) | Greater diversity of wetlands provides additional foraging opportunities |
| Spotted Turtle | Canopy range 500 m | Greater canopy range is associated with open areas that the species is associated with |
|  | Forest 250 ha | Turtles use forest habitats as travel corridors and for aestivation |
|  | Forest wetland 10 ha | Species is known to use forested wetlands for foraging and aestivating |
|  | Scrub wetland 5 ha | Species is known to use scrub wetlands |
|  | Simpsons diversity wetlands 25 ha | Greater wetland diversity provides more habitat |
|  | Topographic position index 1 km | Lower values indicate lower elevation areas where their preferred wetland habitats are likely to be present |

Table 5. Final variables selected for each species distribution model (SDM). Variable contribution (from ensemble of small models in ecospat) and variable importance (from biomod2) values are shown for all model types.

|  |  | Variable Contribution | | Variable Importance | | |
| --- | --- | --- | --- | --- | --- | --- |
| Species | Model Variables | ANN | Maxent | ANN | Maxent | RF |
| American/Black and Gold Bumble Bee | Canopy 10 class mean 100 m | 1.03 | 1.03 |  |  |  |
|  | Canopy std 200 | 1.40 | 1.12 |  |  |  |
|  | Emergent wetland 150 ha | 0.83 | 0.72 |  |  |  |
|  | Forest 200 ha | 1.16 | 1.25 |  |  |  |
|  | Forest Wetland 2000 ha | 1.57 | 0.97 |  |  |  |
|  | Scrub shrub 150 ha | 0.35 | 0.97 |  |  |  |
| Blanding’s Turtle |  |  |  |  |  |  |
|  | Emergent wetland 50 ha | 0.80 | 0.82 |  |  |  |
|  | Forest 500 ha | 0.81 | 0.85 |  |  |  |
|  | Forest wetland 250 ha | 1.08 | 1.08 |  |  |  |
|  | Shannon diversity habitat 25 ha | 1.11 | 1.07 |  |  |  |
|  | Scrub shrub 100 ha | 0.88 | 0.83 |  |  |  |
|  | Scrub wetland 5 ha | 1.29 | 1.27 |  |  |  |
|  | Topographic position index 1 km | 1.09 | 1.14 |  |  |  |
| Eastern Box Turtle |  |  |  |  |  |  |
|  | Canopy x Canopy std 500 m | 1.27 | 1.33 |  |  |  |
|  | Forest 25 ha | 1.23 | 1.02 |  |  |  |
|  | Forest wetland 5 ha | 0.92 | 0.84 |  |  |  |
|  | Forest wetland 75 ha | 0.91 | 0.97 |  |  |  |
|  | Forest wetland 250 ha | 0.96 | 1.06 |  |  |  |
|  | Scrub wetland 25 ha | 0.79 | 0.86 |  |  |  |
|  | Topographic position index 1 km | 0.97 | 0.96 |  |  |  |
| Eastern Massasauga |  |  |  |  |  |  |
|  | 10 class canopy mean value within 200 m |  |  | 0.19 | 0.05 | 0.16 |
|  | Topographic position index 1 km |  |  | 0.16 | 0.12 | 0.31 |
|  | Canopy range 200 m |  |  | 0.19 | 0.05 | 0.10 |
|  | Poorly drained soil percentage 750 m |  |  | 0.20 | 0.08 | 0.21 |
|  | Scrub wetland percentage 300 m |  |  | 0.44 | 0.30 | 0.48 |
|  | Poorly drained soil percentage 150 m |  |  | 0.16 | 0.05 | 0.19 |
| Eastern Foxsnake |  |  |  |  |  |  |
|  | Compound topographic index | 1.29 | 1.05 |  |  |  |
|  | Distance to open water | 1.07 | 1.11 |  |  |  |
|  | Elevation | 1.07 | 0.99 |  |  |  |
|  | Emergent wetland 25 ha | 0.72 | 0.99 |  |  |  |
|  | Shannon diversity wetlands 25 ha | 1.02 | 0.95 |  |  |  |
|  | Topographic position index 500 m | 0.90 | 0.91 |  |  |  |
| Henslow's Sparrow |  |  |  |  |  |  |
|  | Agriculture 25 ha | 0.87 | 0.90 |  |  |  |
|  | Distance to open water | 0.52 | 0.75 |  |  |  |
|  | Forest 5 ha | 1.37 | 1.45 |  |  |  |
|  | Forest wetland 5 ha | 0.84 | 0.78 |  |  |  |
|  | Pasture 25 ha | 1.72 | 1.44 |  |  |  |
|  | Scrub wetland 5 ha | 0.97 | 0.93 |  |  |  |
|  | Scrub wetland 750 ha | 0.96 | 0.88 |  |  |  |
| Northern Long-eared Bat |  |  |  |  |  |  |
|  | Canopy 10 class mean 100 m | 1.45 | 1.25 |  |  |  |
|  | Canopy range 500 m | 1.11 | 1.09 |  |  |  |
|  | Emergent wetland 50 ha | 0.74 | 0.80 |  |  |  |
|  | Forest wetland 5 ha | 1.00 | 1.11 |  |  |  |
|  | Shannon diversity wetlands 25 ha | 0.83 | 0.88 |  |  |  |
|  | Shannon diversity wetlands 1000 ha | 0.96 | 0.92 |  |  |  |
| Spotted Turtle |  |  |  |  |  |  |
|  | Canopy range 500 m | 0.90 | 0.88 |  |  |  |
|  | Forest 250 ha | 0.90 | 0.96 |  |  |  |
|  | Forest wetland 10 ha | 0.91 | 0.98 |  |  |  |
|  | Scrub wetland 5 ha | 1.15 | 1.07 |  |  |  |
|  | Shannon diversity wetlands 25 ha | 1.10 | 1.07 |  |  |  |
|  | Topographic position index 1 km | 1.07 | 1.05 |  |  |  |

R script for biomod2 runs

library(sp)

library(raster)

library(parallel)

library(reshape)

library(ggplot2)

library(biomod2)

obs <- read.csv(file=.csv)

myRespName <- "emr"

sp_occ <- as.numeric (obs[, myRespName])

xy <- obs[, c('x','y')]

predictors<-stack(rasterfiles)

list <- list.files(path='datafolder', pattern =".tif$", full.names=TRUE)

projection<-stack(list)

myBiomodData <- BIOMOD_FormatingData( resp.var = sp_occ, expl.var = predictors, resp.xy = xy, resp.name = myRespName, PA.nb.rep = 1,

PA.nb.absences = 10000, PA.strategy = "random")

myBiomodOptions <- BIOMOD_ModelingOptions(MAXENT.Phillips = list(

memory_allocated = 2048,

background_data_dir = 'default',

maximumbackground = 'default',

maximumiterations = 200,

visible = FALSE,

linear = TRUE,

quadratic = FALSE,

product = FALSE,

threshold = FALSE,

hinge = FALSE,

lq2lqptthreshold = 80,

l2lqthreshold = 10,

hingethreshold = 15,

beta_threshold = -1,

beta_categorical = -1,

beta_lqp = -1,

beta_hinge = -1,

betamultiplier = 0.5,

defaultprevalence = 0.5),

ANN = list(NbCV=10),

RF = NULL)

myBiomodOptions@MAXENT.Phillips$path_to_maxent.jar <- './'

myBiomodModelOut <- BIOMOD_Modeling( data=myBiomodData,

models=c('MAXENT.Phillips','ANN','RF'),

NbRunEval=10,

DataSplit=80,

models.eval.meth = c('KAPPA','TSS','ROC'),

VarImport=10,

SaveObj = TRUE,

do.full.models = FALSE,

parallel=TRUE,models.options = myBiomodOptions)

get_evaluations(myBiomodModelOut)

myBiomodModelEval <- get_evaluations(myBiomodModelOut)

write.table(myBiomodModelEval, file = ".txt", sep = "\t")

varimpt<-get_variables_importance(myBiomodModelOut)

write.table(varimpt, file = ".txt", sep = "\t")

myBiomodEM<-BIOMOD_EnsembleModeling( modeling.output= myBiomodModelOut,

chosen.models = 'all',

em.by = 'all',

eval.metric = c('ROC'),

eval.metric.quality.threshold = c(0.7),

models.eval.meth = c('KAPPA','TSS','ROC'),

prob.mean = FALSE,

prob.cv = FALSE,

prob.ci = FALSE,

prob.ci.alpha = 0.05,

prob.median = FALSE,

committee.averaging = FALSE,

prob.mean.weight = TRUE,

prob.mean.weight.decay = 'proportional',

VarImport = 10)

get_evaluations(myBiomodEM)

myBiomodEMEval <- get_evaluations(myBiomodEM)

write.table(myBiomodModelEval, file = ".txt", sep = "\t")

varimpt<-get_variables_importance(myBiomodEM)

write.table(varimpt, file = ".txt", sep = "\t")

myBiomodProj <- BIOMOD_Projection(modeling.output = myBiomodModelOut,

new.env = projection,

proj.name = 'current',

selected.models = 'all',

binary.meth = 'ROC',

compress = FALSE,

build.clamping.mask = FALSE,

output.format = '.grd')

myBiomodEF <- BIOMOD_EnsembleForecasting(

EM.output = myBiomodEM,

projection.output = myBiomodProj)

R script for ecospat runs

library(gtools)

library(sp)

library(raster)

library(parallel)

library(reshape)

library(ggplot2)

library(biomod2)

library(ecospat)

library(rgdal)

obs <- read.csv(file=.csv,head=TRUE,sep=",")

myRespName <- "species"

sp_occ <- as.numeric (obs[, myRespName])

xy <- obs[, c('x','y')]

predictors<-stack(rasterfiles)

list <- list.files(path='datafolder', pattern =".tif$", full.names=TRUE)

projection<-stack(list)

myBiomodData <- BIOMOD_FormatingData( resp.var = sp_occ, expl.var = predictors, resp.xy = xy, resp.name = myRespName, PA.nb.rep = 1,

PA.nb.absences = 10000, PA.strategy = "random")

myBiomodOptions <- BIOMOD_ModelingOptions()

myBiomodOptions@MAXENT.Phillips$path_to_maxent.jar <- 'path'

library(doParallel);cl<-makeCluster(4);doParallel::registerDoParallel(cl)

my.ESM <- ecospat.ESM.Modeling( data=myBiomodData,

models=c('MAXENT.Phillips','ANN'),

NbRunEval=10,

DataSplit=80,

weighting.score=c("SomersD"),

models.options=myBiomodOptions,

parallel=TRUE,

tune=TRUE)

my.ESM_EF <- ecospat.ESM.EnsembleModeling(my.ESM,weighting.score=c("SomersD"),threshold=0)

my.ESM_proj_current<-ecospat.ESM.Projection(ESM.modeling.output=my.ESM,

new.env=projection)

my.ESM_EFproj_current <- ecospat.ESM.EnsembleProjection(ESM.prediction.output=my.ESM_proj_current,

ESM.EnsembleModeling.output=my.ESM_EF)
